# Uncovering and understanding “transesterification triggers” in aliphatic polyester synthesis with bis-Zn catalysts

**DOI:** 10.1039/d6py00814c

**Published:** 2026-11-11

**Authors:** Federica Tufano, Jack W. J. Hughes, Thomas J. Neal, Karen Radetzky, Marina Lamberti, Jennifer A. Garden

**Affiliations:** a School of Chemistry, The University of Edinburgh Edinburgh EH9 3FJ UK j.garden@ed.ac.uk; b Department of Chemistry and Biology “Adolfo Zambelli” University of Salerno via Giovanni Paolo II 132 84084 Fisciano Salerno Italy

## Abstract

On paper, the generation of block copolymers through sequential copolymerisation of two lactones should be straightforward when using a living polymerisation. In practice, this is often not the case, and random copolymer architectures can occur due to transesterification processes. The ability to predict whether certain lactone monomer combinations will deliver block or random copolymers is currently missing. Through a systematic study of monomer features, this work uncovers the impact of monomer substituents, ester functional group density, Lewis basicity, and chelation potential, to identify the key factors that favour or disfavour transesterification. This work uncovers a general trend where the most Lewis basic monomers promote transesterification. By demonstrating that the effective Lewis basicity can be assigned using ^19^F NMR probe studies, and intrinsic Lewis basicity can be assigned *via*^13^C NMR and FT-IR analysis, this work generates understanding, and in some cases enables prediction, of whether transesterification will occur or not.

## Abstract 2

Sequential polymerisation of lactones to block copolymers should be straightforward, yet this is not always the case. Here, monomer properties are correlated to polymer architecture, examining the balance between propagation and transesterification.

## Graphical abstract

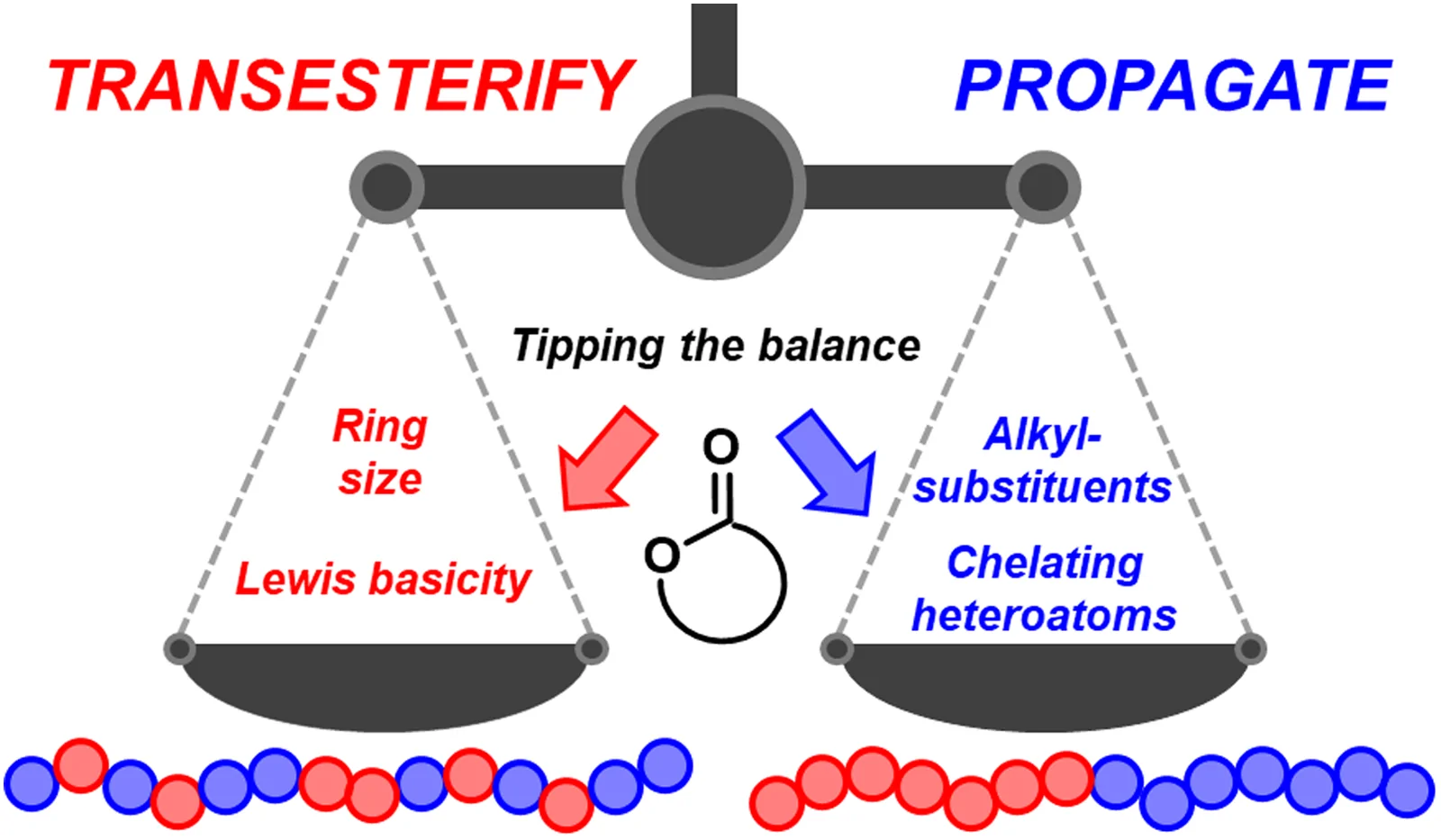

## Introduction

Aliphatic polyesters have attracted increasing attention as potential replacements for conventional fossil-derived plastics, due to their structural versatility and degradability arising from the cleavable ester bonds.^1^ These materials are typically synthesised *via* the metal-catalysed ring-opening polymerisation (ROP) of lactones,^2–4^ yet their large-scale applications remain somewhat restricted by inherent material property limitations. For example, polylactic acid (PLA) is an attractive bioderived, biocompatible and biodegradable thermoplastic^2,5,6^ but its brittle nature and poor thermal stability prevent its use in applications that require high strength or durability.^7^ The synthesis of copolymers is an effective method to overcome these limitations, combining beneficial properties from each homopolymer to tailor the thermal, mechanical, and degradation properties.^8,9^ Block copolymers (BCPs) of lactide (LA) and ε-caprolactone (CL) overcome the brittleness and inflexibility of PLA,^10^ and lactone-derived copolymers are sold as synthetic absorbable sutures marketed as VICRYL™, MAXON™, MONOCRYL™, and BIOSYN™.^11,12^ As material properties are inherently linked to copolymer microstructures, controlling the monomer sequence distribution is highly desirable. This can be challenging with aliphatic polyesters, due to transesterification side-reactions.^13,14^

Transesterification is a powerful technique for rearranging monomer sequences in a polyester chain (Fig. 1, top).^15^ Understanding and controlling the dual roles of transesterification—both as a disruptive side reaction and as a valuable synthetic tool for modifying the polymer structure—is crucial for controlling copolymer architectures. Typically promoted by metal catalysts and high temperatures, transesterification can either occur during polymerisation (*i.e.* a “bottom-up” synthesis),^14^ or post-polymerisation (*i.e.* a “top-down” approach).^16,17^ This rearrangement of the polymer structure can occur through intramolecular or intermolecular processes, generating cyclic or linear polyesters respectively. These dynamic bond-breaking and reforming processes can transform BCPs into statistical architectures, modulating the crystallinity and thermal properties (Fig. 1, middle).^18^ Understanding and predicting when transesterification will occur is key to controlling copolymer structure and properties, yet the question of ‘when and why does transesterification occur instead of propagation?’ remains unanswered.

**Fig. 1:**
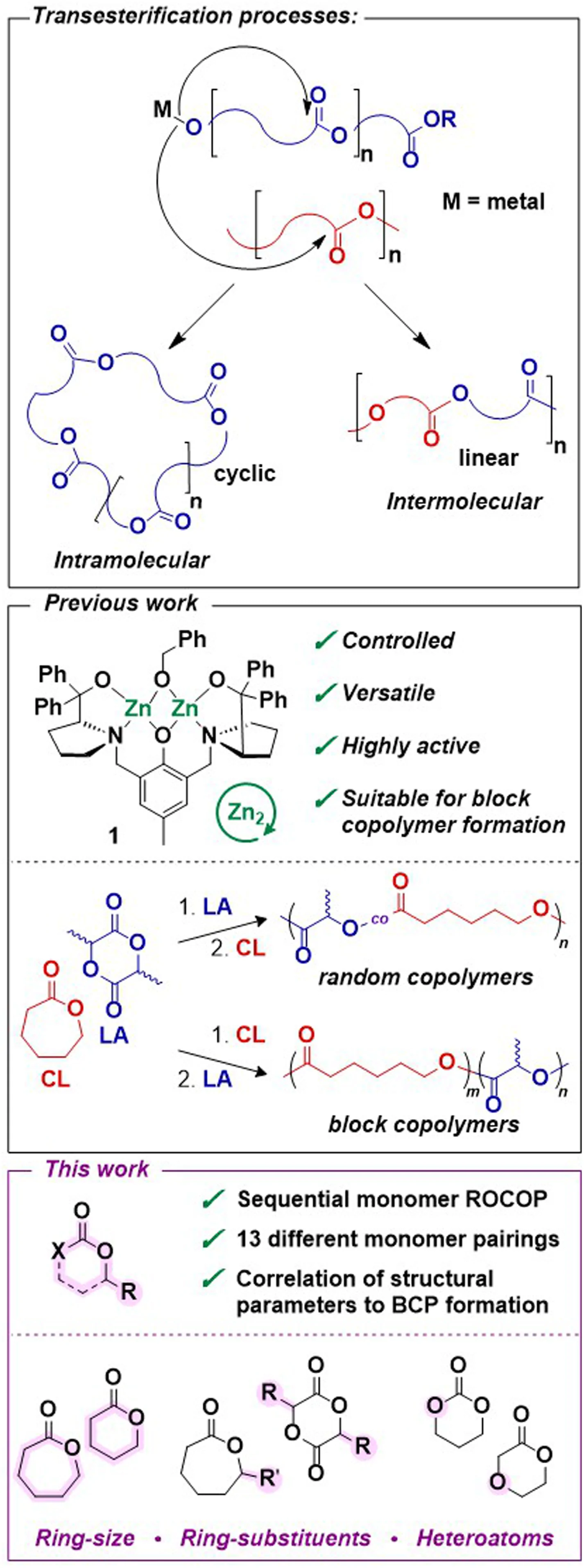
Top: Representation of intramolecular and intermolecular transesterification processes. Middle: Structure of highly active bis-Zn (1) catalyst, capable of sequential lactone ROCOP to give block or random copolymers, depending on the order of monomer addition.^17,34^ Bottom: This work, probing the impact of ring-size, ring substituents and the presence of heteroatoms within the ring. R = Me or H, R′ = ^*n*^Bu or H.

The ‘blocky’ nature of copolymers can be controlled through external stimuli, inherent catalyst features, or the nature of the monomers.^19–27^ It is well-known, yet not well-understood, that transesterification is particularly prevalent when a living PLA* chain is extended by adding ε-caprolactone (CL),^15,17,28–31^ with only a handful of catalysts being able to deliver PLA–PCL BCPs from a living PLA* chain.^29–32^ Yet with other monomer/catalyst combinations, BCPs can be produced regardless of the order of monomer addition.^33^ Some studies have indicated that the addition of a second monomer with reduced ring-strain, and thus lower propagation rates, favours transesterification. However, this alone has not been sufficient to predict whether BCPs or random copolymer structures will be obtained.^17,34–36^

Understanding transesterification in metal-catalysed lactone polymerisation is a complex challenge, as multiple factors may influence the competition between propagation and transesterification, including: Lewis acidity; coordination geometry and steric confinement of the metal;^16^ the steric and electronic profile of the monomer and polymer; monomer ring-strain; propagation rate; the proximity of ester groups in the polymer chain; the binding mode of a propagating polymer chain-end to the catalyst metal centre; and the reaction conditions (*e.g.* temperature and time).^20,22,23,37^ This work systematically interrogates the importance of different lactone monomer features. Using an *in situ* generated bimetallic zinc catalyst (1, Fig. 1), which has previously shown excellent activity and control in the ROP of CL, LA, β-butyrolactone (BL) and δ-valerolactone (VL),^17,34^ this study uncovers the influence of monomer substituents, the distance between ester linkages and the presence of chelating heteroatoms upon transesterification (Fig. 1, bottom).^16,17,34,38^ Through NMR and FT-IR studies, this work also reveals how monomer Lewis basicity can tip the balance between propagation and transesterification, correlating a measurable property with the formation of block or random copolymer structures.

## Results and discussion

### Steric bulk and the presence of ring-substituents

Previous studies have shown that bis-Zn 1 (Fig. 1, middle) is active for the ROP of a range of lactones, and delivered PCL-*block*-PLA from a “PCL first” route (*i.e.* adding LA to a living PCL* chain), but random PLA-*ran*-PCL copolymers when using a “PLA first” route (adding CL to a living PLA* chain).^34^ This was not merely an artefact of reaction time, as with the “PCL first” route, no transesterification occurred even when the reaction was heated for 24 h after the complete polymerisation of LA. In contrast, in the “PLA first” route the addition of CL transesterified the PLA* chain even at low levels of CL conversion. A key difference between propagating PLA and PCL chains is that the PLA chains feature a secondary alkoxide adjacent to the metal centre (Zn–O–*CHMe*–C(O)–PLA), disfavouring transesterification when PLA is the second block. By contrast, PCL chains have a primary alkoxide (Zn–O–*CH*_2_–(CH_2_)_4_–C(O)–PCL), favouring transesterification when PCL is the second block. To probe whether the increased steric bulk of secondary alkoxide chain ends disfavours transesterification, glycolide (GA) was investigated as the unsubstituted analogue of *rac*-lactide (Scheme 1). It was hypothesised that the Zn–alkoxide unit of a propagating poly(glycolide) (PGA) chain, with a primary alkoxide active chain end, would be a better nucleophile than a propagating PLA chain due to decreased steric bulk, facilitating transesterification when PGA was the second block. Throughout the studies reported in this manuscript, the structure of the copolymers was generally analysed at nearly full conversion of the second monomer and as soon as monomer consumption ceased, to ensure that the observed microstructures reflected the kinetic competition during chain extension rather than arbitrary post-polymerisation scrambling.

**Scheme 1:**
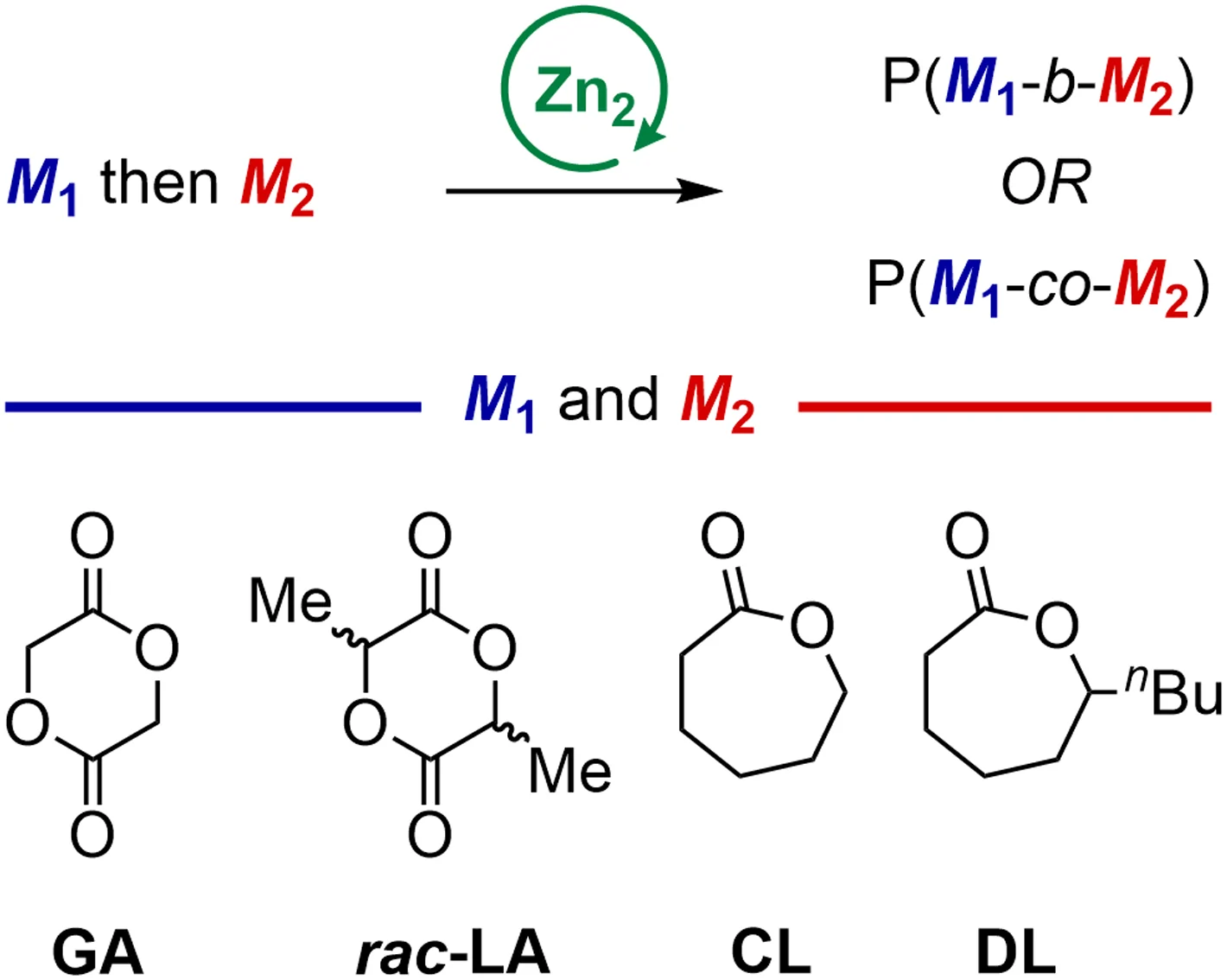
Probing the influence of ring-substituents [glycolide (GA) *versus rac*-lactide (*rac*-LA) and ε-caprolactone (CL) *versus* ε-decalactone (DL)] in copolymerisations with sequential monomer addition.

The order of monomer addition proved essential for the selective synthesis of BCPs (Table 1, entries 1–3 *vs*. entries 4 and 5). Addition of CL first, followed by GA, gave PCL-*block*-PGA structures. This was determined by calculating the average monomer sequence lengths (*l*_GA_ and *l*_CL_), which were in excellent agreement with the equivalents of CL and GA added (entry 1, see SI section S1.3 for details).^39^ The *R* value gives a measure of the randomness of the copolymer chain and here, the *R* value lies close to zero indicating a well-defined blocky structure. *R* values close to 1 imply a randomised copolymer structure, and values above 1 can be observed for diester monomers such as GA or LA, which can be split into glycolic acid or lactic acid units. Supporting the formation of BCPs, ^1^H NMR resonances for both the PCL and PGA blocks were present, with only trace resonances observed for linking units between the PCL and PGA blocks (see Fig. S17–S21 for details). DOSY NMR spectroscopy showed a single diffusion coefficient for the PCL and PGA resonances, providing further evidence for a BCP (Fig. S19). Extending the chain length by using 180 equivalents of CL followed by 20 equivalents of GA maintained well-controlled BCP structures (entry 2, *R* = 0.1). Adding GA to a living PCL* chain thus mimics observations with CL and *rac*-LA, where the addition of *rac*-LA to a living PCL* chain also delivers BCPs, without transesterification.^17^

**Table 1:** Ring-opening copolymerisations of glycolide (GA), ε-caprolactone (CL), *rac*-lactide (LA) and ε-decalactone (DL) using *in situ* generated bis-Zn 1 as the catalyst system

Entry	*M*_1_ (eq.)	*M*_2_ (eq.)	Time (min)		Conv. (%)		Molar comp *M*_1_ : *M*_2_		*M*_n_ (kDa)		*Đ* b	*l* c		*R* c
			*M*_1_	*M*_2_	*M*_1_	*M*_2_	Theo.^a^	NMR	Theo.^a^	Exp.^b^		GA/LA	CL	
1^d^	CL (20)	GA (20)	10	2	99	99	50 : 50	56 : 44	4.6	5.6^e^	—	20	17	0.08
2^d^	CL (180)	GA (20)	30	2	99	99	90 : 10	96 : 4	22.8	34.0	1.8	32	145	0.10
3^d^^,^^f^	GA (20)	CL (20)	5	30	99	95	50 : 50	49 : 51	2.4^h^	—	—	2	3	1.0
4^f^^,^^g^	GA + CL (20) (20)		40		99	35	42 : 58	43 : 57	3.1^h^	—	—	0.5^i^	4	1.3
5^j^	LA (20)	CL (20)	10	10	99	99	50 : 50	44 : 56	5.1	11.8	1.6	1	2	1.3
6^g^	LA + CL (20) (80)		10		73	98	20 : 80	23 : 77	11.4	—	—	1	5	1.9
7	CL (100)	DL (100)	10	22.3 h	99	91	52 : 48	54 : 46	26.8	25.1	1.7	Block copolymers		
8	DL (100)	CL (100)	19.6 h	10	91	97	48 : 52	57 : 43	26.5	22.2	1.1	Block copolymers		
9	LA (100)	DL (100)	15	24 h	>90	0	100 : 0	100 : 0	—	—k	—	PLA homopolymer		
10	DL (100)	LA (100)	18.8 h	60	92	94	49 : 51	45 : 55	26.4	27.4	1.1	Block copolymers		

a *M* ^theo^_n_ = (*M*_*M*_1__ × ([*M*_1_]_0_/[BnOH]) × *C*_*M*_1__%) + (*M*_*M*_2__ × ([*M*_2_]_0_/[BnOH]) × *C*_*M*_2__%), where *M*_*M*_1__ is the mass of monomer 1, and *C*_*M*_1__% is the %conversion of monomer 1.

b *M* ^exp^_n_ and *Đ* determined by SEC analysis in THF using polystyrene standards, through RI detection.

c Average lengths (*l*) of glycolidyl or lactidyl blocks in copolymer chains and randomness factor (*R*) calculated using eqn (S1)–(S22) (see SI for details).

d The CL : GA composition was kept ≤50% GA in all cases, so that the copolymers were soluble despite the limited solubility of PGA in common organic solvents.

e *M*_n_,NMR reported instead of *M*_n_,SEC.

f [GA] = 0.33 M (a lower concentration was used due to poor solubility), 85 °C.

g Simultaneous addition of monomers (see SI Table S2 for additional details for GA and CL).

h *M*_n_ and *Đ* data not provided due to limited polymer solubility.

i 0.5 Glycolidyl units represents one glycolyl unit (O–CH_2_–C(O)–).

j Data from literature ref. 17.

k SEC data was not collected due to the formation of PLA homopolymer. Bis-Zn 1 has been reported to polymerise LA with relatively good control over the molecular weight and dispersity in the absence of DL.^17,34^

Reversing the sequence of monomer addition, by adding CL to a propagating PGA* chain, instead generated random copolymers (*R* = 1.0, entry 3 *cf*. *R* = 0.08, entry 1). The average sequence lengths are significantly shorter, with only 2–3 sequential GA or CL units along the chain, respectively (Table 1, entry 3), and DSC analysis confirmed a random copolymer architecture (Fig. S24). This mirrors observations with PLA-*ran*-PCL copolymers generated through addition of CL to a PLA* chain (entry 3 *vs*. entry 5). Simultaneous addition of CL and GA also gave random copolymers (entry 4, see SI Fig. S25–S28), with CL insertion promoting transesterification during polymerisation. Intriguingly, this indicates that there is something specific about CL that triggers transesterification for both PLA* and PGA* chains. It is not solely the steric bulk of the secondary alkoxide in a living PCL–PLA* chain that prevents transesterification, as the PCL–PGA* copolymers with a primary alkoxide chain-end also retained a well-defined blocky structure. This indicates that other monomer features are also important, such as the ester functional group density and chelation potential (*vide infra*).

Initial comparisons suggested that the PLA–PCL copolymers are transesterified more than the PGA–PCL analogues, both with simultaneous and sequential monomer addition, when CL was added as the second monomer (*e.g.* entry 4, *R* = 1.3 *vs*. entry 6, *R* = 1.9). As demonstrated in the detailed microstructural studies by Kasperczyk, Bero, and coworkers, *R* values >1 arise from selective transesterification events involving the cleavage of internal ester bonds within the lactidyl or glycolidyl units, leading to specific sequence distributions such as “CL–L–CL” and “L–CL–L” triads.^40^ Here, the *R* values >1 are thus due to a high concentration of sequences where the lactide or glycolide diester is cleaved into lactic acid units “L” or glycolic acid units “G”. To further interrogate this observation, the synthesis of triblock PCL–PGA–PCL and PCL–PLA–PCL copolymers was investigated (see SI sections S2.2.2 and S2.2.4 for further details). In all cases, increasing the equivalents of CL gave a pronounced increase in the randomness of the polymer chain (Tables 1, S2 and S3). Intriguingly, higher *R* values were obtained for the PLA–PCL copolymers than the PGA–PCL copolymers, with transesterification occurring in both the PCL and PLA domains. In the PCL–PGA copolymers, transesterification preferentially occurred in the PGA block (see SI, Table S3 for details). This is attributed to the lack of methyl substituents in PGA compared to PLA; methyl groups provide both steric and electronic stabilisation of the ester group.

Overall, this demonstrates that CL-triggered transesterification is a powerful method of modifying PCL–PLA and PCL–PGA copolymer structures, with the CL loading and/or conversion controlling the extent of transesterification. The transesterification trends of PCL–PGA and PCL–PLA copolymers are broadly similar. In both cases, BCPs can be produced *via* a “PCL first” route but the addition of CL to a living PGA* or PLA* chain causes transesterification. GA, with no methyl substituent, does not transesterify PCL* chains. LA ROP is slower than GA ROP, with the ring-substituents in LA and PLA hindering monomer coordination to the metal as well as subsequent nucleophilic attack, thus hampering propagation. This is reflected in the sequential copolymerisation of LA with ε-decalactone (DL), the *n*-butyl-substituted analogue of CL. Only PLA chains were observed, with no DL ROP (entry 9), suggesting that PLA* is unable to perform nucleophilic attack upon DL. This is attributed to the sterically bulky ring-substituents on both the DL monomer and living PLA* chain. Notably, bis-Zn 1 gave good activity and control in DL homopolymerisation (refer to SI section S2.1.3 for details), albeit much slower than that of CL due to the bulky electron-donating *n*-butyl substituent disfavouring monomer coordination and nucleophilic attack for both steric and electronic reasons. Switching the order of addition to a “poly(ε-decalactone) (PDL) first” route successfully generated PDL–PLA copolymers (entry 10, SI section S2.2.6), indicating that the key challenge with the PLA first route is the inability of a living PLA* chain to initiate DL polymerisation under the conditions tested.

The sequential polymerisation of DL and CL, through either a “PCL first” route or a “PDL first” route, generated BCPs structure in both cases although the control was improved with a “DL first” route (Table 1, entries 7 and 8, see SI section S2.2.5 for additional details). The BCP structures were evidenced by the presence of just two ester resonances in the ^13^C NMR spectrum at 173.7 and 173.4 ppm (Fig. S43), corresponding to PCL and PDL segments, respectively – random PCL–PDL copolymers have additional ^13^C NMR ester resonances present.^41,42^ This provides an interesting contrast from the PGA–PCL and PLA–PCL studies, where adding CL to a PGA* or PLA* chain always triggered transesterification. Notably, the ester units are further apart from each other in the PDL chain than in PGA and PLA. Such ester group density in a polymer chain not only impacts the chain flexibility and the potential for polymer chelation to a metal centre, but also the electrophilicity of ester groups due to the proximity of an inductively electron-withdrawing heteroatom. These observations raised the question of whether a greater proximity between ester units disfavoured transesterification, prompting investigation into BCP formation using macrolactones and cyclic esters featuring an additional heteroatom within the ring.

### Relative proximity of ester units

As CL induced transesterification of PGA and PLA but not PDL, this raised the question of whether transesterification was promoted by facile Lewis base coordination and the high electrophilicity of the carbonyl units within ester-dense PGA and PLA. Simultaneously, the flexibility of the propagating PCL chain, with a lower ester-group density, was hypothesised to facilitate nucleophilic attack and enhance transesterification. To probe the impact of the ester group spacing upon transesterification, pentadecalactone (pDL) was therefore investigated along with GA and CL (Scheme 2). Macrolactones such as pDL have a relatively low ring-strain due to the large ring-size, and are thus typically slow to polymerise.^43–45^ Accordingly, the efficacy of bis-Zn 1 for the homopolymerisation of pDL ROP was established and found to convert 50 equivalents in 72 hours, giving poly(pentadecalactone) (PpDL) with a molar mass distribution (*Đ*) of 2.1. High dispersities and the formation of cyclic oligomers are relatively common for polymers synthesised through the ROP of macrolactones, which has been attributed to similar propagation and transesterification rates due to the relatively low ring-strain of macrolactones, whereas propagation is generally faster than transesterification in strained ring lactones. The subsequent addition of CL gave PpDL-*b*-PCL BCPs (Table 2, entries 1 and 2). This was evidenced by the presence of two carbonyl resonances in each ^13^C NMR spectrum (*δ* = 174.0 ppm for PpDL and *δ* = 173.5 ppm for PCL, Fig. S63), with distinct melting transitions corresponding to the crystalline domains of PpDL and PCL in the DSC curve (Fig. S65). The same trend was observed for pDL and DL copolymerisations through a sequential “pDL first” route, which generated PpDL-*block*-PDL copolymers (see SI section S2.2.8 for details).

**Scheme 2:**
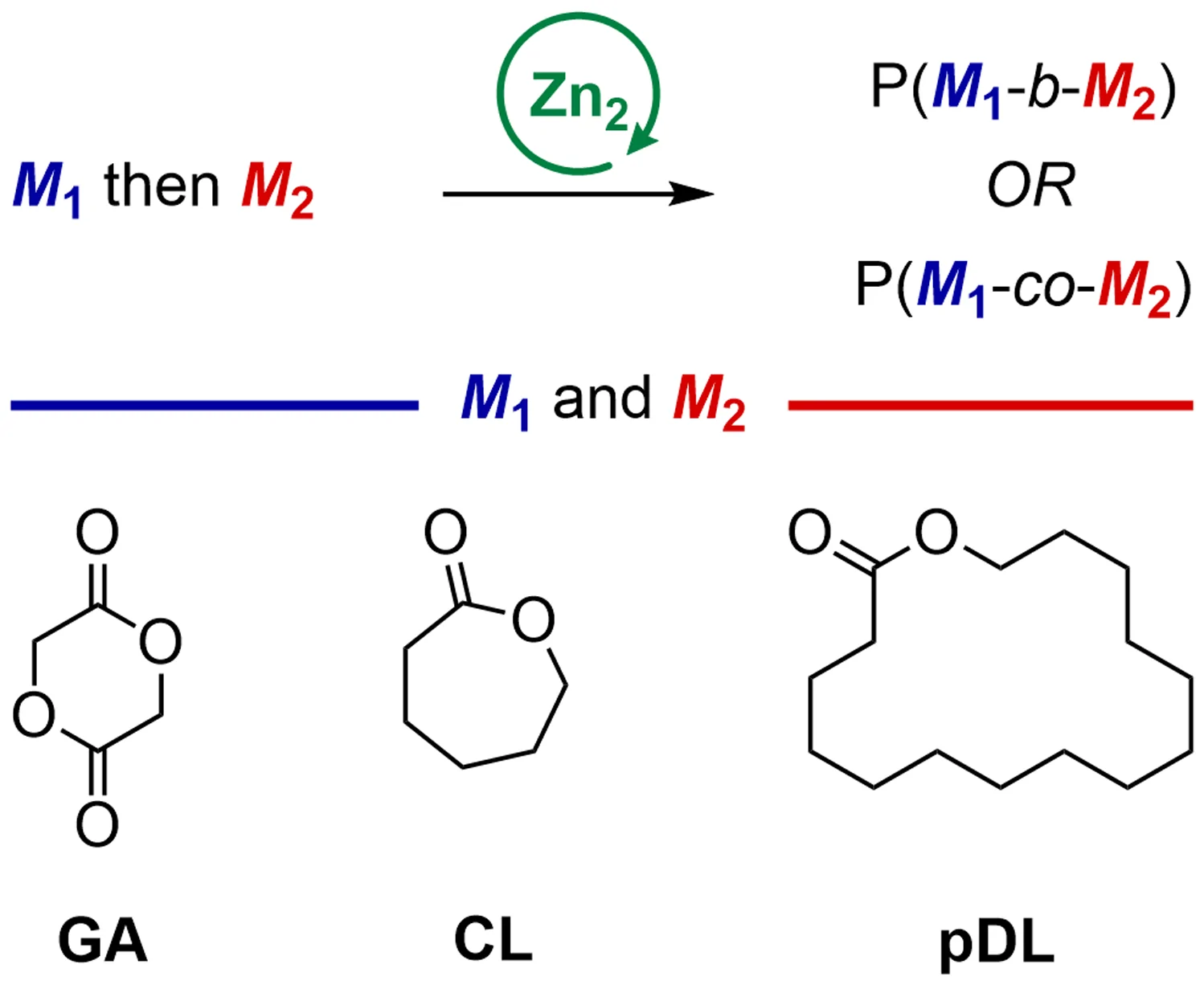
Investigating the influence of ester proximity (glycolide (GA), ε-caprolactone (CL) and pentadecalactone (pDL)) in copolymerisations with sequential monomer addition.

**Table 2:** Sequential^a^ and simultaneous^b^ copolymerisation of pentadecalactone (pDL) with ε-caprolactone (CL)

Entry	*M*_1_ (eq.)	*M*_2_ (eq.)	Molar comp. (*M*_1_ : *M*_2_)	*M*_n_ (kDa)		*Đ* d	Copolymer structure
				Theo.^c^	Exp.^d^		
1^a^	pDL (40)	CL (40)	44 : 56	17.2	20.0	2.0	Block
2^a^	pDL (40)	CL (80)	20 : 80	20.6	26.0	3.3	Block
3^a^	CL (40)	pDL (40)	63 : 37	10.4	42.0	1.7	Random
4^b^	CL (40) + pDL (40)		55 : 45	15.9	13.3	1.9	Random

a Sequential monomer addition.

b Simultaneous monomer addition.

c Calculated from conversion *M*^theo^_n_ conversion *M*_1_ × *M*_*M*_1__ × ([*M*_1_]_0_/[LZn_2_OBn]) + conversion *M*_2_ × *M*_*M*_2__ × ([*M*_2_]_0_[LZn_2_OBn]).

d Obtained by SEC in THF using PS standards.

Reversing the order of monomer addition by adding pDL to a propagating PCL* chain (Table 2, entry 3) induced transesterification, as identified by the appearance of PpDL–PCL junction peaks in the ^13^C NMR spectrum (Fig. S67). Due to the low ring-strain and slow propagation of pDL, transesterification competes with pDL propagation when pDL is added to PCL* as the second monomer. Conversely, the relatively rapid propagation rate of CL as the second monomer to a PDL* chain favours propagation to deliver BCPs. Simultaneous copolymerisation of pDL with CL also produced random copolymers (Table 2, entry 4), which is attributed to the random insertion of both CL and pDL units from a mixed monomer feed as well as possible transesterification (Fig. S71–S73).

Adding pDL to a living PDL* chain gave no propagation (Table S5), which is attributed to the steric bulk of the *n*-butyl substituent disfavouring nucleophilic attack and ring-opening, while simultaneous copolymerisation of DL and pDL gave somewhat blocky structures (section S2.2.8, Fig. S80 and S81). Transesterification is typically observed when the second monomer is slower to polymerise, as it can outcompete the slower propagation rate. For example, CL induces transesterification of PGA* (*vide supra*), whilst pDL induces transesterification of PCL*. Transesterification is also disfavoured when the ester groups of the second monomer are in close proximity (*e.g.* GA does not transesterify PCL*). Yet this is not always the case, as DL has a slow propagation rate and identical ester spacing to CL, yet does not transesterify PCL* chains (*vide supra* and SI section S2.2.5 for additional details), likely due to the relatively large size of the *n*-butyl ring substituent. Overall, these observations indicate that ester group proximity, along with monomer ring-strain and propagation rate, are all important factors but do not give the full story. This prompted investigation into the effect of additional heteroatoms in the lactone ring that could form chelate complexes with the metal centre. Since transesterification is catalysed by coordination of an ester carbonyl (from the polymer chain) to the Lewis acidic metal centre, the formation of a chelate complex with the active polymer chain end was hypothesised to disfavour polyester coordination and thus limit transesterification. For example, GA and LA could all form 5-membered chelate complexes at the active chain end (Zn–O–CH_*x*_(Me)_2−*x*_–C=O–), whereas CL, DL and pDL could not. Accordingly, a range of heteroatom containing lactones were studied.

### Presence of heteroatoms within lactone monomers

To probe the influence of Lewis basic heteroatoms in lactone monomers on the extent of transesterification, the copolymerisation of CL or LA with six-membered rings, namely δ-valerolactone (VL), trimethylene carbonate (TMC) and *para*-dioxanone (*p*DO) was investigated (Scheme 3). TMC and *p*DO are structural isomers, and structurally similar to VL, which was previously shown to transesterify living PLA* and PCL* chains using bis-Zn 1 after long reaction times.^17^ Homopolymerisation studies showed that bis-Zn 1 is an effective catalyst for both TMC and VL, efficiently polymerising essentially 100 equivalents of monomer within a matter of minutes (see SI sections S2.1.5 and S2.1.6). The homopolymerisation of *p*DO was not studied in detail due to the poor solubility of poly(*para*-dioxanone) (P*p*DO) in common organic solvents. The extension of propagating PCL* or PLA* chains with TMC, GA or *p*DO led to blocky copolymers in all cases, as evidenced by the low *R* values (Table 3, see SI for details). This lack of transesterification may be partly due to the rapid polymerisation of TMC, GA and *p*DO, with the proximal electron-withdrawing heteroatoms increasing the electrophilicity of the carbonyl group of the monomer, thus enhancing nucleophilic attack at the monomer and favouring propagation over transesterification. The trends with VL, which does not contain an additional heteroatom within the ring, are different. Addition of VL to PCL* gave blocky structures initially (Table 3, entry 7), while extending the reaction time promoted transesterification.^17^ The addition of VL to a PLA* chain gave transesterification at all times measured (Fig. S110). This shows that the presence of additional heteroatoms in the second monomer added plays a significant role in reducing transesterification reactions, allowing the successful generation of BCPs. As electron-withdrawing heteroatoms would increase the electrophilicity of the polyester carbonyl units, potentially favouring transesterification, the lack of transesterification is thus attributed to the heteroatoms forming chelation complexes with the Lewis acidic zinc centres, disfavouring coordination of another polyester chain to the metal and mitigating transesterification.

**Scheme 3:**
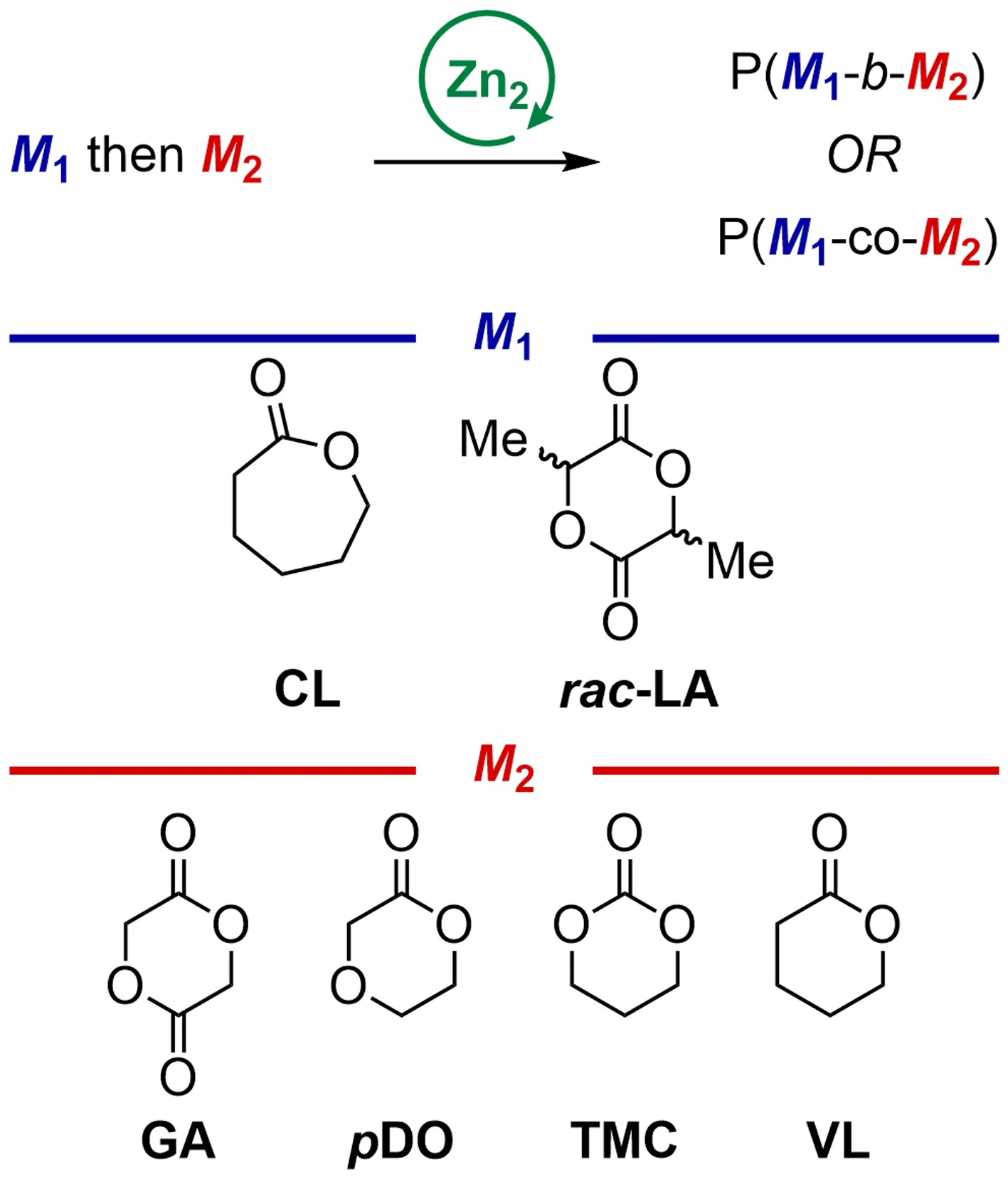
Investigating the role of heteroatoms (glycolide (GA), *para*-dioxanone (*p*DO), trimethylene carbonate (TMC) and δ-valerolactone (VL)) in copolymerisations with sequential monomer addition.

**Table 3:** Sequential polymerisation of *rac*-lactide (*rac*-LA) or ε-caprolactone (CL) followed by trimethylene carbonate (TMC), *para*-dioxanone (*p*DO), δ-valerolactone (VL) or glycolide (GA)

Entry	*M*_1_ a	*M*_2_ a	Time (min)		*M*_2_ Conv	Molar Comp. (*M*_1_ : *M*_2_)	*R* b
			*M*_1_	*M*_2_			
1	*rac*-LA	TMC	30	0.08	99	45 : 55	0.12
2	*rac*-LA	*p*DO	30	5	40	51 : 49	0.08
3	*rac*-LA	VL	30	240^c^	95	52 : 48	—d
4^e^	*rac*-LA	GA	30	20	—f	85 : 15	—g
5	CL	TMC	30	0.08	99	54 : 46	—h
6	CL	*p*DO	30	1	72	52 : 48	0.09
7	CL	VL	30	30	76	62 : 38	—i
8	CL	GA	10	2	99	56 : 44	0.08

a Unless otherwise specified, 20 equivalents of *M*_1_ and *M*_2_ were used in all cases at 70 °C in toluene solvent at 1 M. Full conversion of *M*_1_ (>99%) was observed in each instance.

b Where possible, *R* values were calculated based on formulae reported in literature (see SI for calculations).^46–48^

c Extended reaction times were required to reach high VL conversion, see Fig. S110 for additional time points.

d Random copolymer structure determined by ^1^H and ^13^C NMR (Fig. S104–S106).

e *rac*-LA and GA polymerised at 0.5 M in toluene, poor solubility of a 20–20 PLA–PGA block copolymer required a longer PLA chain to be formed (80 eq.).

f Poor solubility of GA in common organic solvents prevented the determination of conversion.

g While an *R* value was not calculated for the PLA-*b*-PGA synthesised, comparison to the work of Kasperczyk suggests that negligible transesterification occurs.^49^

h Calculation of the average block lengths indicates *l*_CL_ = 18 and *l*_TMC_ = 14 (see SI section S2.3.2 for details).^50^

i Block structure determined by ^1^H and ^13^C NMR (Fig. S101–S103).

Overall, these studies provide important insight, as the possibility for chelation complexes formed from the last-inserted monomer can prevent transesterification pathways from occurring. This conclusion is further supported by the PCL–PLA, PCL–PGA and PLA–PGA studies, as all of these copolymers contain a second ester unit that can form a chelation complex. No transesterification was observed when either LA or GA was added to a living PCL* chain, or when GA was added to a living PLA* chain, providing further evidence that the formation of a chelate complex helps to impede transesterification. To probe this further, we investigated the potential competition between monomer and polymer coordination, by using the FT-IR shifts to give a quantitative indication of the Lewis basicity.

### Indexing monomer and polymer Lewis basicity *via* NMR spectroscopy and FT-IR spectroscopy

Lewis basicity influences multiple aspects of metal-catalysed cyclic ester ROP, including monomer coordination to enhance propagation, polymer coordination to enhance transesterification,^16^ and chelation to change the nature of the metal–O_polymer_ bond. The relative strengths of the monomer and polymer Lewis basicity were thus investigated using NMR spectroscopic methods developed by Franz and co-workers.^51^ Pentafluorobenzoic acid (PFBA) was used as an ^19^F NMR probe to index lactone monomers and polyesters, based on their hydrogen-bond acceptor (HBA) character. HBA character has been used as a proxy parameter to study Lewis basicity through a variety of spectroscopic and calorimetric techniques.^52–55^ Here, we use HBA character, measured by ^19^F NMR probe studies, as a method of indexing cyclic esters as a family of Lewis bases, in line with the principles outlined by Laurence and Gal.^53^ So far, this methodology has been applied only to small molecules, and not macromolecules. The use of this approach to understand copolymer structures is also unprecedented.

To determine the number of equivalents of the hydrogen bond acceptor required to saturate the PFBA probe, 1–20 equivalents of monomer or polymer repeat unit was added to a PFBA solution, at constant temperature and PFBA concentration (see SI for details). Saturation was achieved upon the observation of a plateau in the change of the ^19^F chemical shift, based on the diagnostic *para*-fluorine atom of PFBA (*i.e.* Δ*δ*_p_^19^F upon increasing equivalents was <0.1 ppm). The monomers and polymers tested within this work are oxygen-based Lewis bases and thus show relatively weak HBA character, with approximately 10 equivalents of monomer required to saturate the probe (Fig. S113).^51^ To be confident of probe saturation and to maintain consistency across all samples, the change in chemical shift (Δ*δ*_p_^19^F) is reported for 20 equivalents of HBA (Table 4), as some monomers and polymers (l-LA, PVL and PTMC) showed Δ*δ*_p_^19^F > 0.1 ppm upon increasing the HBA equivalents from 10 to 20. The greater the value of Δ_p_*δ*^19^F, the greater the *effective* Lewis basicity, which is defined by the induced change of a Lewis acidic probe upon binding to a Lewis base.^56,57^ In addition, ^13^C NMR and FT-IR spectroscopy data for the carbonyl resonances provides a measure of the *intrinsic* Lewis basicity, *i.e.* the uncoordinated, free Lewis base (Table 4). The HBA character was typically lower for polymers than the monomers, suggesting that monomer coordination would typically be favoured over polymer coordination (see SI Tables S7 and S8, Fig. S114 for additional details).

**Table 4:** Δ*δ*_p_^19^F data for lactones and polyesters binding to PFBA, along with *δ*_C=O_^13^C NMR data and C=O FT-IR stretching frequencies for lactones (see Tables S7 and S8 for additional details, including polymer data)^a^

Entry	*M*	Δ*δ*_p_^19^F NMR^b^ (ppm)	^13^C NMR peak for C=O in CDCl_3_^c^ (ppm)	FT-IR C=O stretching frequency^d^ (cm^−1^)	Assigned relative Lewis basicity
1	GA	0.3	163.6	1760	Very low
2	l-LA	0.6	167.5	1753	Low
	*rac*-LA		167.5	1752	
3	*p*DO	0.9	166.6	1740	Medium
4	VL	1.3	171.4	1734	Medium–high
5	TMC	1.6	148.6^e^	1747	High
6	CL	1.7	175.7	1729	High
7	DL	2.0	175.9	1730	High

a 20 molar eq. of hydrogen bond acceptor (HBA) (monomer or repeat unit of polymer) in CH_2_Cl_2_ (27% CD_2_Cl_2_, PFBA probe concentration = 5.45 µM) at 300 ± 1 K analysed by ^19^F NMR spectroscopy (558 MHz).

b Δ*δ*_p_^19^F reported for *para*-fluorine of PFBA in the presence of HBA compared to PFBA external standard. See SI for additional information.

c Spectra shown in Fig. S115.

d Values taken from literature ref. 36 and 58–63.

e The ^13^C NMR shift for TMC is significantly lower as the carbonyl ^13^C is part of a carbonate unit, rather than an ester unit.

While all the monomers investigated showed comparable HBA character, with Δ*δ*_p_^19^F values ranging from 0.3 to 2.0 ppm, several trends emerged (Table 4). Firstly, a direct correlation between a lower HBA character (inferring lower effective Lewis basicity) and smaller lactone ring size was observed, which is in agreement with literature reports of HBA character of lactones.^54,64^ The seven-membered lactones, DL and CL, displayed the highest Δ*δ*_p_^19^F values, indicating the highest HBA character. Secondly, the presence of electron-donating alkyl substituents generally enhanced the Lewis basicity leading to greater HBA character. For example, l-LA gave a higher Δ*δ*_p_^19^F value than GA (0.6 *vs*. 0.3 ppm), and DL gave a greater Δ*δ*_p_^19^F value than CL (2.0 *vs*. 1.7 ppm). Thirdly, within the six-membered ring series, carbonates had higher HBA character than lactones (*e.g.* Δ*δ*_p_^19^F = 1.6 for TMC *vs*. 1.3 ppm for VL). This observation is attributed to oxygen lone pair donation from both of the oxygens adjacent to the carbonyl bond in TMC, increasing the electron density and thus increasing the HBA character. Finally, the presence of remote heteroatoms reduced the HBA character, with *p*DO, l-LA and GA displaying the lowest HBA ability (Δ*δ*_p_^19^F = 0.9 ppm for *p*DO, 0.6 ppm for l-LA and 0.3 ppm for GA). The remote oxygen atoms can inductively withdraw electron density from the carbonyl group, reducing the HBA potential. Importantly, the HBA trends determined herein, regarding alkyl-substitution and ring-size, agree with the work of Berthelot, Yáñez and co-workers,^54,64^ as well as computational studies that demonstrate the increased Lewis basicity of CL over LA.^23^

The coordinating power (*i.e.* the relative intrinsic Lewis basicity) of an ester group can be inferred from the carbonyl stretching frequency in the FT-IR spectrum. The lower the wavenumber, the weaker the carbonyl bond (due to increased electron delocalisation), and thus the higher the intrinsic Lewis basicity. The ^13^C_C=O_ NMR shift also provides a useful handle; the ^13^C_C=O_ NMR resonance of esters is lower than that of ketones due to the presence of the electron-donating O atom. Thus, the greater the electron donation from O (due to orbital overlap in the monomer ring), the lower the ^13^C_C=O_ NMR resonance and the higher the intrinsic Lewis basicity. Gathering FT-IR carbonyl stretching frequency, *δ*^13^C_C=O_ NMR shifts and Δ*δ*_p_^19^F values gives some indication of the relative strengths of monomer and polymer coordination power (Tables 4, S7 and S8). The FT-IR and *δ*^13^C_C=O_ NMR values refer solely to the intrinsic Lewis basicity, which may be altered through coordination to a Lewis acid. However, the FT-IR and ^13^C NMR carbonyl resonances give fairly good agreement with the HBA studies, indicating some correlation between effective and intrinsic Lewis basicity. The use of FT-IR stretching frequencies is particularly beneficial for polymers that are insoluble in common NMR solvents, such as P*p*DO and PGA (Table S7). Taken together, the HBA values, FT-IR and ^13^C NMR shifts give important insight into the potential Lewis basicity of monomers and polymers, providing tools to help to quantify the intensity of the ester group-catalyst interactions.

Importantly, the data shows that monomers with a higher Lewis basicity have a tendency to promote transesterification (Fig. 2). For example, LA monomer has a low Lewis basicity and typically forms block structures *i.e.* does not transesterify PCL* or PDL* chains (Fig. 2A). In contrast, CL monomer shows high Lewis basicity, and favours random copolymer structures (Fig. 2B), *i.e.* CL transesterifies PLA* and PGA* chains. In the presence of stronger (highly electron-donating) Lewis bases such as CL, the metal–O_polymer_ alkoxide chain is expected to become weaker, dissociating the polymer chain, increasing the nucleophilicity of the alkoxide group and favouring transesterification (Fig. 3B).^18^ Indeed, literature reports from several different research groups have shown that Lewis base coordination to Zn increases the Zn–O_phenoxide_ bond lengths in salophen complexes (Table S9). Reviewing the literature also revealed that there is a correlation between the Zn–O_phenoxide_ bond lengths and the HBA character of the Lewis donor, with the latter determined by Franz and co-workers.^51^ Previous work has established the use of HBA character to draw comparisons within families of Lewis bases (*e.g.* pyridines and imidazoles). Here, this literature search indicates that within each family of Lewis bases, there is a correlation between HBA character and Zn–O_phenoxide_ bond length, and thus Lewis basicity (Table S9).^53,65–71^ In contrast to CL, when LA is added as the second monomer the lower monomer Lewis basicity is less likely to cause dissociation of the metal–O_polymer_ bond, disfavouring transesterification (Fig. 3A). Furthermore, the PLA* chain end, which is generated *via* propagation of the added LA monomer, is expected to be more strongly coordinated to the metal centre due to chelation of the carbonyl in close proximity to the metal centre (Fig. 3A).^19–23,33,72^ The facile formation of BCPs from PLA* with *p*DO or TMC, and PCL* with *p*DO or TMC, indicates that the Lewis basic heteroatoms in these monomers also enable stabilising chelation interactions with the metal centre. DL is a notable exception, as despite the high Lewis basicity, DL did not transesterify PCL*, indicating that the steric bulk of the substituents may play a more significant role than the Lewis basicity. While the ^19^F NMR studies indicate that the polymers are generally weaker Lewis bases than the monomers, polymer coordination to the metal centre may also contribute to chain dissociation and transesterification.

**Fig. 2:**
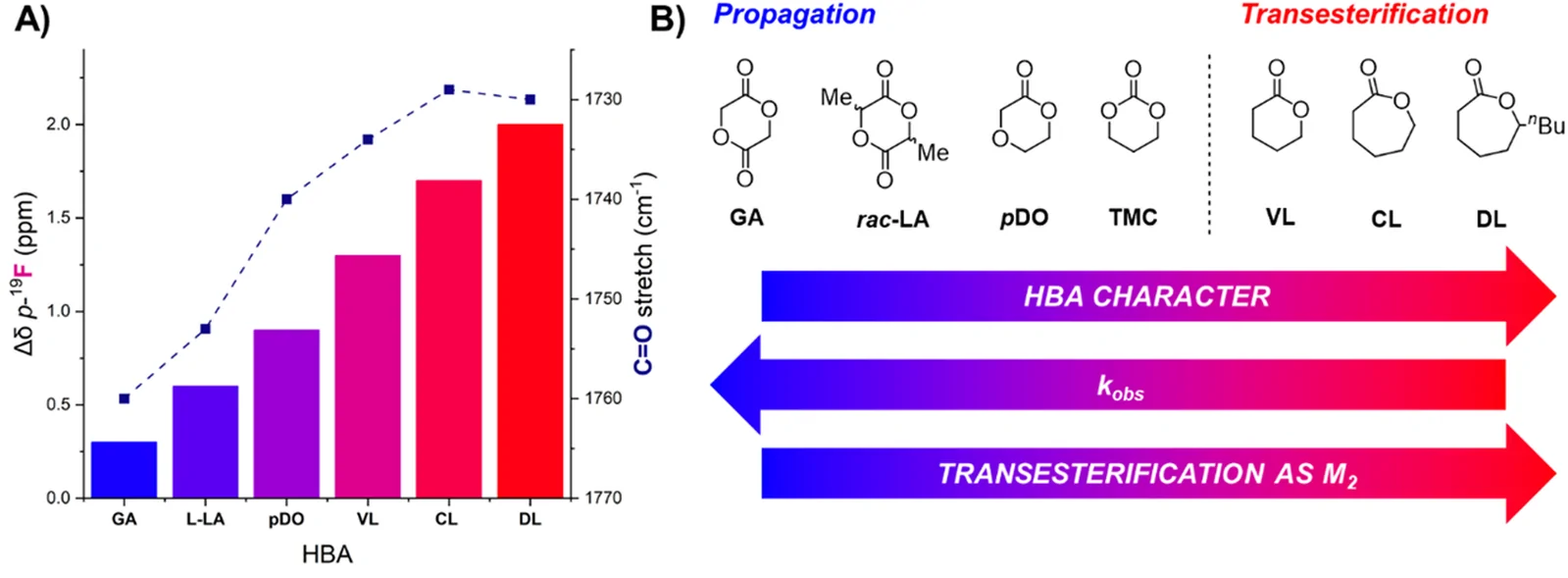
(A) Plot of Δ*δ*_p_^19^F (bar chart) and FT-IR C=O stretching frequency (scatter plot) for cyclic ester monomers investigated within this study. The values of Δ*δ*_p_^19^F and *υ*C=O generally increase with increasing Lewis basicity; (B) graphical overview of how HBA character and *k*_obs_ using catalyst 1 influence propagation *versus* transesterification (see SI, section S2.1.7 for *k*_obs_ data).

**Fig. 3:**
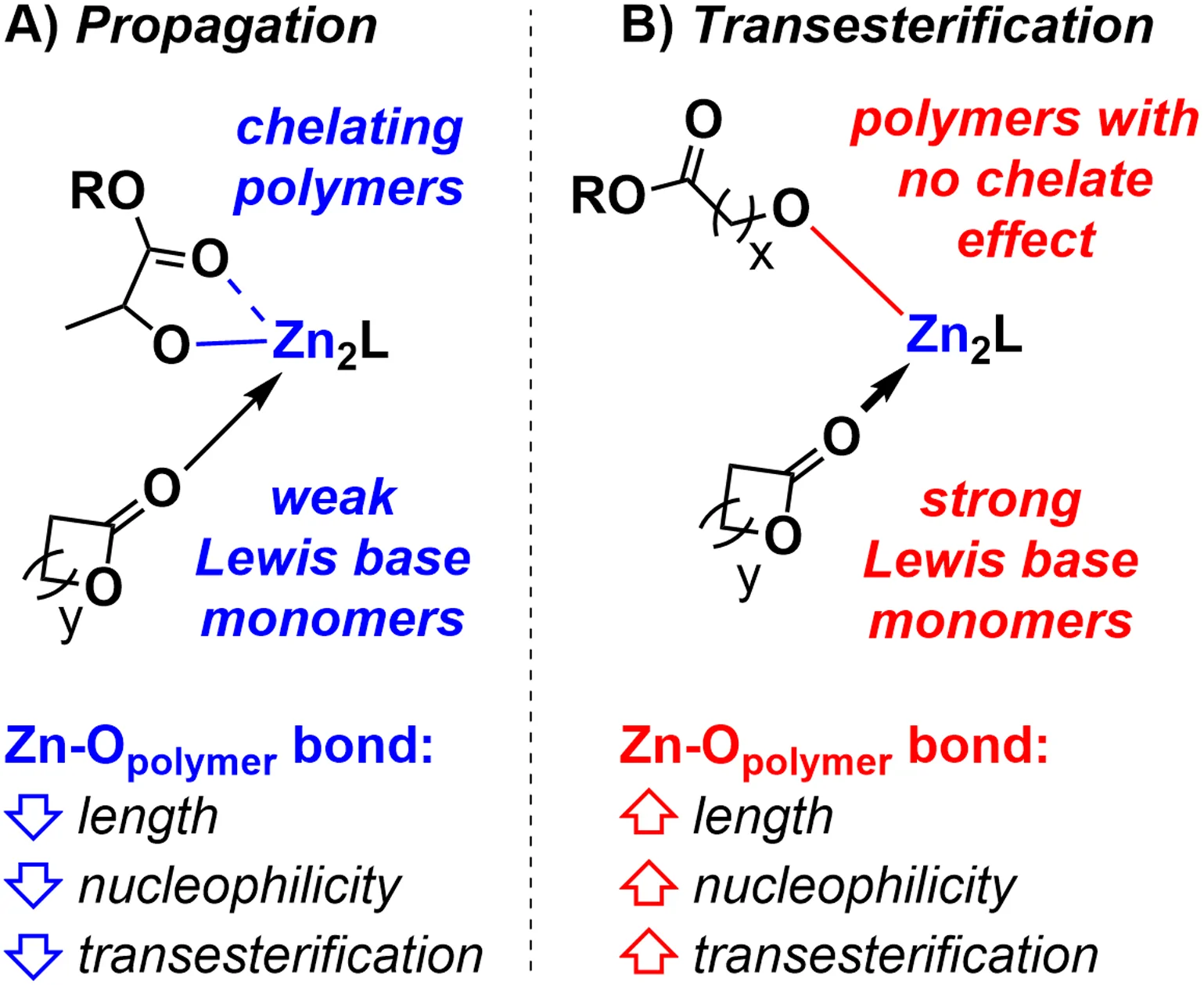
(A) Weak Lewis bases and chelation hinders the dissociation of propagating polymer chains, favouring propagation and disfavouring transesterification; (B) strong Lewis bases promote dissociation of propagating polymer chains, favouring transesterification and disfavouring propagation. OR = initiator or propagating polymer chain. PGA, PLA and P*p*DO are proposed to form chelate structures.

To experimentally test our hypothesis that highly Lewis basic monomers promote transesterification, the copolymerisation of VL with CL or LA was investigated, as our studies indicated that VL has a relatively high intrinsic and extrinsic Lewis basicity. The sequential copolymerisation of VL followed by LA generated BCPs, as determined by ^1^H and ^13^C NMR analysis (Fig. S104–S106). In contrast, we have shown that the sequential copolymerisation of LA followed by VL gives PLA-*ran*-PVL copolymers under identical reaction conditions (*vide supra*).^17^ This shows that VL transesterifies PLA*, but LA does not transesterify PVL*, in line with the monomer Lewis basicity, chelation potential and propagation rate of the second monomer (Fig. 2B). Addition of CL monomer to a PVL* chain resulted in transesterification (Fig. S98–S100), whereas the inverse order initially gives a BCP (Fig. S101–103) which then undergoes transesterification after prolonged reaction times.^17^ This provides support that VL and CL both have strong enough Lewis basic character to promote transesterification when added as *M*_2_, and that a greater extent of transesterification occurs with CL than VL.

To investigate the universality of the relationship between Lewis basicity, propagation rate and transesterification, we surveyed the literature to see whether previous publications matched our observations of which monomers can tip the balance between transesterification and propagation. This search targeted diblock copolymers prepared *via* sequential monomer addition, using metal-based catalysts (excluding bis-Zn 1). It is important to note that the reaction temperature and metal selected can also influence the extent of transesterification (*e.g.* Bu_2_Sn(OR)_2_ > Bu_3_SnOR > Ti(OR)_4_ > Zn(OR)_2_ > Al(OR)_3_).^73–75^ Furthermore, not all monomer combinations studied here have been reported, which makes it challenging to draw universal comparisons. While there are a few exceptions,^28,76–78^ the metal-based catalyst literature generally shows that:

• GA does not transesterify PLA*,^79,80^ PCL*,^80–82^ PTMC*^83^ or P*p*DO*^11^

• LA does not transesterify PTMC*,^21,84–92^ PVL*,^93^ PCL*^94–102^ or PDL*^103–106^

• P*p*DO does not transesterify P(TMC-*co*-CL)*^107^ or PCL*^11,108^ but may transesterify PGA*^109^ (note that the latter has partly been attributed to P*p*DO depolymerisation to *p*DO at high temperatures)

• TMC transesterifies PGA*,^83^ does not efficiently form copolymers from PLA*,^21,87,110^ and does not transesterify PCL*.^111–113^

• VL transesterifies PLA*^27,114^ but not PCL*^115–118^ or PTMC*^110^

• CL transesterifies PLA*^16,119–123^ but not PVL*,^115,116^ PTMC*^111^ or PDL*^124^

• DL does not transesterify PCL* (attributed to the steric bulk of the *n*-butyl substituent)^106,124^

While not a completely clear-cut trend, this literature survey shows a relationship between monomer Lewis basicity and increased potential for transesterification, in polymerisations with sequential monomer addition. Firstly, GA and LA are weak Lewis bases with fast propagation rates, that can also form metal chelation complexes at the active chain end. This prevents transesterification when GA or LA is added as the second monomer. Secondly, monomers with higher Lewis basicity, namely *p*DO, TMC, VL and CL, favour transesterification when added as the second monomer – but not in all cases. Transesterification is particularly prevalent when the first polymer block contains a high density of ester linkages, such as PGA and PLA. Finally, in spite of its high Lewis basicity, DL does not transesterify PCL. This is attributed to the bulky *n*-butyl substituent, and DL may also promote the transesterification of ester-dense polymers such as PGA* and PLA*. Overall, this data shows that transesterification processes can be highly complex, but certain monomer features promote propagation (chelating heteroatoms and ring-substituents), while others favour transesterification (high Lewis basicity). This aligns with previous studies, which showed that the formation of relatively stable 5-membered cyclic chelation complexes from lactidyl or glycolidyl units disfavours the propagation of cyclic esters that do not form such stable chelation complexes, such as CL.^19^ While this effect appears to be ligand and metal dependent, similar observations have been made for catalysts based on aluminium,^22,125,126^ magnesium,^22,127,128^ and zinc.^21,22,129^

## Conclusions

The systematic synthesis and characterisation of block and random polyester architectures, using 13 different monomer pairs, shows several key trends. This was made possible using bis-Zn 1 as a highly versatile catalyst, which is capable of the homo- and copolymerisation of CL, DL, GA, LA, pDL, *p*DO, TMC and VL in various combinations. With sequential monomer additions to bis-Zn 1, propagation to form diblock copolymers is generally favoured over transesterification to form random copolymers when the homopolymerisation rate is *M*_2_ > *M*_1_. However, this is not true in all instances. For example, initiation of TMC ROP from a living PLA* chain gave block copolymers, whereas VL ROP gave random PLA–PVL copolymers, despite the similar *k*_obs_ values of TMC and VL. This work also highlights that monomer ring-strain is insufficient as a sole predictor of propagation or transesterification. Previous studies indicated that monomers with low ring-strain promote transesterification with bis-Zn 1, yet *p*DO does not transesterify PLA* or PCL*.^130^

Here, ^19^F NMR hydrogen bond acceptor studies combined with ^13^C NMR and FT-IR data allow comparisons to be drawn between different lactone monomers in terms of Lewis basicity. For the first time, these measurable parameters have been systematically correlated to understanding and predicting the competition between transesterification and propagation across a series of cyclic ester monomers, which uncovered the relationship between monomer Lewis basicity and polymer architectures. Relatively strong Lewis basic monomers, such as CL, generally favoured transesterification, not only with bis-Zn 1 but also with many other catalysts reported in literature. Here, we attribute this to monomer-induced dissociation of a propagating polymer chain from the active metal centre. In contrast, less Lewis basic monomers, such as GA and LA, are less likely to promote transesterification, with bis-Zn 1 or many other metal-based catalysts. This is attributed to chelation of the active polymer chain-end to the metal along with the lower monomer basicity disfavouring chain dissociation.

This work reinforces the importance of commonly considered monomer parameters, including propagation rate, ring strain, ring-substituents and the nature of the metal centre. The presence of a chelating chain-end, alkyl substituents, low Lewis basicity and heteroatom units within the lactone added as *M*_2_ all generally favour propagation over transesterification. Taken together, the results using bis-Zn 1 in this work, as well as data from other catalysts reported in the surveyed literature, show that understanding the monomer features that preferentially induce transesterification can provide useful design parameters for targeting new copolymers with desired structures and properties, for example through tailoring the order of monomer addition without the need for extensive catalyst modulation. Whether the monomer Lewis basicity favours intramolecular or intermolecular transesterification remains an open question which will be the subject of follow-on studies. Beyond polymer synthesis, transesterification processes are also key in the chemical recycling of polyesters, to potentially close the loop on polyester materials.

## Author contributions

F. T., J. W. J. H. wrote the manuscript and performed the bulk of the reactions, with editing and revisions by T. J. N., M. L. and J. A. G. Reactions involving DL with LA and CL were performed by K. R. together with T. J. N., and J. A. G. provided overall project supervision.

## Conflicts of interest

There are no conflicts to declare.

## Supplementary Material

PY-OLF-D6PY00814C-s001

## Data Availability

Supplementary information (SI): synthesis details and characterisation data for homopolymers and copolymers including NMR spectroscopy, mass spectrometry, SEC and DSC. See DOI: https://doi.org/10.1039/d6py00814c.
