# Supplementary material for: Uncovering and understanding “transesterification triggers” in aliphatic polyester synthesis with bis-Zn catalysts
Source: Polym Chem. 2026 Nov 11. Online ahead of print. doi: 10.1039/d6py00814c (PMC13754029; doi:10.1039/d6py00814c)
Supplement: Supplementary file 1 [file PY-OLF-D6PY00814C-s001.pdf]

Supplementary Information (SI) for Polymer Chemistry.
This journal is © The Royal Society of Chemistry

Supporting Information

Uncovering and Understanding "Transesterification Triggers" in Aliphatic Polyesters with bis-Zn Catalysts

Federica Tufano,†[1,2] Jack W. J. Hughes,†[1] Thomas J. Neal,[1] Karen Radetzky,[1] Marina Lamberti,[2] Jennifer A. Garden[1]*

†Authors contributed equally

1. School of Chemistry, The University of Edinburgh, Edinburgh EH9 3FJ, U.K.
2. Department of Chemistry and Biology "Adolfo Zambelli", University of Salerno, via Giovanni Paolo II, 132, 84084 Fisciano, Salerno, Italy

Table of Contents

1. Materials and methods 3
   1.1. Materials 3
   1.2. General polymerisation set up 4
   1.3. Calculating *R* values and average monomer sequence lengths for PGA/PCL copolymers 4
2. Polymerisations 6
   2.1. Homopolymerisations 6
      2.1.1. ε-Caprolactone (CL) ROP 6
      2.1.2. *Rac*-Lactide (LA) 7
      2.1.3. ε-Decalactone (DL) ROP 7
      2.1.4. Pentadecalactone (pDL) ROP 9
      2.1.5. δ-Valerolactone (VL) ROP 11
      2.1.6. Trimethylene carbonate (TMC) ROP 13
      2.1.7. Overview of k_{obs} data 16
   2.2. Sequential and simultaneous copolymerisation: ring size and α-substituent 16
      2.2.1. PCL-PGA diblocks 16
      2.2.2. PCL-PGA triblocks 25
      2.2.3. PCL-PLA diblocks 25
      2.2.4. PCL-PLA triblocks 25
      2.2.5. PDL-PCL diblocks 34
      2.2.6. PDL-PLA diblocks 43
      2.2.7. PpDL-CL diblocks 48
      2.2.8. PpDL-DL diblocks 57
   2.3. Sequential copolymerisation, heteroatom containing monomers 62
      2.3.1. PLA-P*p*DO diblocks, LA first 62
      2.3.2. PCL-P*p*DO diblocks, CL first 65
      2.3.3. PLA-PTMC diblocks, LA first 66
      2.3.4. PCL-PTMC diblocks, CL first 69
      2.3.5. PLA-PGA diblocks, LA first 72
   2.4. Sequential copolymerisations with VL 75
      2.4.1. PVL-PCL 75
      2.4.2. PCL-PVL 77
      2.4.3. PVL-PLA 79
      2.4.4. PLA-PVL 81
3. Hydrogen bond acceptor (HBA) character for monomers and polymers 83
   3.1. Methodology 83
   3.2. Exemplar spectra 84
   3.3. $\Delta\delta_p$ vs equivalents for all monomers considered in this study 85
4. References 89

1. Materials and methods

1.1. Materials

All manipulations involving air- or moisture-sensitive compounds were performed in a glovebox and using standard Schlenk techniques under an argon atmosphere. All dry solvent was collected from a solvent purification system (Innovative Technologies), dried over activated 4 Å molecular sieves and stored under argon. Chloroform-*d* ($CDCl_3$) for NMR spectroscopy studies was degassed by three freeze−pump−thaw cycles and stored over activated 4 Å molecular sieves under argon atmosphere. *Rac*-lactide (*rac*-LA) was purified by double recrystallization in toluene followed by sublimation and stored in the glovebox freezer (-35 °C). Glycolide (GA) was recrystallised thrice from ethyl acetate and dried for 24 h at 50 ºC under reduced pressure (0.002 mbar). *Rac*-β-butyrolactone (β-BL) *para*-dioxanone (*p*DO), ε-caprolactone (CL), ε-decalactone (DL) and delta-valerolactone (VL) were stirred over CaH_2 overnight and distilled under reduced pressure and stored at -35 ºC in the glovebox. Trimethylene carbonate (TMC) and pentadecalactone (PDL) were purified by double recrystallisation from tetrahydrofuran and toluene, respectively. Other reagents and solvents were obtained from Sigma-Aldrich, Fisher Scientific, Honeywell and Fluorochem and used without further purification. The **LZn_2Et** complex used in this study was prepared as described in the literature.[1]

All NMR spectra were recorded on either a Bruker AVA500, Bruker AVA600, Bruker PRO500 or Bruker AVA400 spectrometer at 303 K and referenced to the protio impurity of commercial deuterated solvents (e.g. $CDCl_3$ – δ 7.26 ppm for ^1H NMR and 77.16 ppm for ^{13}C NMR) as an internal standard. The Diffusion-Ordered Spectroscopy (DOSY) NMR experiments were performed at 298 K on a Bruker Ascend 2 channel instrument operating at a frequency of 500 MHz for proton resonance under TopSpin (version 3.2, Bruker Biospin, Karlsruhe) and equipped with a z-gradient DCH/5mm tuneable “CryoProbe”™ probe and a GRASP II gradient spectroscopy accessory providing a maximum gradient output of 53.5 G/cm (5.35G/cmA). Diffusion ordered NMR data was acquired using the Bruker pulse program dstebpgp3s with a spectral width of 10330 Hz (centred on 6.175 ppm) and 32768 data points. A relaxation delay of 2 s was employed along with a diffusion time (D) of 100 ms and a longitudinal eddy current delay (LED) of 5 ms. Bipolar gradients pulses (d/2) of 1.5 ms and homospoil gradient pulses of 0.6 ms were used. The gradient strengths of the 3 homospoil pulses were -13.17%, -17.13%, -15.37%. 16 experiments were collected with the bipolar gradient strength, initially at 5% (1st experiment), linearly increased to 95% (16th experiment). All gradient pulses were smooth-square shaped (SMSQ10.100) and after each application a recovery delay of 200 µs used. The experiment was run with 16 scans per increment, employing one stimulated echo with two spoiling gradients. DOSY plots were generated by using the DOSY processing module of TopSpin. Parameters were optimised empirically to find the best quality of data for presentation purposes. Diffusion coefficients were calculated by fitting intensity data to the Stejskal-Tanner expression

SEC analyses of the filtered polymer samples were carried out in either: a) SEC grade THF at a flow rate of 1 mL min^{-1} at 35 °C on a 1260 Infinity II SEC single detection system with mixed bed C PLgel columns (300×7.5 mm); or b) SEC grade $CHCl_3$ at a flow rate of 0.8 mL min^{-1} at 30 °C on a 1260 Infinity II SEC single detection system with mixed bed C PLgel columns (300 ×7.5 mm). In both instances, calibration was performed with narrow polystyrene standards.

A TA Instruments DSC 2500 was employed to conduct DSC experiments. About 5 mg of sample were sealed in an aluminum hermetic pan and heated to 200 °C. After equilibrating, the sample was cooled to - 80 °C, equilibrated, and heated to 200 °C. Data was obtained over three cycles of heating and cooling with a rate of 5 °C/min.

1.2. General polymerisation set up

In a glovebox, in an air-tight vial with a magnetic stirrer bar, complex **LZn_2Et** (11.0 mg, 13.9 μmol) was dissolved in dry toluene (0.30 mL). To this, 100 μL of a BnOH stock solution in dry toluene (15 mg/mL) was added. This solution was stirred at 70 °C for 30 mins using DrySyn heating blocks. Subsequently, the given amount of monomer dissolved in dry toluene (such that the total $[M]_0$ = 1.0 M) was pre-stirred at 70 ºC for 10 minutes before being added to the catalyst solution. The polymerisation was stirred at 70 °C for the required time, when an aliquot was taken and quenched in excess $CDCl_3$.

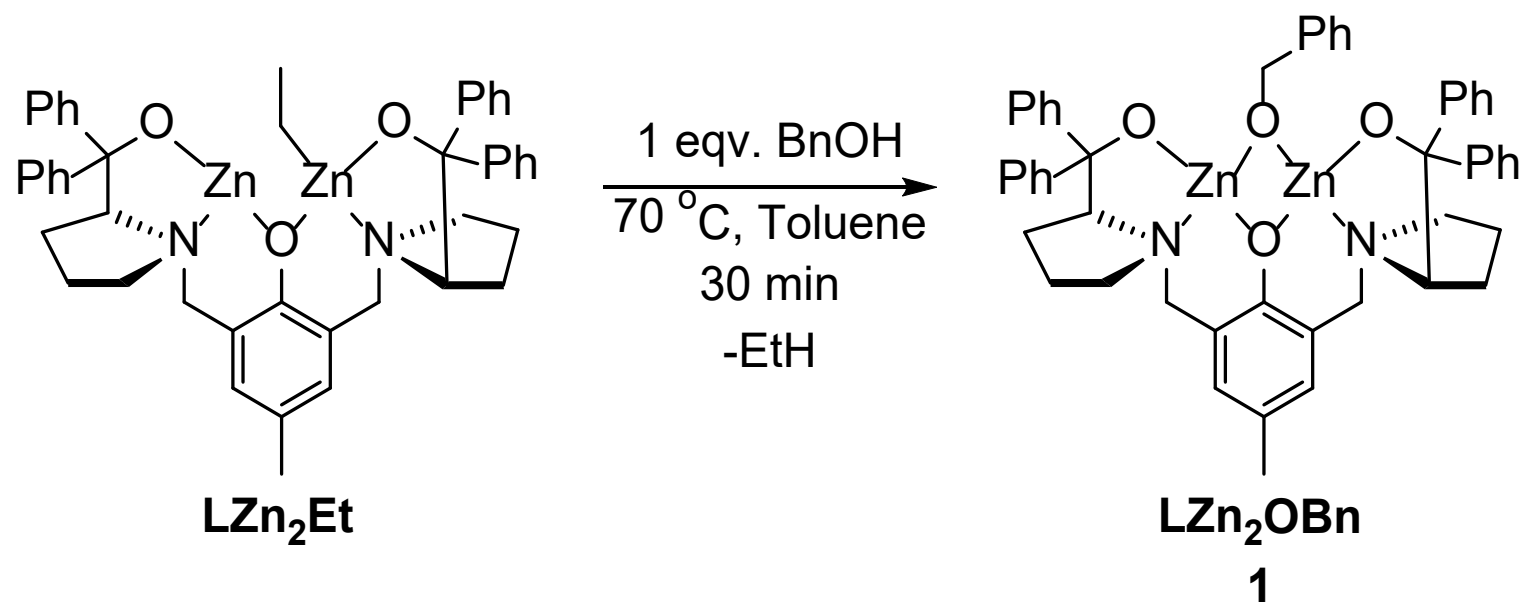

Scheme S1. Activation of pre-catalyst ***LZn_2Et*** *to form the active catalyst* ***1****.*

1.3. Calculating *R* values and average monomer sequence lengths for PGA/PCL copolymers

^{1}H NMR spectroscopy can be used to quantify the PGA-PCL copolymer structures produced after transesterification has taken place. Figure S1 shows a representative ^{1}H NMR spectrum for PGA-PCL copolymers, highlighting the relevant ^{1}H NMR resonances.

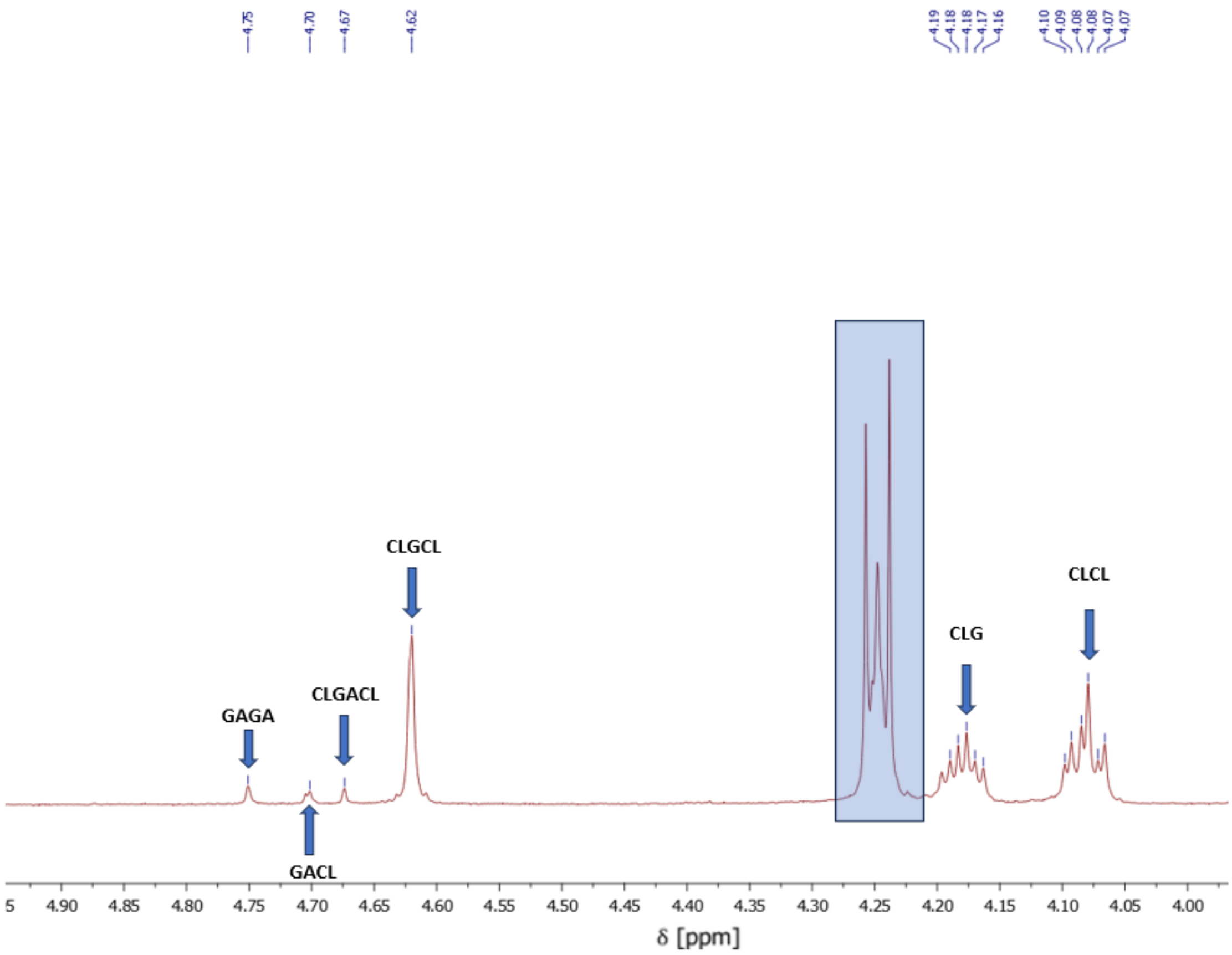

Figure S1. An example ^{1}H NMR spectra of an aliquot of PGA_{20}-PCL_{80} sample to demonstrate the information extracted from the experimental NMR spectra to calculate average sequence length l and randomness R. Unreacted CL is indicated in a blue box.

The average lengths l_e of glycolidyl (GA) and ε-caprolactyl (CL) blocks can be determined from integrations of the ^{1}H NMR spectra using the following equations.[2]

$$l_e^{GA} = \frac{1}{2}\frac{GA+GACL+CLGA-CL+CLGCL}{CLGCL+\frac{1}{2}(GACL+CLGACL)} \quad \text{(S1)}$$

$$l_e^{CL} = \frac{1}{2}\frac{CLG+CL}{CLG} \quad \text{(S2)}$$

A yield of the second mode of transesterification T_{II} can be calculated from

$$T_{II}=\frac{CLGCL}{CLGCL_R} \quad \text{(S3)}$$

$$CLGCL_R = \frac{k'^2}{(k'+1)^3} \quad \text{(S4)}$$

$$k'=\frac{1-N_G}{N_G} \quad \text{(S5)}$$

$$N_{GA}=\frac{1}{2}\frac{GA+GACL+CLGACL+CLGCL}{GA+GACL+CLGACL+CLGCL+CLG+CL} \quad \text{(S6)}$$

$$N_G = \frac{2N_{GA}}{N_{GA}+1} \quad \text{(S7)}$$

where [CLGCL] indicates the experimental concentration of CLGCL sequences in the copolymer chain, and $CLGCL_R$ indicates the concentration of CLGCL sequences in completely random chains. N_G and N_{GA} indicate, respectively, the concentration of glycolyl (G) and glycolidyl (GA) units in the copolymer chain, and k' denotes the ratio of the concentrations of caproyl to glycolyl units in the copolymer chain.

A degree of randomness of the copolymer chain can be calculated from the following equation:

$$R = \frac{l_R^{GA}}{l_e^{GA}} = \frac{l_R^{CL}}{l_e^{CL}} \quad \text{(S8)}$$

$$l_R^{GA} = \frac{(k'+1)}{2k'} \quad \text{(S9)}$$

$$l_R^{CL} = (k'+1) \quad \text{(S10)}$$

where l_R^{GA} and l_R^{CL} indicate, respectively, the average length of glycolidyl and ε-caprolactyl blocks in a completely random copolymer chain.

2. Polymerisations

2.1. Homopolymerisations

2.1.1. ε-Caprolactone (CL) ROP

Under an argon atmosphere in a glovebox, CL (157.5 mg, 152.9 µL, 1.38 mmol) was dissolved in dry toluene (1 mL). In a separate vial, **LZn_2Et** (11 mg, 13.9 µmol) was dissolved in dry toluene (0.39 mL) and BnOH (1.5 mg, 1.4 µL, 13.9 µmol) from a stock solution (0.5 µM in toluene) was added. The two solutions were stirred separately for 30 min at 70 °C, to ensure solubility and that both solutions were at the desired temperature. The two solutions were subsequently combined by adding the CL solution to the catalyst solution. Aliquots were taken at suitable times until the reaction was quenched with $CDCl_3$. The final product was obtained as a white powder by precipitation in cooled acidified methanol and compared with previously reported spectra.[1]

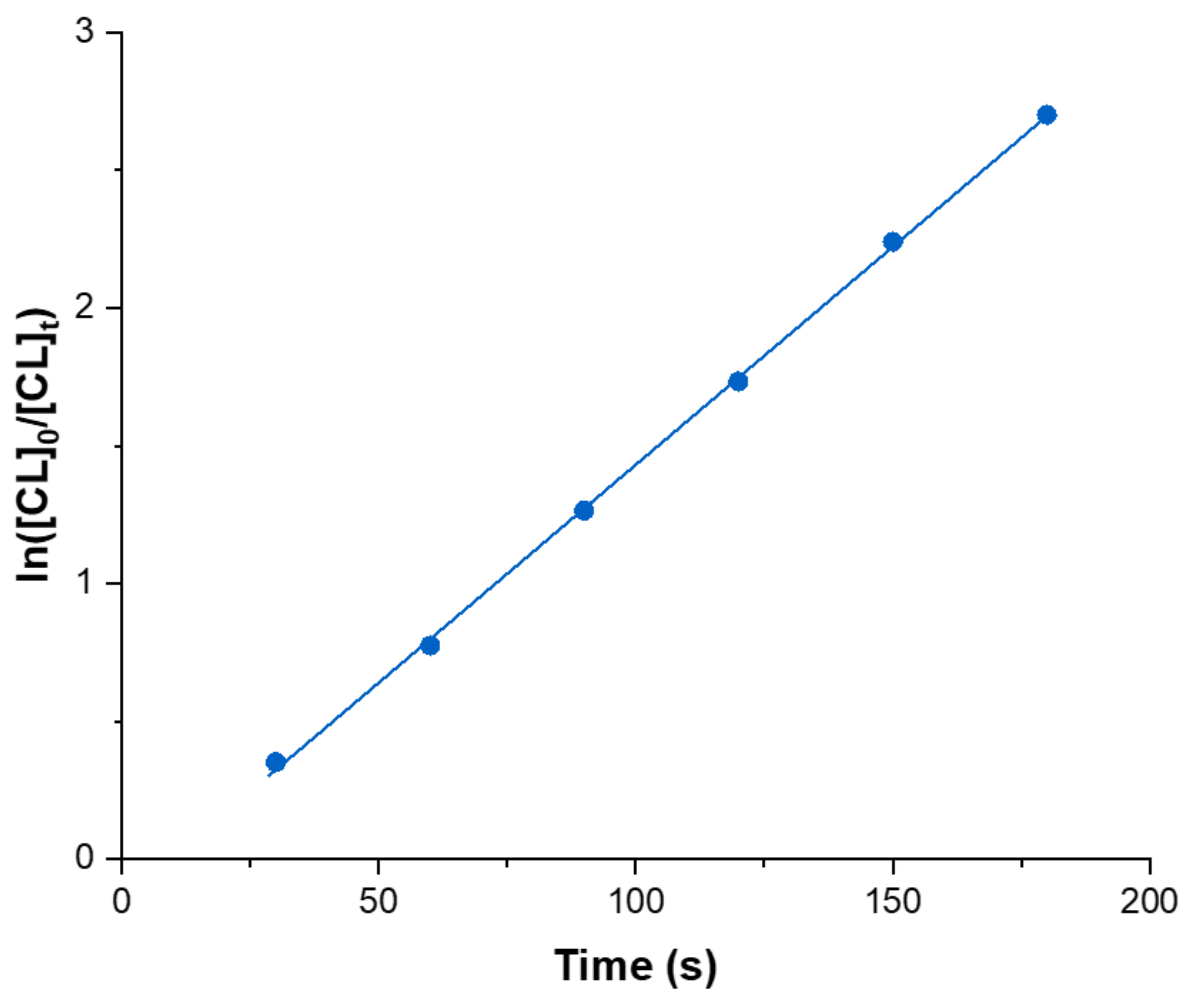

Figure S2: Kinetic plot for the ROP of CL (k_{obs} = 1.58 x 10^{-2} s^{-1}, R> 0.99).

2.1.2. *Rac*-Lactide (LA)

Under an argon atmosphere in a glovebox, *rac*-LA (200.1 mg, 1.38 mmol) was dissolved in dry toluene (1 mL). In a separate vial, **LZn_2Et** (11 mg, 13.9 µmol) was dissolved in dry toluene (0.39 mL) and BnOH (1.5 mg, 1.4 µL, 13.9 µmol) from a stock solution (0.5 µM in toluene) was added. The two solutions were stirred separately for 30 min at 70 °C, to ensure solubility and that both solutions were at the desired temperature. The two solutions were subsequently combined by adding the catalyst solution to the rac-LA solution. Aliquots were taken at suitable times until the reaction was quenched with $CDCl_3$. The final product was obtained as a white powder by precipitation in cooled acidified methanol and compared with previously reported spectra.[1]

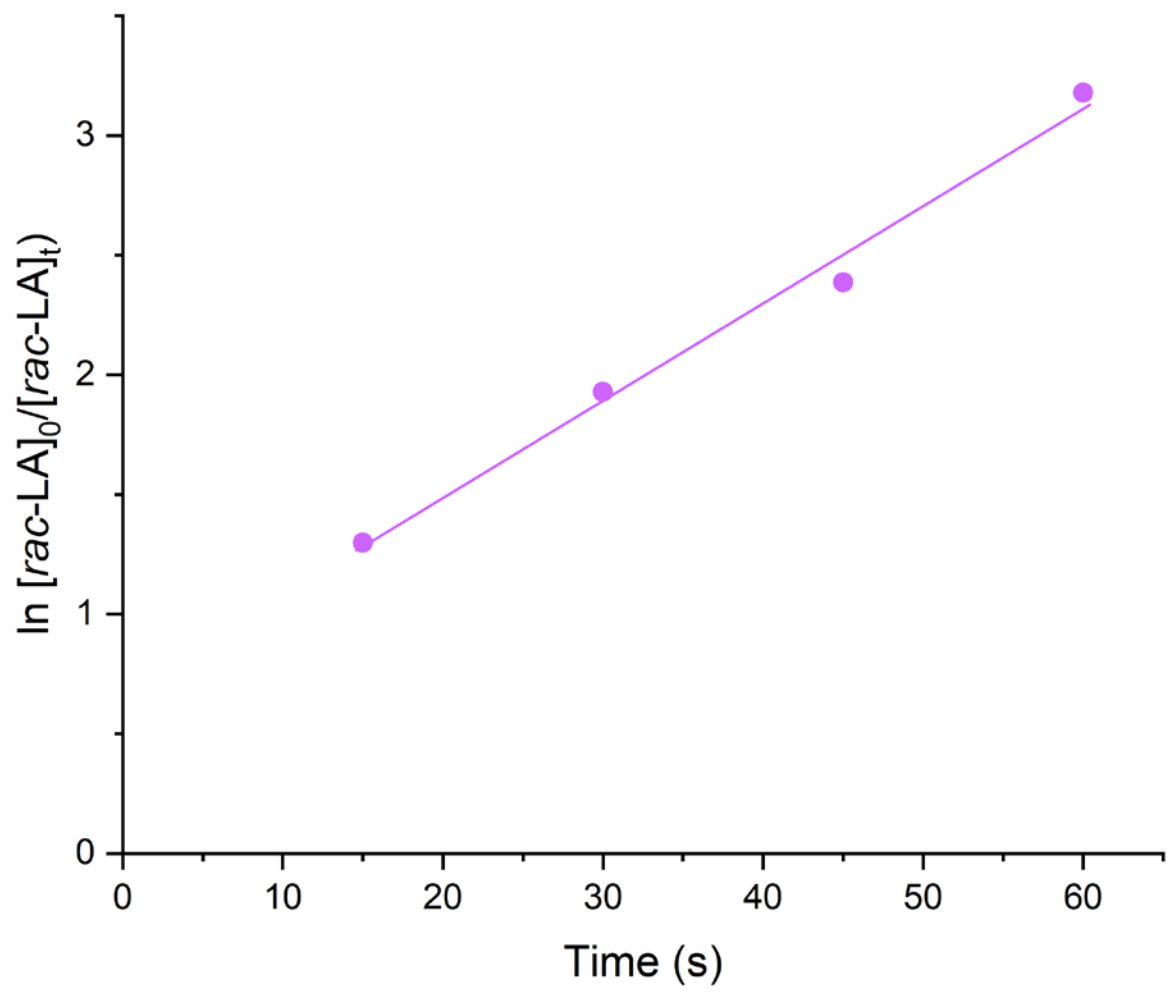

Figure S3: Kinetic plot for the ROP of rac-LA (k_{obs} = 4.07 x 10^{-2} s^{-1}, R = 0.99).

2.1.3. ε-Decalactone (DL) ROP

Under an argon atmosphere in a glovebox, DL (234.5 mg, 240.3 µL, 1.38 mmol) was dissolved in dry toluene (1 mL). In a separate vial, **LZn_2Et** (11 mg, 13.9 µmol) was dissolved in dry toluene (0.39 mL) and BnOH (1.5 mg, 1.4 µL, 13.9 µmol) from a stock solution (0.5 µM in toluene) was added. The two solutions were stirred separately for 30 min at 70 °C, to ensure solubility and that both solutions were at the desired temperature. The two solutions were subsequently combined by adding the DL solution to the catalyst solution. Aliquots were taken at suitable times until the reaction was quenched with $CDCl_3$. The final product was obtained as an oil by precipitation in cooled acidified methanol.

^{1}H NMR (500 MHz, THF-d_8): δ 4.88-4.83 (quint, 1H·n, -C*H*BuO-), 2.26-2.23 (t, 2H·n, -COCH_2-), 1.61-1.57 (m, 2H·n, -COCH$_2$CH_2-), 1.55-1.50 (m, 4H·n, -CH_2CHCH_2C$_3$H$_7$-), 1.37-1.25 (m, 4H·n, -CH_2-), 0.90-0.88 (m, 3H·n, -CH_3).

^{13}C NMR (126 MHz, THF-d_8): δ 173.1, 74.2, 34.9, 28.6, 26.0, 23.7, 14.6.

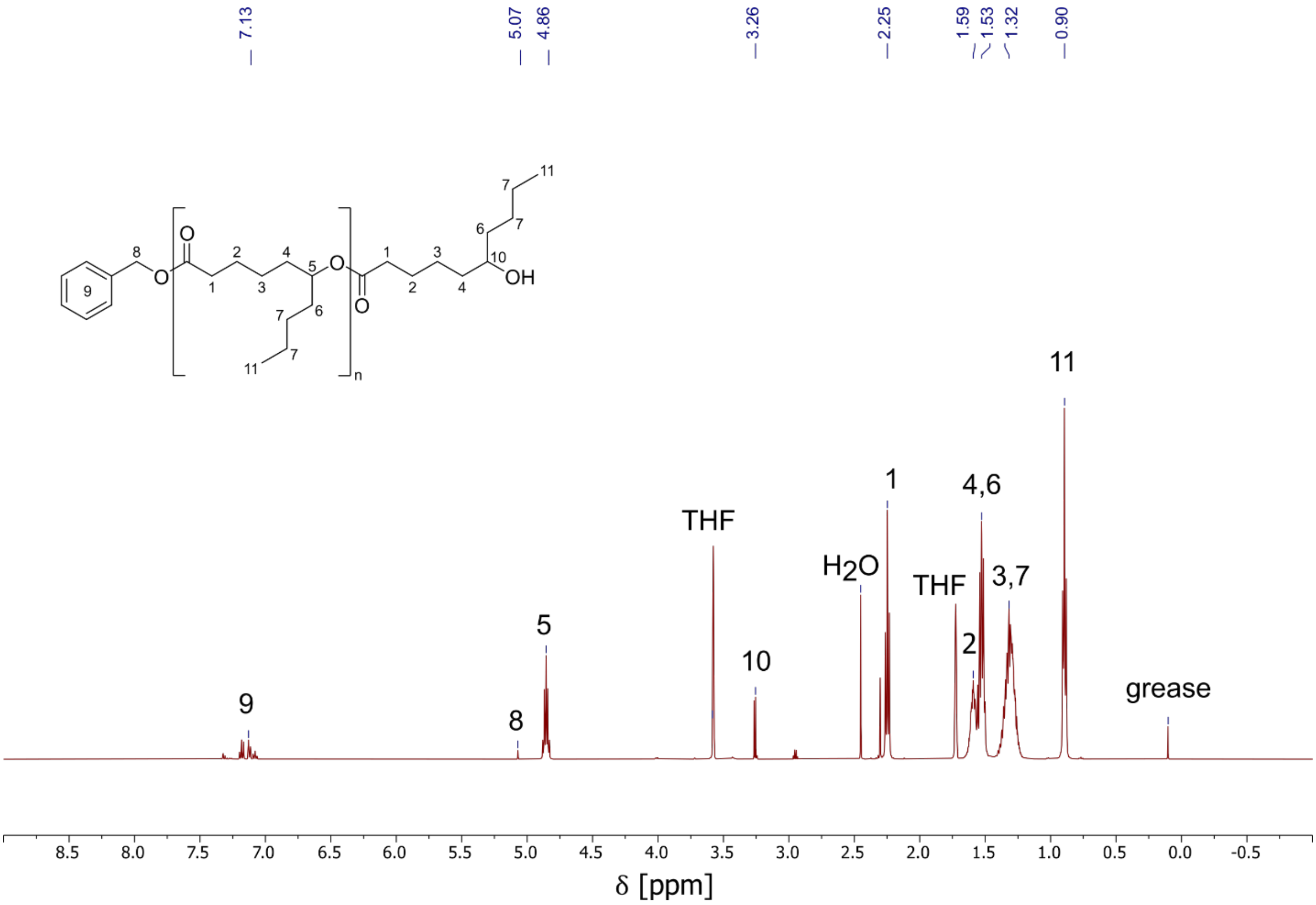

Figure S4. ^{1}H NMR spectrum (500 MHz, THF-d_8) of PDL.

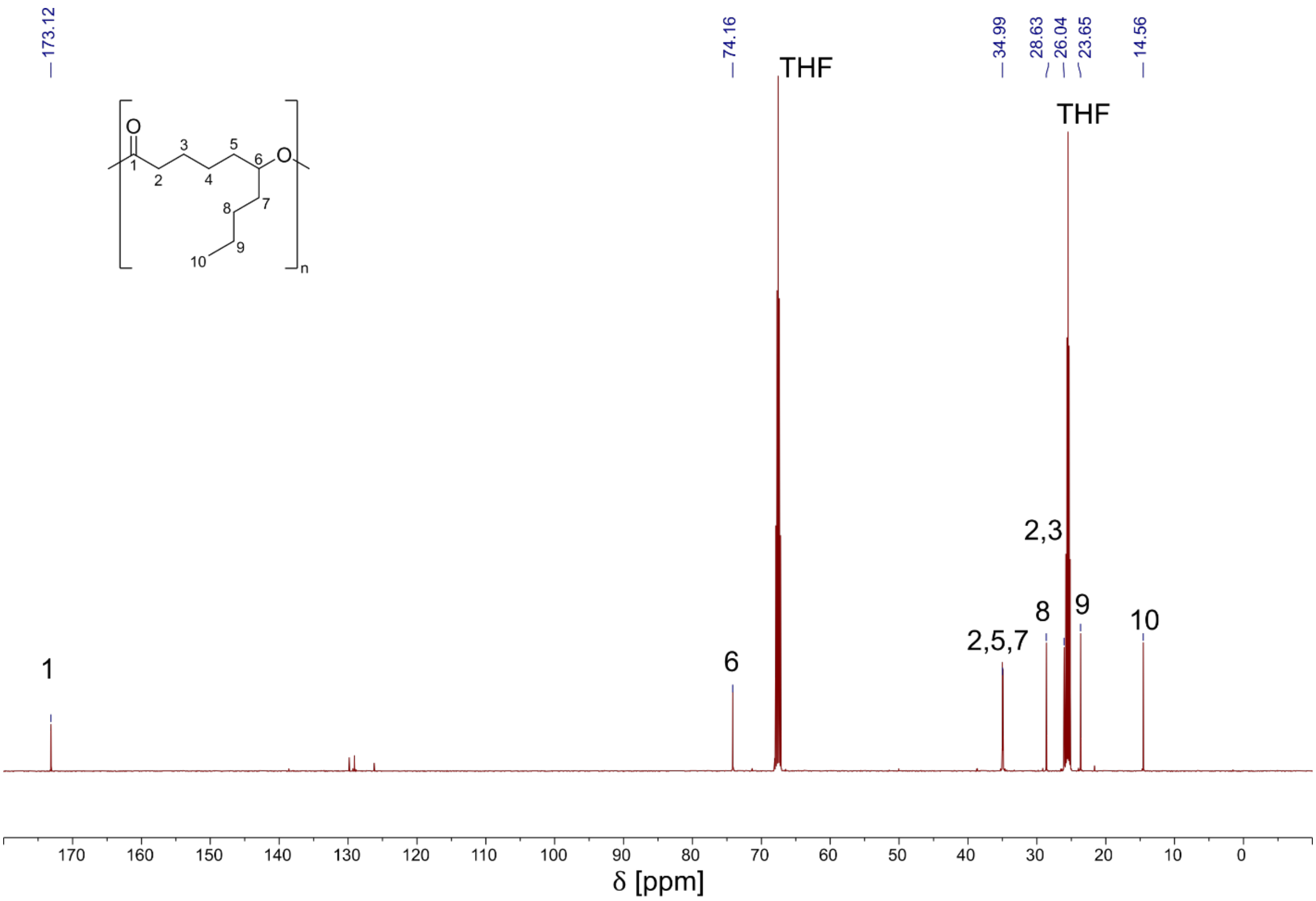

Figure S5.^{13}C NMR spectrum (126 MHz, THF-d_8) of PDL.

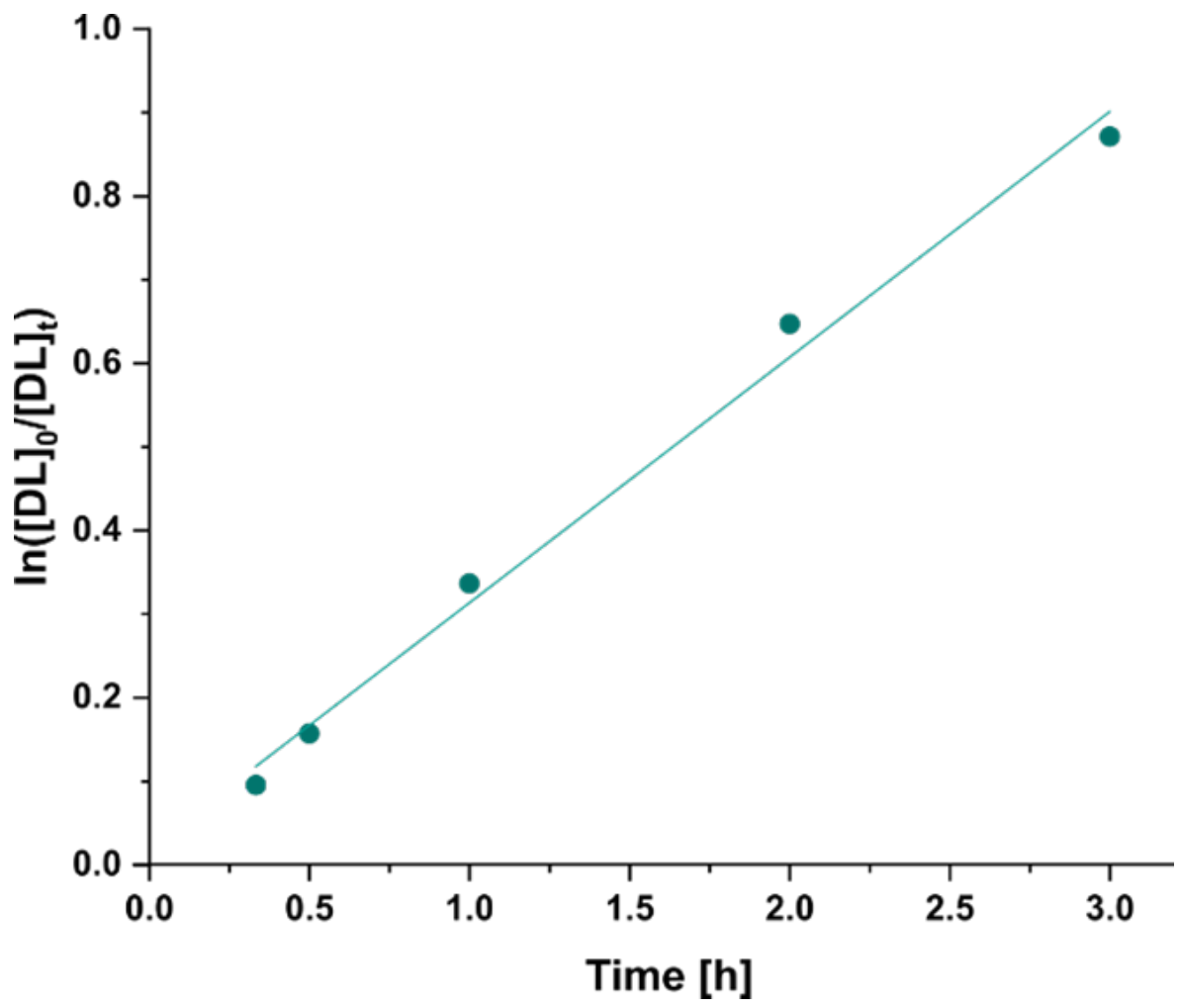

Figure S6. Kinetic plot for the homopolymerisation of DL (k_{obs} = 8.2 × 10^{-5} s^{-1}, R^2 = 0.99).

Table S1. Molecular weight and dispersity over time for the homopolymerisation of DL.

Entry	Time (h)	Conv.[a] (%)	$M_{n,theo}$[b] (kDa)	$M_{n,obs}$[c] (kDa)	$Đ$[c]
1	1	29	4.9	4.5	1.1
2	2	48	8.1	7.3	1.1
3	3	58	9.9	8.7	1.1
4	4.5	67	11.4	9.8	1.1
5	28	96	16.4	13.8	1.1

[a] determined from ^{1}H NMR
[b] calculated from conversion $M_{n,theo}$ = conversion × M_{DL}×($[DL]_0$/[**LZn_2OBn**])
[c] obtained by SEC in THF using a PS standard. Molecular weights are corrected by a factor of 0.91.

2.1.4. Pentadecalactone (pDL) ROP

Under an argon atmosphere in a glovebox, pDL (165 mg, 0.69 mmol) was dissolved in dry toluene (0.6 mL). In a separate vial, **LZn_2Et** (11 mg, 13.9 µmol) was dissolved in dry toluene (0.39 mL) and BnOH (1.5 mg, 1.4 µL, 13.9 µmol) from a stock solution (0.5 µM in toluene) was added. The two solutions were stirred separately for 30 min at 70 °C and then combined by adding pDL to the catalyst solution. Aliquots were taken at suitable times until the reaction was quenched with $CDCl_3$. The final product was obtained as an oil by precipitation in cooled acidified methanol.

^{1}H NMR (500 MHz, $CDCl_3$): δ 4.08-4.06 (t, 2H·n, -CH_2O-), 2.30 (t, 2H·n, -COCH_2-), 1.66-1.60 (m, 2H·n, -COCH$_2$CH_2-), 1.30-1.27 (m, 18H·n, -CH_2).

^{13}C NMR (126 MHz, $CDCl_3$): δ 174.0, 64.4, 34.4, 29.7 29.6, 29.6, 29.6, 29.5, 29.3, 29.2, 28.7, 26.0, 25.0.

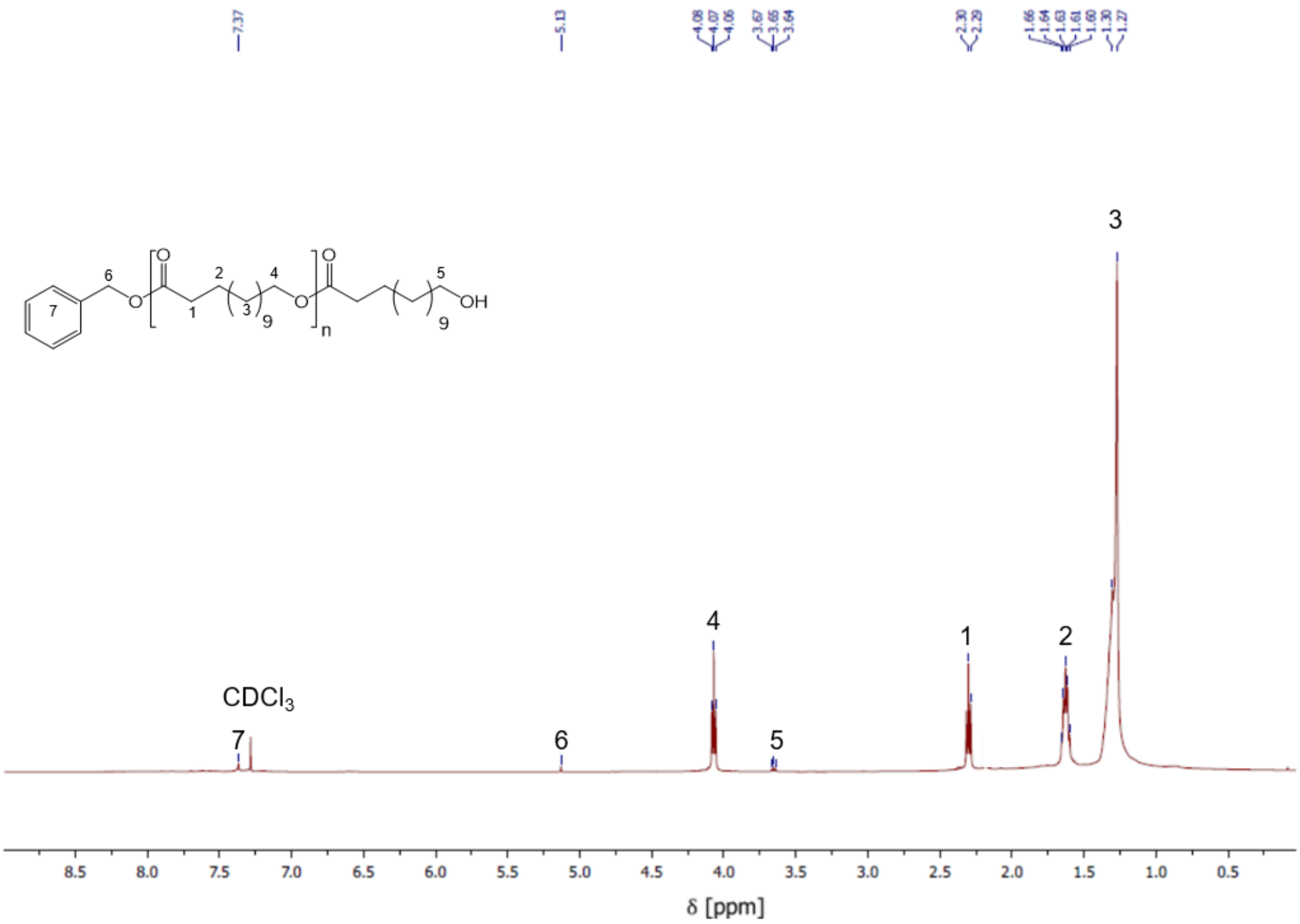

Figure S7. ^{1}H NMR spectrum (500 MHz, $CDCl_3$) of PpDL.

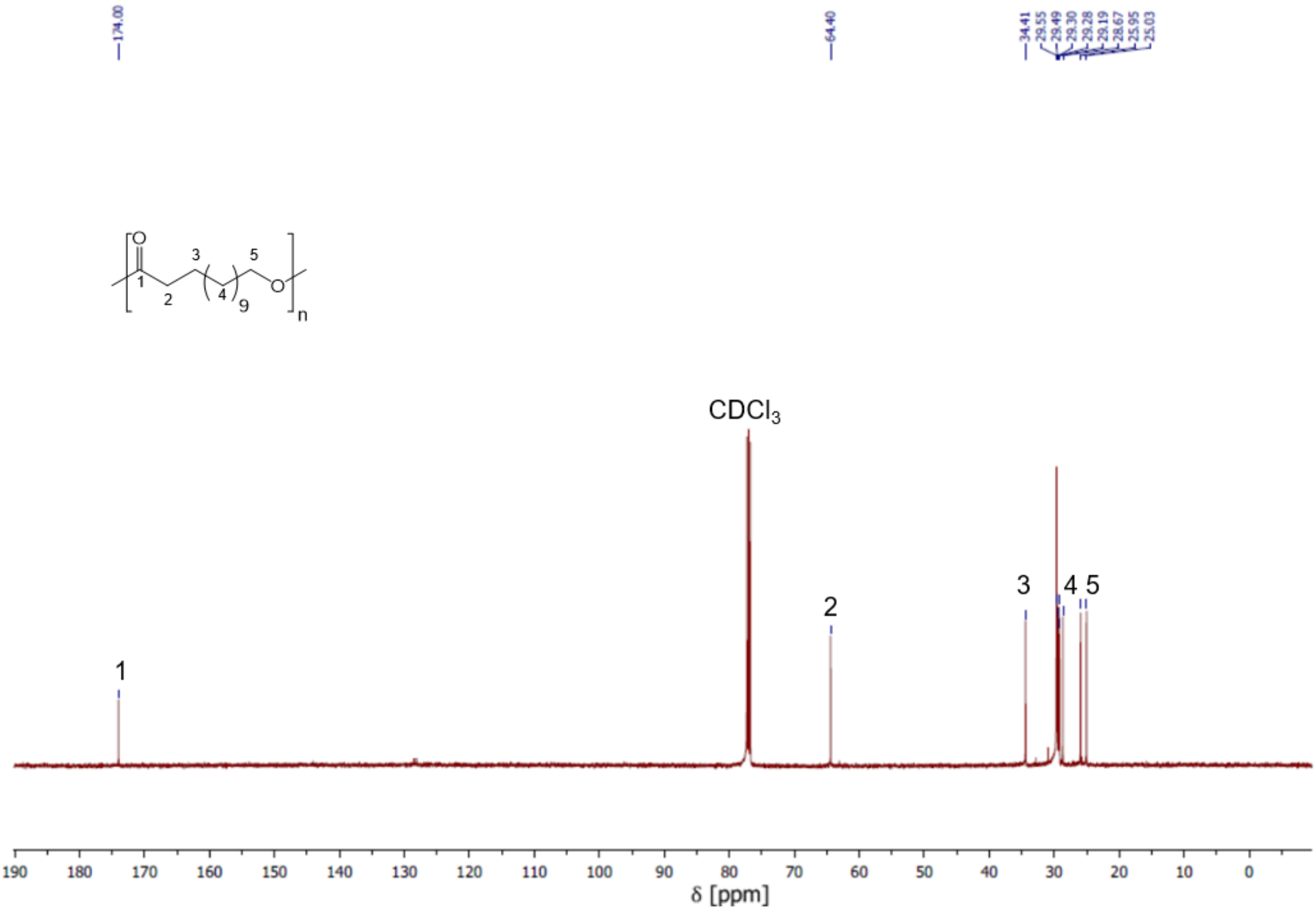

Figure S8. ¹H NMR spectrum (126 MHz, $CDCl_3$) of PpDL.

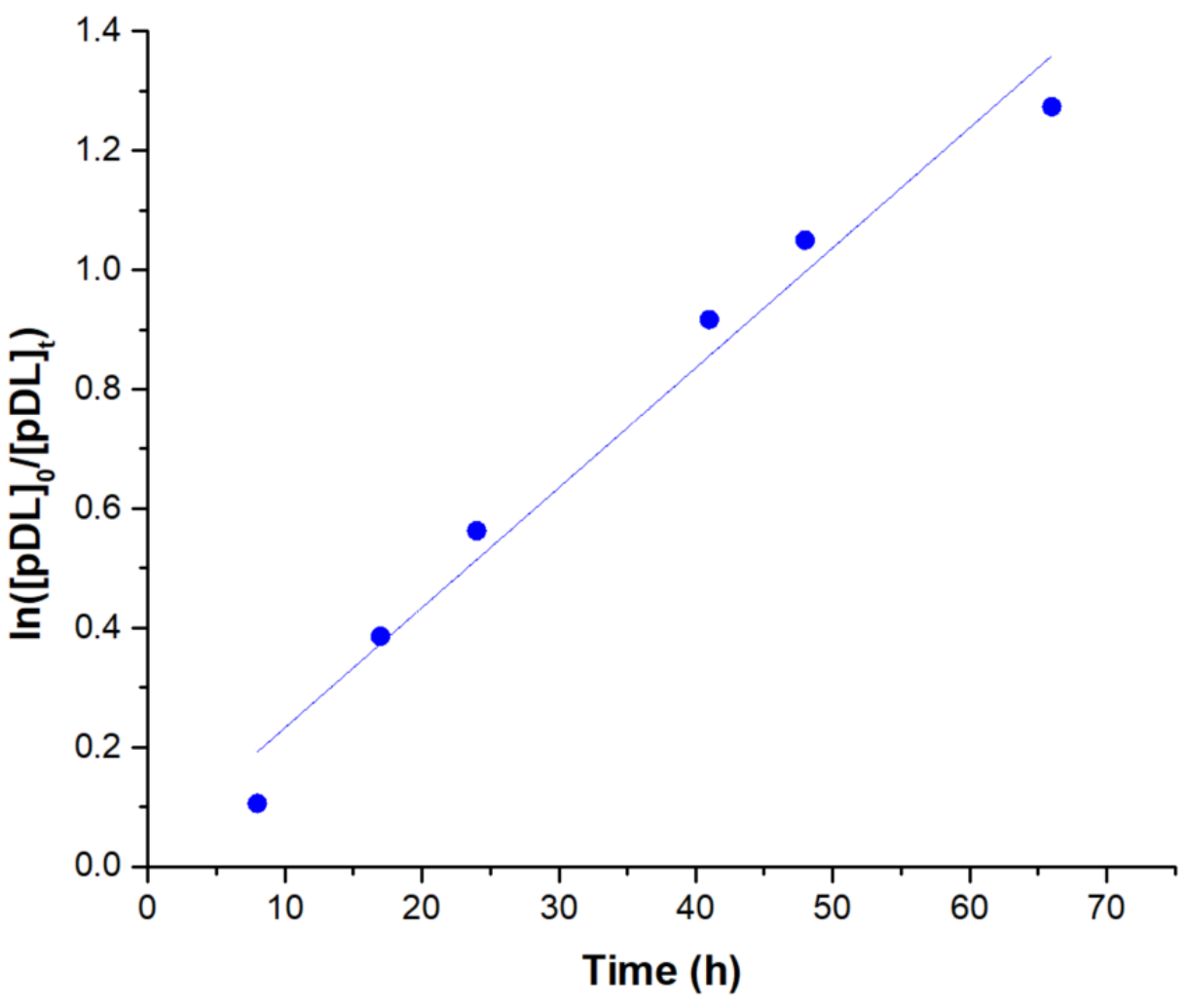

Figure S9. Kinetic plot for the homopolymerisation of pDL (k_{obs} = 3.2 x 10^{-2} h^{-1}, R^2 = 0.98).

2.1.5. δ-Valerolactone (VL) ROP

Under an argon atmosphere in a glovebox, VL (138.0 mg, 1.38 mmol) was dissolved in dry toluene (1 mL). In a separate vial, **LZn_2Et** (11 mg, 13.9 μmol) was dissolved in dry toluene (0.39 mL) and BnOH (1.5 mg, 1.4 μL, 13.9 μmol) from a stock solution (0.5 μM in toluene) was added. The two solutions were stirred separately for 30 min at 70 °C and then combined by adding the VL solution to the catalyst solution. Aliquots were taken at suitable times until the reaction was quenched with $CDCl_3$. The final product was obtained as a white solid in cooled acidified methanol.

^{1}H NMR (500 MHz, $CDCl_3$) δ 4.07 (t, 2H·n, $-CH_2O-$), , 2.39-2.31 (m, 2H, $-CH_2CO-$), 1.72-1.63 (m, 4H·n, $-CH_2CH_2CH_2O-$).

^{13}C NMR (126 MHz, $CDCl_3$) δ 173.4, 64.1, 33.8, 28.2, 21.6.

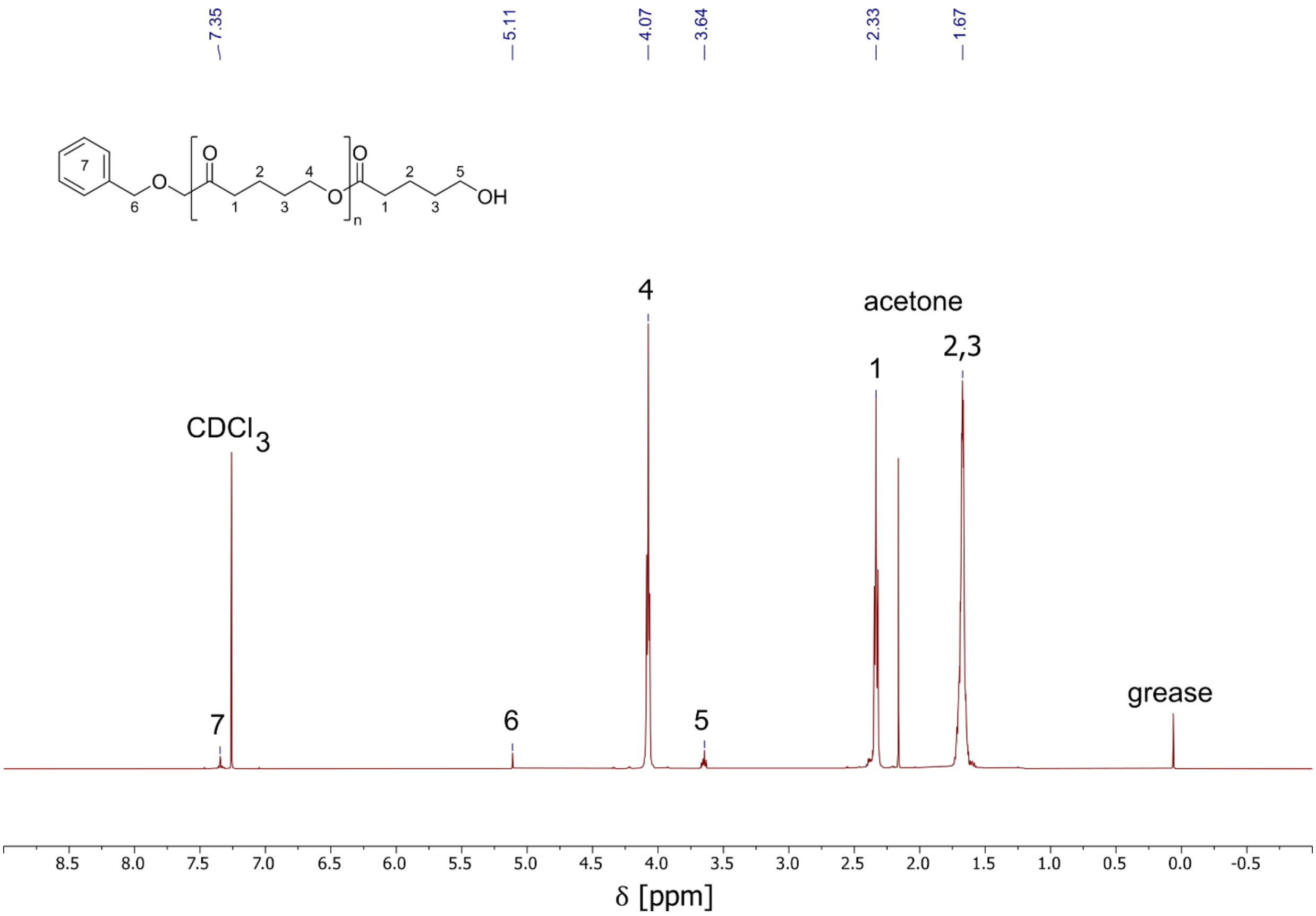

Figure S10. ^{1}H NMR spectrum (500 MHz, $CDCl_3$) of PVL.

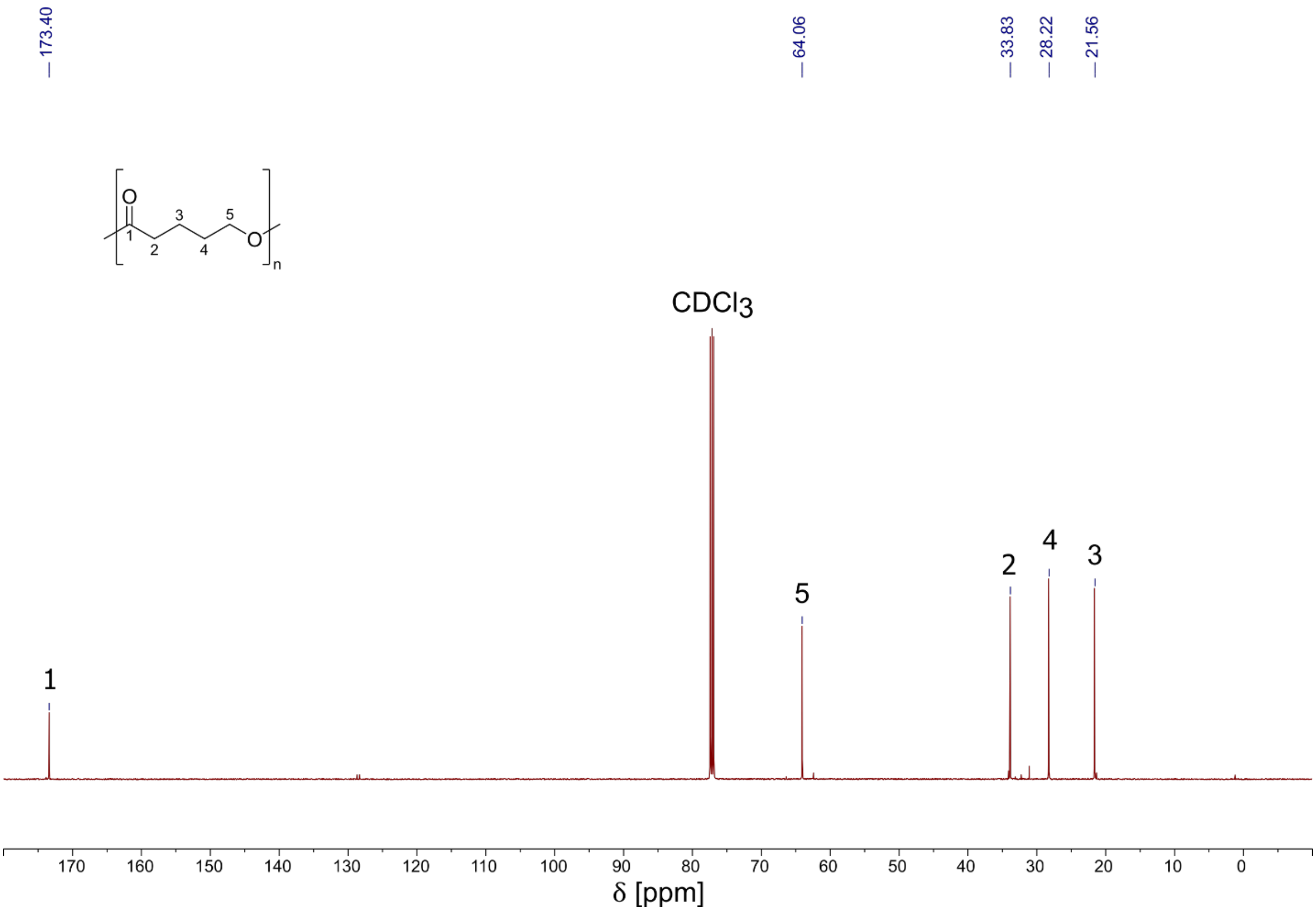

Figure S11. ^{13}C NMR spectrum (126 MHz, $CDCl_3$) of PVL.

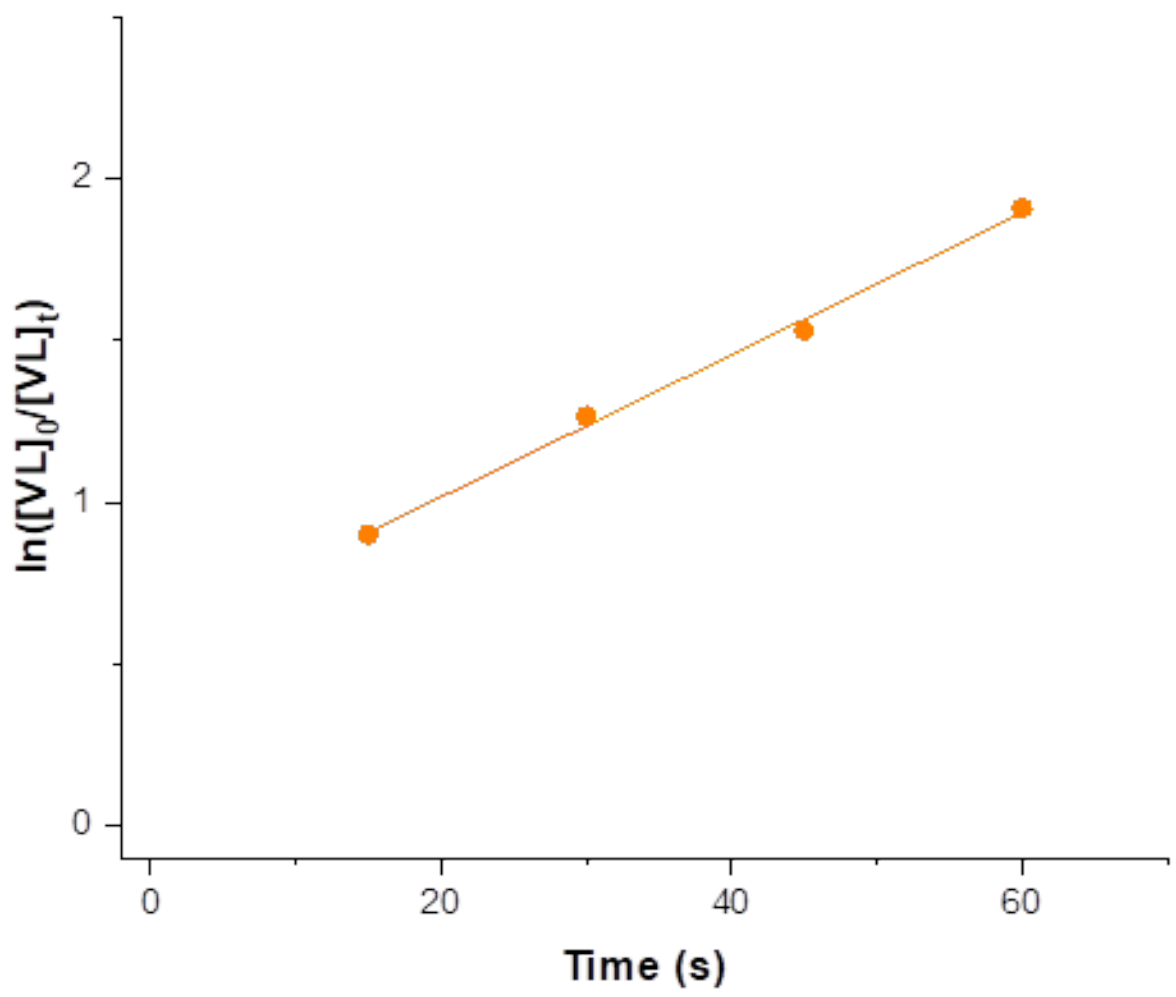

Figure S12. Kinetic plot for the homopolymerisation of VL (k_{obs} = 2.2 × 10^{-2} s^{-1}, R^2 > 0.99).

2.1.6. Trimethylene carbonate (TMC) ROP

Under an argon atmosphere in a glovebox, TMC (140.8 mg, 1.38 mmol) was dissolved in dry toluene (1 mL). In a separate vial, **LZn_2Et** (11 mg, 13.9 μmol) was dissolved in dry toluene (0.39 mL) and BnOH (1.5 mg, 1.4 μL, 13.9 μmol) from a stock solution (0.5 μM in toluene) was added. The two solutions were stirred separately for 30 min at 70 °C and then combined by adding the TMC solution to the catalyst solution. Aliquots were taken at suitable times until the reaction was quenched with $CDCl_3$. The final product was obtained as a white solid in cooled acidified methanol.

^{1}H NMR (500 MHz, $CDCl_3$) δ 4.33-4.30 (t, 2H, -CH_2O-), 4.27-4.24 (t, 2H·n, -CH_2O), 2.08-2.04 (t, 2H·n, -CH_2CH$_2$O).

^{13}C NMR (126 MHz, $CDCl_3$) δ 154.9, 64.3, 28.0.

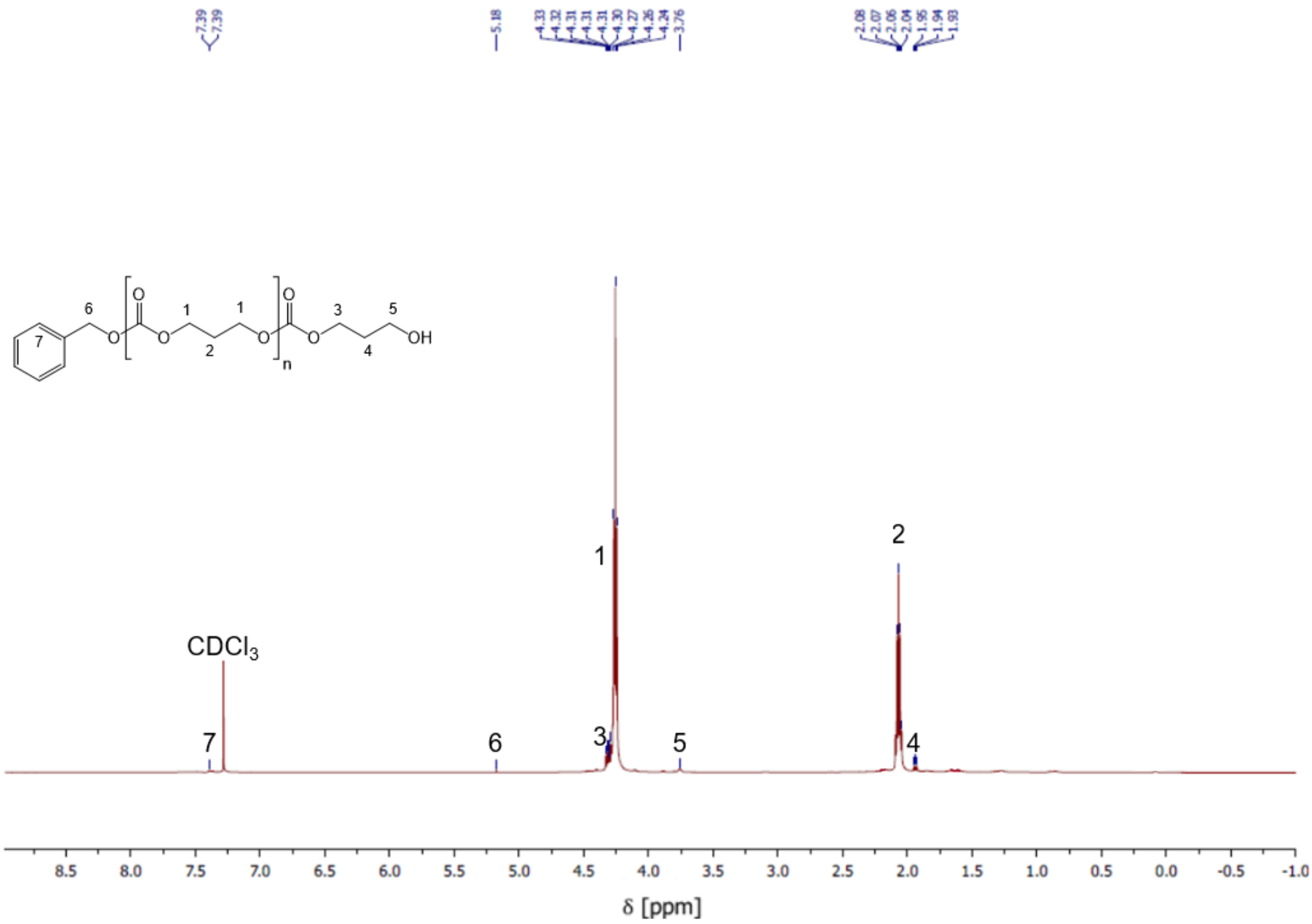

Figure S13. ^{1}H NMR spectrum (500 MHz, $CDCl_3$) of PTMC.

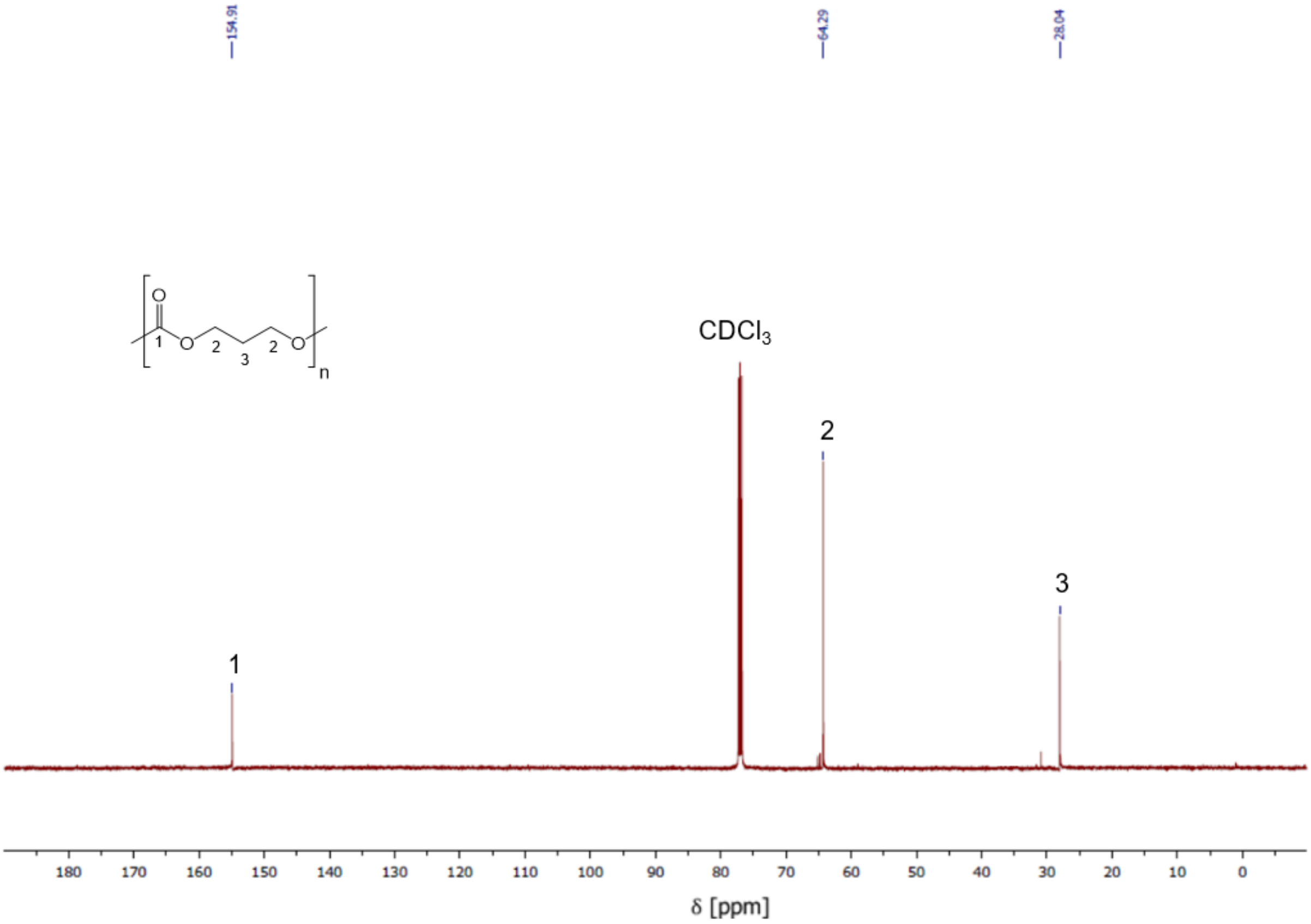

Figure S14. ^{13}*C NMR spectrum (126 MHz,* $CDCl_3$*) of PTMC.*

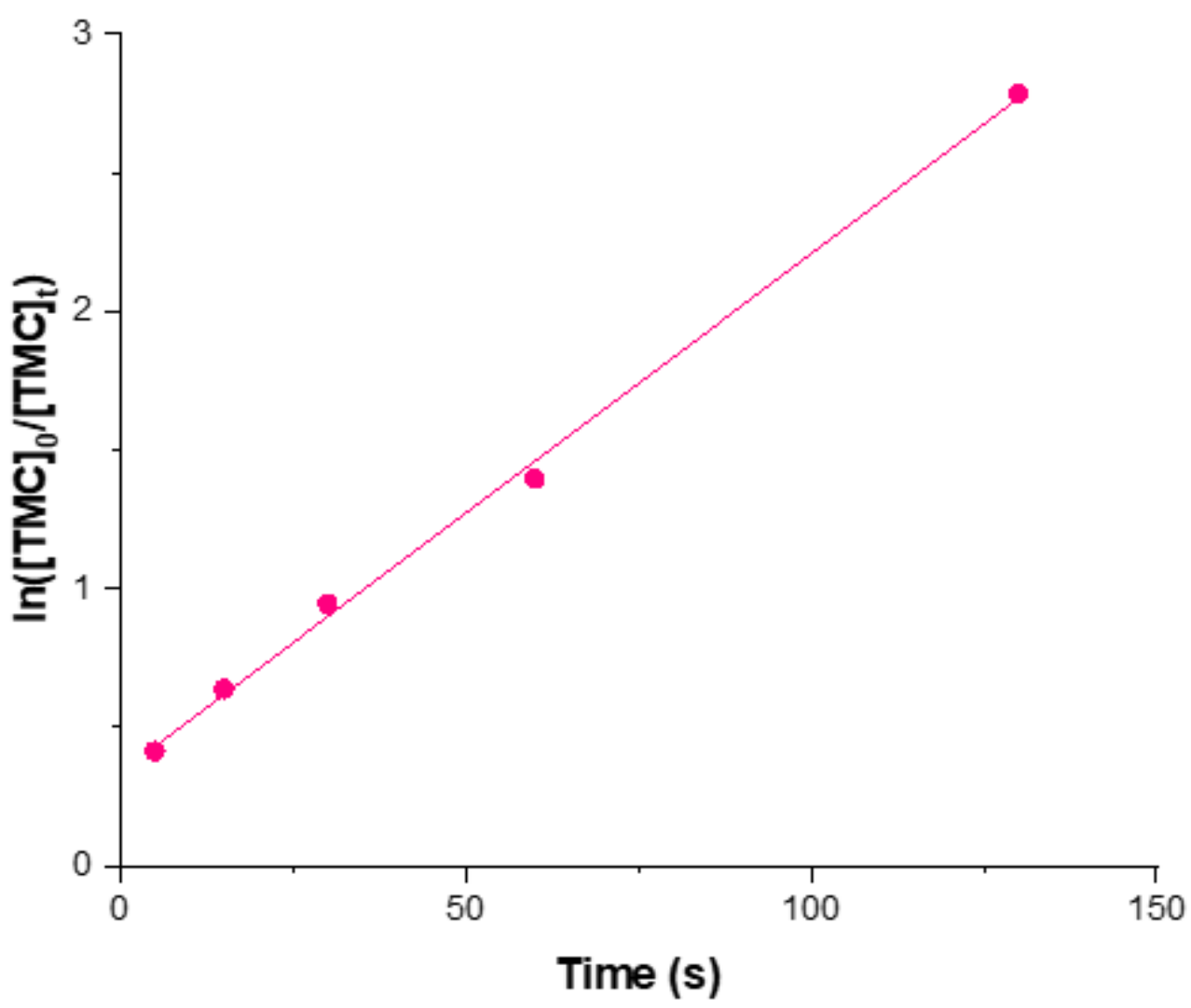

Figure S15. Kinetic plot for the homopolymerisation of TMC ($k_{obs} = 1.9 \times 10^{-2}\ s^{-1}$*,* $R^2 > 0.99$*).*

2.1.7. Overview of k_{obs} data

Below is an overview of the k_{obs} data for homopolymerisation of the lactones considered in this study with bis-Zn catalyst **LZn_2OBn 1** at 1 mol% loading, except from pentadecalactone at 2 mol% loading. Note that GA and *p*DO data was not collected due to issues with the homopolymer solubility in common organic solvents. All kinetics were recorded at 1 M monomer concentration in toluene solvent at 70 ºC, after a 1:1 mixture of **LZn_2Et** and BnOH were stirred together in toluene solvent for 30 mins at 70 ºC to generate **LZn_2OBn** *in situ*.

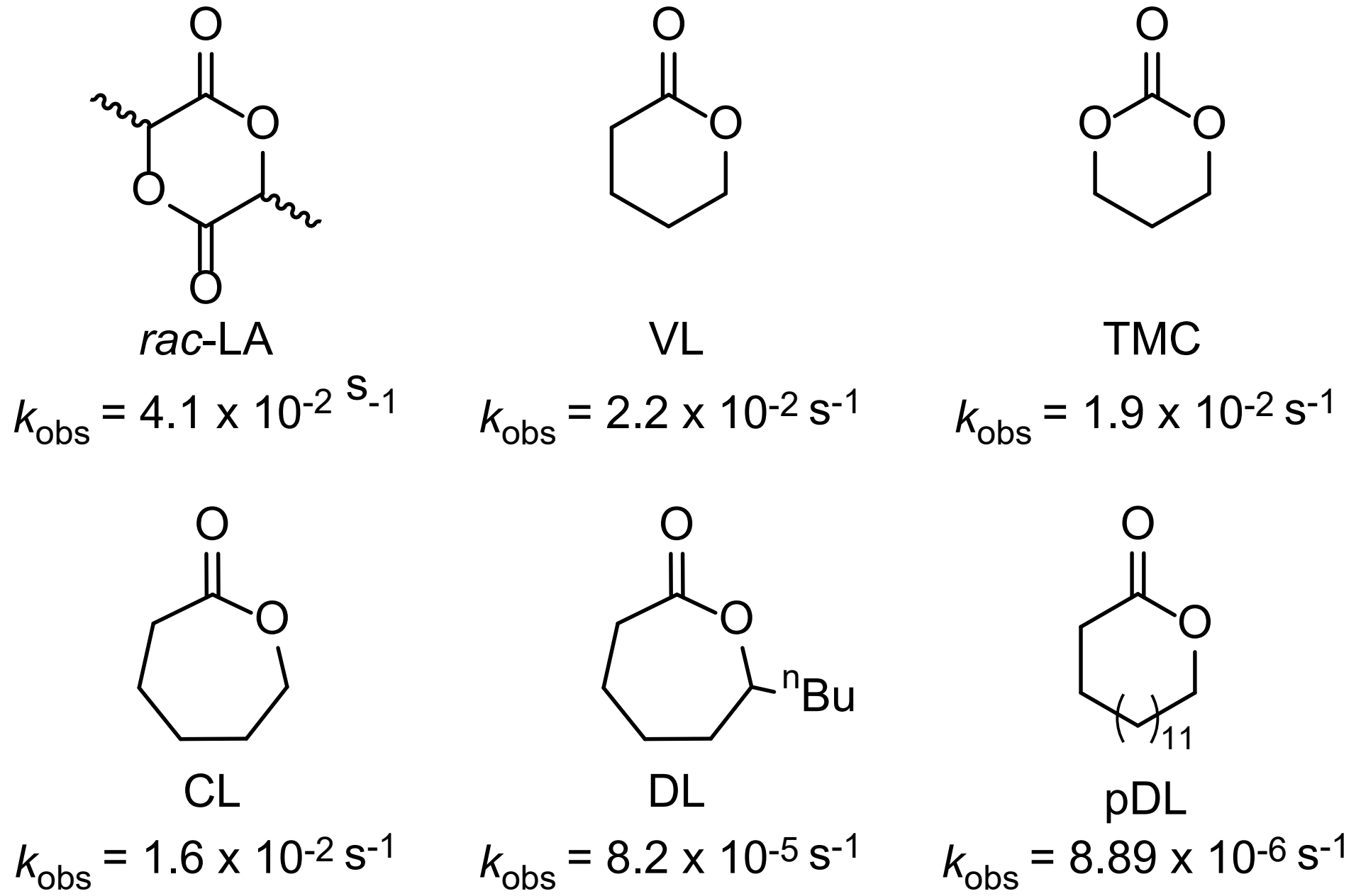

*Figure S16. Monomer structures and k_{obs} for ROP with **LZn_2OBn** under identical conditions (70 ºC, toluene, $[M]_0$ = 1 M, 1 mol% catalyst loading) except from pDL which was performed at 2 mol% catalyst loading.*

2.2. Sequential and simultaneous copolymerisation: ring size and α-substituent

2.2.1. PCL-PGA diblocks

2.2.1.1. Sequential addition: CL first

Under an argon atmosphere in a glovebox, CL was dissolved in dry toluene (0.2 mL). In a separate vial, **LZn_2Et** (11 mg, 13.9 µmol) was dissolved in dry toluene (0.1 mL) and BnOH (1.5 mg, 1.4 µL, 13.9 µmol) from a stock solution (0.5 µM in toluene) was added. The two solutions were stirred separately for 30 min at 70 °C and then combined by adding CL to the catalyst solution. An aliquot was taken and the reaction mixture was added to GA (30 mg, 0.27 mmol) in toluene (2 mL), previously stirred at 85 °C. The reaction was quenched with $CDCl_3$ and the final product was obtained as a white solid in cooled acidified methanol.

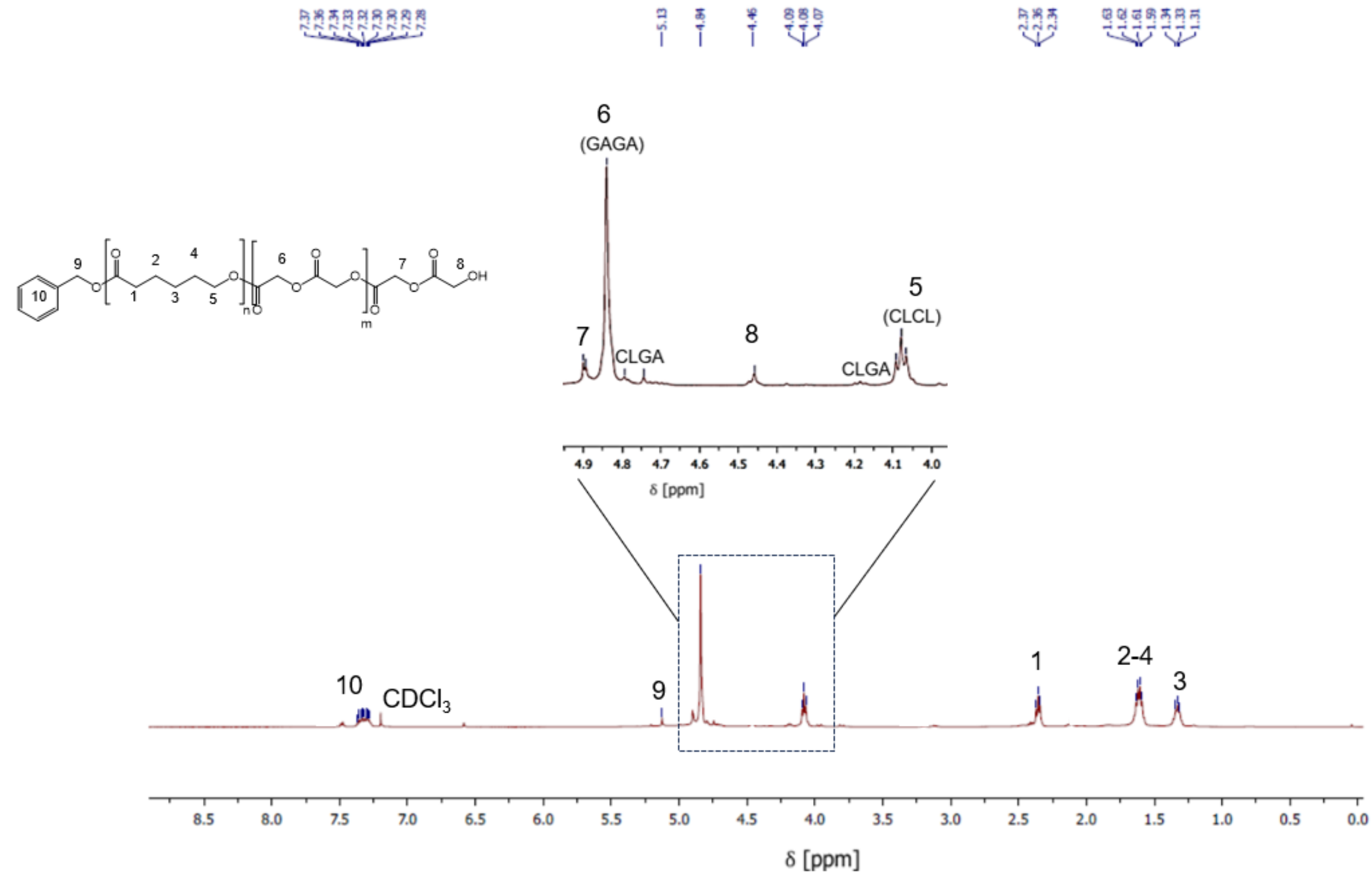

Figure S17. ^{1}H NMR spectrum (500 MHz, $CDCl_3$ + TFA)of PCL_{20}^{*} with 20 eq. of GA (note: TFA was added to enhance the solubility of the resultant PCL_{20}-PGA_{20} copolymers) (Table 1, entry 1).

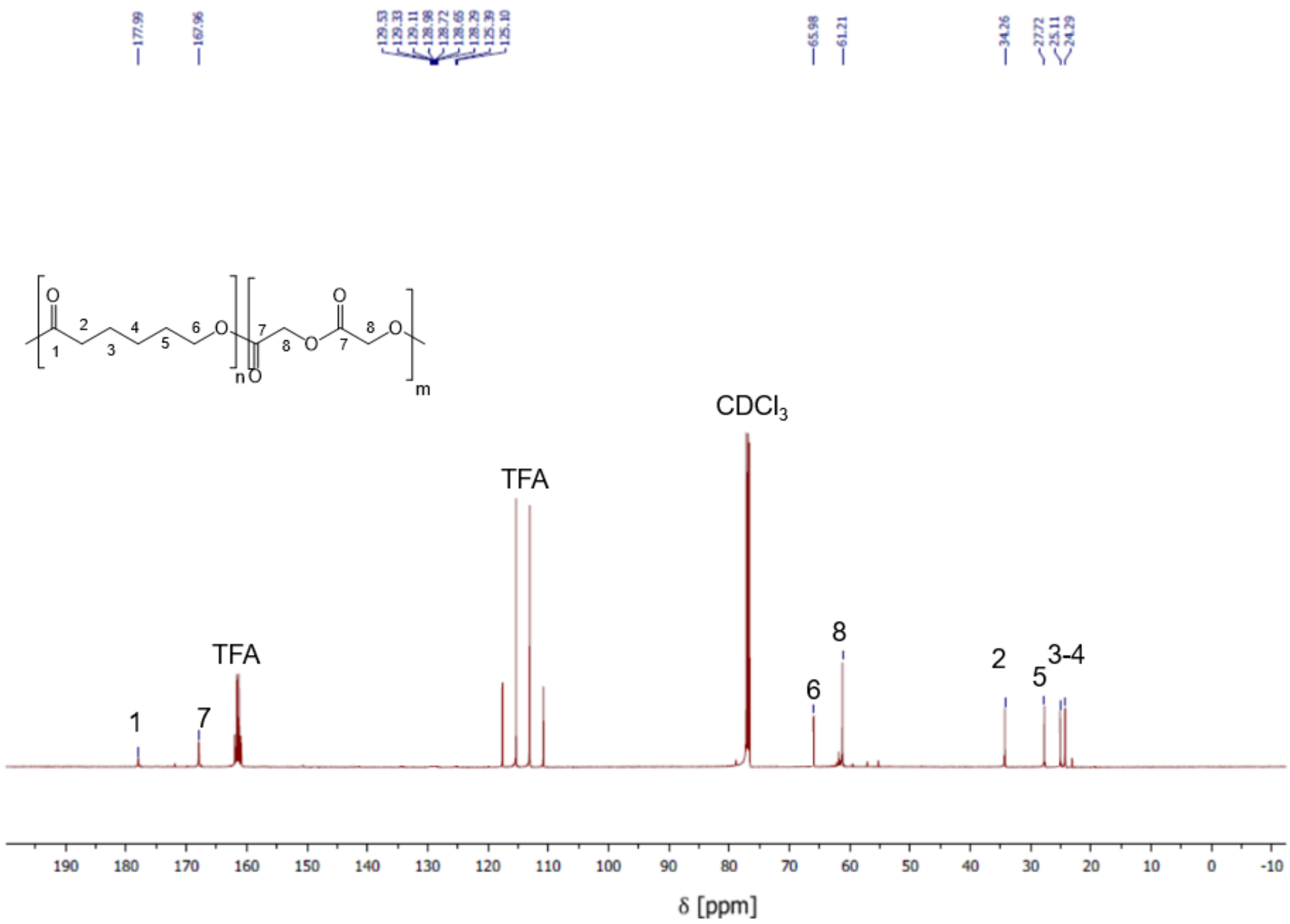

Figure S18. Quantitative ^{13}C NMR spectrum (126 MHz, $CDCl_3$ + TFA) of PCL_{20}^{*} with 20 eq. of GA (note: TFA was added to enhance the solubility of the resultant PCL_{20}-PGA_{20} copolymers) (Table 1, entry 1).

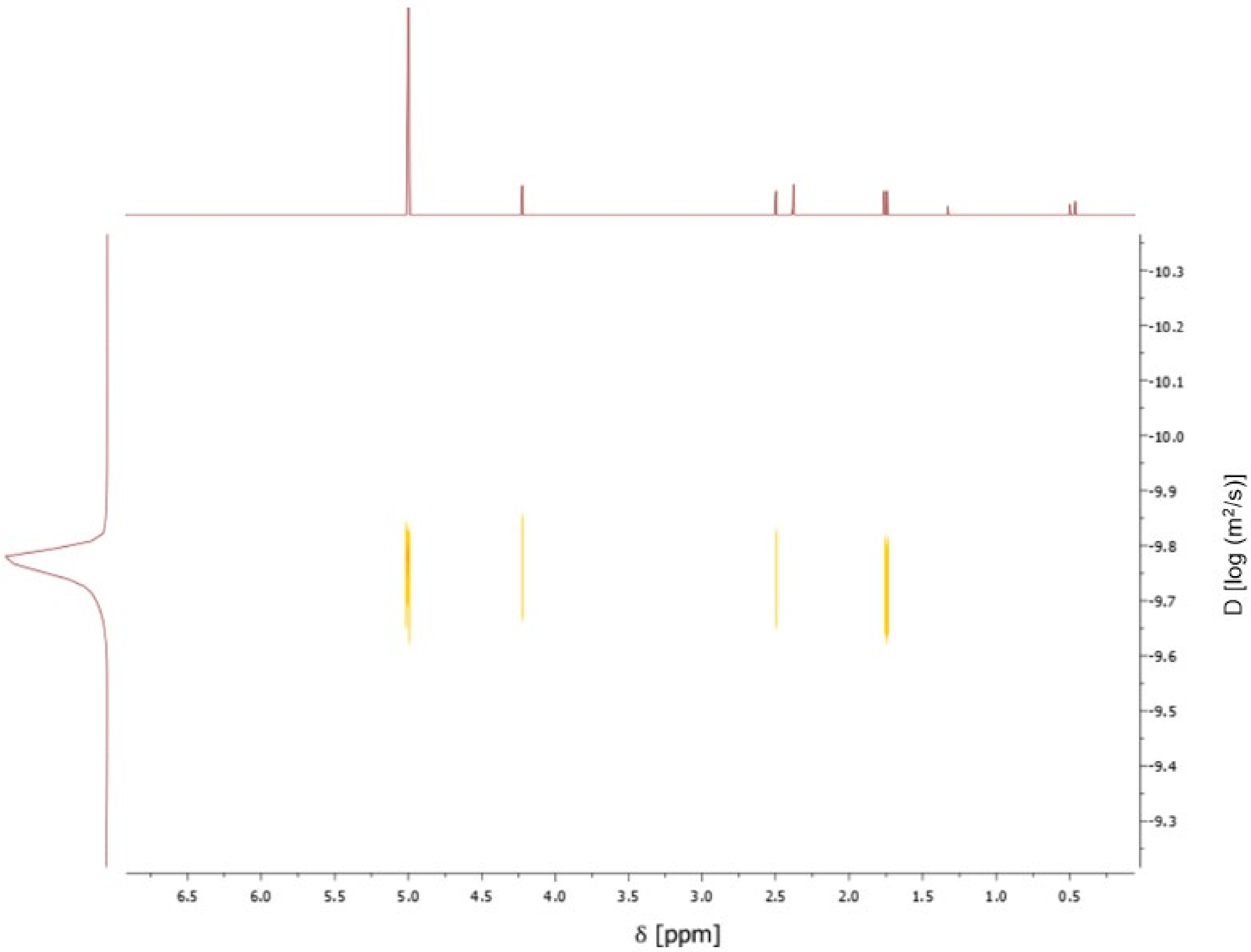

Figure S19. ^{1}H DOSY NMR spectrum (500 MHz, $CDCl_3$ + TFA) of PCL_{20}^* with 20 eq. of GA.

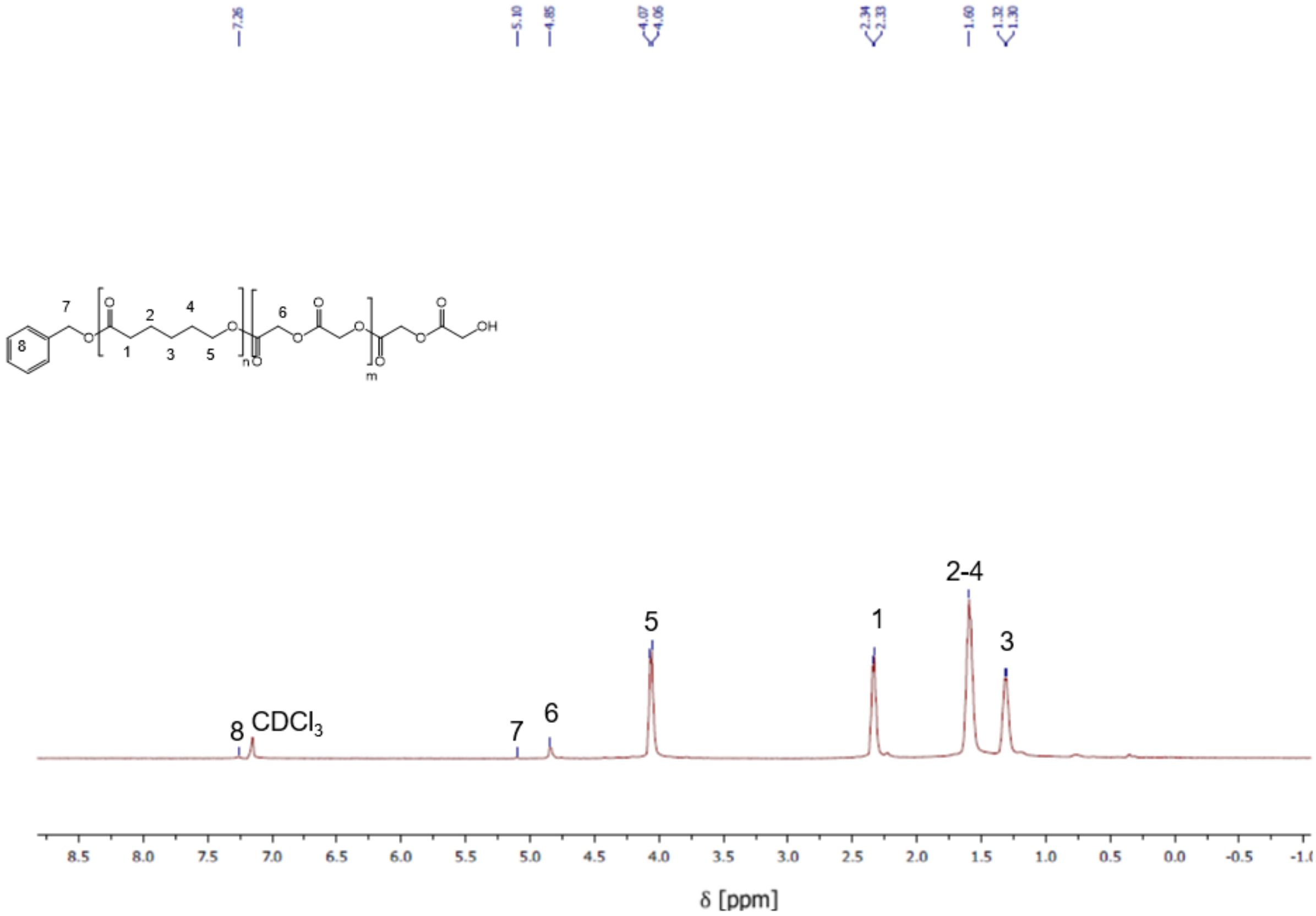

Figure S20. ^{1}H NMR spectrum (500 MHz, $CDCl_3$ + TFA) of PCL_{180}^{*} with 20 eq. of GA (note: TFA was added to enhance the solubility of the resultant PCL_{180}-PGA_{20} copolymers) (Table 1, entry 2).

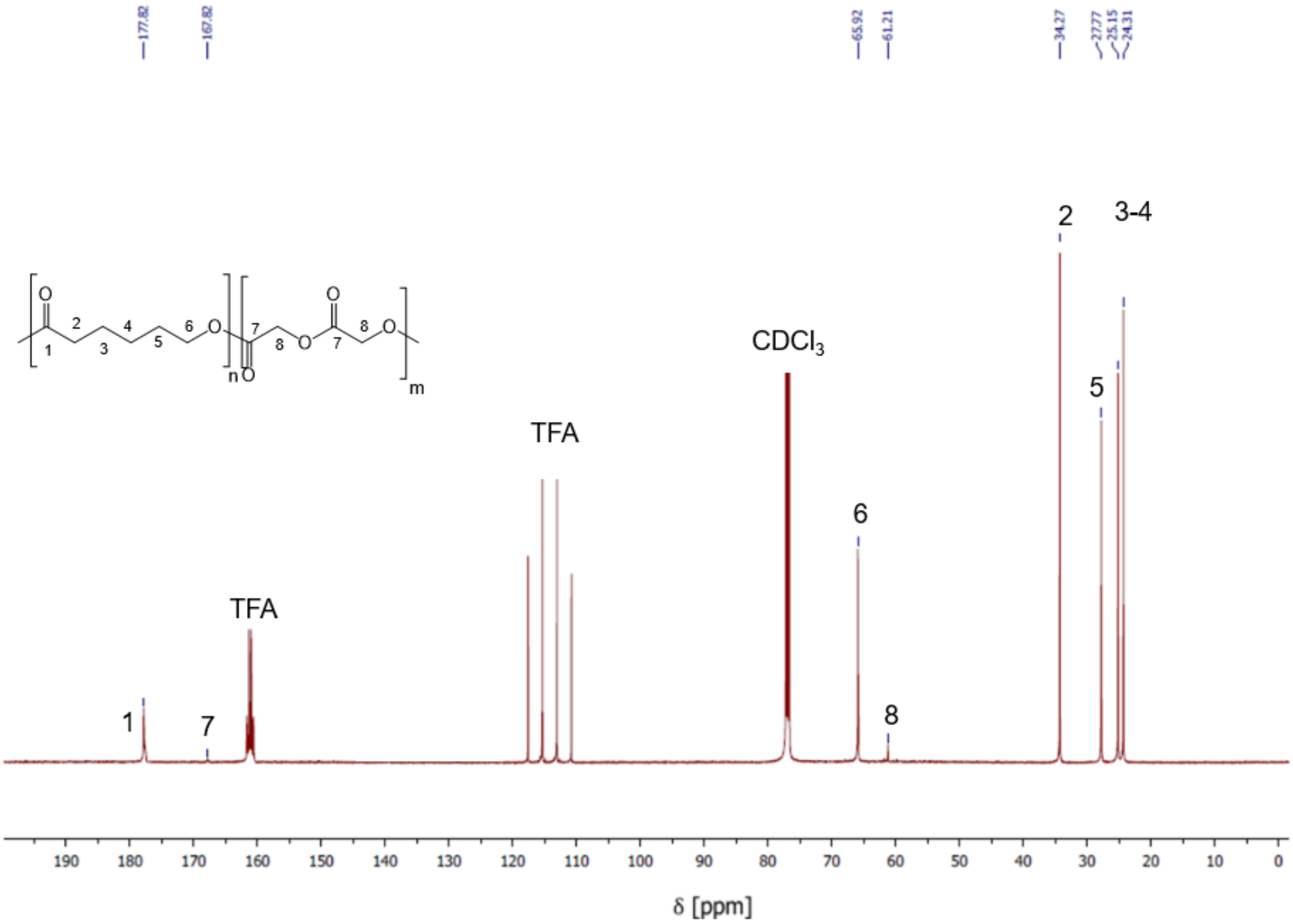

Figure S21. Quantitative [13]*C NMR spectrum (126 MHz, $CDCl_3$ + TFA) of PCL_{180}^{*} with 20 eq. of GA (note: TFA was added to enhance the solubility of the resultant PCL_{180}-PGA_{20} copolymers) (Table 1, entry 2).*

2.2.1.2. Sequential addition: GA first

Under an argon atmosphere in a glovebox, GA (30 mg, 0.27 mmol) was melted at 85 °C. In a separate vial, **LZn_2Et** (11 mg, 13.9 µmol) was dissolved in dry toluene (0.1 mL) and BnOH (1.5 mg, 1.4 µL, 13.9 µmol) from a stock solution (0.5 µM in toluene) was added. The solution was stirred separately for 30 min and then added to the molten GA. An aliquot was taken and neat CL (30 mg, 0.27 mmol) was added to the reaction mixture. The reaction was quenched with $CDCl_3$ and the final product was obtained as a white solid by precipitation in cold, acidified methanol.

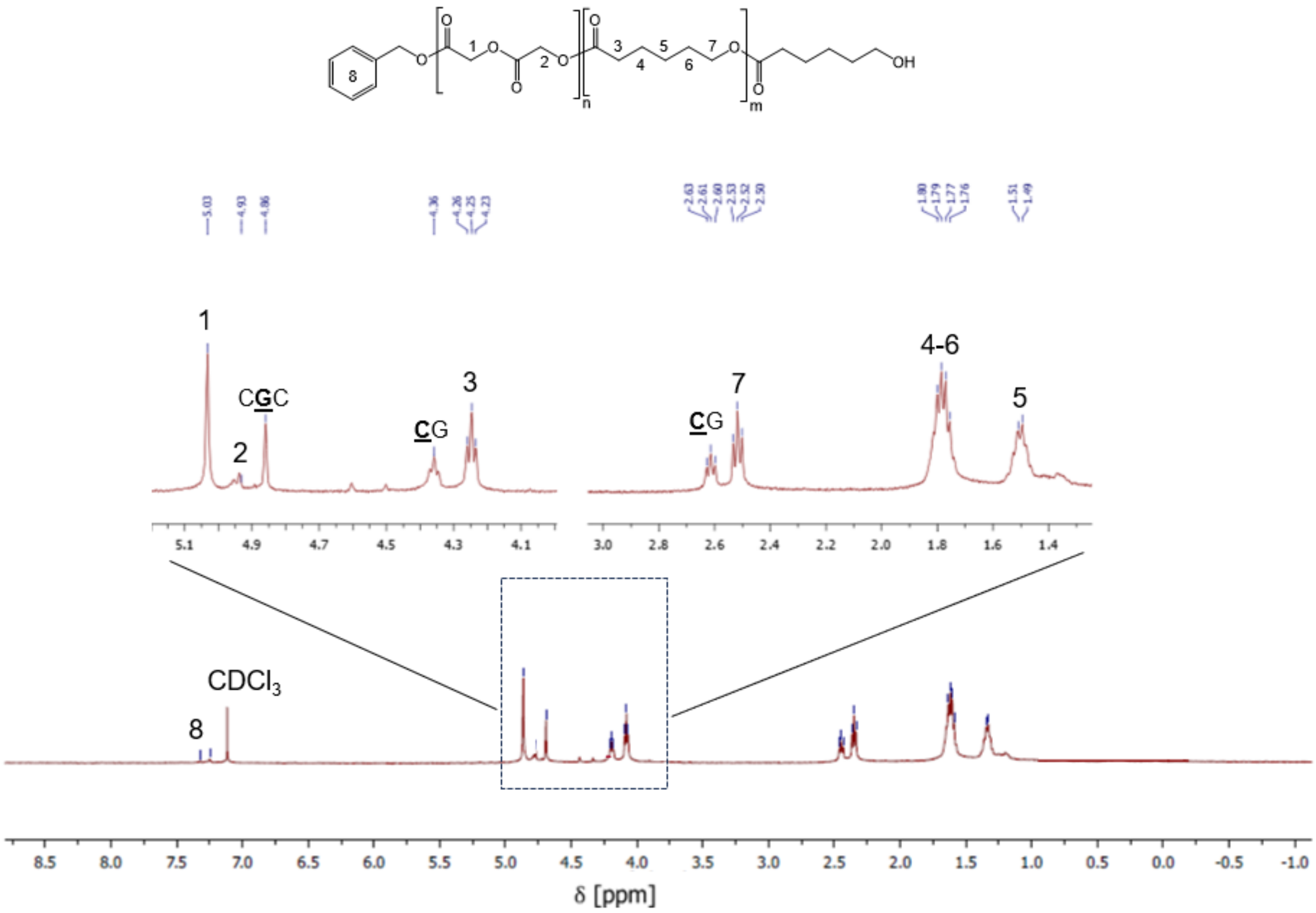

Figure S22. ^{1}H NMR spectrum (500 MHz, $CDCl_3$) of PGA_{20}^{*} with 20 eq. of CL (Table 1, entry 3).

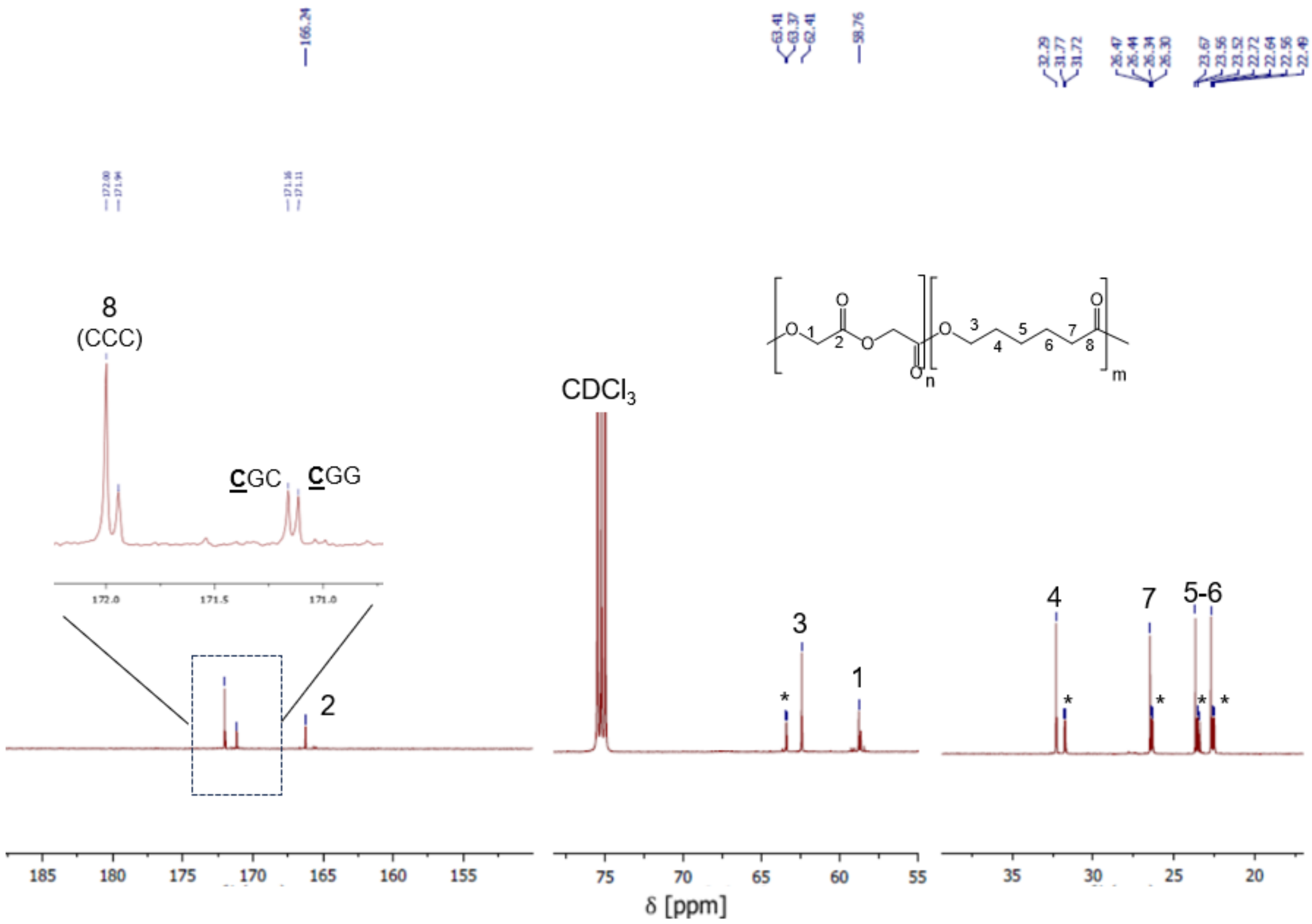

Figure S23. Quantitative ^{13}C NMR spectrum (500 MHz, $CDCl_3$) of PGA_{20}^{*} with 20 eq. of CL (indicates the resonance CLGCL, of a glycolyl unit between CL units) (Table 1, entry 3).*

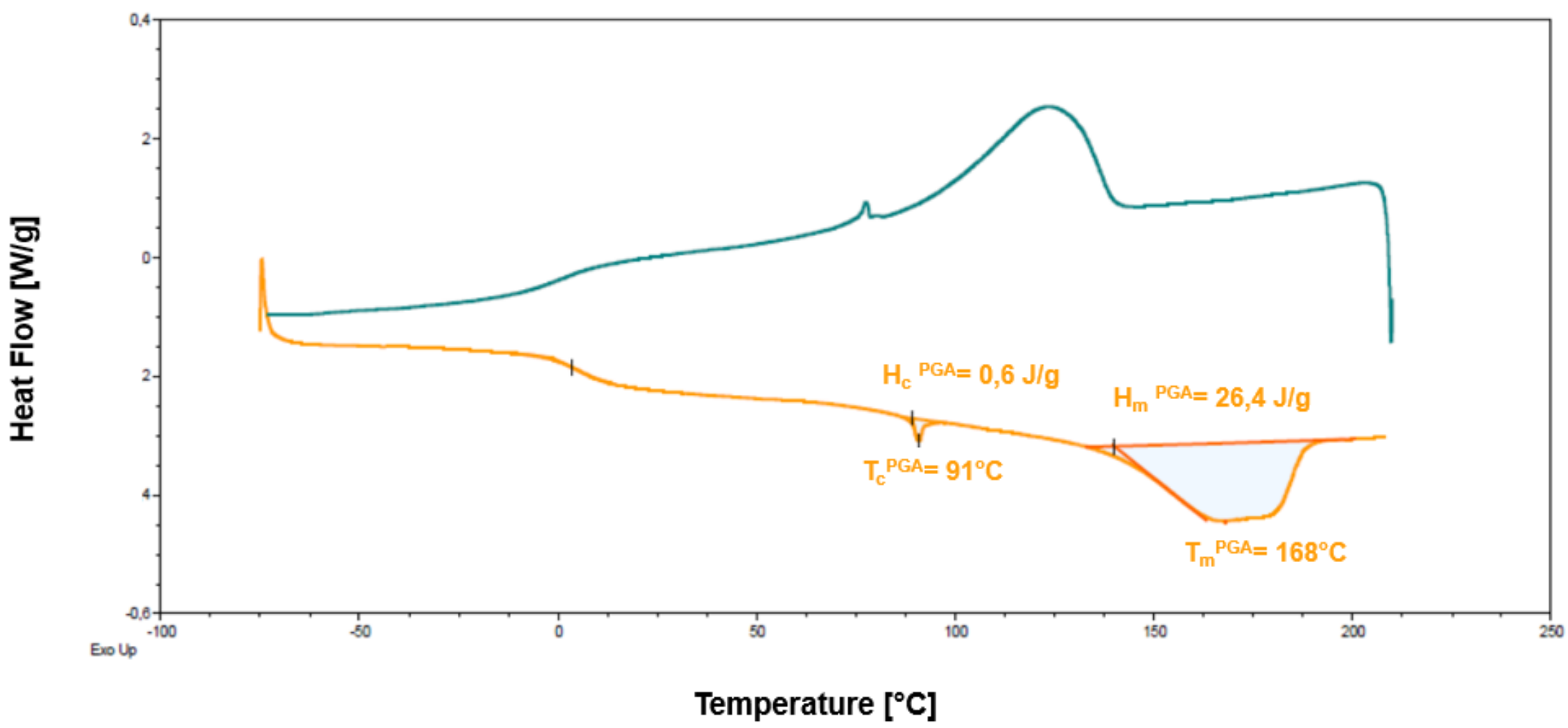

Figure S24. DSC curve (first cooling, second heating) of PGA_{20}^{*} with 20 eq. of CL.

2.2.1.3. Simultaneous addition

Under an argon atmosphere, CL (125 mg, 1.1 mmol) and GA (30 mg, 0.27 mmol) were combined and stirred at 85 °C for 30 minutes in a vial in the glovebox. In a separate vial, **LZn_2Et** (11 mg, 13.9 µmol) was dissolved in dry toluene (0.1 mL) and BnOH (1.5 mg, 1.4 µL, 13.9 µmol) from a stock solution (0.5 µM in toluene) was added. The solution was stirred

separately for 30 min and then added to the mixture of GA and CL. Aliquots were taken at suitable times until the reaction was quenched with $CDCl_3$. The final product was obtained as a white solid in cooled acidified methanol.

Simultaneous addition of GA and CL (Table 1, entry 5) gave copolymers with average glycolide sequence lengths (l_{GA}) as low as 0.5. As each GA monomer contains two glycolyl ester units (GG), this value indicates that there is, on average, only one glycolyl ester unit between two caprolactone units (*i.e.* a CGC triad). The corresponding *R* values are ≥ 1.2, which can occur when one of the monomer units can itself be transesterified (*i.e.* from a glycolidyl GG unit to a glycolyl G unit).

Table S2. Additional timepoints for Table 1, entry 4: simultaneous addition of CL (80 eq.) and GA (20 eq.).

Entry	Time (min)	Conv. (%) CL	Conv. (%) GA	l_{GA} [b]	l_{CL} [b]	R [c]
1	2	99	8	-	-	-
2	7	99	15	1	2	1.2
3	20	99	30	0.6	3	1.3
4	40	99	35	0.5	4	1.3

[a] $M_n^{theo} = (M^{M1} \times ([M_1]_0/[BnOH]) \times C_{M1}\%) + (M^{M2} \times ([M_2]_0/[BnOH]) \times C_{M2}\%)$.
[b] Average sequence of glycolidyl blocks and of caproyl blocks, calculated using ^{1}H NMR spectroscopy.
[c] Calculated using ^{1}H NMR spectroscopy.

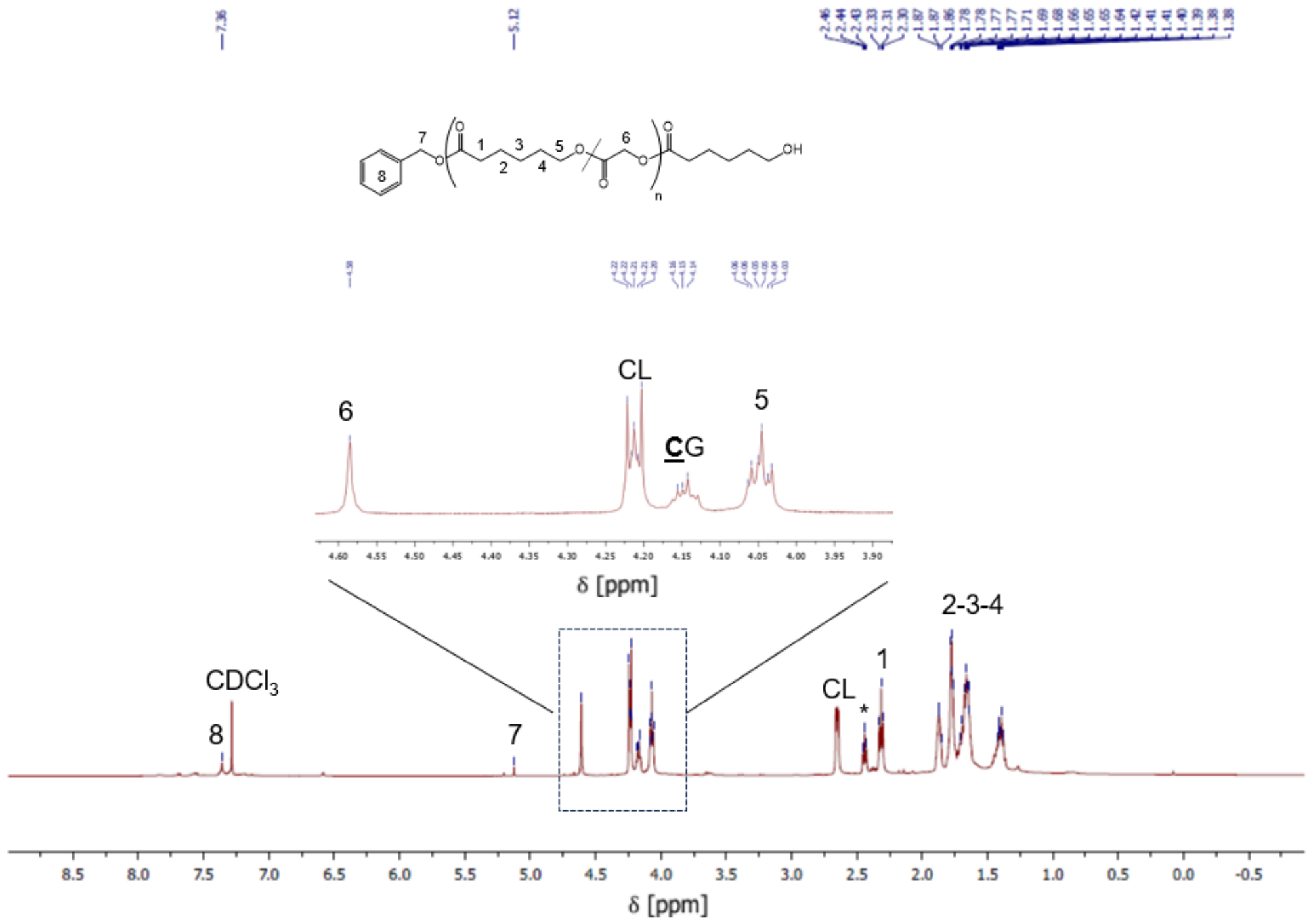

Figure S25. Example ^{1}H NMR spectrum (500 MHz, $CDCl_3$) of PGA_{20}-PCL_{80} with simultaneous monomer addition (indicates the resonance CLGCL, of a glycolyl unit between CL units) (Table 1, entry 4).*

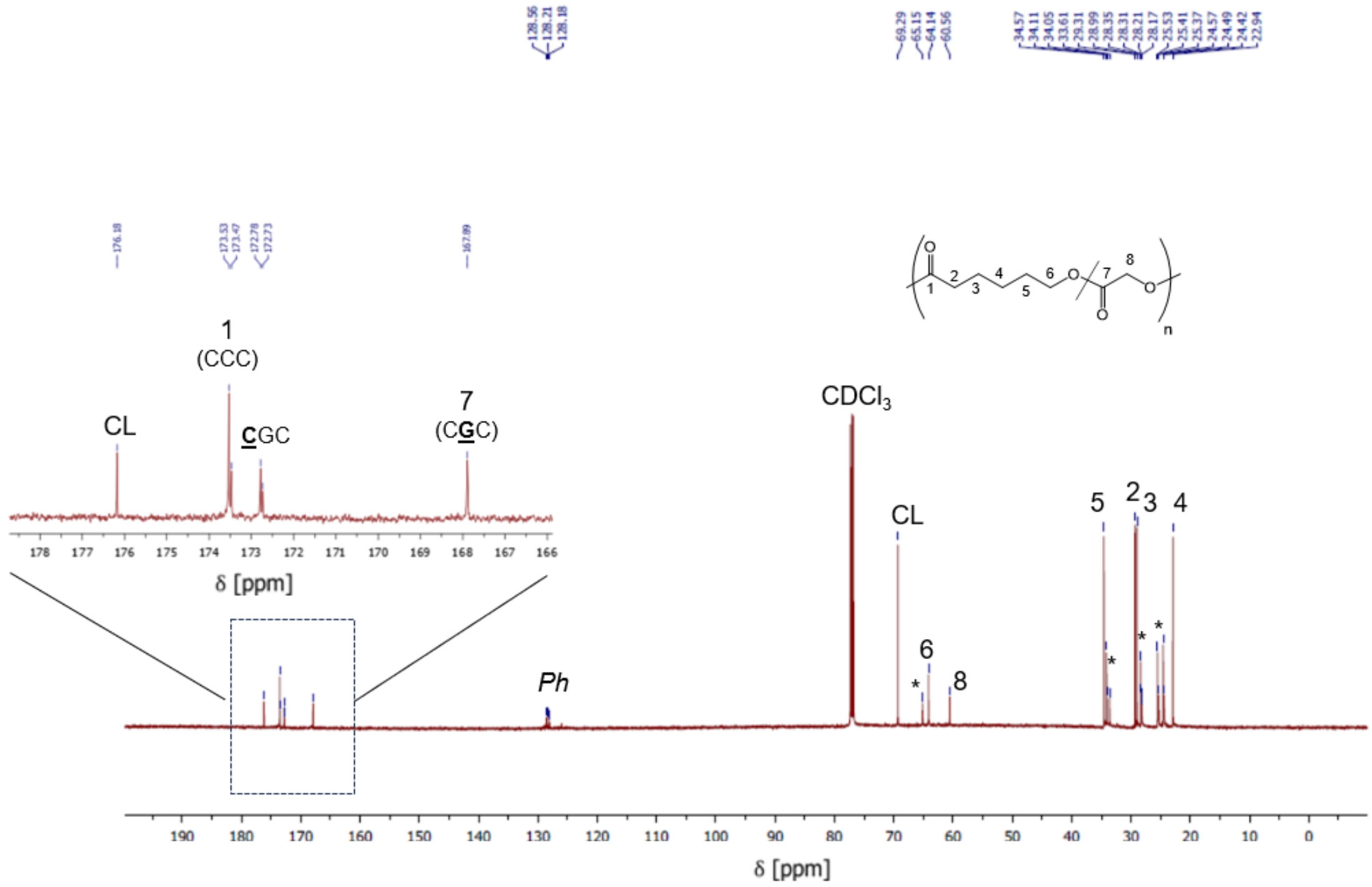

Figure S26. Example of quantitative ^{13}C NMR spectrum (126 MHz, $CDCl_3$) of PGA_{20}-PCL_{80} with simultaneous monomer addition (indicates the heterojunctions) (Table 1, entry 4).*

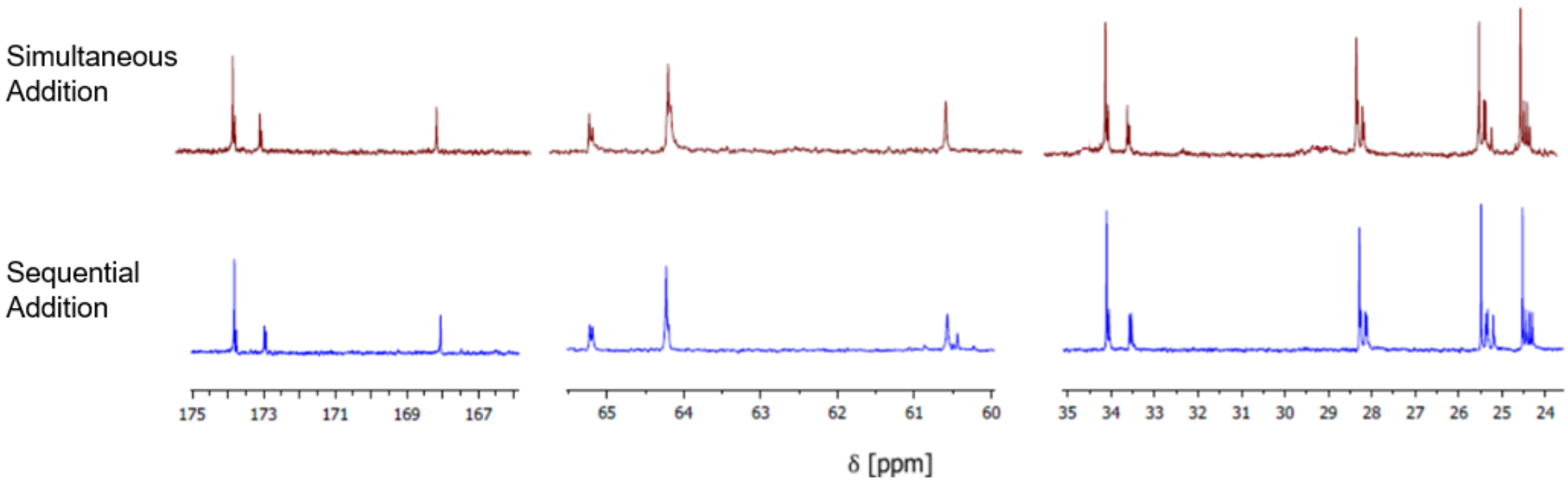

Figure S27. Comparison of quantitative ^{13}C NMR spectrum (126 MHz, $CDCl_3$) of PGA-PCL copolymers, obtained through simultaneous (top) and sequential addition through PGA first (bottom).*

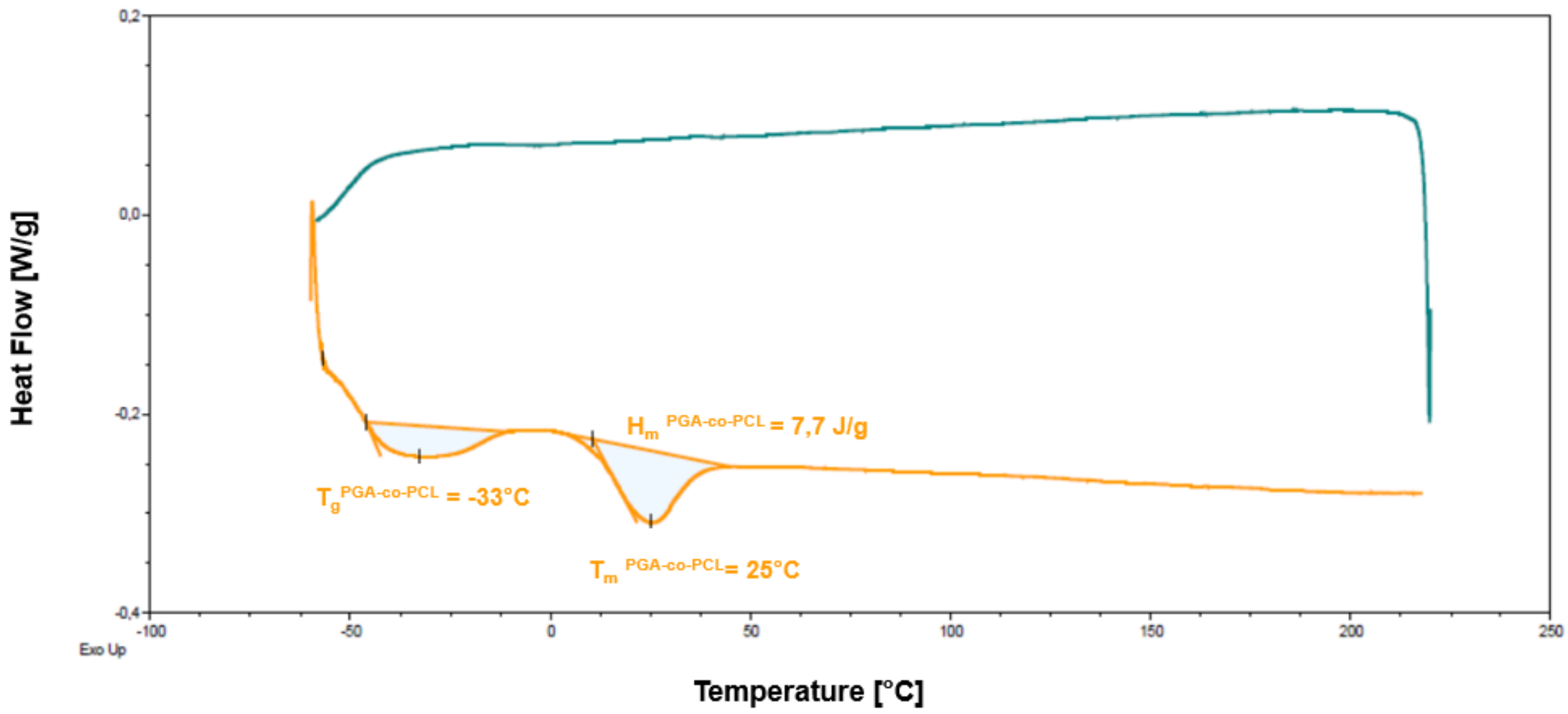

Figure S28. DSC curve (first cooling, second heating) of PGA_{20}-PCL_{80} (40 minute reaction) (Table 1, entry 4).

2.2.2. PCL-PGA triblocks

Under an argon atmosphere, CL was dissolved in dry toluene (0.2 mL) in a vial in the glovebox. In a separate vial, **LZn_2Et** (11 mg, 13.9 µmol) was dissolved in dry toluene (0.39 mL) and BnOH (1.5 mg, 1.4 µL, 13.9 µmol) from a stock solution (0.5 µM in toluene) was added. The two solutions were stirred separately for 30 min at 70 °C and then combined by adding CL to the catalyst solution. An aliquot was taken and the reaction mixture was added to a toluene solution of GA (30 mg, 0.27 mmol) of 2 mL, previously stirred at 85 °C. Neat CL was subsequently added to the reaction mixture. The product was obtained as a white powder by precipitation in cold, acidified methanol.

2.2.3. PCL-PLA diblocks

Under an argon atmosphere in a glovebox, CL (125 mg, 1.1 mmol) was dissolved in dry toluene (1 mL). In a separate vial, **LZn_2Et** (11 mg, 13.9 µmol) was dissolved in dry toluene (0.39 mL) and BnOH (1.5 mg, 1.4 µL, 13.9 µmol) from a stock solution (0.5 µM in toluene) was added. The two solutions were stirred separately for 30 min at 70 °C and then combined by adding CL to the catalyst solution. An aliquot was taken and a toluene solution of *rac*-LA (40 mg, 0.28 mmol) in 1 mL, previously stirred at 70°C; was added to the stirring solution. After total conversion of *rac*-LA was achieved, neat CL was added to the reaction mixture. Aliquots were taken at suitable times until the reaction was quenched with $CDCl_3$. The final product was obtained as a white solid in cooled acidified methanol.

2.2.4. PCL-PLA triblocks

Triblock PCL-PGA and PCL-PLA copolymers: Following the above protocol, either 10 or 80 equivalents of neat CL were added to living PCL_{80}-*block*-PGA_{20}* and PCL_{80}-*block*-PLA_{20}* copolymers. Aliquots were taken at suitable times until the reaction was quenched with $CDCl_3$. The final product was obtained as a white solid in cooled acidified methanol.

The PCL-PGA and PCL-PLA copolymer structures were analysed by ^1H NMR. The randomness value and number average sequence lengths of the PCL-PGA were calculated according to Equations S1-S12 . For the PCL-PLA copolymers, the relative intergrals (*i*) of the of the diad resonances were used to calculate randomness (*R*) and the number average sequence lengths for M_1 and M_2 (l_{PM1} and l_{PM2}) using the following equations.[3,4]

^{1}H NMR spectroscopy can be used to quantify the PCL-PLA copolymer structure produced after transesterification has taken place. The relative integrals (i) of the diad resonances were used to calculate randomness (R) and the number average sequence lengths for LA and CL (l_e^{LA} and l_e^{CL}) using the following equations:[3]

$$i_{\mathrm{LA}} = i_{\mathrm{L-L}} + i_{\mathrm{L-C}} \tag{S11}$$

$$i_{\mathrm{CL}} = i_{\mathrm{C-C}} + i_{\mathrm{C-L}} \tag{S12}$$

$$f_{\mathrm{LA}} = \frac{i_{\mathrm{LA}}}{i_{\mathrm{LA}}+i_{\mathrm{CL}}} \tag{S13}$$

$$f_{\mathrm{CL}} = \frac{i_{\mathrm{CL}}}{i_{\mathrm{LA}}+i_{\mathrm{CL}}} \tag{S14}$$

Where f_{LA} and f_{CL} are relative mole fractions of PLA and PCL in the copolymer, respectively. Following this the average dyad relative molar fractions $f_{\mathrm{C\text{-}C}}$, $f_{\mathrm{C\text{-}L}}$, and $f_{\mathrm{L\text{-}L}}$, were calculated:

$$f_{\mathrm{C-C}} = f_{\mathrm{CL}} \times f_{\mathrm{C-C/CL}} \tag{S15}$$

$$f_{\mathrm{C-L}} = f_{\mathrm{CL}} \times f_{\mathrm{C-L/CL}} \tag{S16}$$

$$f_{\mathrm{L-L}} = 1 - (f_{\mathrm{C-C}} + f_{\mathrm{C-L}}) \tag{S17}$$

Where,

$$f_{C-C/CL} = \frac{i_{\mathrm{C-C}}}{i_{\mathrm{CL}}} \tag{S18}$$

$$f_{\mathrm{C-L/CL}} = \left(\frac{i_{\mathrm{C-L}}}{i_{\mathrm{CL}}}\right) \times 2 \tag{S19}$$

Finally, l_e^{LA}, l_e^{CL} and R can be calculated using the following equations:

$$l_e^{LA} = \frac{2f_{\mathrm{LA}}}{f_{\mathrm{C-L}}} \tag{S20}$$

$$l_e^{CL} = \frac{2f_{\mathrm{CL}}}{f_{\mathrm{C-L}}} \tag{S21}$$

$$R = \frac{f_{\mathrm{C-L}}}{2\times f_{\mathrm{LA}}\times f_{\mathrm{CL}}} \tag{S22}$$

In both cases, the R value was significantly higher with the PCL-PLA copolymers than the PCL-PGA copolymers. For example, addition of 10 equivalents of CL gave a R value of 0.5 for PCL-PLA*, and a R value of 0.04 for PCL-PGA* (Table 2, entry 2 *vs* entry 1), indicating a greater extent of transesterification of the PCL-PLA* copolymers. With both PCL-PGA* and PCL-PLA* precursors, increasing the equivalents of CL gave a pronounced increase in the randomness of the polymer chain (Table 2, entry 1 *vs* 3 and entry 2 *vs* 4).

Table S3. Addition of CL to living poly(CL-block-GA) and poly(CL-block-LA*) copolymer precursors.*

Entry	M_1 (eq.)	M_2 (eq.)	M_3 (eq.)	Time (h)			l_{M2} [a]	l_{CL}	R [b]	M_2 [c] Int. (%)	CCC [c] Int. (%)
				M_1	M_2	M_3					
1	CL (80)	GA (20)	CL (10)	0.5	0.03	1	20	82	0.04	95	99
2	CL (80)	*rac*-LA (20)	CL (10)	0.5	0.5	24	2	12	0.5	81	86
3	CL (80)	GA (20)	CL (80)	0.5	0.03	24	1	15	0.6	43	93
4	CL (80)	*rac*-LA (20)	CL (80)	0.5	0.5	24	1	10	0.9	71	79

Full conversion of M (>99%) was observed in each instance.
[a] Average sequence of glycolidyl blocks (entries 1 and 3) and of lactide blocks (entries 2 and 4), calculated using ^{1}H NMR spectroscopy.
[b] Calculated using ^{1}H NMR spectroscopy.
[c] Relative intensities of the homotriads resonance, calculated using ^{1}H spectroscopy for entries 1 and 3 (GAGAGA), and ^{13}C NMR spectroscopy for entries 2 and 4 (LALALA).

^{1}H NMR spectroscopic analysis of the intensities of the triads in the PCL-PGA copolymer structures provided further mechanistic insights. After the addition of 10 equivalents of CL (entry 1), the relative intensities of the resonances corresponding to the homotriads, CCC (4.08 ppm) and GAGAGA (4.75 ppm), were very high (95% and 99%), aligning with a highly blocky character. In contrast, addition of 80 eq. of CL substantially decreased the intensity of the GGG triad from 95% to 43% (entry 3), while the intensity of the CCC triad underwent only a slight reduction to 93%. Quantitative ^{13}C NMR spectroscopy showed that under identical conditions but using *rac*-LA as the comonomer gave a more uniform decrease in both the CCC (173.5 ppm) and LALALA (169.5 ppm) resonances (79% and 71% respectively, entry 4). This indicates that with PCL-PGA copolymers, transesterification preferentially occurred in the PGA block leaving the PCL blocks mostly intact, whereas with the PCL-PLA copolymer, transesterification occurs in both the PCL and PLA domains. This difference in the preferred site of transesterification is attributed to the difference in structure between the ester bonds in the PGA and PLA chain backbones. In PLA, the methyl group both sterically and electronically stabilises the ester group, disfavouring nucleophilic attack compared to PGA, where the ester bonds are more susceptible to transesterification.

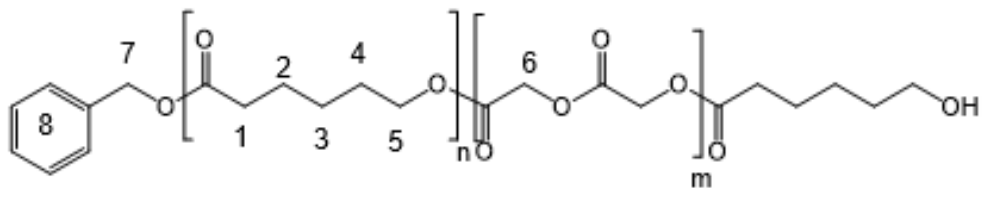

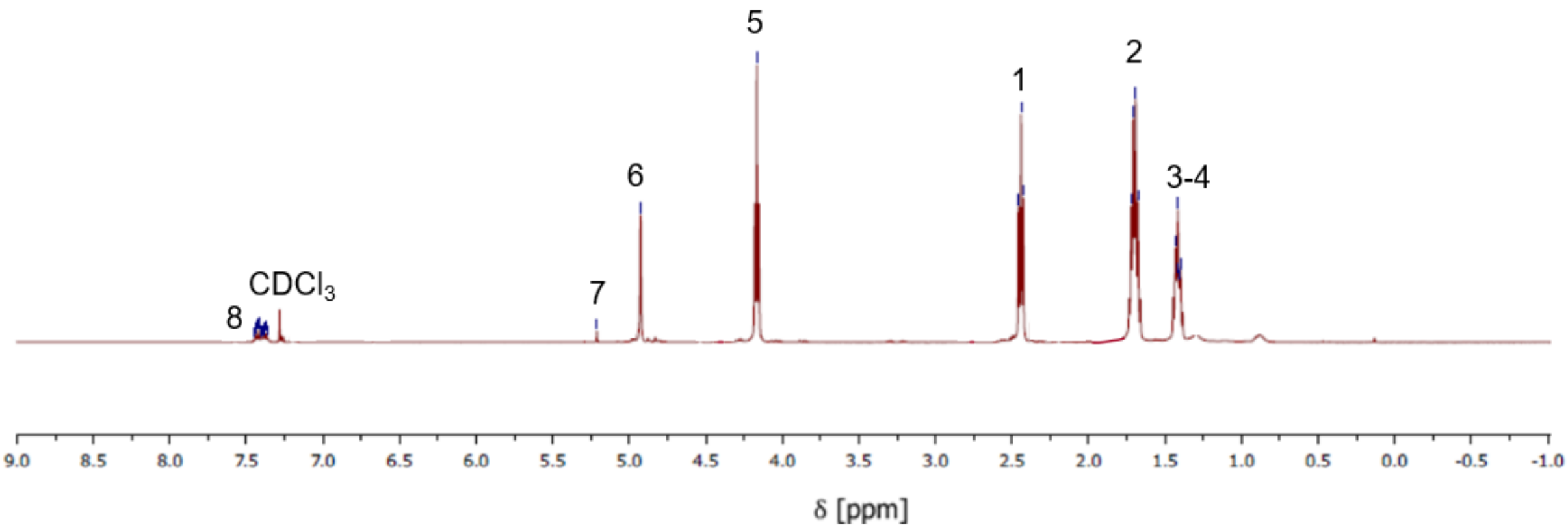

Figure S29. ^{1}H NMR spectrum (500 MHz, $CDCl_3$) of PCL_{80}-PGA_{20}-PCL_{10} (Table S3, entry 1).

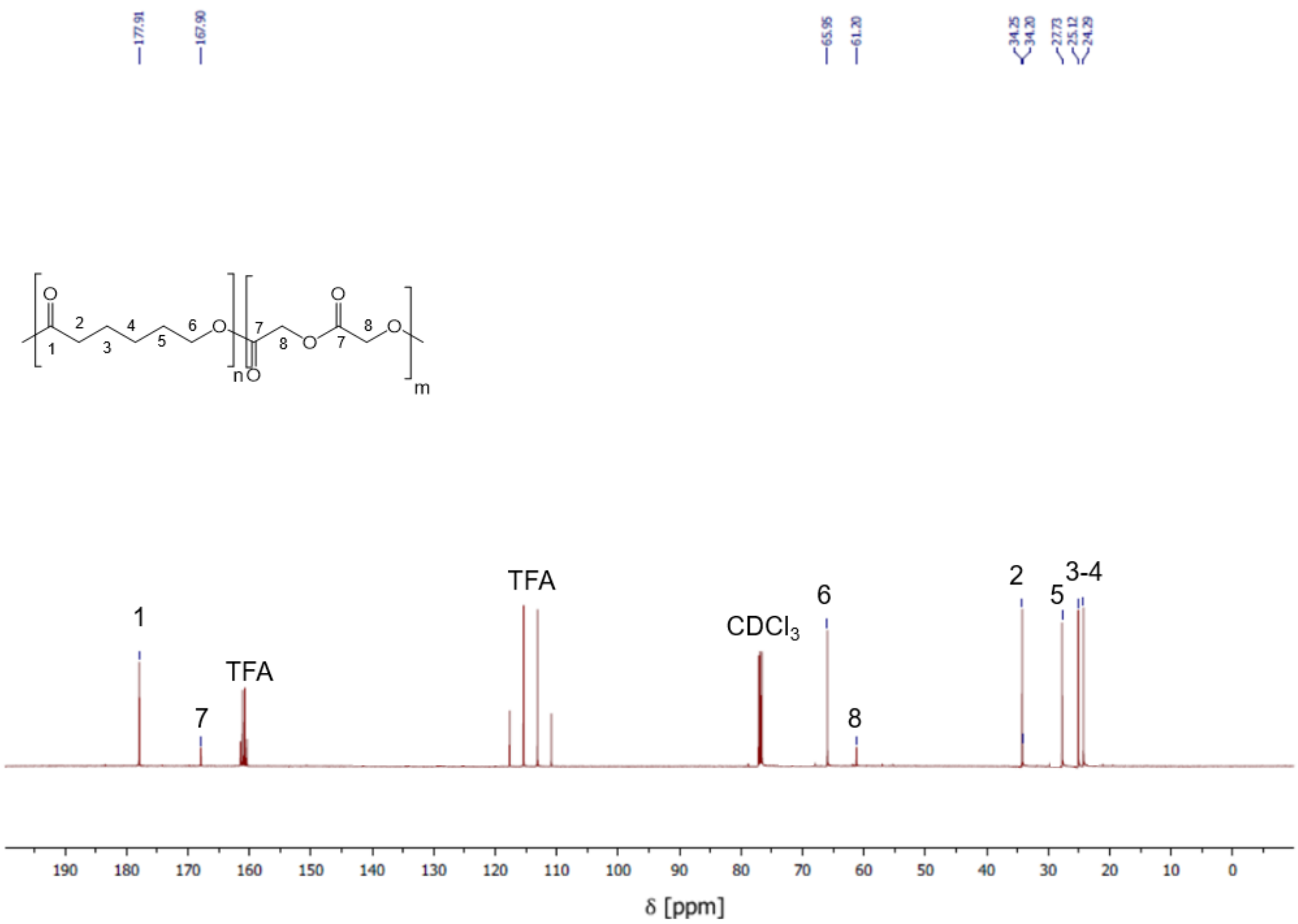

Figure S30. Quantitative ^{1}H NMR spectrum (126 MHz, $CDCl_3$ + TFA) of PCL_{80}-PGA_{20}-PCL_{10} (note: TFA was added to enhance the solubility of the resultant PCL_{20}-PGA_{20} copolymers) (Table S3, entry 1).

.

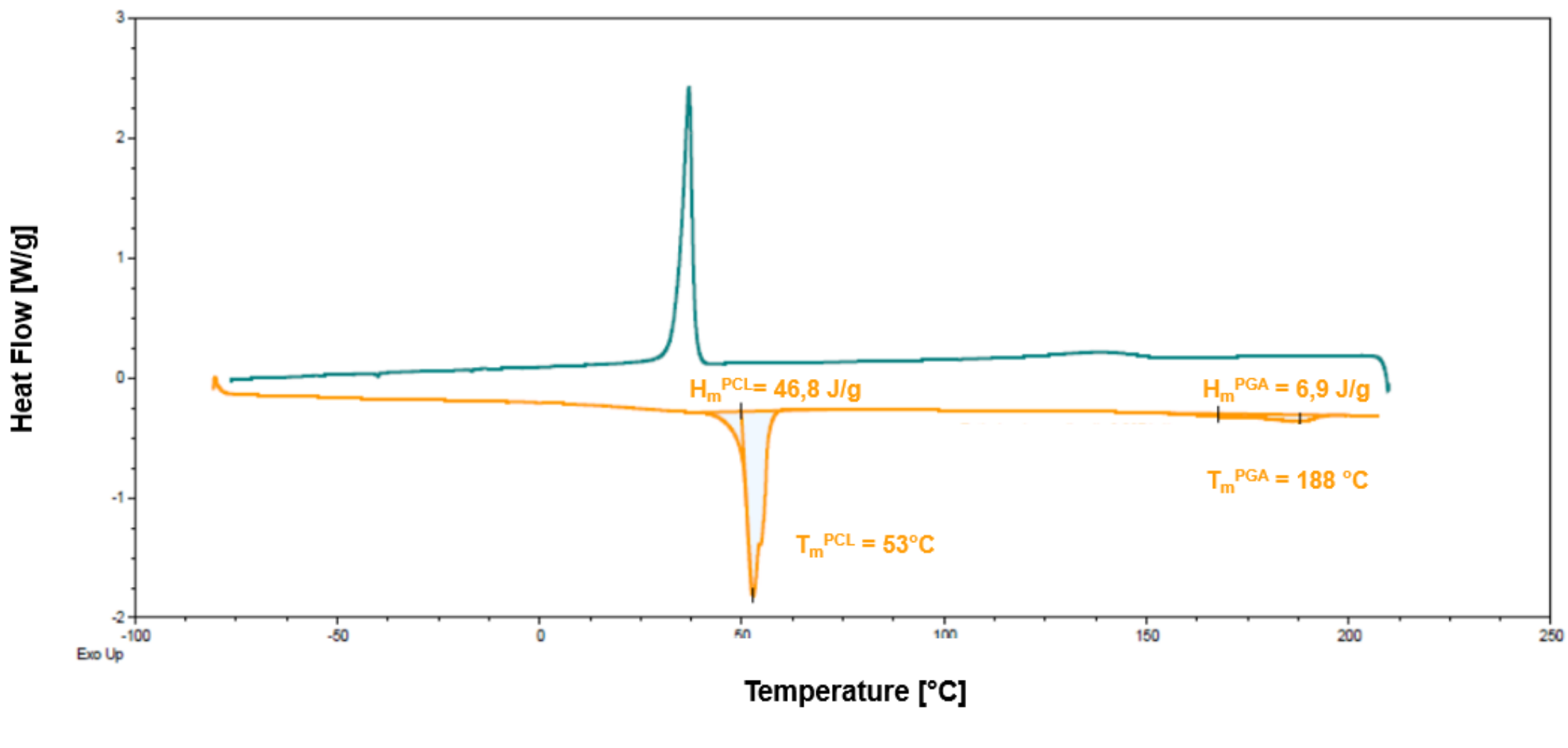

Figure S31. DSC curve of PCL_{80}-PGA_{20}-PCL_{10} (Table S3, entry 1).

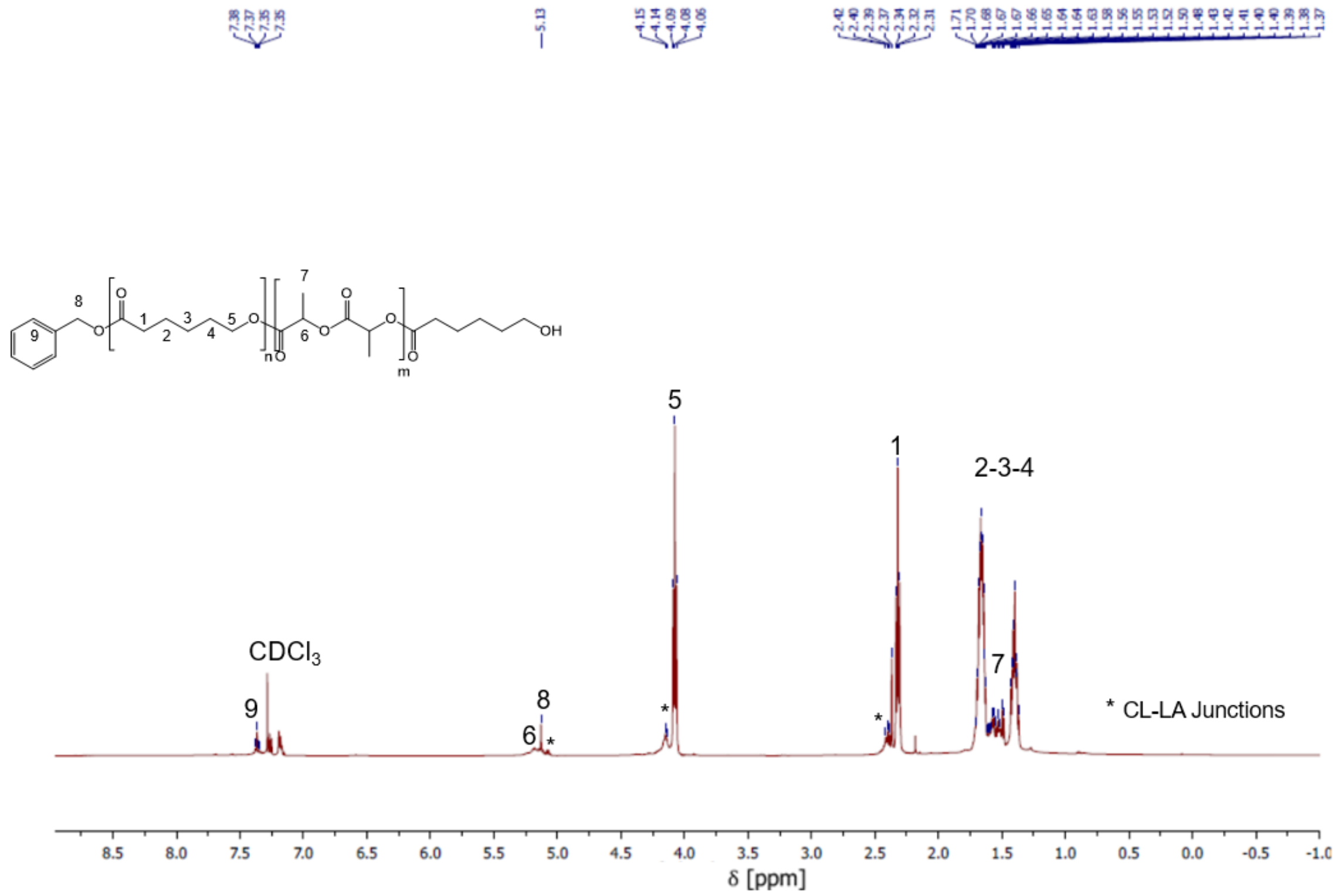

Figure S32. ^{1}H NMR spectrum (500 MHz, $CDCl_3$) of PCL_{80}-PLA_{20}-PCL_{10} (Table S3, entry 2).

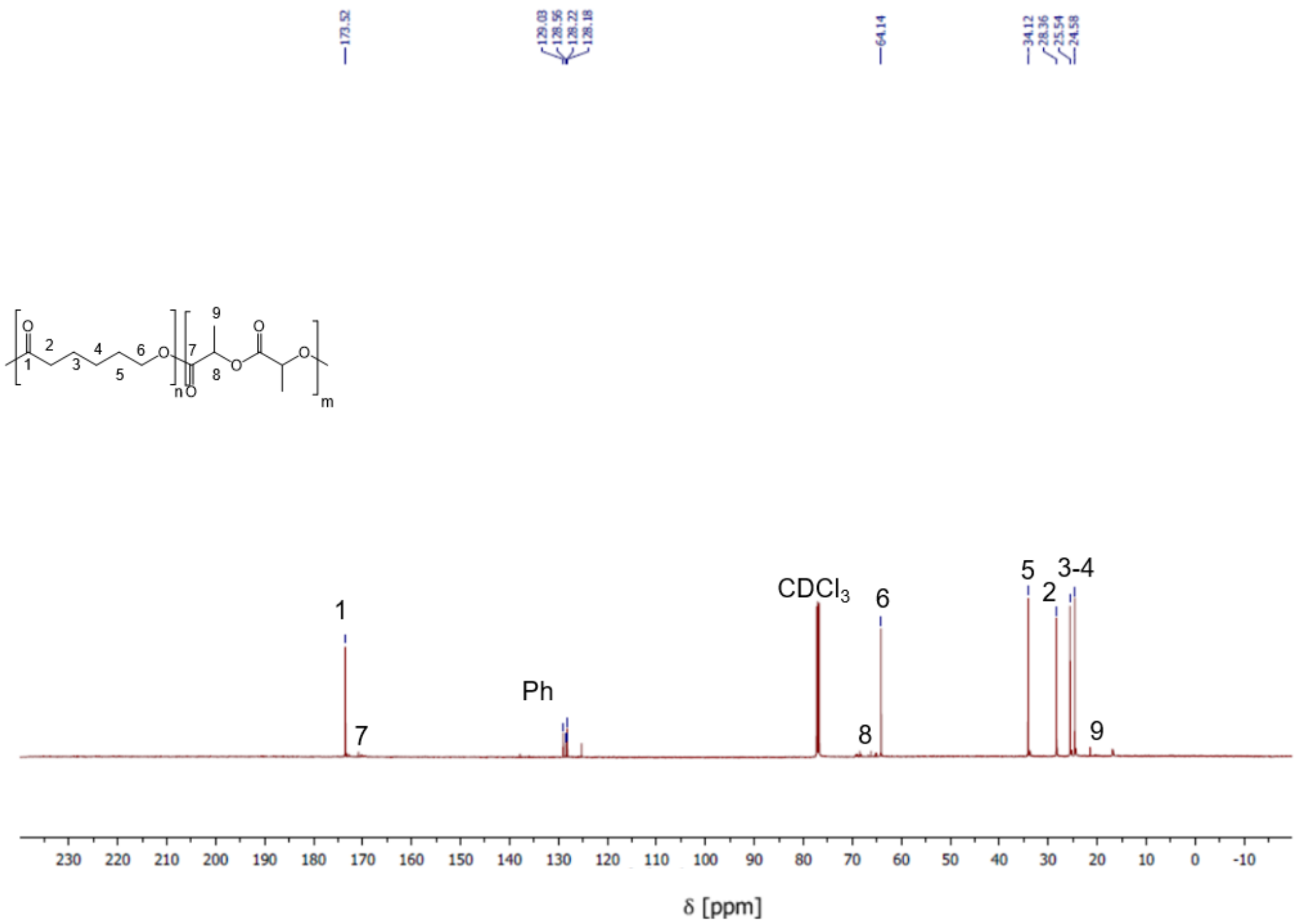

Figure S33. Quantitative ^{13}C NMR spectrum (126 MHz, $CDCl_3$) of PCL_{80}-PLA_{20}-PCL_{10} (Table S3, entry 2).

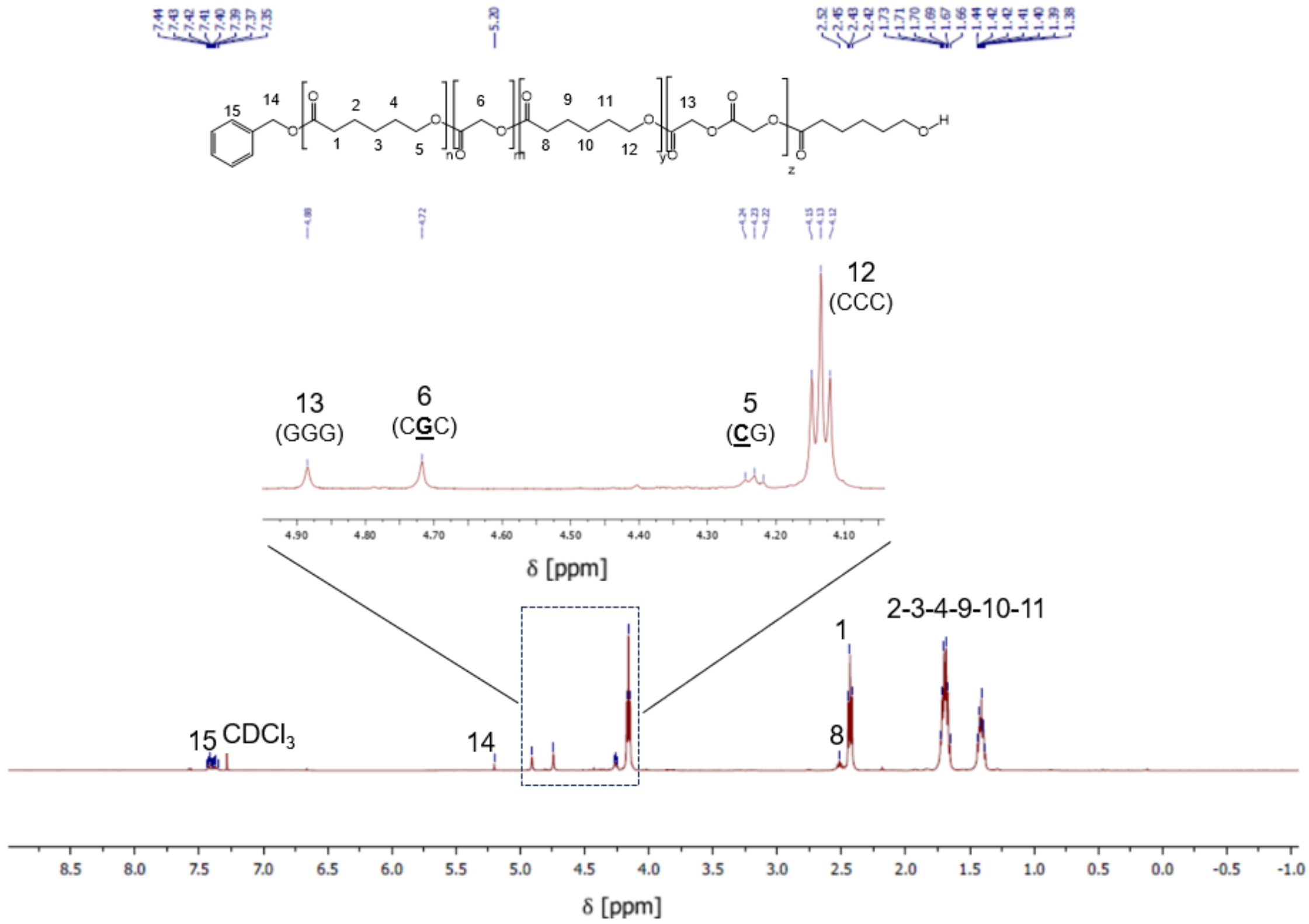

Figure S34. ¹H NMR spectrum (500 MHz, CDCl₃) of PCL₈₀-PGA₂₀-PCL₈₀ (Table S3, entry 3).

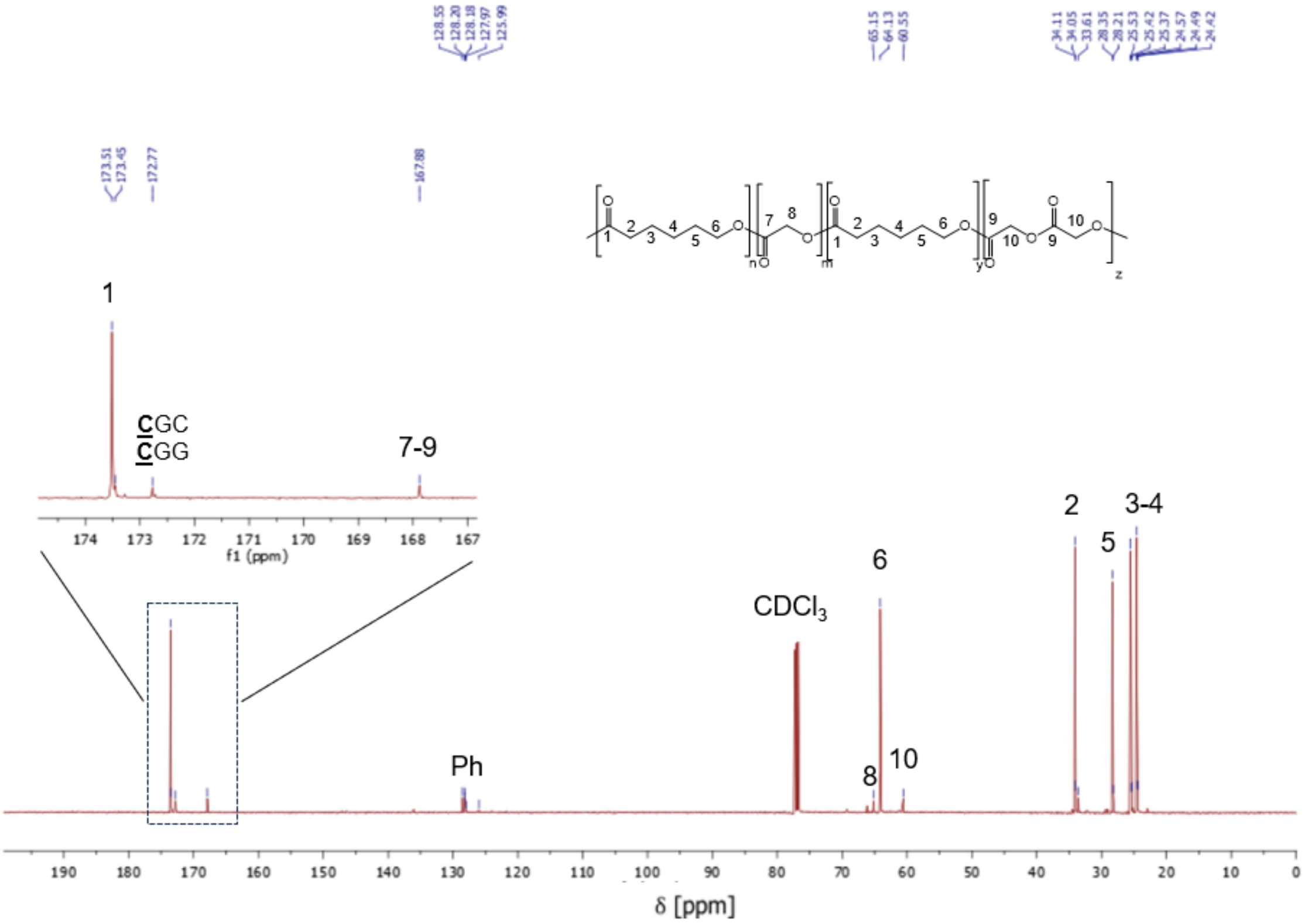

Figure S35. Quantitative ¹H NMR spectrum (126 MHz, CDCl₃) of PCL₈₀-PGA₂₀-PCL₈₀ (Table S3, entry 3).

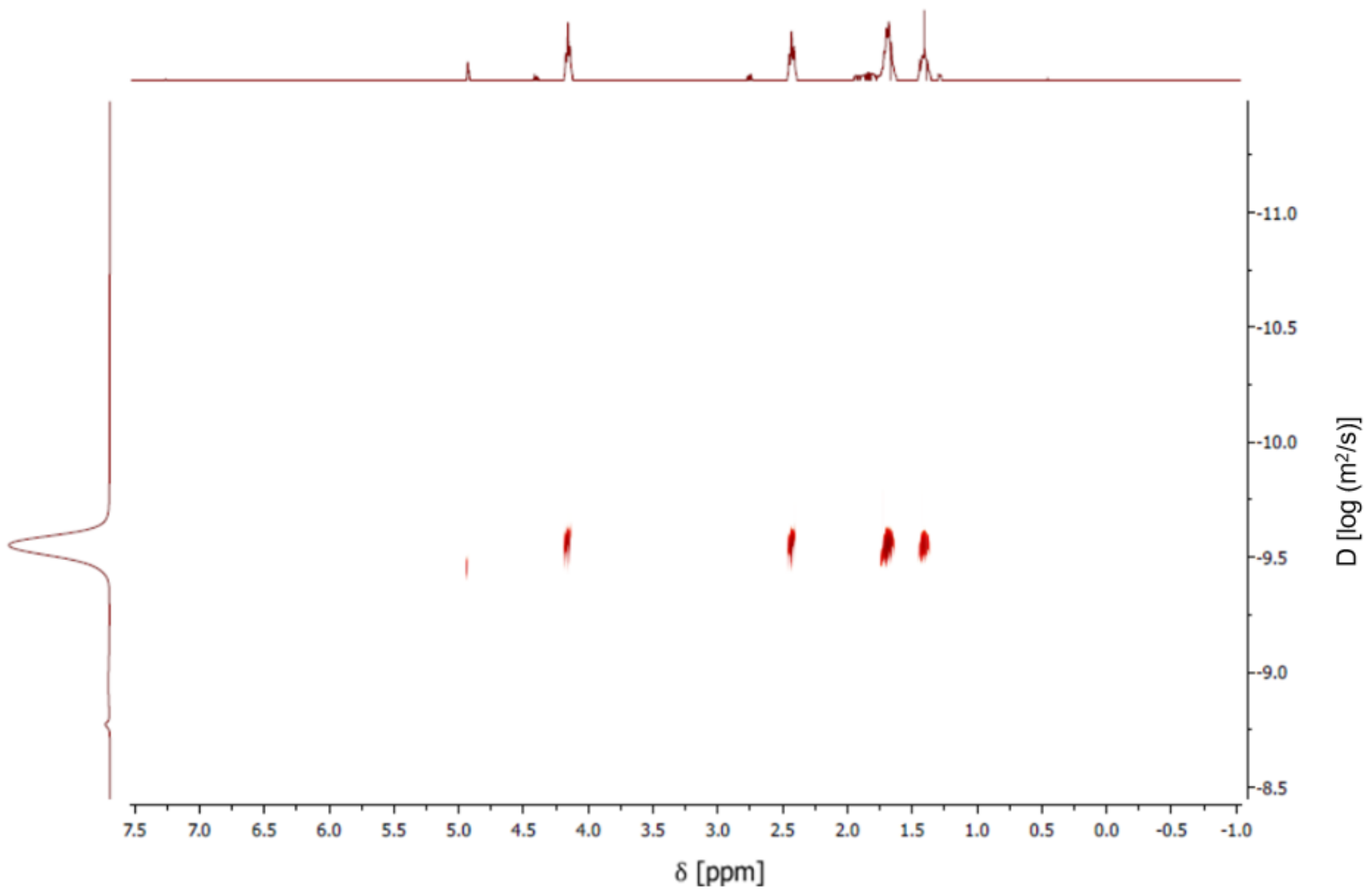

Figure S36. ¹H DOSY NMR spectrum (500 MHz, $CDCl_3$) of PCL_{80}-PGA_{20}-PCL_{80} (Table S3, entry 3).

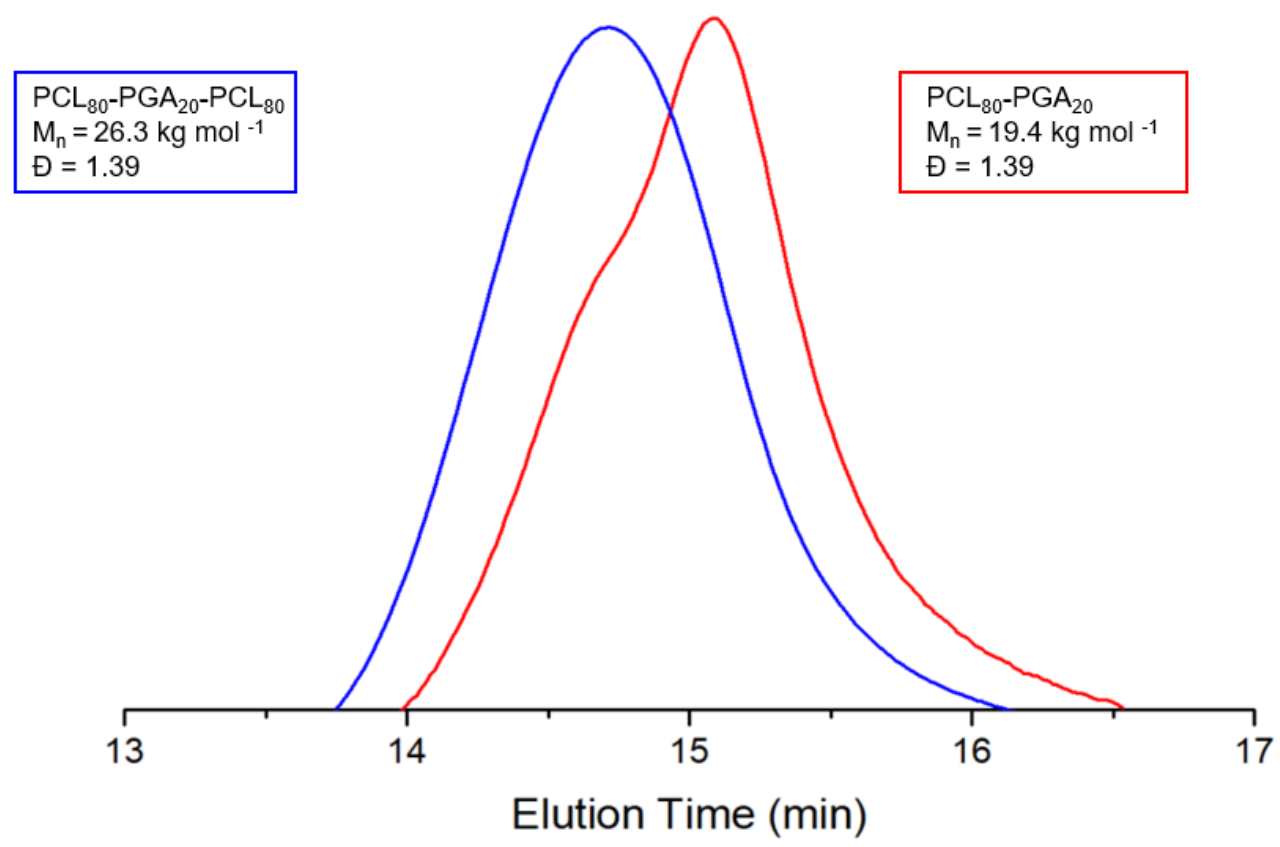

Figure S37. SEC traces of aliquots taken during the PCL_{80}-PGA_{20}-PCL_{80} copolymerisation (Table S3, entry 3).

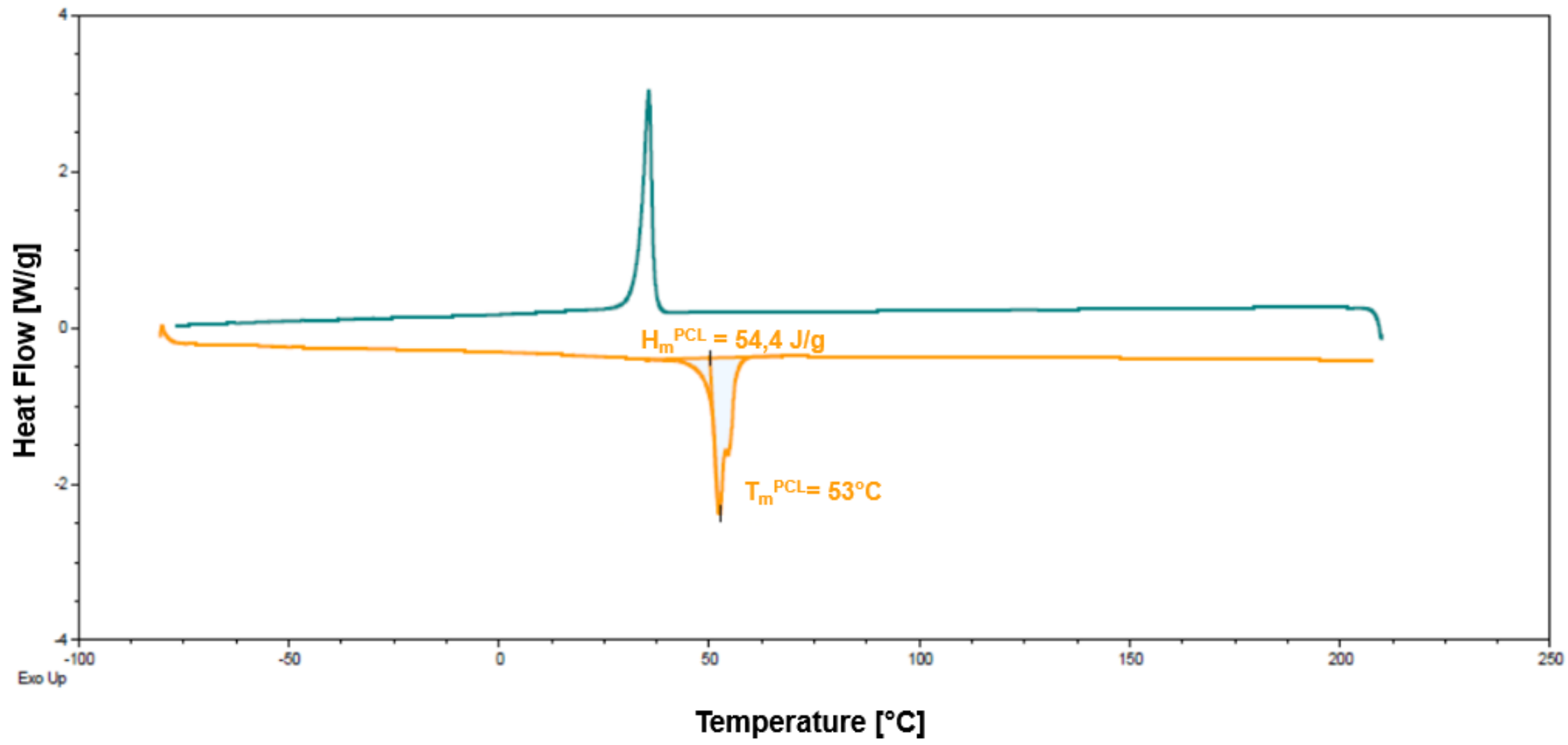

Figure S38. DSC curve of PCL_{80}-PGA_{20}-PCL_{80} (Table S3, entry 3).

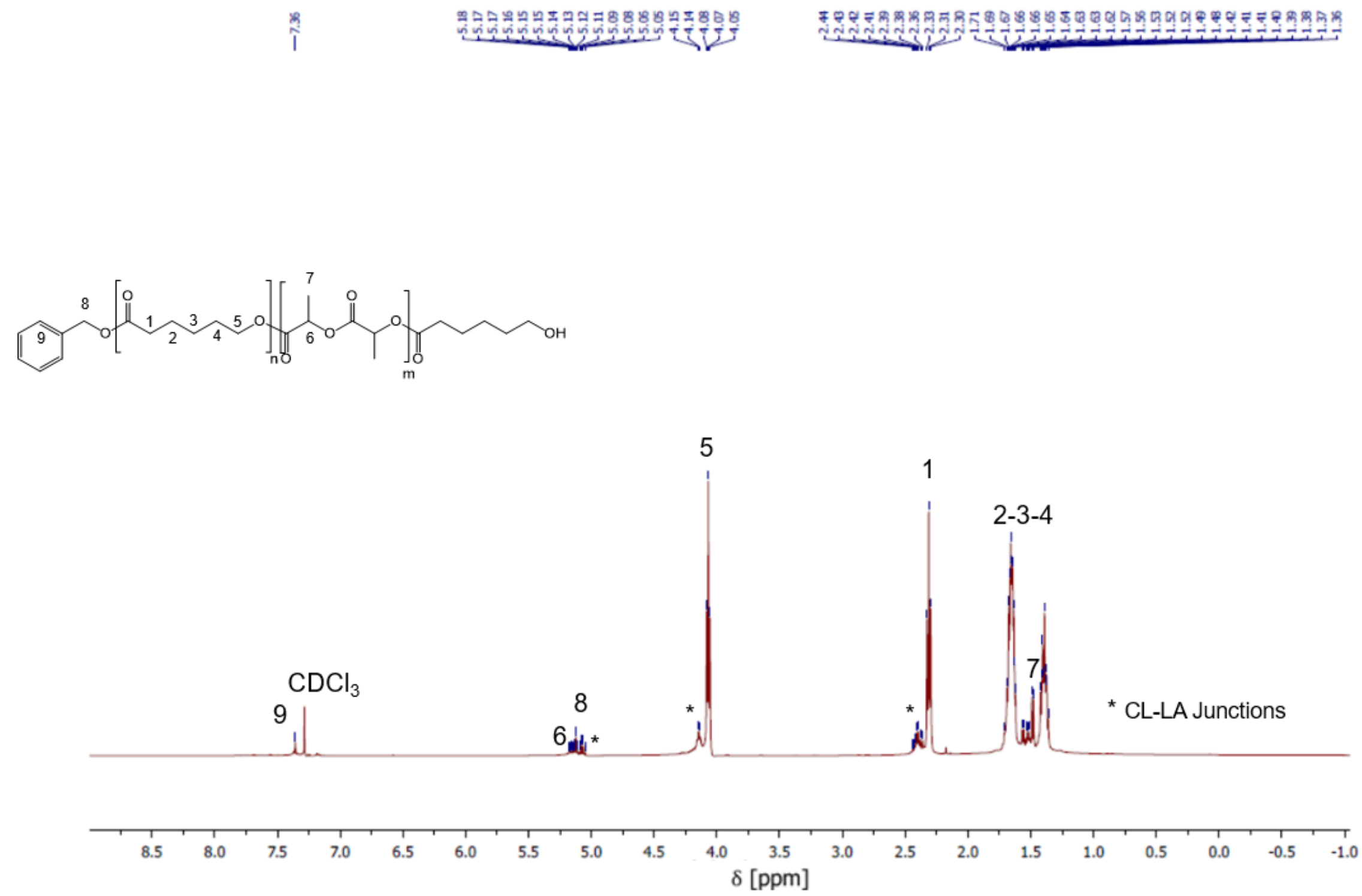

Figure S39. ^{1}H NMR spectrum (500 MHz, $CDCl_3$) of PCL_{80}-PLA_{20}-PCL_{80} (Table S3, entry 4).

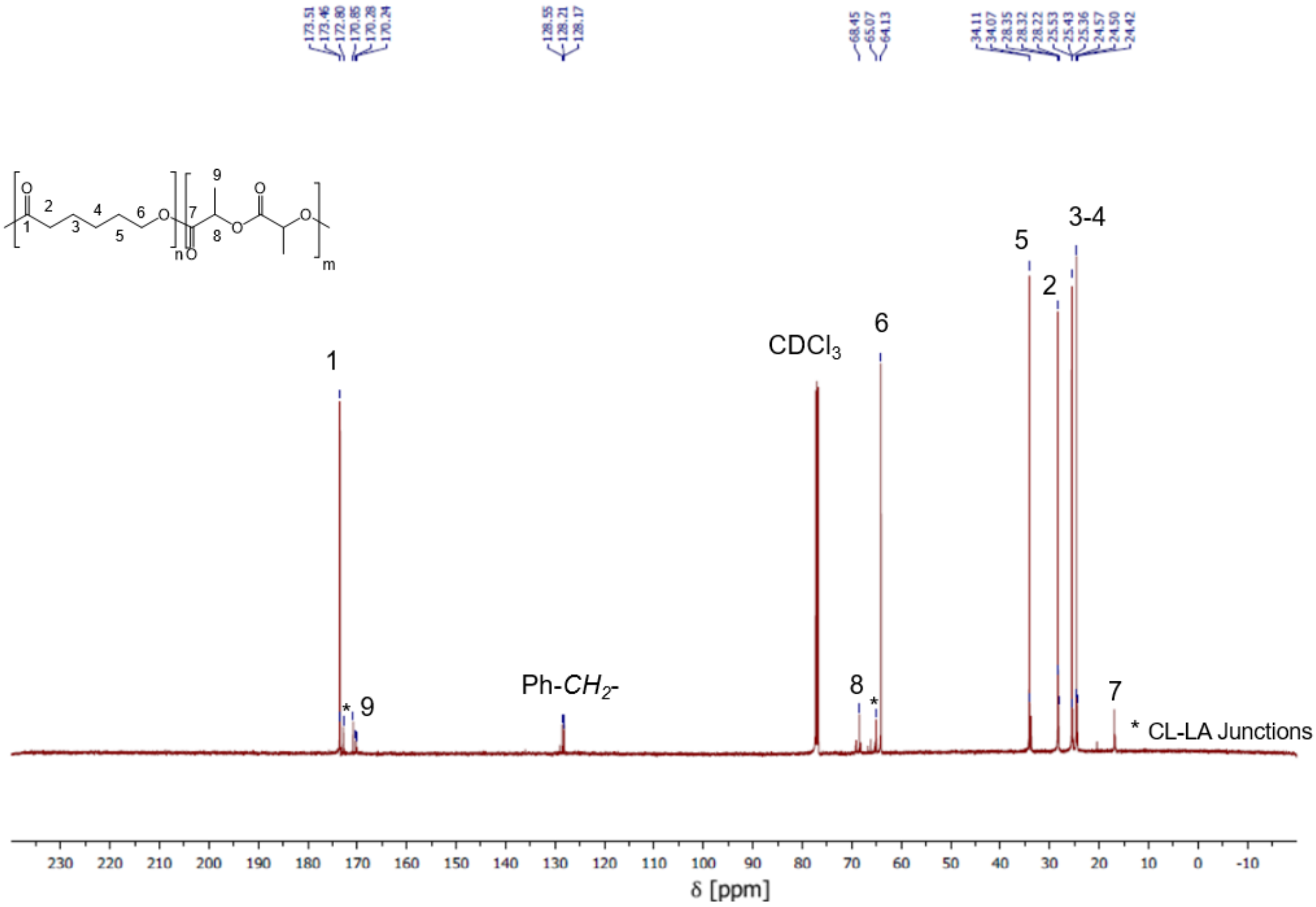

Figure S40. Quantitative ^{13}C NMR spectrum (126 MHz, $CDCl_3$) of PCL_{80}-PLA_{20}-PCL_{80} (Table S3, entry 4).

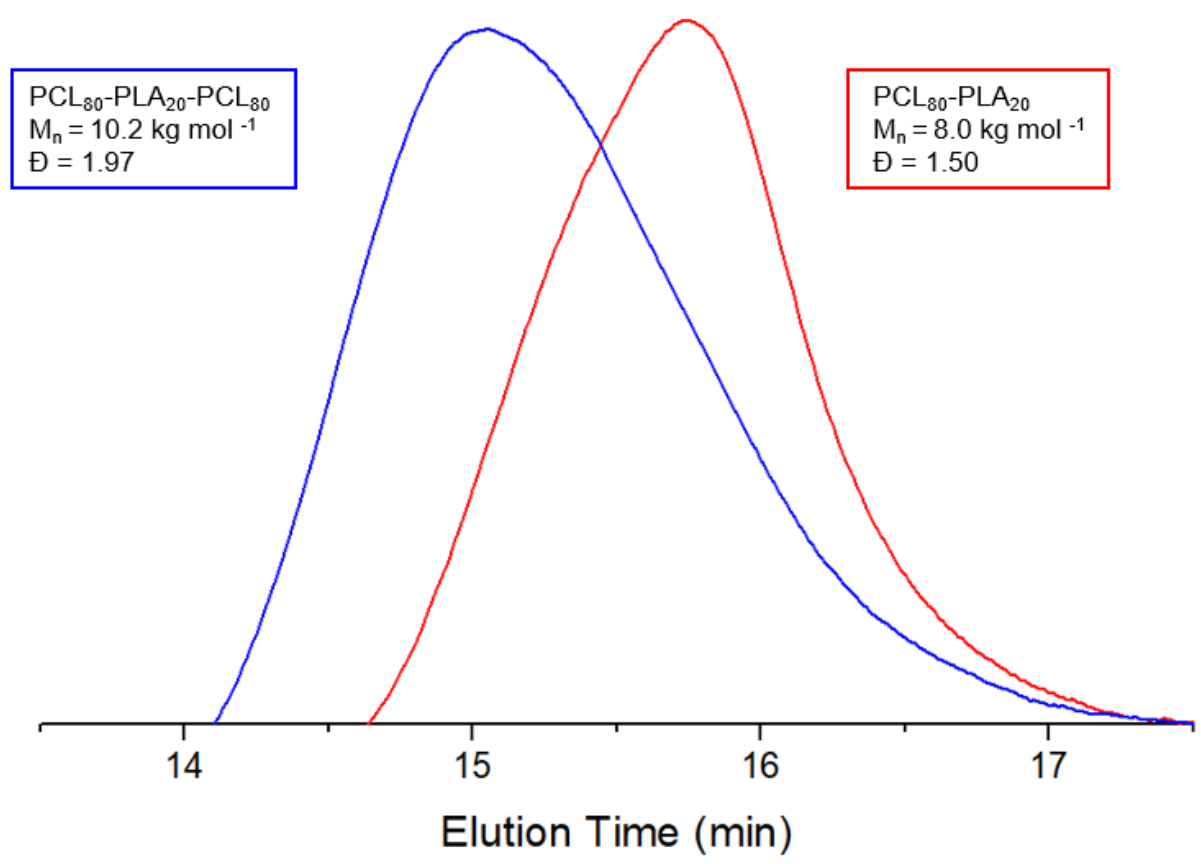

Figure S41. SEC traces of aliquots taken during the PCL_{80}-PLA_{20}-PCL_{80} copolymerisation (Table S3, entry 4).

2.2.5. PDL-PCL diblocks

2.2.5.1. Sequential addition: CL first

Under an argon atmosphere in glovebox, CL (157.2 mg, 152.6 µL, 1.39 mmol) was dissolved in dry toluene (1 mL). In a separate vial, **LZn_2Et** (11 mg, 13.9 µmol) was dissolved in dry toluene (0.39 mL) and BnOH (1.5 mg, 1.4 µL, 13.9 µmol) from a stock solution (0.5 µM in toluene) was added. The two solutions were stirred separately for 30 min at 70 °C and then combined by adding CL to the catalyst solution. After 10 minutes, an aliquot was taken and DL (226.2 mg, 231.7 µL, 1.33 mmol) was added to the stirring solution. Further aliquots were

taken at appropriate times to monitor the reaction until termination by $CDCl_3$ after 22.3 h. The product was obtained as a white powder by precipitation in cold, acidified methanol.

^{1}H NMR (500 MHz, $CDCl_3$) δ 4.87-4.82 (quint, 1H·n, -C*H*(C_4H_9)OCO-), 4.07-4.04 (t, 2H·n, -$COC_4H_8CH_2O$-), 2.31-2.28 (t, 2H·n, -$COCH_2C_3H_6CH_2O$-), 2.28-2.25 (t, 2H·m, -$COCH_2C_3H_6$CBuHO-) , 1.68-1.59 (m, 4H·n + 2H·m, -$CH_2CH_2CH_2$-) , 1.55-1.48 (m, 4H·m, -$CH_2CHCH_2C_3H_7$), 1.41-1.36 (m, 2H·n, -$CH_2CH_2CH_2$-), 1.32-1.23 (m, 6H·m, -$CH_2CH_2CH_2$-), 0.89-0.86 (t, 3H·m, -CH_3).

^{13}C NMR (126 MHz, $CDCl_3$) δ 173.7, 173.4, 74.0, 64.3, 34.6, 34.3, 34, 33.9, 28.5, 27.6, 25.7, 25.1, 25.1, 24.7, 22.7, 14.1.

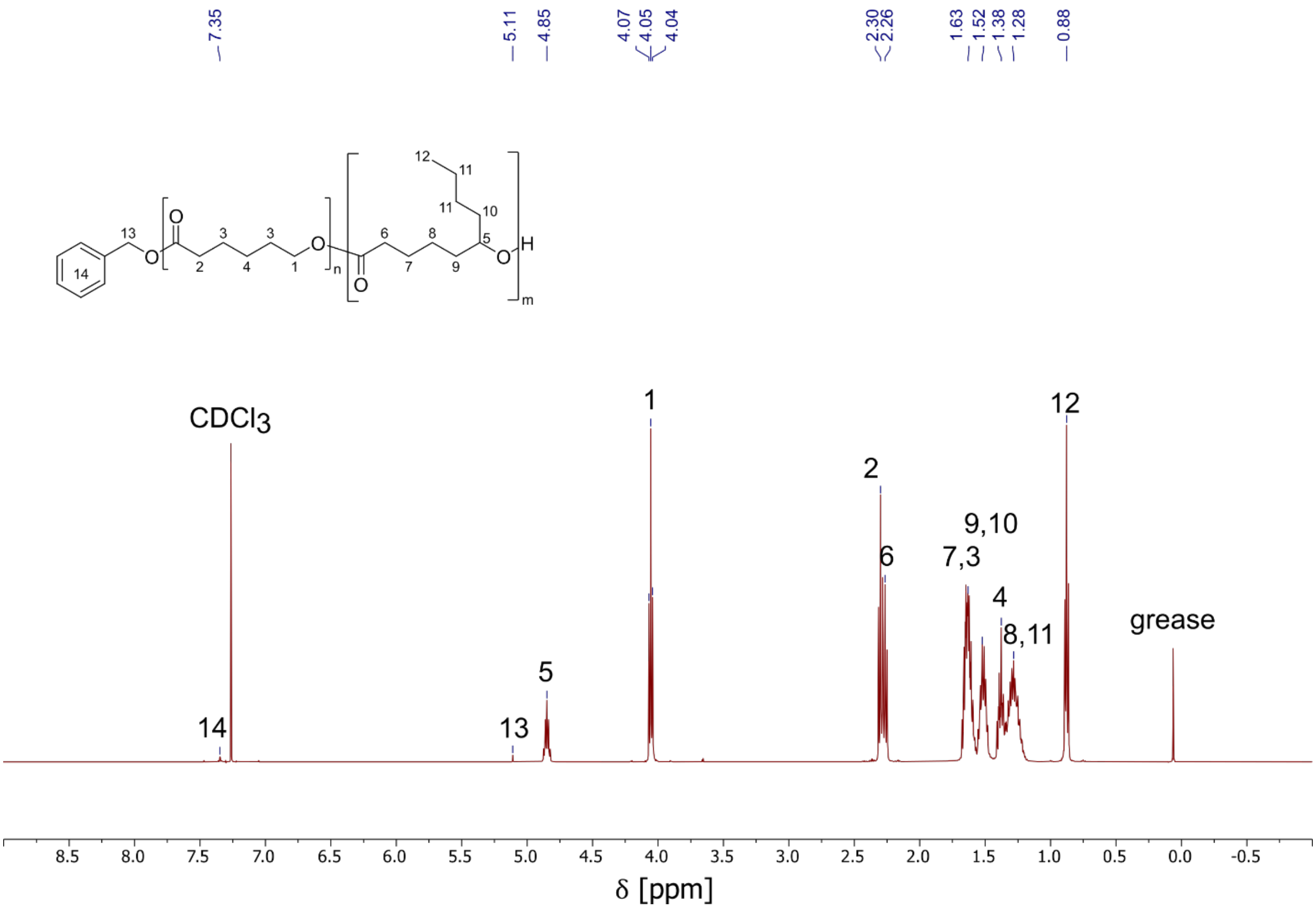

Figure S42. ^{1}H NMR spectrum (500 MHz, $CDCl_3$) of PCL-PDL (Table 1, entry 7).

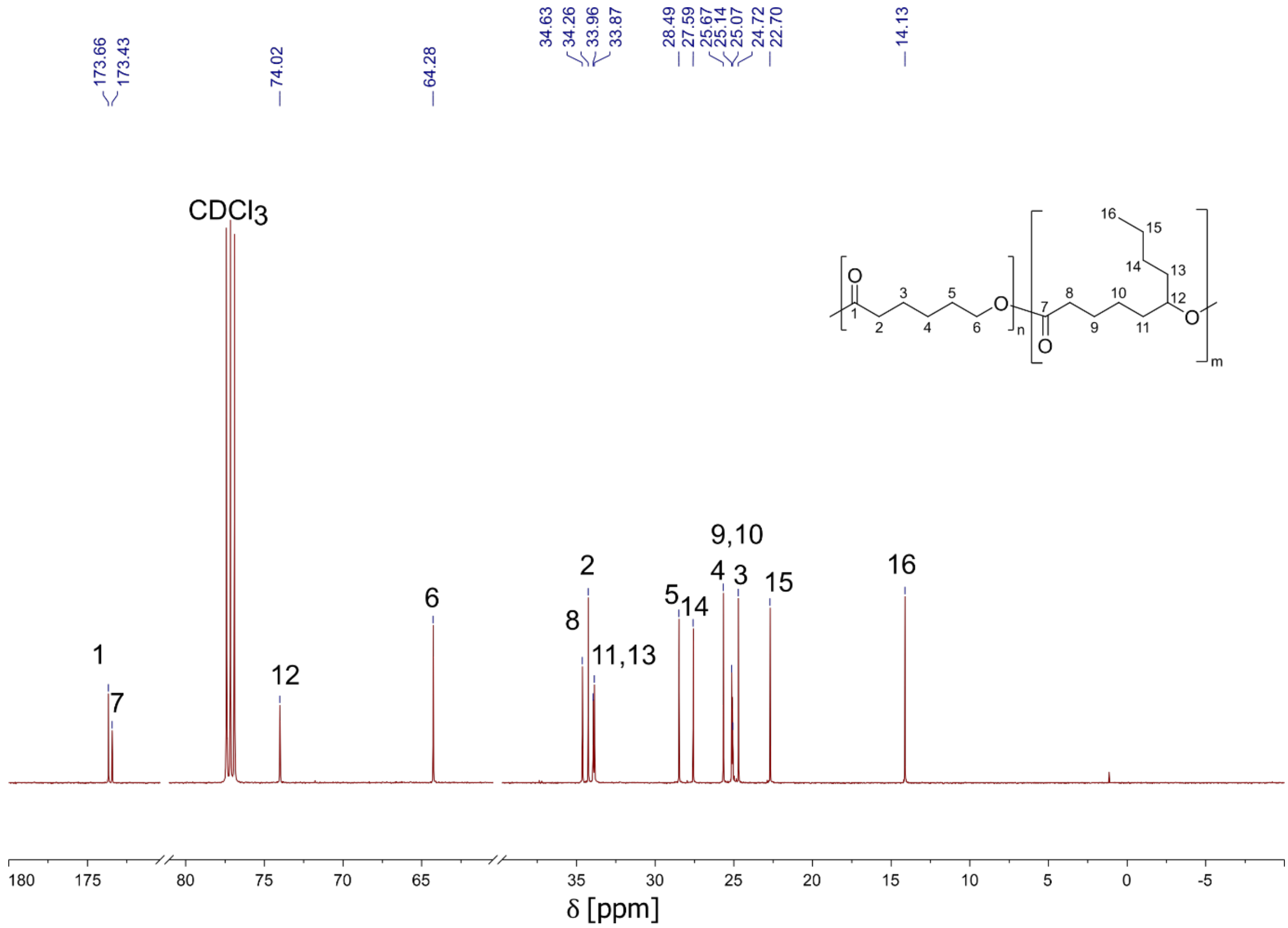

Figure S43. ^{13}C NMR spectrum (126 MHz, $CDCl_3$) of PCL-PDL (Table 1, entry 7).

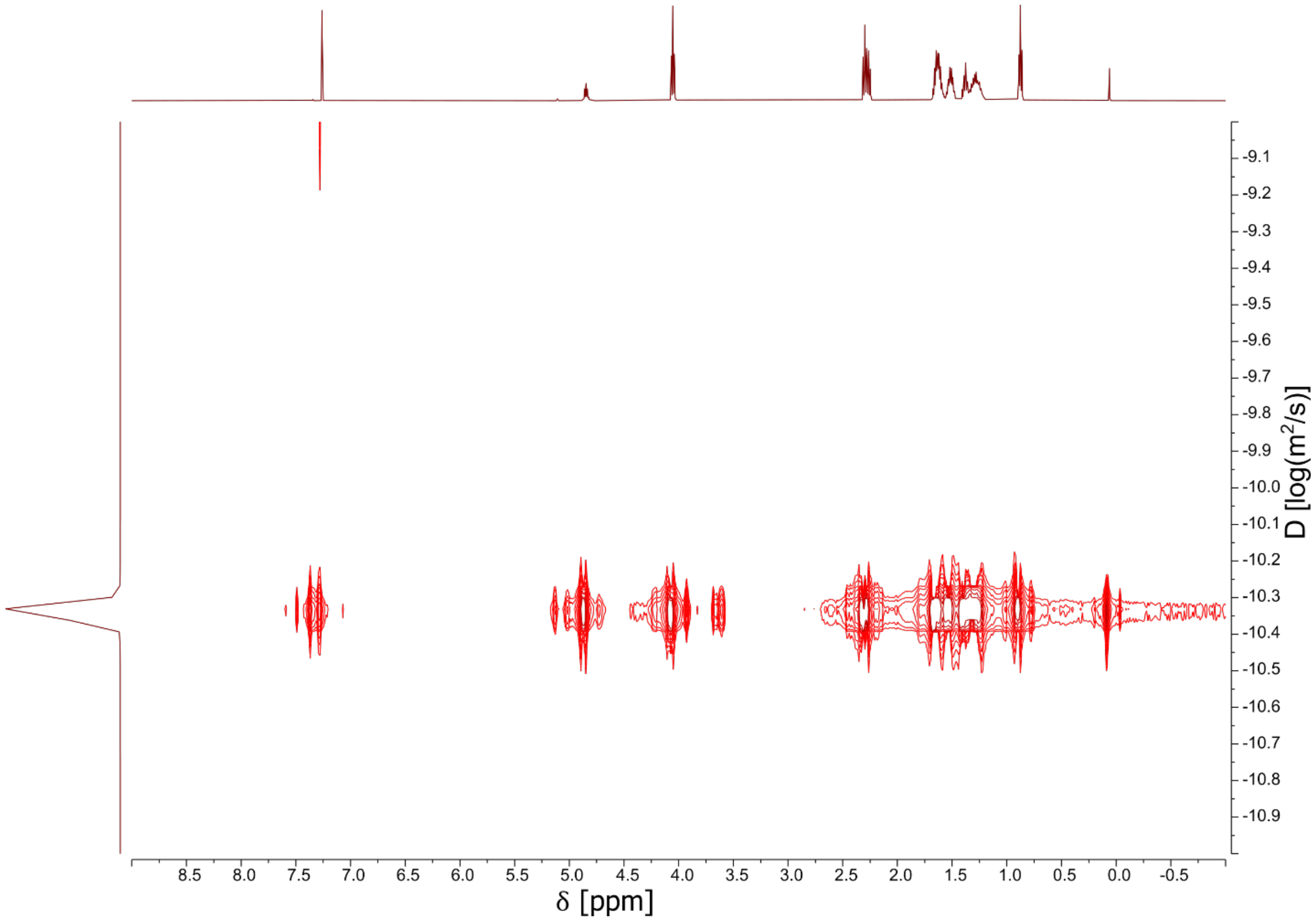

Figure S44. ^{1}H DOSY NMR spectrum (500 MHz, $CDCl_3$) of PCL-PDL (Table 1, entry 7).

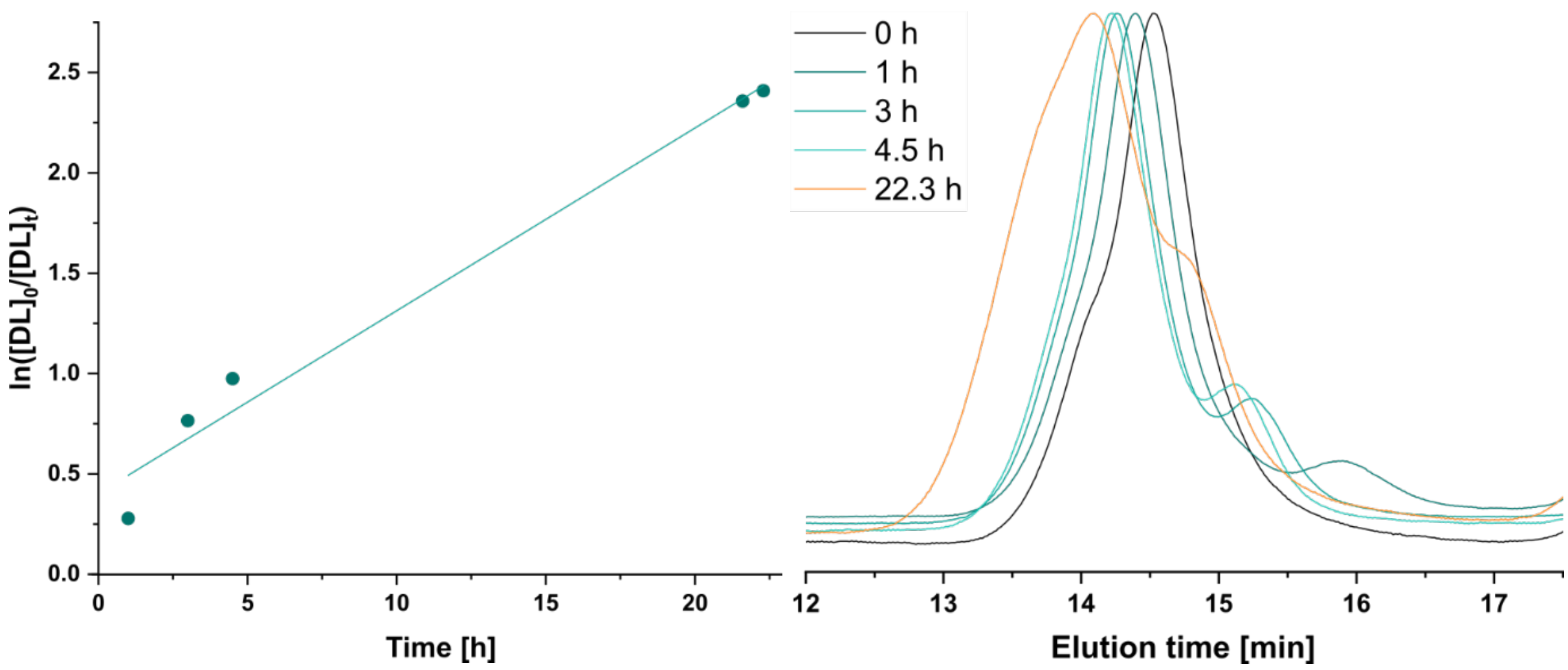

Figure S45. Kinetic plot for the polymerization of DL in the synthesis of PCL-PDL (k_{obs} = 2.5 × 10^{-5} s^{-1}, R^2 = 0.98) (left) and SEC chromatograms of aliquots taken during the second (DL) step of the copolymerization (right).

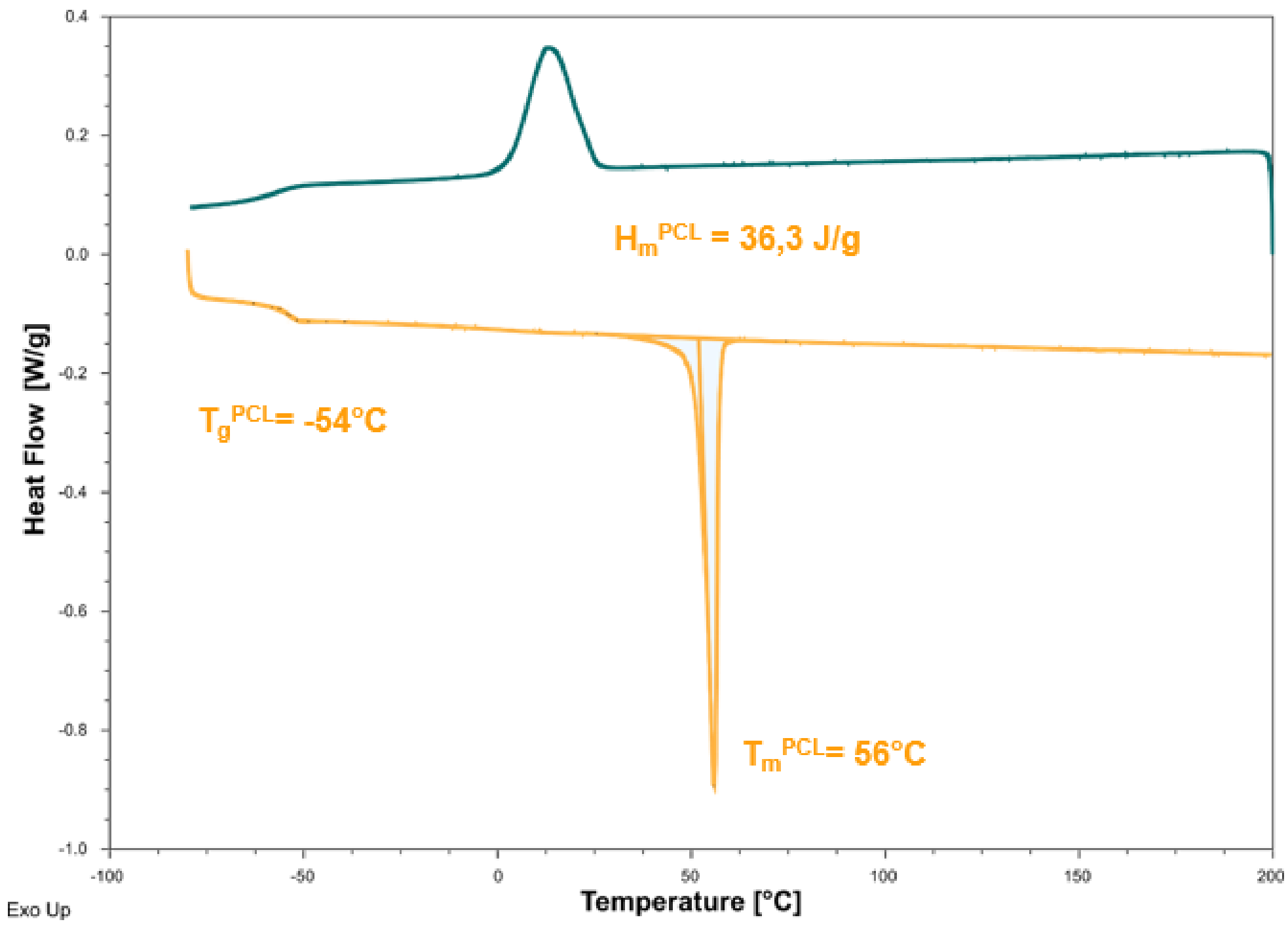

Figure S46. DSC curve of PCL-PDL for the first cooling and second heating cycle (Table 1, entry 7).

2.2.5.2. PDL first

Under an argon atmosphere in a glovebox, DL (234.5 mg, 240.2 µL, 1.38 mmol) was dissolved in dry toluene (1 mL). In a separate vial, **LZn_2Et** (11 mg, 13.9 µmol) was dissolved in dry toluene (0.39 mL) and BnOH (1.5 mg, 1.4 µL, 13.9 µmol) from a stock solution (0.5 µM in toluene) was added. The two solutions were stirred separately for 30 min at 70 °C and then combined by adding the DL to the catalyst solution. After 18.8 h, an aliquot was taken and CL (191.5 mg, 1.33 mmol) was added. Aliquots were taken at appropriate times to monitor the reaction until termination by quenching with $CDCl_3$. The product was obtained as a white powder by precipitation in cold, acidified methanol.

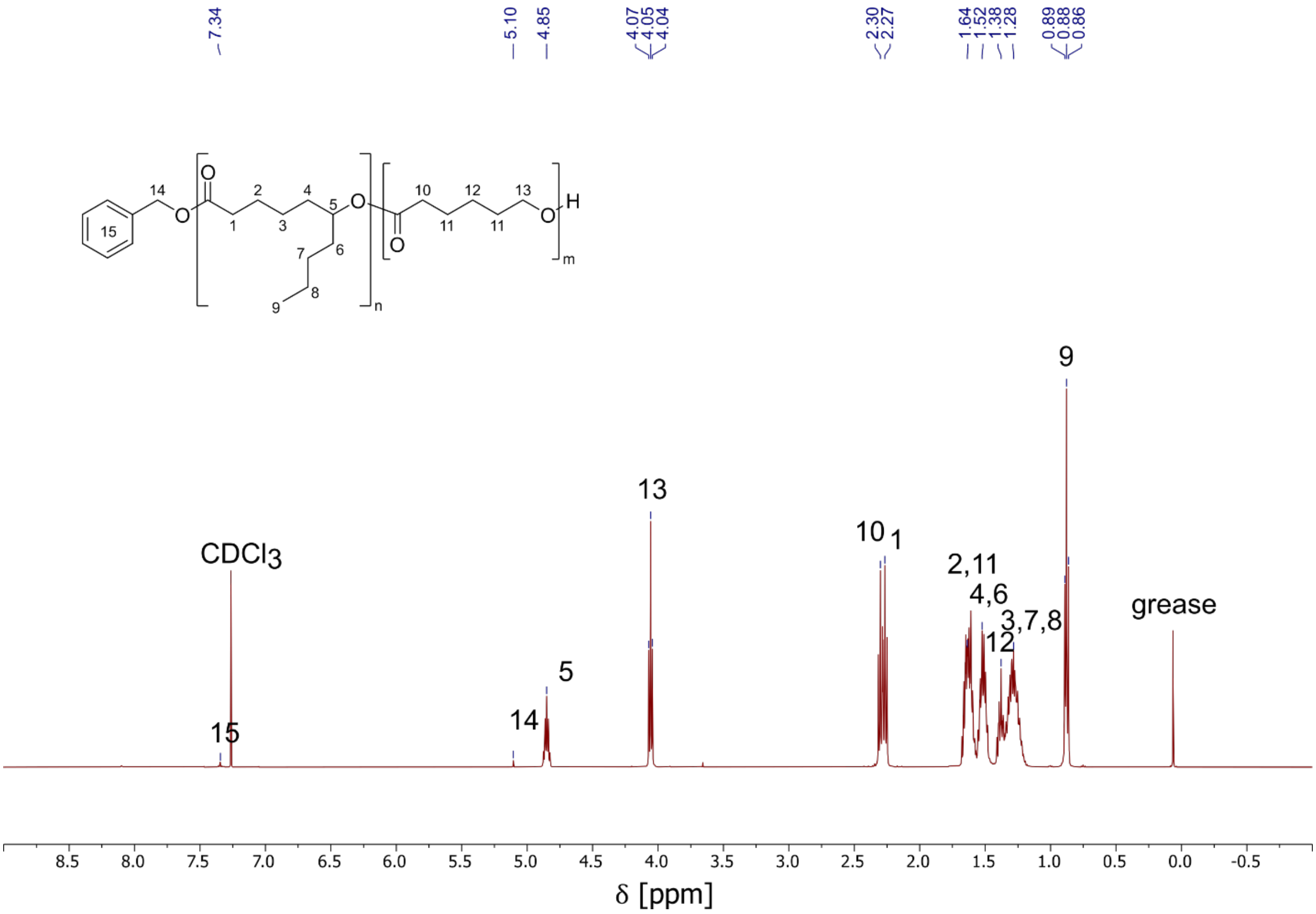

Figure S47. ^{1}H NMR spectrum (500 MHz, $CDCl_3$) of PDL-PCL assigned according to literature (Table 1, entry 8).[5]

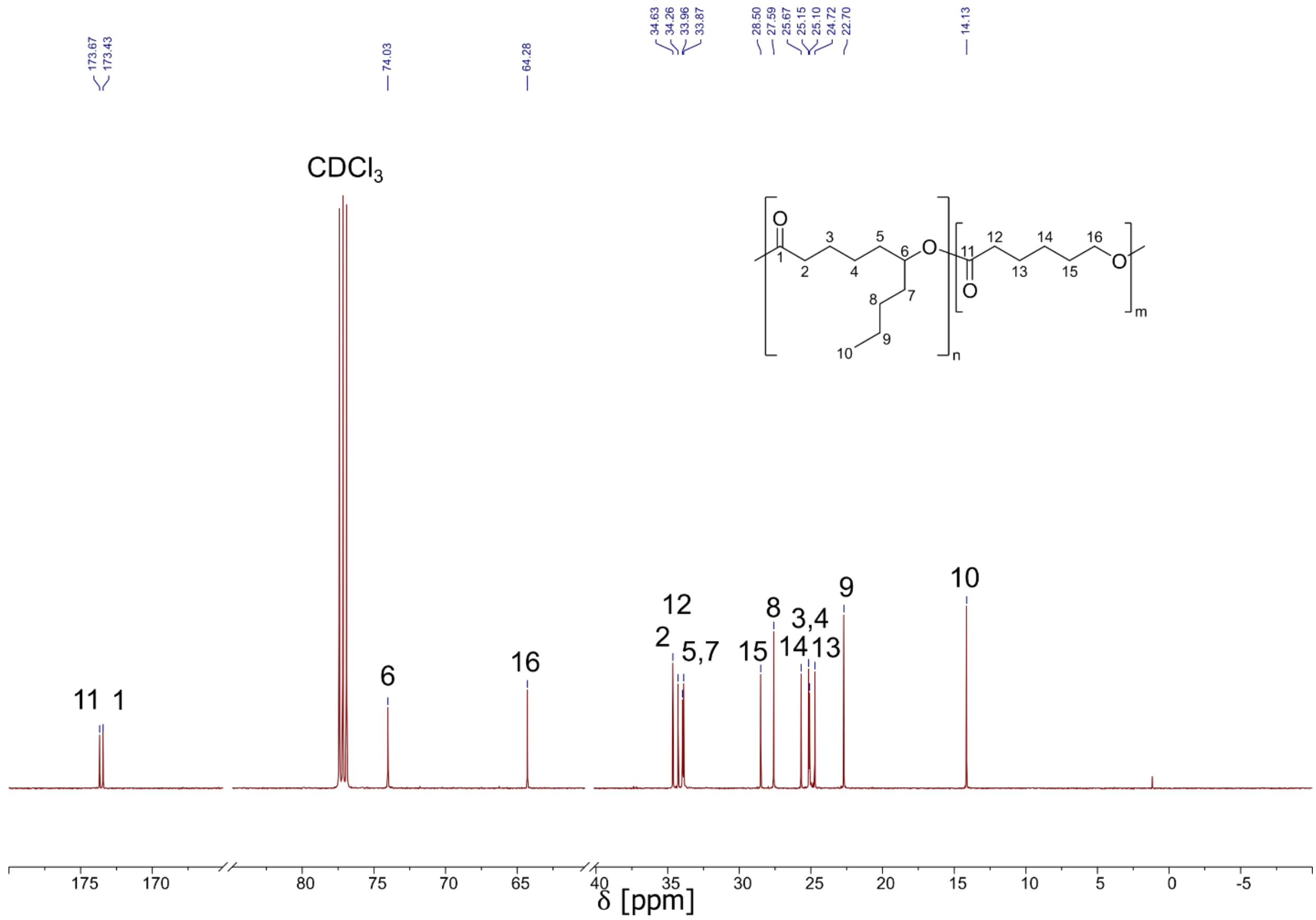

Figure S48. ^{13}C NMR spectrum (126 MHz, $CDCl_3$) of PDL-PCL assigned according to literature (Table 1, entry 8).[5]

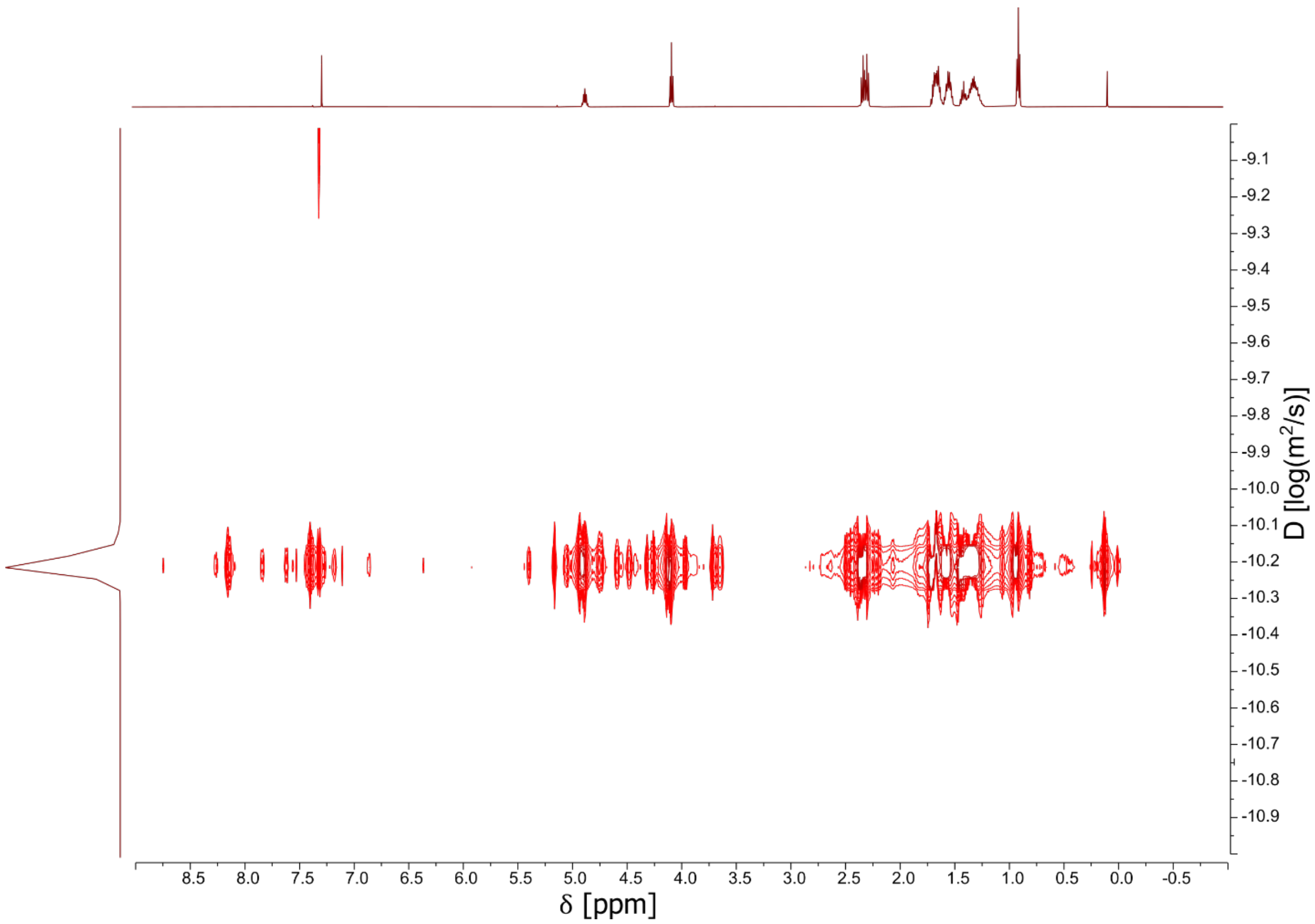

Figure S49. ^{1}H DOSY NMR spectrum (500 MHz, $CDCl_3$) of PDL-PCL (Table 1, entry 8).

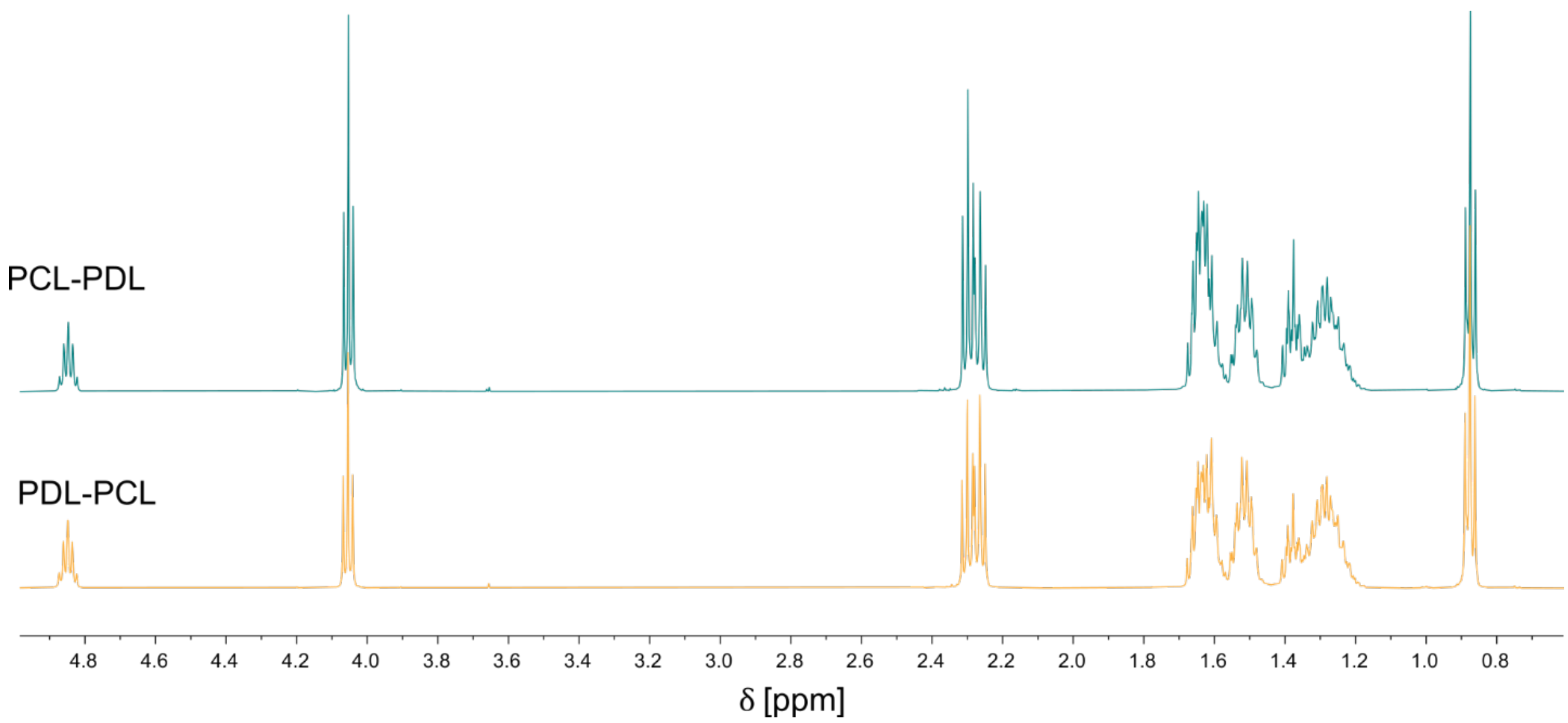

Figure S50. Stacked ^{1}H NMR spectra (500 MHz, $CDCl_3$) of PCL-PDL and PDL-PCL (Table 1, entry 7 (above) and entry 8 (below)).

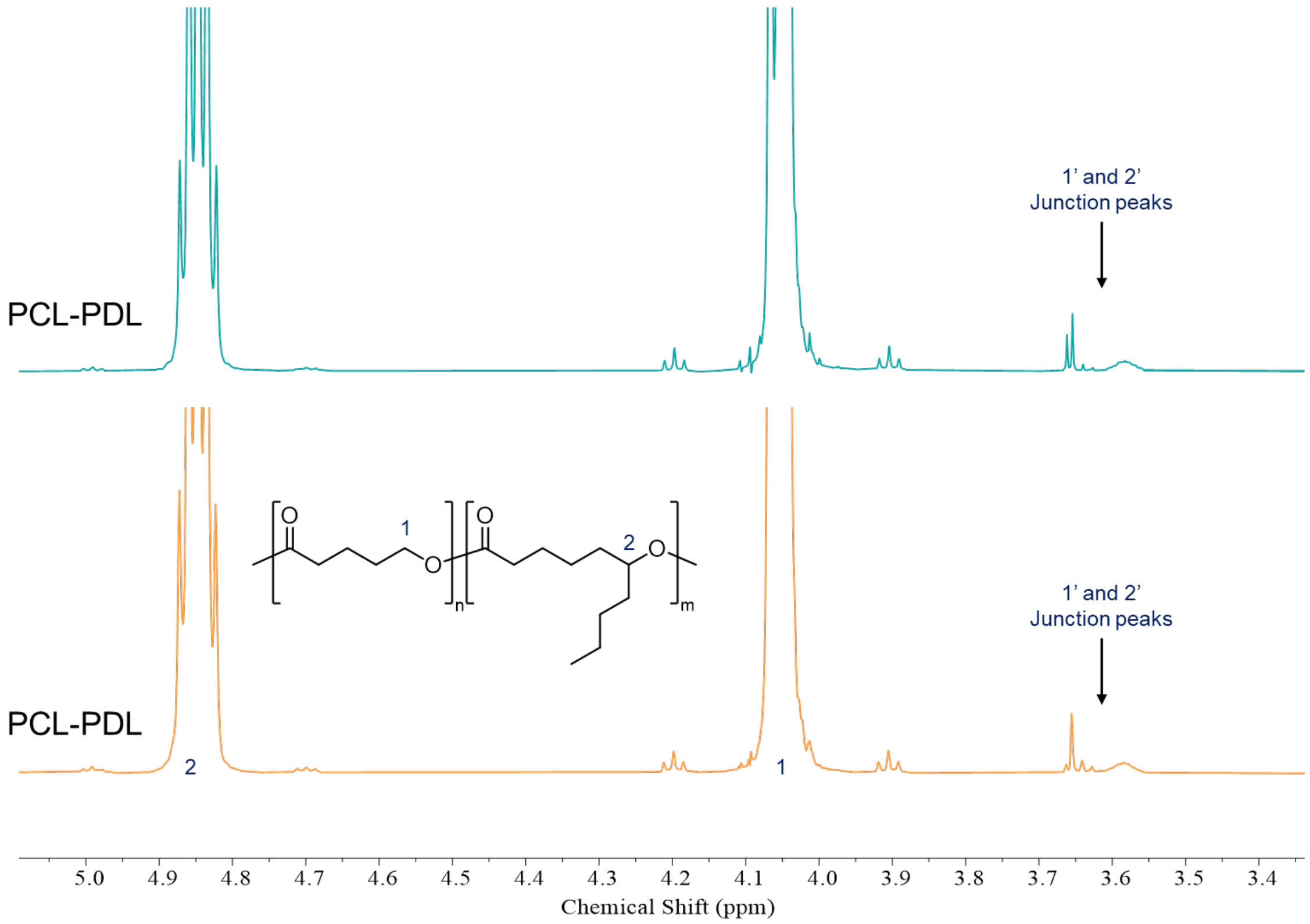

Figure S51. Zoom in of relevant ^1H signals and junction peaks for DL/CL BCPs (Table 1, entry 7 (above) and entry 8 (below)). Assigned based on the work of Bourissou and co-workers.[6]

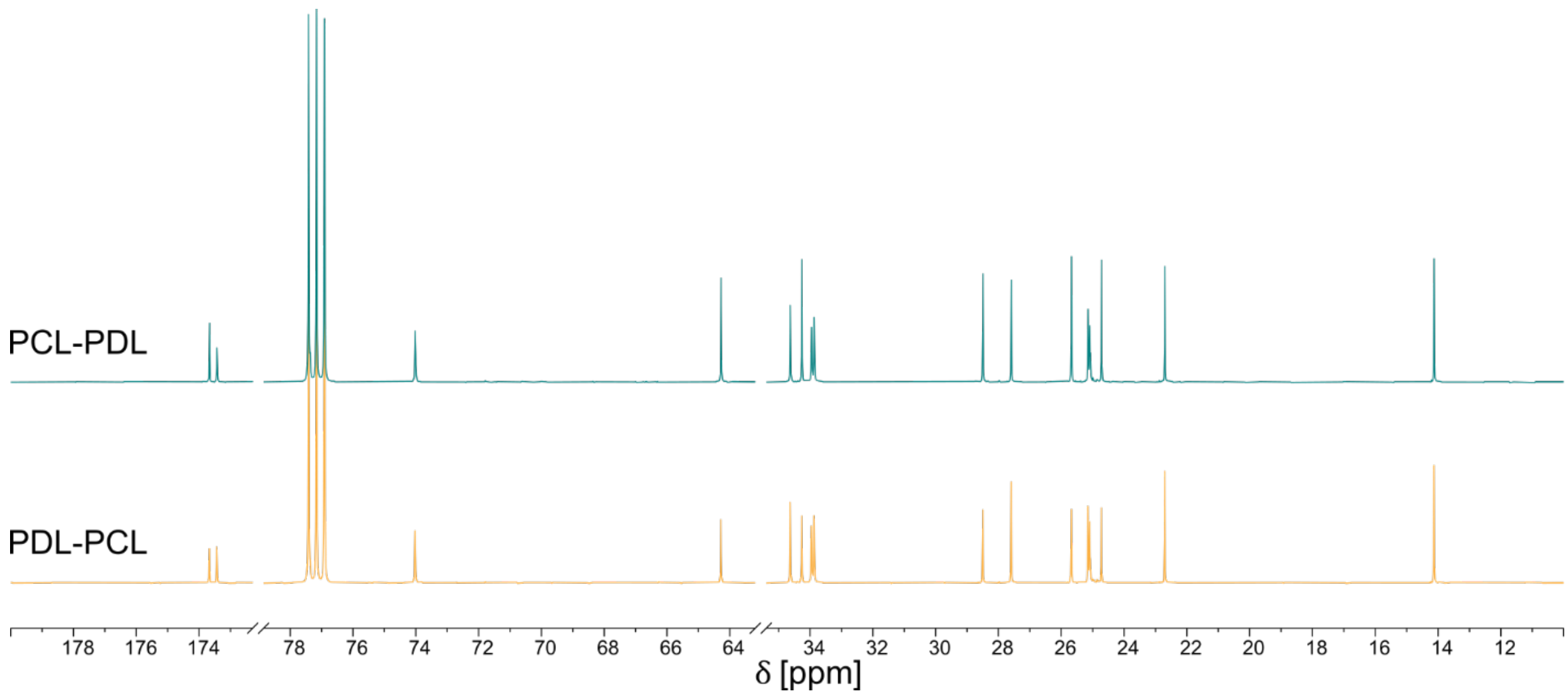

Figure S52. Stacked ^{13}C NMR spectra (126 MHz, $CDCl_3$) of PCL-PDL and PDL-PCL (Table 1, entry 7 (above) and entry 8 (below)).

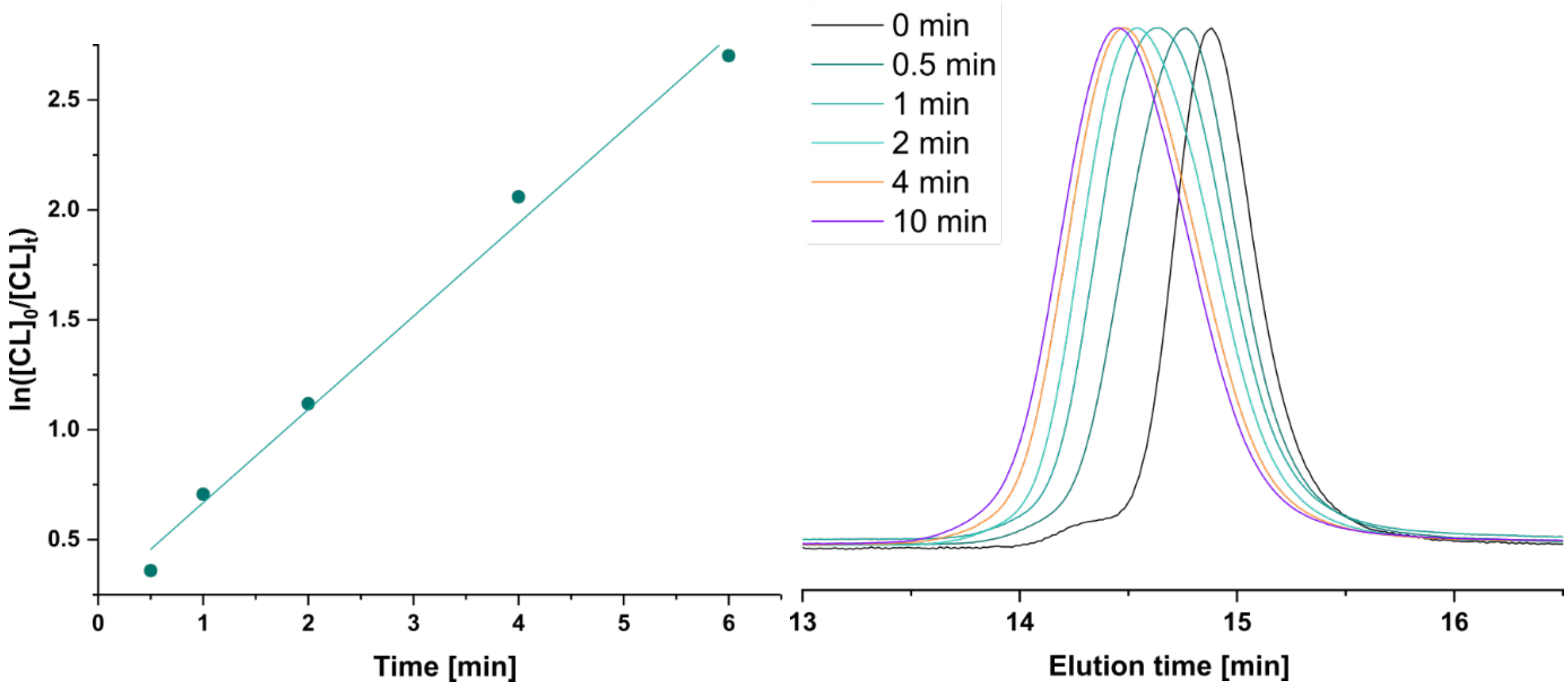

Figure S53. Kinetic plot for the polymerization of CL in the synthesis of PDL-PCL (k_{obs} = 7.1 × 10^{-3} s^{-1}, R^2 = 0.99) (left) and SEC chromatograms of aliquots taken during the second (CL) step of the copolymerization (right).

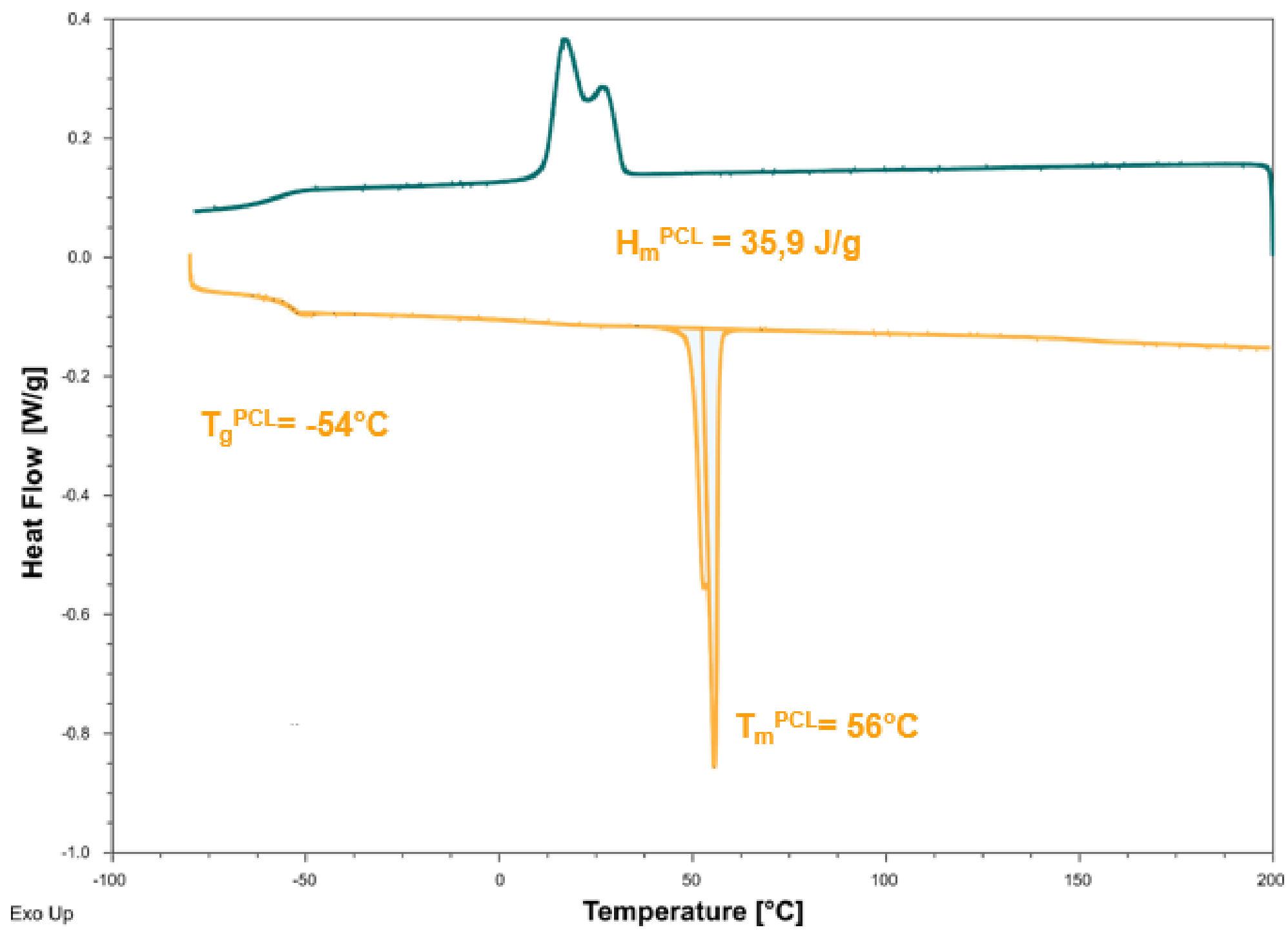

Figure S54. DSC curve of the first cooling and second heating curve of PDL-PCL.

2.2.6. PDL-PLA diblocks

Under an argon atmosphere, DL (234.5 mg, 240.2 µL, 1.38 mmol) was dissolved in dry toluene (1 mL). In a separate reaction vial, **LZn_2Et** (11 mg, 13.9 µmol) was dissolved in dry toluene (0.39 mL) and BnOH (1.5 mg, 1.4 µL, 13.9 µmol) from a stock solution (0.5 µM in toluene) was added. The two solutions were stirred separately for 30 min at 70 °C and then combined by adding the DL to the catalyst solution. After 18.8 h, an aliquot was taken and the solution was added to a solution of *rac*-LA (191.5 mg, 1.33 mmol) in 1 mL toluene that was pre-stirred *via* the same procedure (30 minutes at 70 °C) to aid *rac*-LA dissolution. Aliquots were taken at appropriate times to monitor the reaction until termination by quenching with $CDCl_3$. The product was obtained as a white solid by precipitation in cold, acidified methanol. Block structure confirmed by comparison to spectra by Odelius and co-workers.[7]

^{1}H NMR (500 MHz, $CDCl_3$) δ 7.37-7.31 (m, 5H, aromatic), 5.24-5.13 (m, 2H·m, -C*H*(CH_3)O-), 4.87-4.82 (quint, 1H·n, -C*H*BuO-), 2.28-2.25 (t, 2H·n, -COC$H_2C_3H_6$CBuHO-), 1.62-1.48 (m, 6H·n + 6H·m, -COCH_2CH_2-,-CH_2CHO-, -CH(CH_3)O-), 1.35-1.22 (m, 6H·n, -$CH_2CH_2CH_2$-), 0.89-0.86 (t, 3H·n, -CH_2CH_3).

^{13}C NMR (126 MHz, $CDCl_3$) δ 173.5, 169.7, 169.5, 169.2, 74.0, 69.3, 69.2, 51.0, 34.6, 34, 33.9, 31.1, 27.6, 25.1, 25., 25.0, 22.7, 16.9, 16.8, 16.7, 14.1.

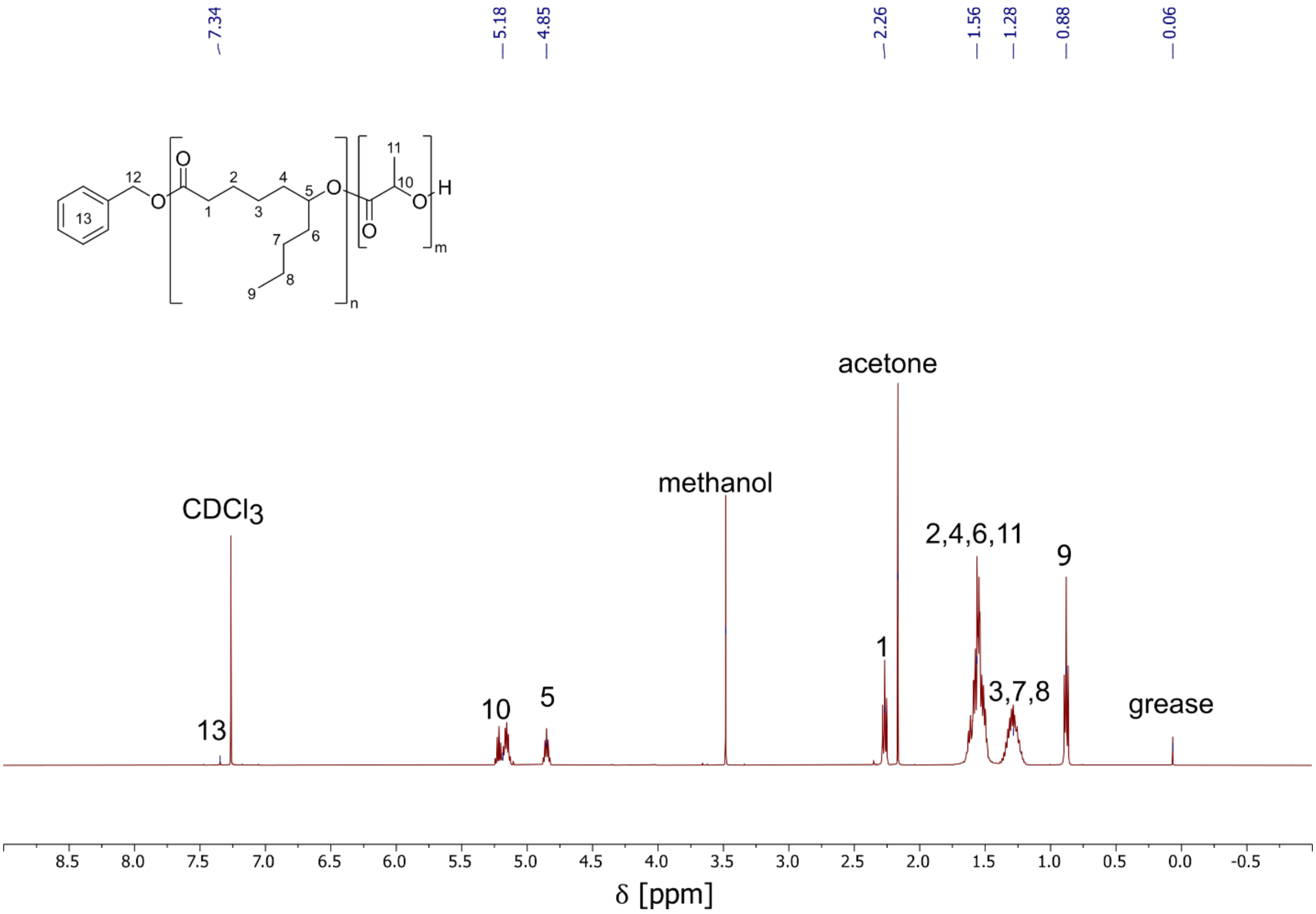

Figure S55. ^{1}H NMR spectrum (500 MHz, $CDCl_3$) of PDL-PLA (Table 1, entry 10).

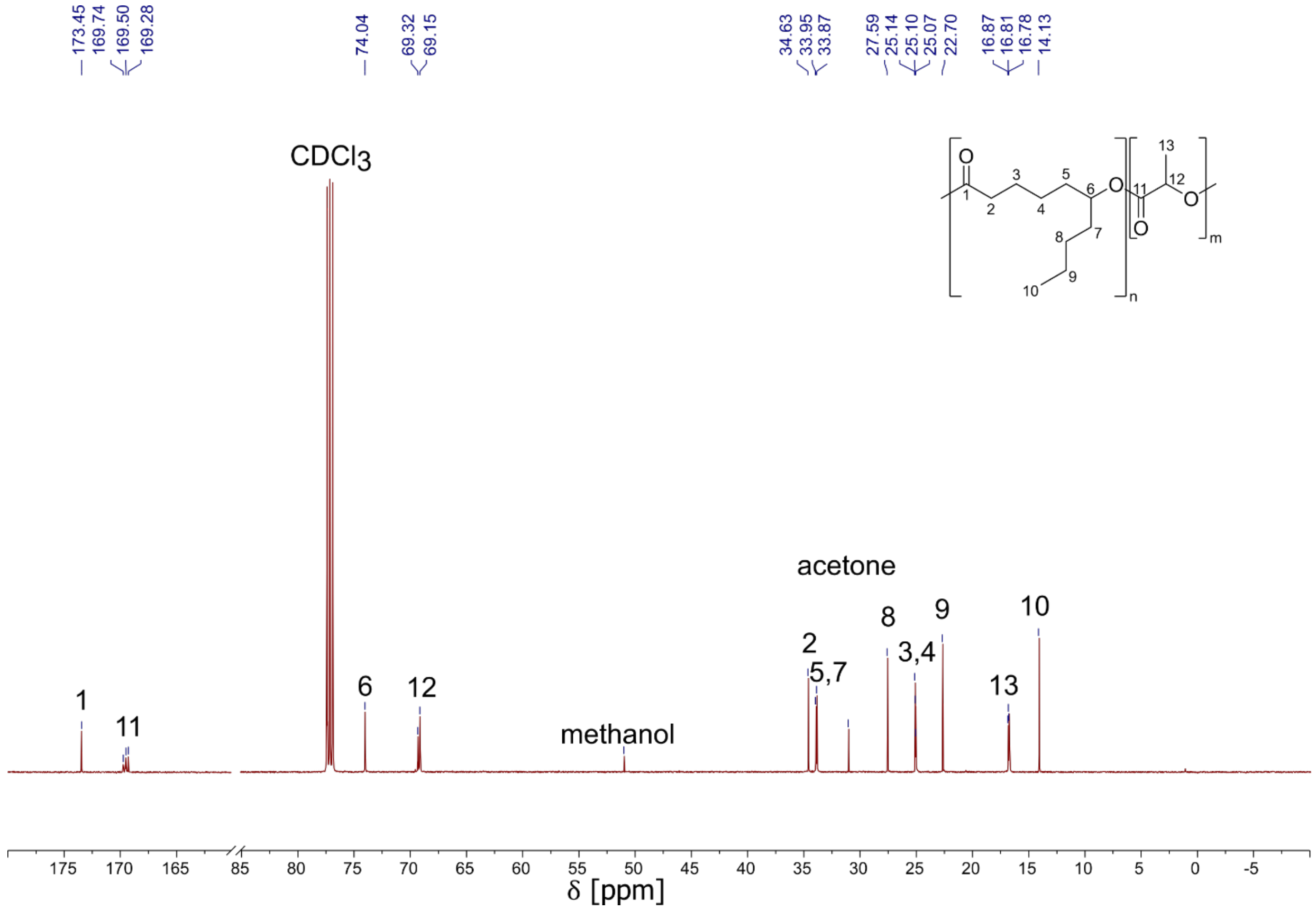

Figure S56. ^{13}C NMR spectrum (126 MHz, CDCl$_3$) of PDL-PLA (Table 1, entry 10).

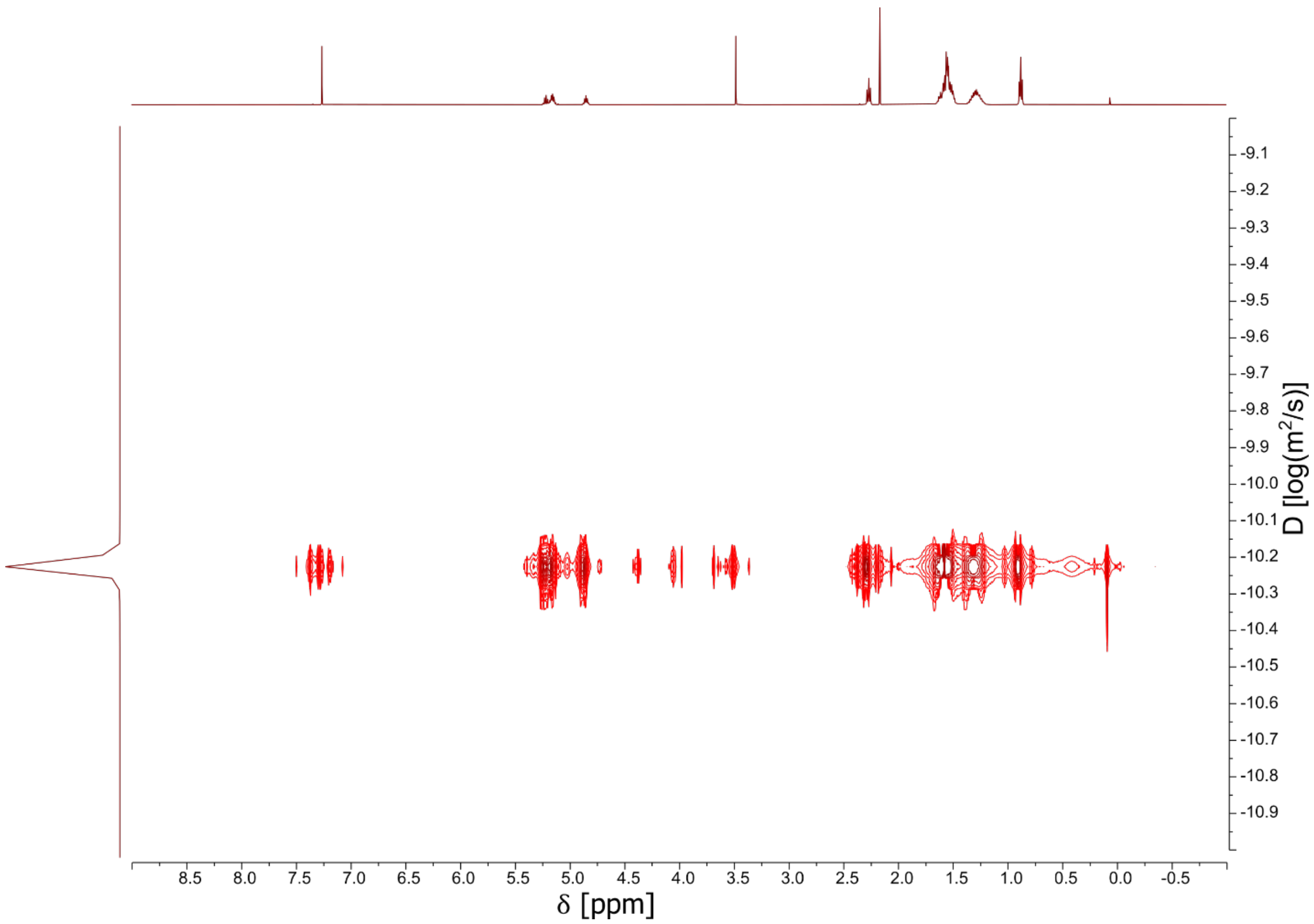

Figure S57. ^{1}H DOSY spectrum (500 MHz, $CDCl_3$) of PDL-PLA (Table 1, entry 10).

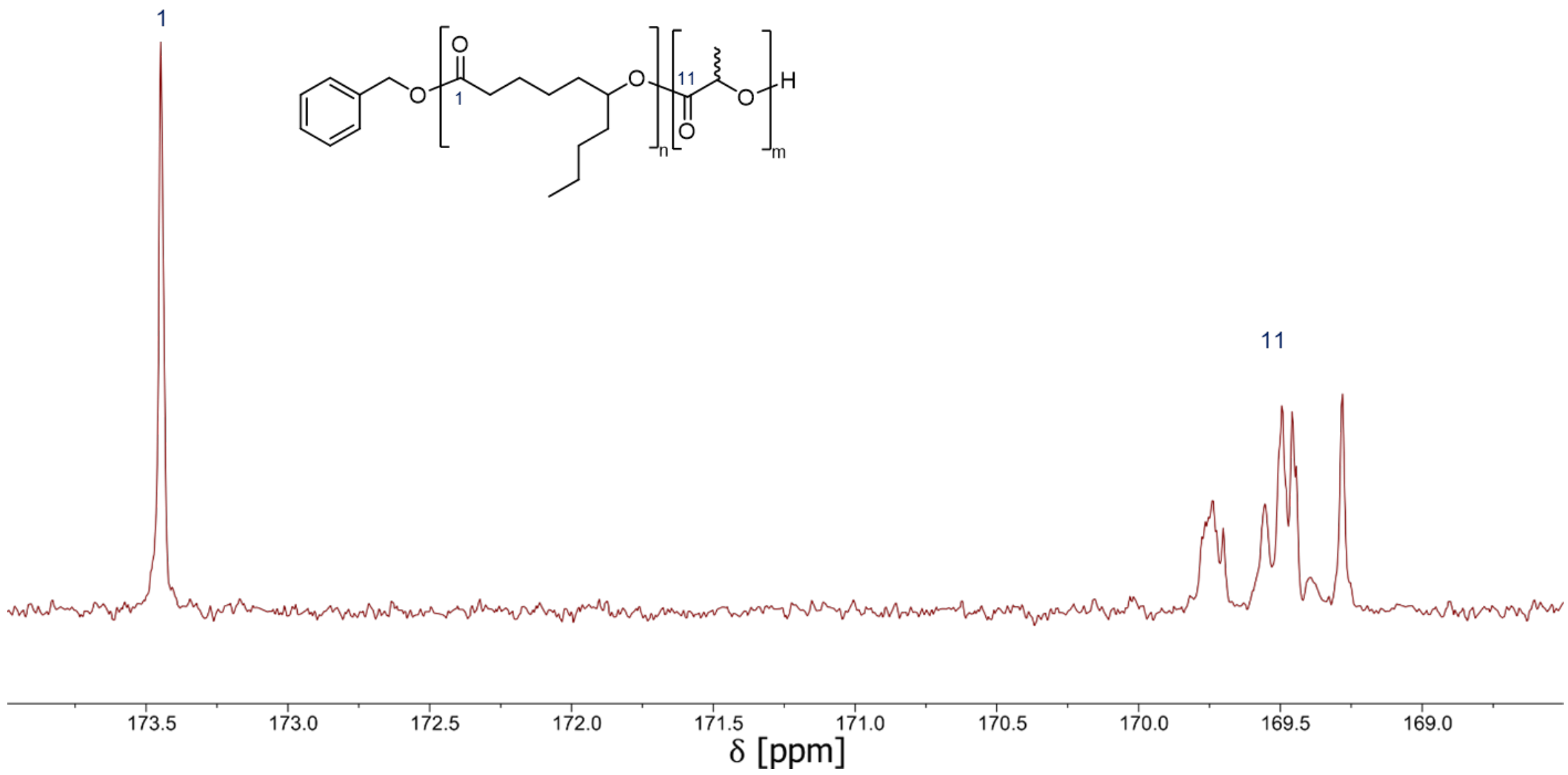

Figure S58. Zoom in of S56, PDL-PLA ^{13}C NMR spectrum (126 MHz, $CDCl_3$) demonstrating the difference in carbonyl peak splitting associated with PDL and PLA (i.e. only one carbonyl environment is observer for PDL, whereas several are observed for PLA due to rac-LA being used, with no tacticity control) (Table 1, entry 10).

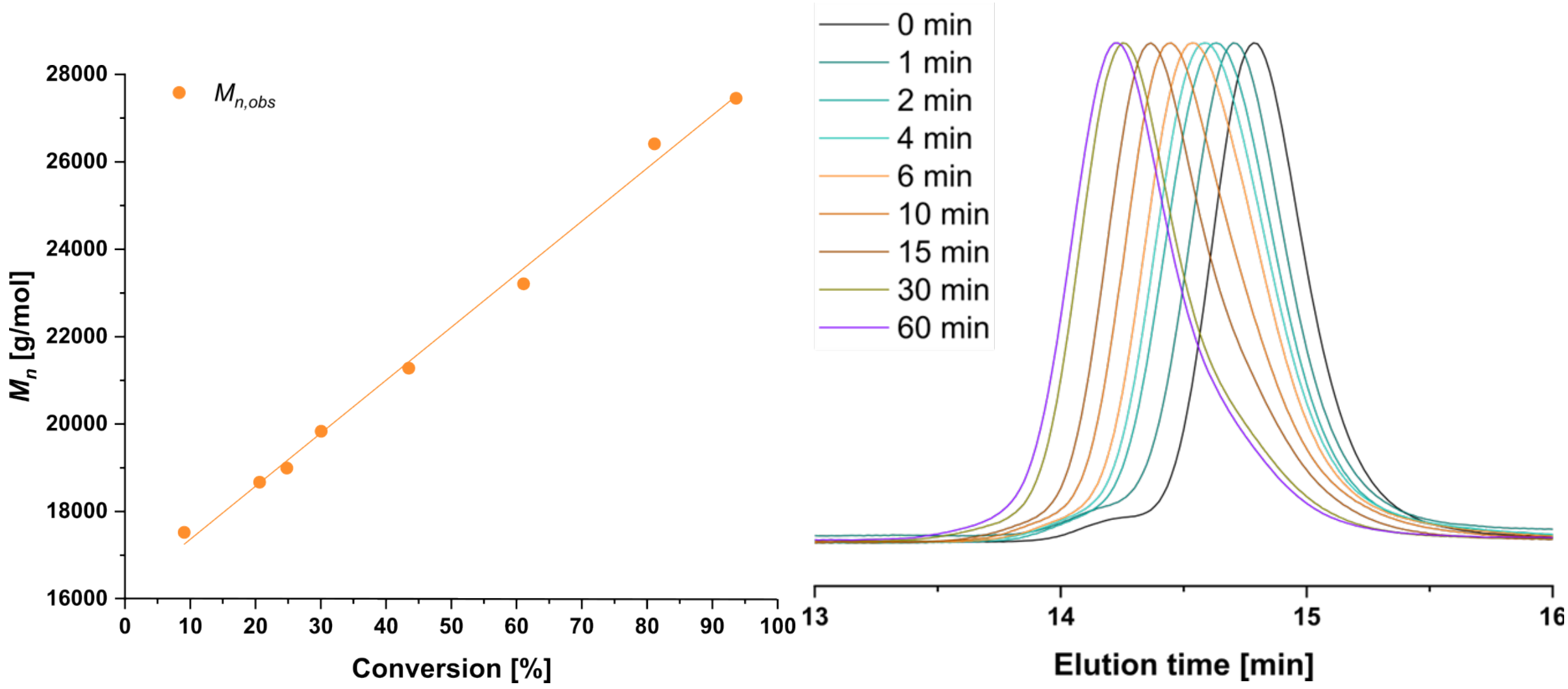

Figure S59. Rac-*LA ROP from PDL*. M*$_{n,obs}$ *obtained vs conversion (left), highlighting living character.* $M_{n,obs}$ *calculated from SEC traces of aliquots taken during the copolymerisation (right) using a refractive index detector calibrated against PS standards (Table 1, entry 10).*

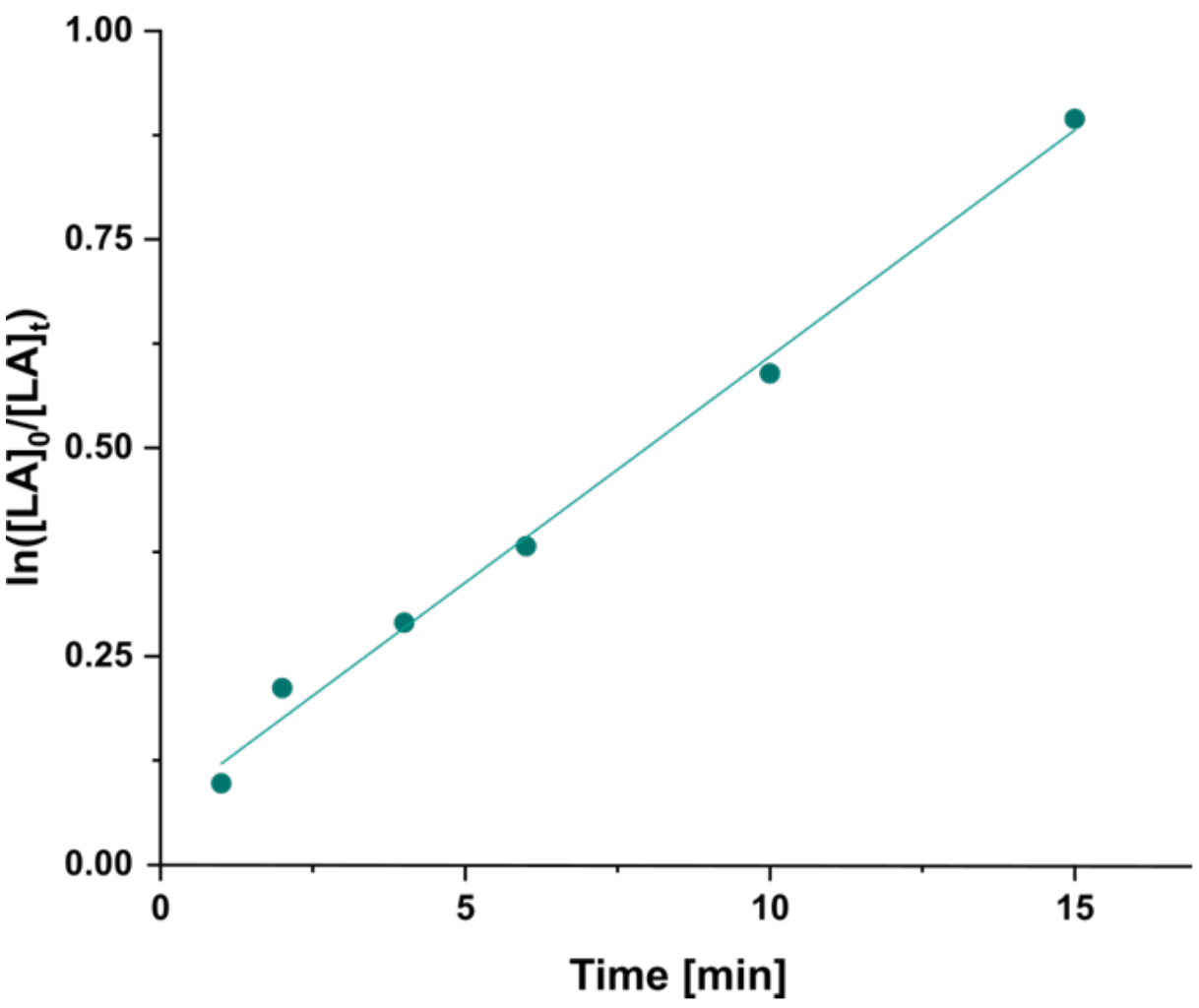

Figure S60. Kinetic plot for rac-LA conversion in the sequential copolymerisation of PDL-PLA (k_{obs} = 9.0 × 10^{-4} s^{-1}, R^2 = 0.99) (Table 1, entry 10).

Table S4. Molecular weight and dispersity over time for the PDL-PLA synthesis from PDL at near full conversion (>94%) (Table 1, entry 10).*

Entry	Time (min)	Conv.[a] (%)	$M_{n,theo}$[b] (kDa(	$M_{n,obs}$[c] (kDa)	$Đ$[c]
1[d]	0	0	15.4	14.6[d]	1.1
2	1	9	16.8	17.5	1.1
3	2	21	18.2	18.7	1.1
4	4	25	19.0	19.0	1.1
5	6	30	20.0	19.8	1.1
6	10	44	21.8	21.3	1.1
7	15	61	24.0	23.2	1.1
8	30	81	27.6	26.4	1.1
9	60	94	29.1	27.4	1.1

[a] determined from ^{1}H NMR
[b] calculated from conversion,
$M_{n,Theo}$ = $conversion_{DL} \times M_{DL} \times ([DL]_0/[LZn_2OBn]) + conversion_{LA} \times M_{LA} \times ([LA]_0/[$**LZn₂OBn**$])$
[c] obtained by SEC in THF using a PS standard.
[d] Molecular weight is corrected by a factor of 0.91 for PDL.

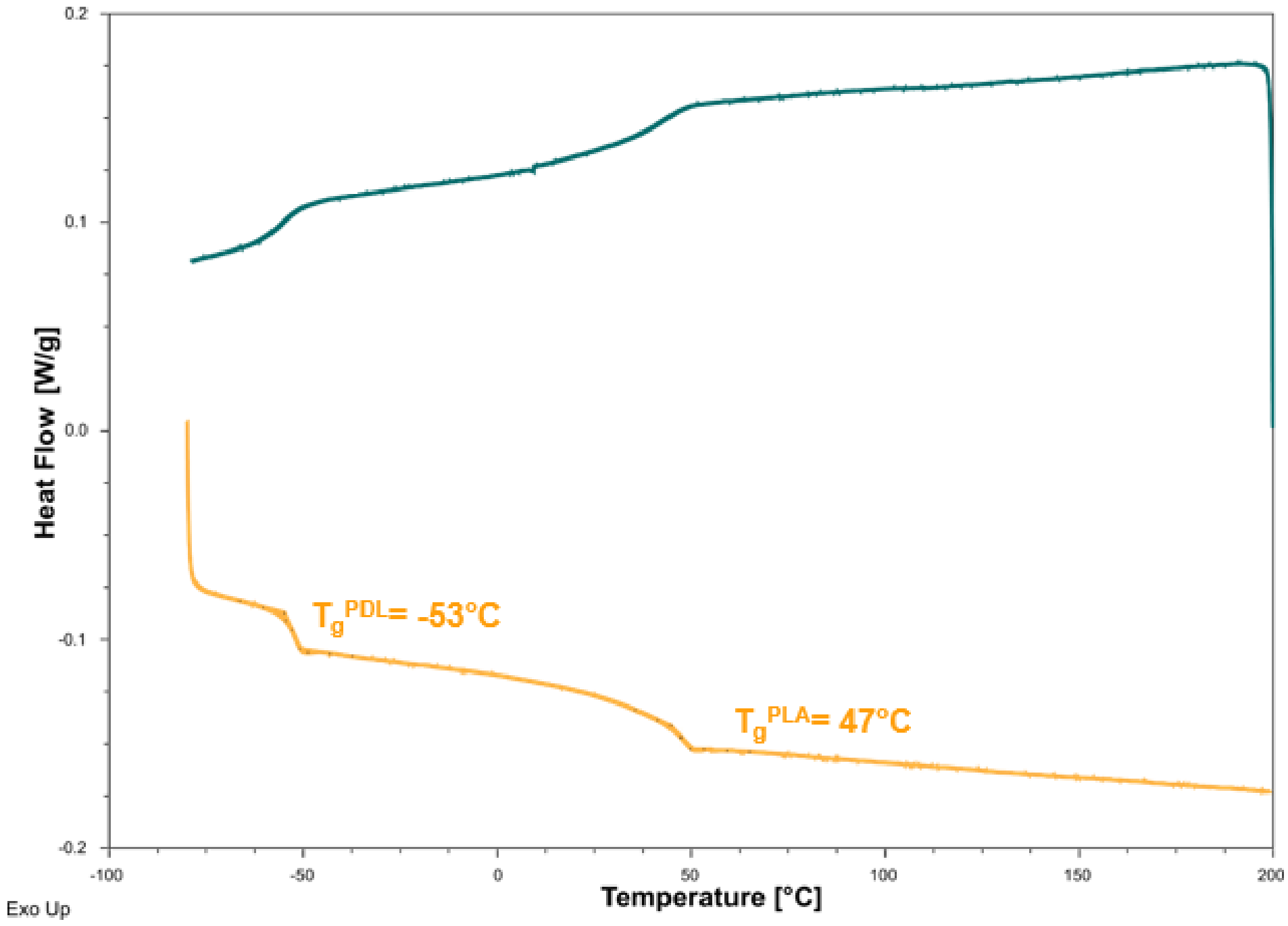

Figure S61. DSC curve (first cooling, second heating) of PDL-PLA (Table 1, entry 10).

2.2.7. PpDL-CL diblocks

2.2.7.1. Sequential addition, pDL first

Under an argon atmosphere in a glovebox, pDL (132 mg, 0.69 mmol) was dissolved in dry toluene (0.2 mL). In a separate reaction vial, **LZn_2Et** (11 mg, 13.9 µmol) was dissolved in dry toluene (0.1 mL) and BnOH (1.5 mg, 1.4 µL, 13.9 µmol) from a stock solution (0.5 µM in toluene) was added. The solutions were stirred separately for 30 min at 70 °C and then combined by adding the pDL solution to the catalyst solution. An aliquot was taken and the resulting polymer solution was added to a solution of CL (125 mg, 0.69 mmol) in 0.2 mL toluene that was pre-stirred via the same procedure (30 minutes at 70 °C). Aliquots were taken at appropriate times to monitor the reaction until termination by quenching with $CDCl_3$. The product was obtained as a white solid by precipitation in cold, acidified methanol.

^{1}H NMR (500 MHz, $CDCl_3$) δ 4.07-4.05 (m, 2H·n + 2H·m, $-COCH_2(CH_2)_nCH_2O-$), 2.32-2.27 (m, 2H·n + 2H·m , $– COCH_2-$), 1.68-1.60 (m, 4H·n + 2H·m, $-COCH_2CH_2-$), 1.41-1.35 (m, 2H·m, $-CH_2CH_2CH_2$), 1.29-1.25 (m, 18H·n, $-CH_2-$).

^{13}C NMR (126 MHz, $CDCl_3$) δ 174.0, 173.5, 64.4, 64.2, 34.4, 34.1, 29.7, 29.6, , 29.5, 29.3, 29.3, 29.2, 28.7, 28.4, 25.9, 25.5, 25.0, 24.6 (note that there are multiple resonances due to the methylene groups of the PpDL block and heterojunctions between pDL and CL units).

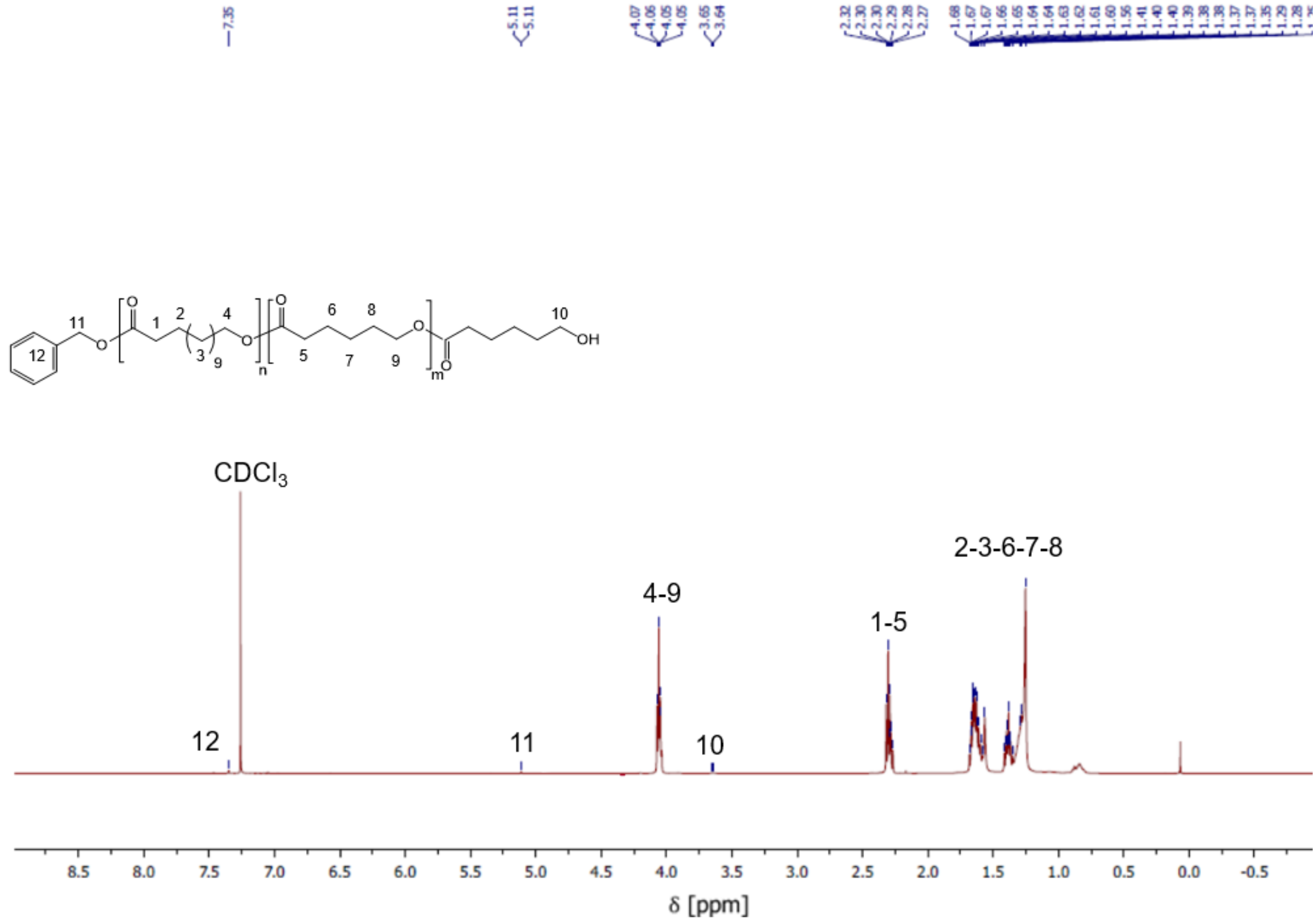

Figure S62. ¹H NMR spectrum (500 MHz, $CDCl_3$) of $PpDL_{40}$-PCL_{80} (Table 2, entry 2).

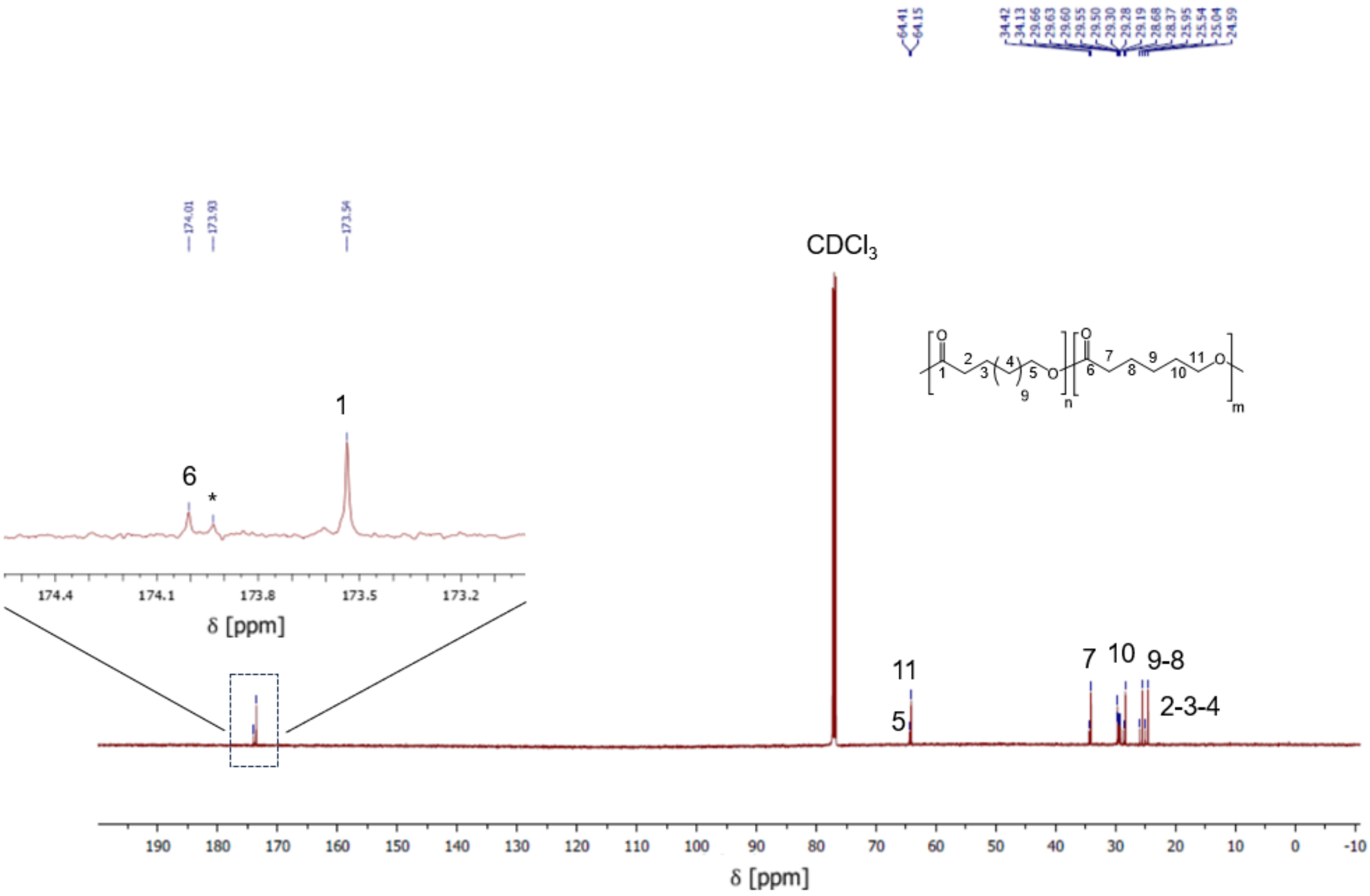

Figure S63. Quantitative ^{13}C NMR spectrum (126 MHz, $CDCl_3$) of $PpDL_{40}$-PCL_{80} (Table 2, entry 2).

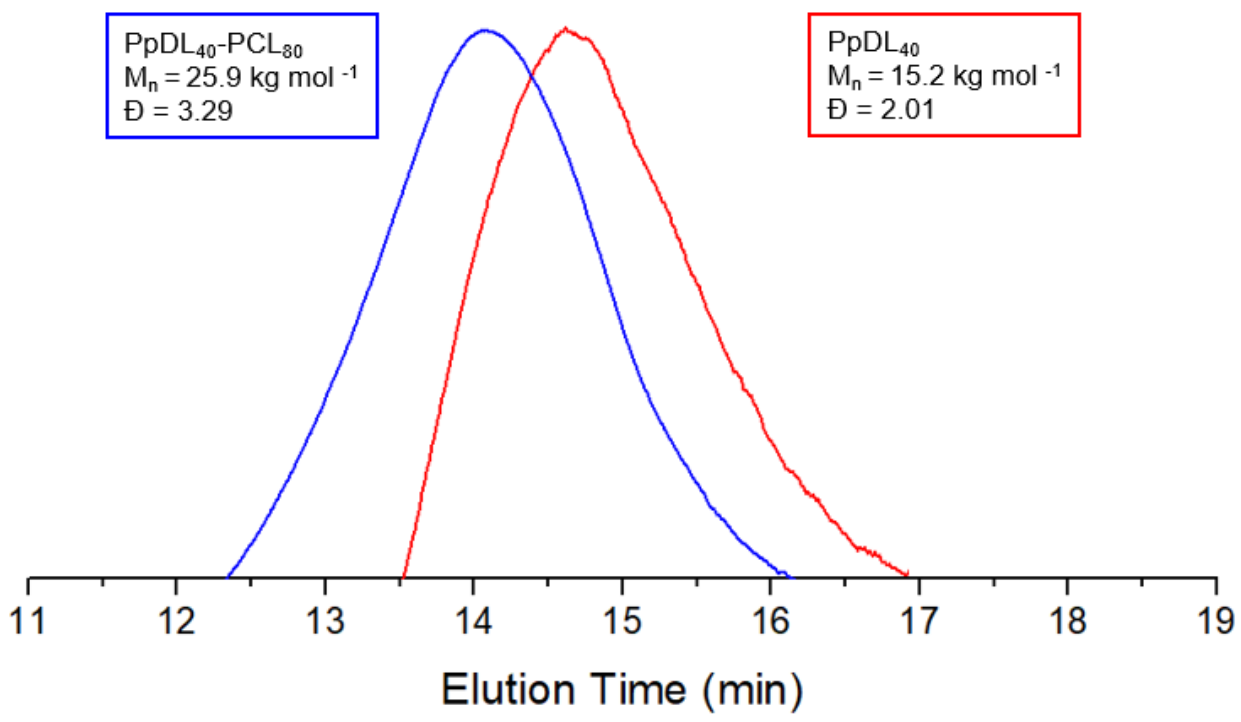

Figure S64. SEC traces of aliquots taken during the $PpDL_{40}$-PCL_{80} copolymerisation (Table 2, entry 2).

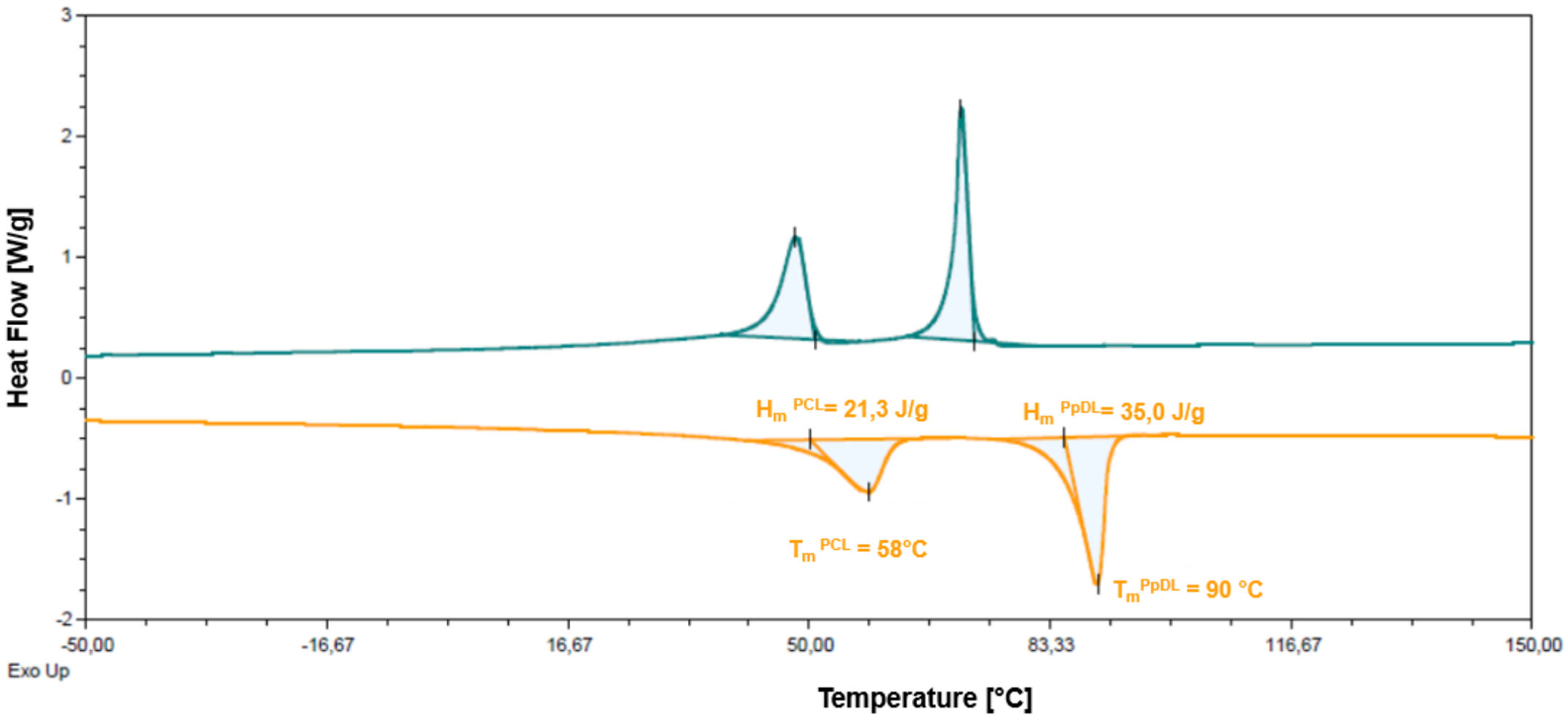

Figure S65. DSC curve (first cooling and second heating) of PpDL40-PCL80 (Table 2, entry 2).

2.2.7.2. Sequential addition, CL first*

Under an argon atmosphere in a glovebox, CL (63 mg, 0.56 mmol) was dissolved in dry toluene (0.5 mL). In a separate reaction vial, **LZn_2Et** (11 mg, 13.9 µmol) was dissolved in dry toluene (0.1 mL) and BnOH (1.5 mg, 1.4 µL, 13.9 µmol) from a stock solution (0.5 µM in toluene) was added. The two solutions were stirred separately for 30 min at 70 °C and then combined by adding the pDL to the catalyst solution. An aliquot was taken and the polymer solution was added to neat pDL (132 mg, 0.56 mmol). Aliquots were taken at appropriate times to monitor the reaction until termination by quenching with $CDCl_3$. The product was obtained as a white solid by precipitation in cold, acidified methanol.

^{1}H NMR (500 MHz, $CDCl_3$) δ 4.15-4.07(m, 2H·n + 2H·m, – $COCH_2(CH_2)_nCH_2O$-), 2.32-2.30 (m, 2H·n, -$COCH_2CH_2$), 1.67-1.63 (m, 2H·n + 2H·m , – $COCH_2$-), 1.68-1.60 (m, 4H·n + 2H·m, -$COCH_2CH_2$-), 1.41-1.35 (m, 2H·m, -$CH_2CH_2CH_2$), 1.29-1.25 (m, 18H·n, -CH_2-).

^{13}C NMR (126 MHz, $CDCl_3$) δ 174.0, 173.9, 173.6, 173.5, 64.5, 64.4, 64.1, 64.0, 34.4, 34.3, 34.2, 34.1, 30.9, 29.7, 29.6, 29.5, 29.4, 29.3, 29.2, 29.1, 28.7, 28.4, 28.3, 25.9, 25.5, 25.0, 24.6, 24.5 (note that there are multiple resonances due to the methylene groups of the PpDL block and heterojunctions between pDL and CL units).

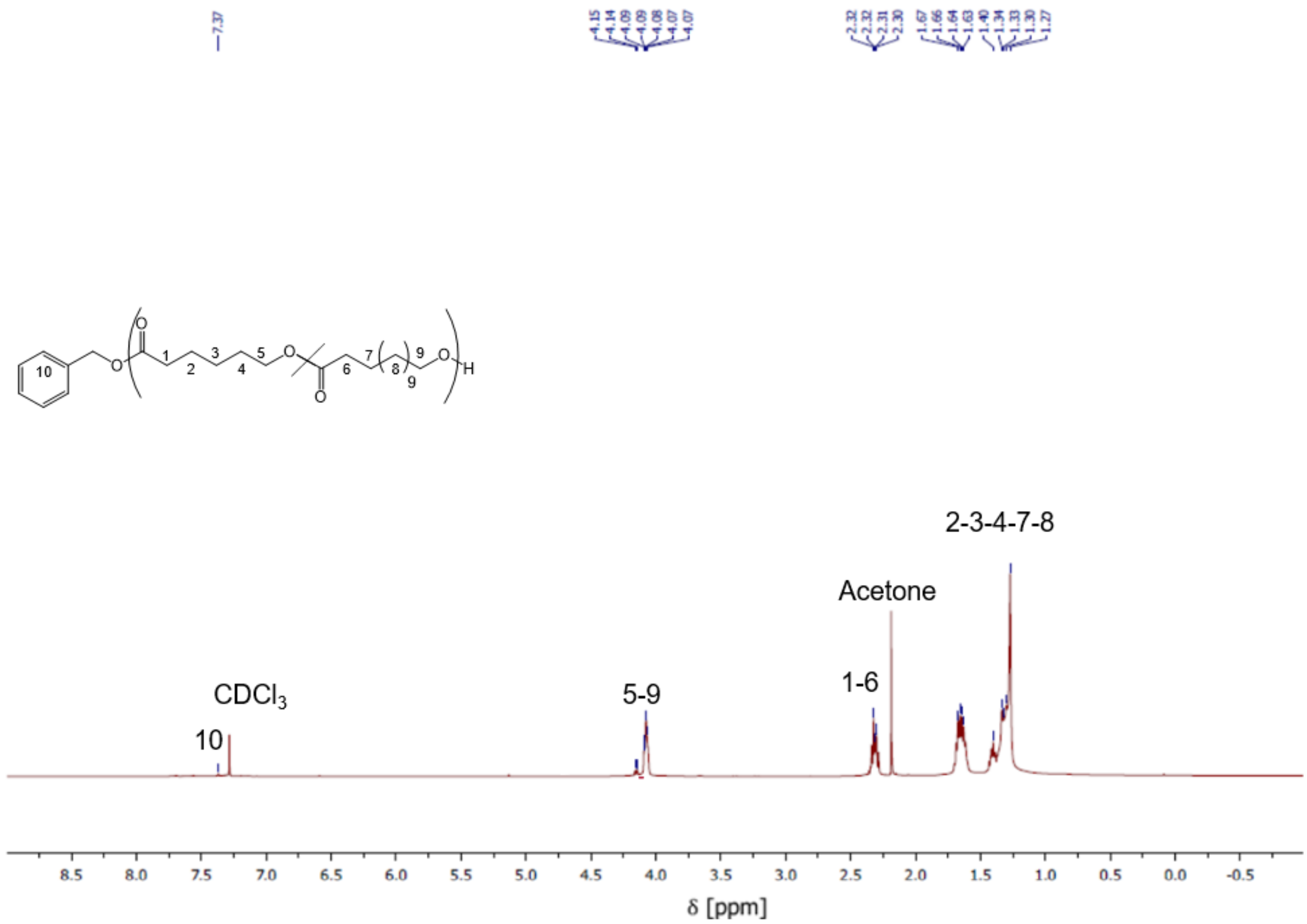

Figure S66. ¹H NMR spectrum (500 MHz, $CDCl_3$) of PCL_{40}-$PpDL_{40}$ (Table 2, entry 3).

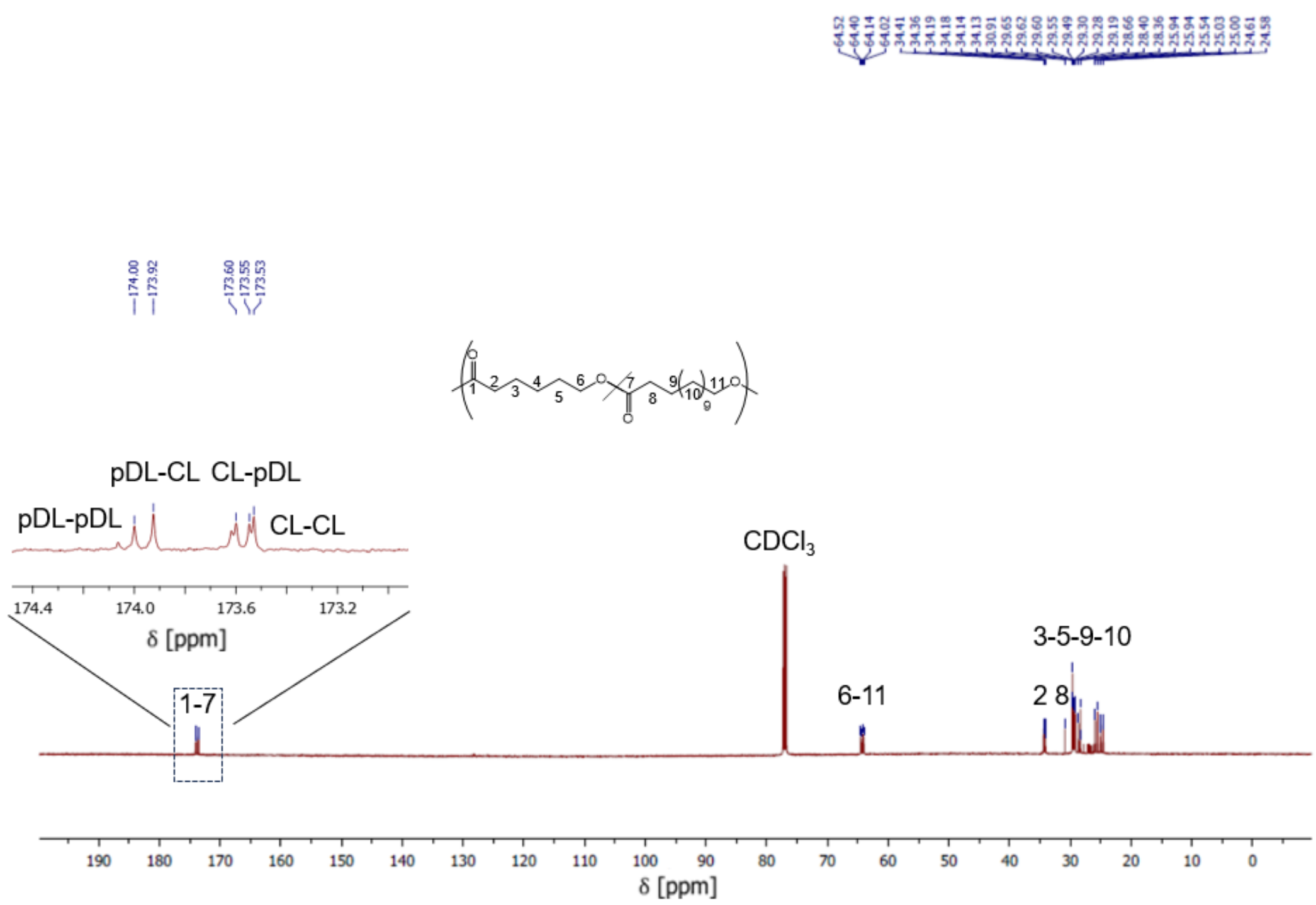

Figure S67. Quantitative ¹³C NMR spectrum (126 MHz, $CDCl_3$) of PCL_{40}-$PpDL_{40}$ (Table 2, entry 3).

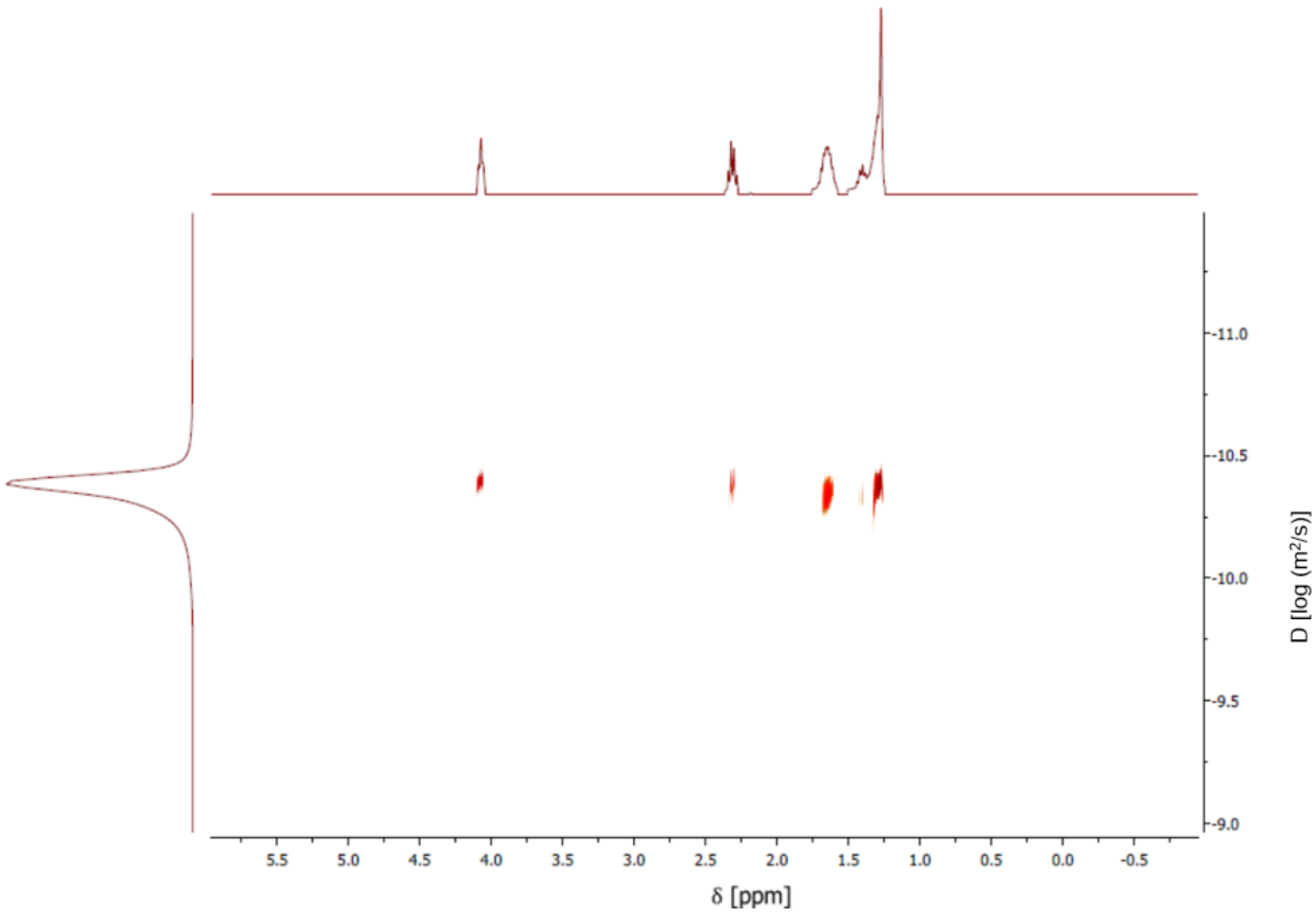

Figure S68. ^1H DOSY NMR spectrum (500 MHz, $CDCl_3$) of PCL_{40}-$PpDL_{40}$ (Table 2, entry 3).

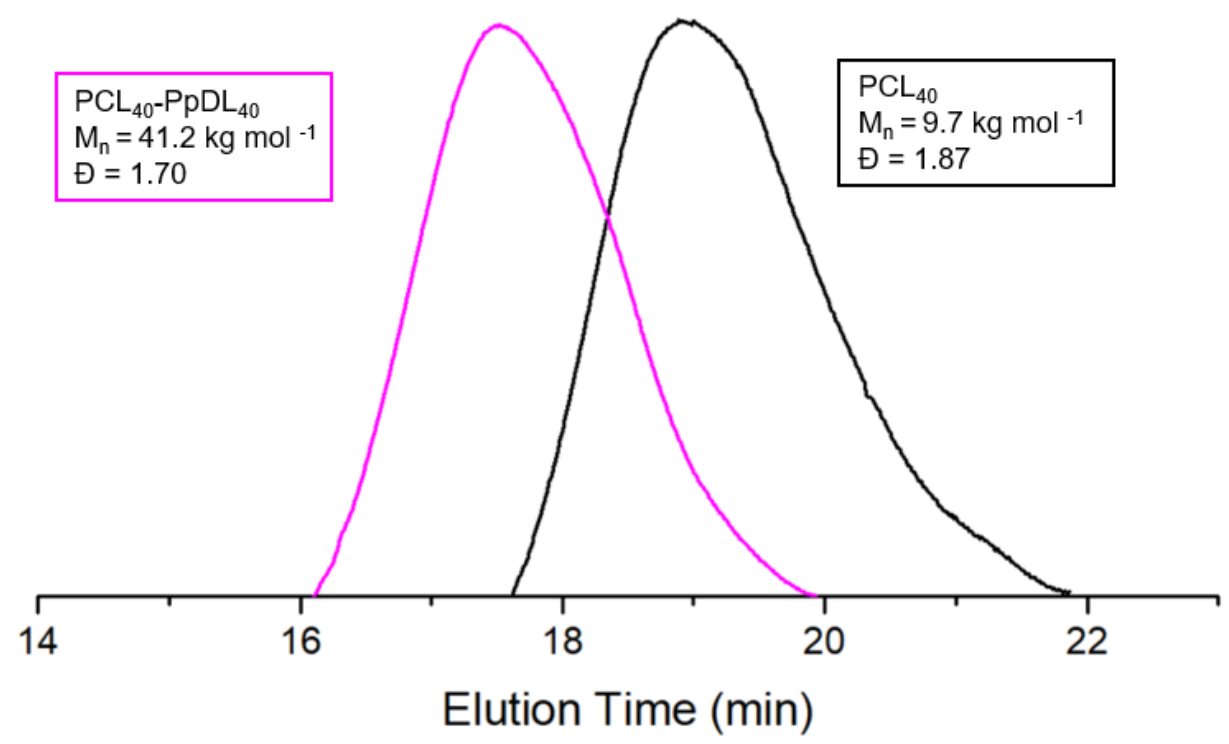

Figure S69. SEC traces of aliquots taken during the PCL_{40}-$PpDL_{40}$ copolymerisation (Table 2, entry 3).

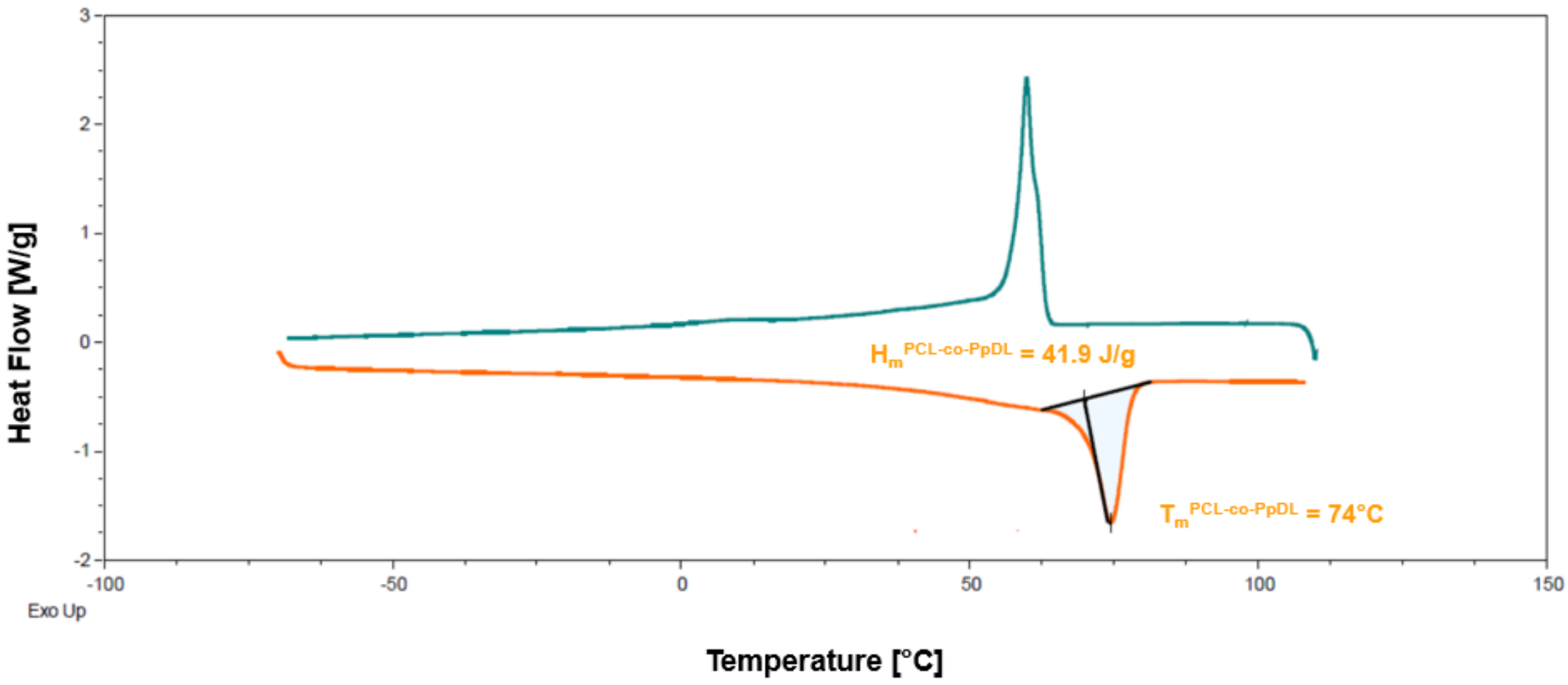

Figure S70. DSC curve (first cooling and second heating) of PCL-PpDL (Table 2, entry 3).

2.2.7.3. Simultaneous addition

Under an argon atmosphere in a glovebox, CL (63 mg, 0.55 mmol) and pDL (132 mg. 0.55 mmol) were dissolved in dry toluene (0.4 mL). In a separate vial, **LZn_2Et** (11 mg, 13.9 µmol) was dissolved in dry toluene (0.1 mL) and BnOH (1.5 mg, 1.4 µL, 13.9 µmol) from a stock solution (0.5 µM in toluene) was added. The two solutions were stirred separately for 30 min at 70 °C and then combined by adding the pDL and CL solution to the catalyst solution. Aliquots were taken at appropriate times to monitor the reaction until termination by quenching with $CDCl_3$. The product was obtained as a white powder by precipitation in cold, acidified methanol.

^{1}H NMR (500 MHz, $CDCl_3$) δ 4.15-4.07(m, 2H·n, -CH_2O-), 2.32-2.30 (m, 2H·n, -CO$CH_2$$CH_2$), 1.67-1.63 (m, 6H·n + 6H·m, -$COCH_2CH_2$-, -CH_2CHO-, -CH(CH_3)O-), 1,34-1,27 (m, 6H·n, -$CH_2CH_2CH_2$-).

^{13}C NMR (126 MHz, $CDCl_3$) δ 174.0, 173.9, 173.6, 173.5, 64.5, 64.4, 64.1, 64.0, 34.4, 34.3, 34.2, 34.1, 29.7, 29.6, 29.5, 29.4, 29.3, 29.2, 28.7, 28.4 28.3, 26.0, 25.6, 25.0, 24.6, 24.5 (note that there are multiple resonances due to the methylene groups of the PpDL block and heterojunctions between pDL and CL units).

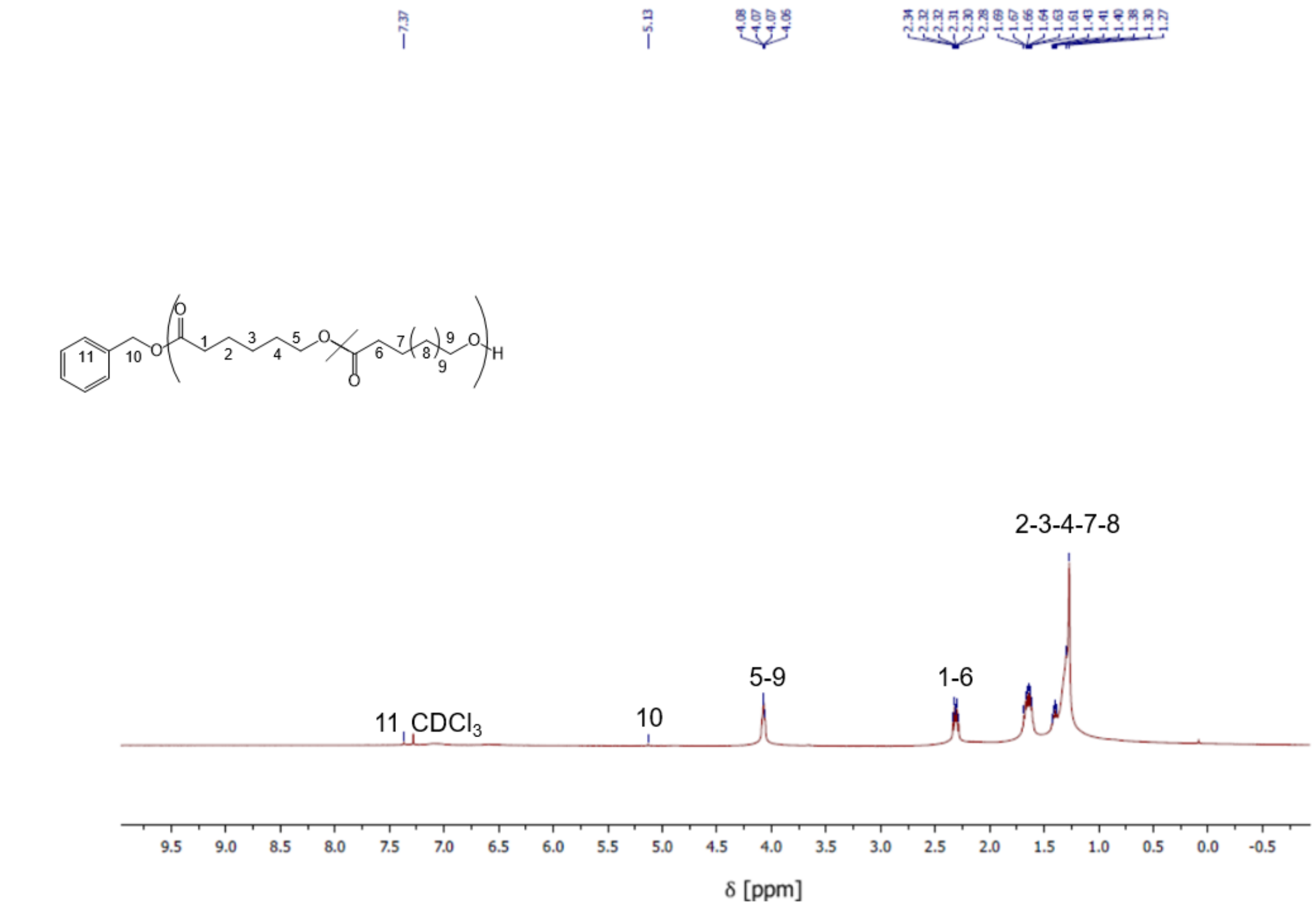

Figure S7. ¹H NMR spectrum (500 MHz, CDCl₃) of PCL₄₀-PpDL₄₀ (Table 2, entry 4).

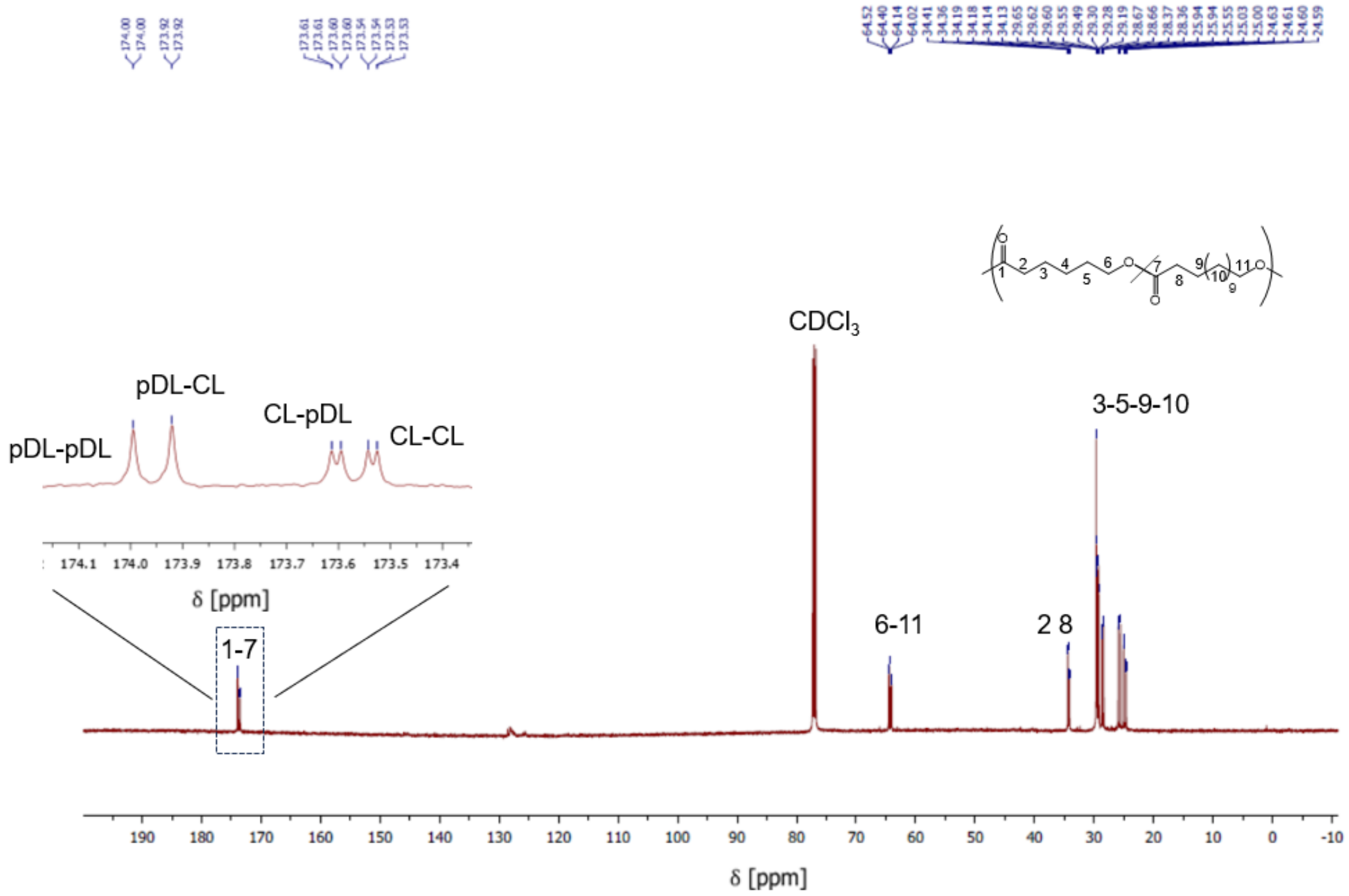

Figure S8. Quantitative ¹³C NMR spectrum (126 MHz, CDCl₃) of PCL₄₀-PpDL₄₀ (Table 2, entry 4).

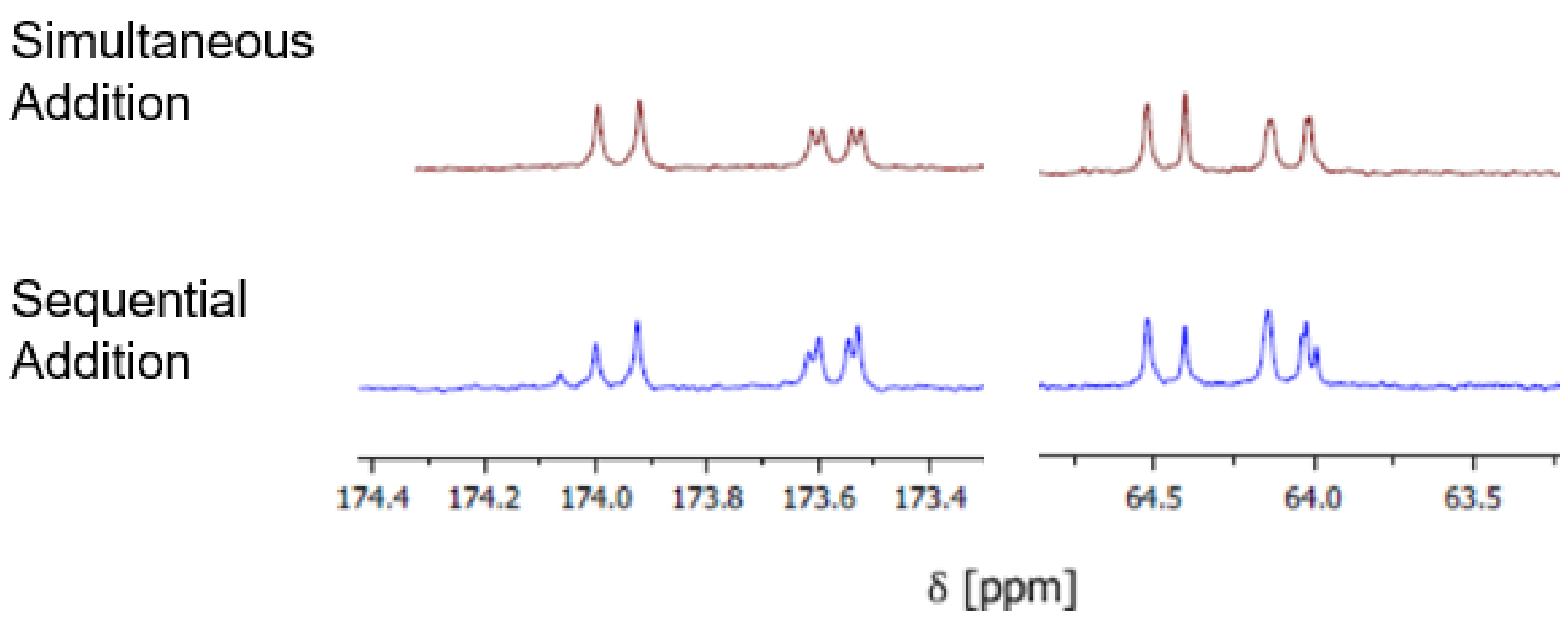

Figure S9. Comparison of quantitative ^{13}C NMR spectrum (126 MHz, $CDCl_3$) of PCL-PpDL copolymers, obtained through simultaneous (top) and sequential addition by PCL first (bottom).*

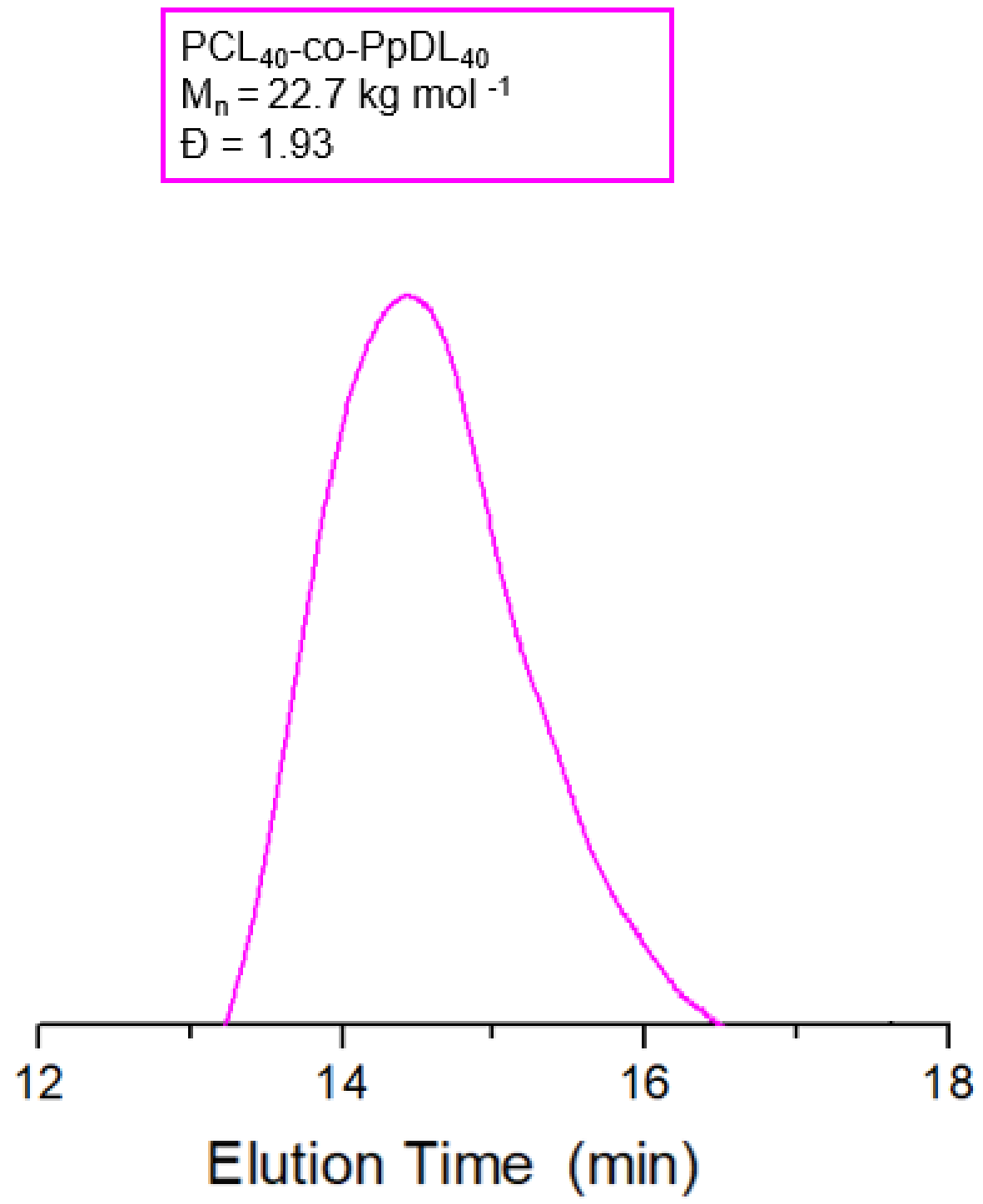

Figure S10. SEC traces of aliquots taken during the PCL_{40}-$PpDL_{40}$ copolymerisation (Table 2, entry 4).

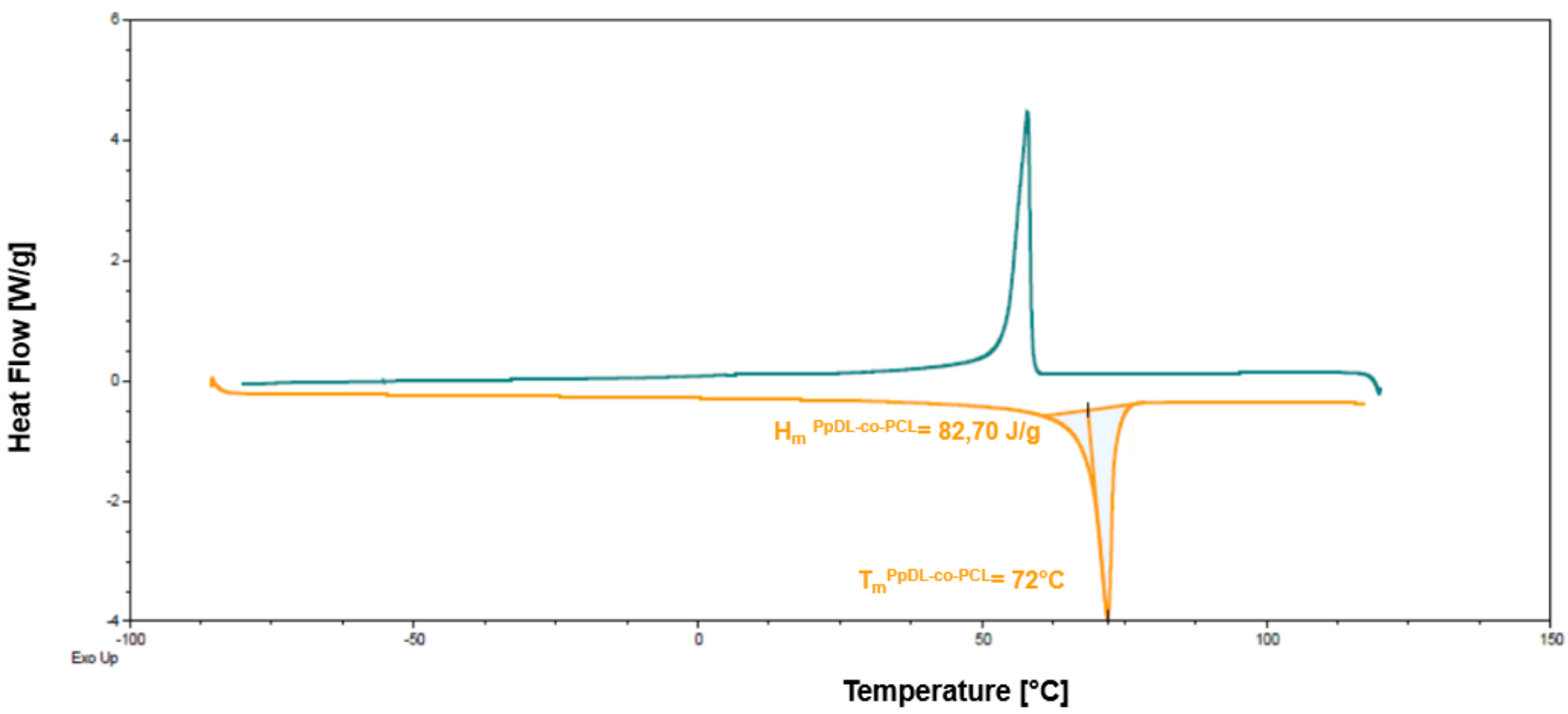

Figure S11. DSC curve (first cooling and second heating) of PpDL-co-PCL (Table 2, entry 4).

2.2.8. PpDL-DL diblocks

2.2.8.1. Sequential addition, pDL first

Under an argon atmosphere in a glovebox, pDL (165 mg, 0.69 mmol) was dissolved in dry toluene (0.6 mL). In a separate vial, **LZn_2Et** (11 mg, 13.9 µmol) was dissolved in dry toluene (0.1 mL) and BnOH (1.5 mg, 1.4 µL, 13.9 µmol) from a stock solution (0.5 µM in toluene) was added. The two solutions were stirred separately for 30 min at 70 °C and then combined by adding the pDL to the catalyst solution. An aliquot was taken and the solution was added to neat DL (146 mg, 0.69 mmol). Aliquots were taken at appropriate times to monitor the reaction until termination by quenching with $CDCl_3$. The product was obtained as a white solid by precipitation in cold, acidified methanol.

^{1}H NMR (500 MHz, $CDCl_3$) δ 4.88-4.86 (quint, 1H·m, -C*H*BuO-), 4.08-4.06 (t, 2H·n, -CH_2O-), 2.32-2.28 (m, 2H·n +2H·m , -COCH_2-), 1.65-1.63 (m, 2H·n + 2H·m, -COCH_2CH_2-), 1.35-1.27 (m, 18H·n, -CH_2CH_2CH_2-; m, 4H·n, -CH_2), 0.89-0.86 (t, 3H·n, -CH_2CH_3).

^{13}C NMR (126 MHz, $CDCl_3$) δ 174.0, 173.3, 73.9, 64.4, 34.5, 34.4, 29.7, 29.3, 29.2, 28.7, 27.5, 25.9, 25.0, 22.6, 14.0.

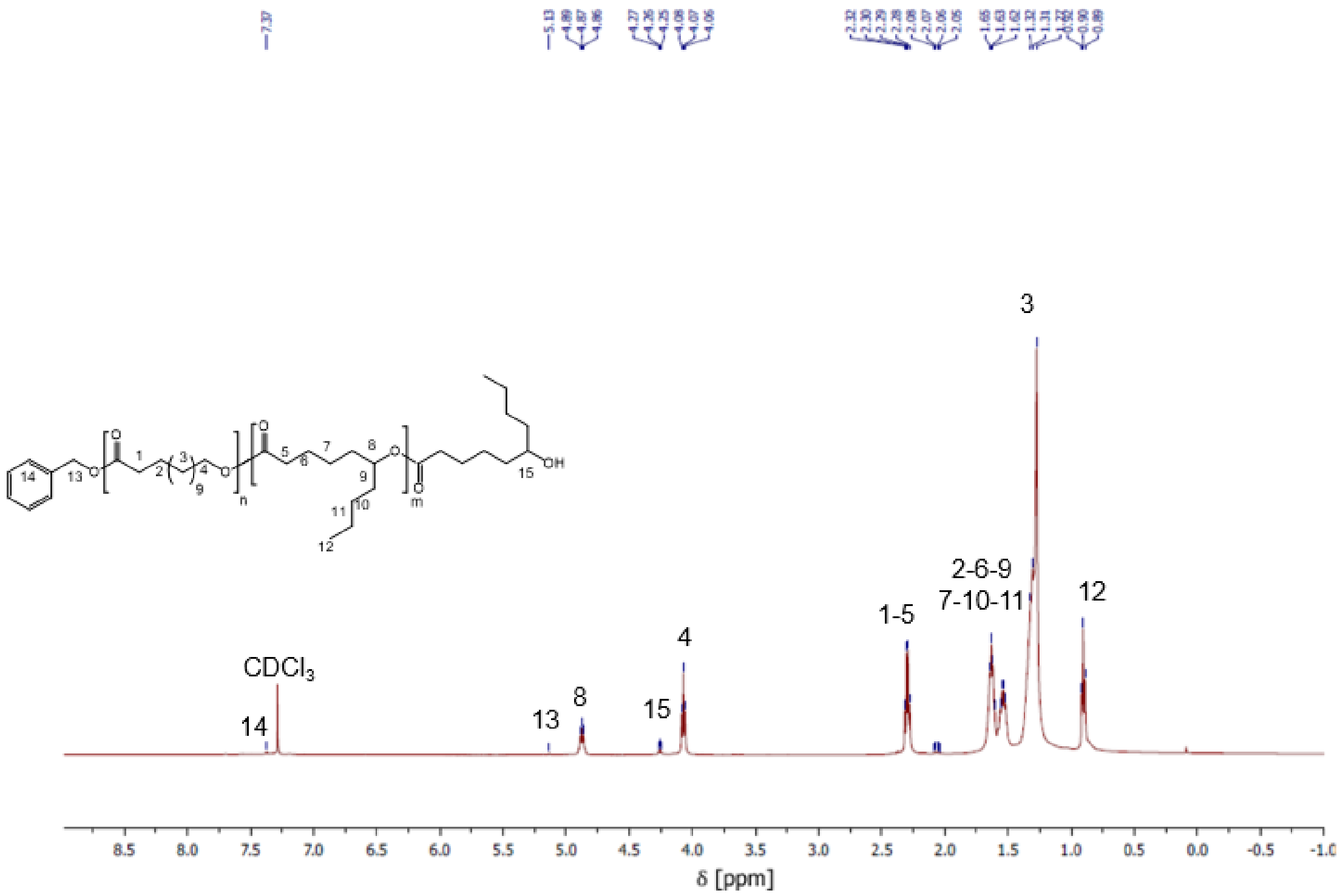

Figure S12. ^{1}H NMR spectrum (500 MHz, $CDCl_3$) of PpDL$_{40}$-PDL$_{40}$ (Table S5, entry 3).

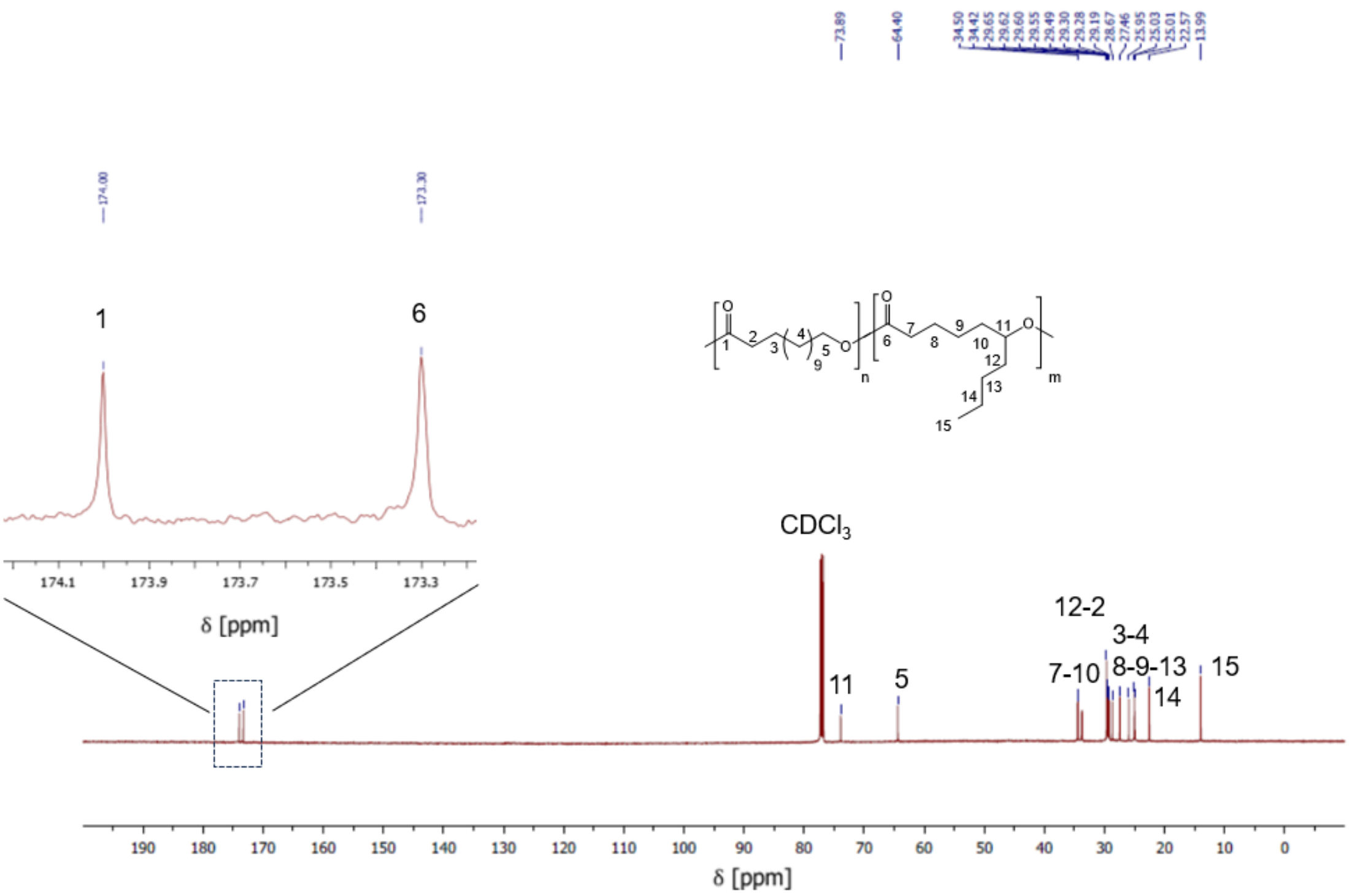

Figure S13. Quantitative ^{13}C NMR spectrum (126 MHz, $CDCl_3$) of PpDL$_{40}$-PDL$_{40}$ (Table S5, entry 3).

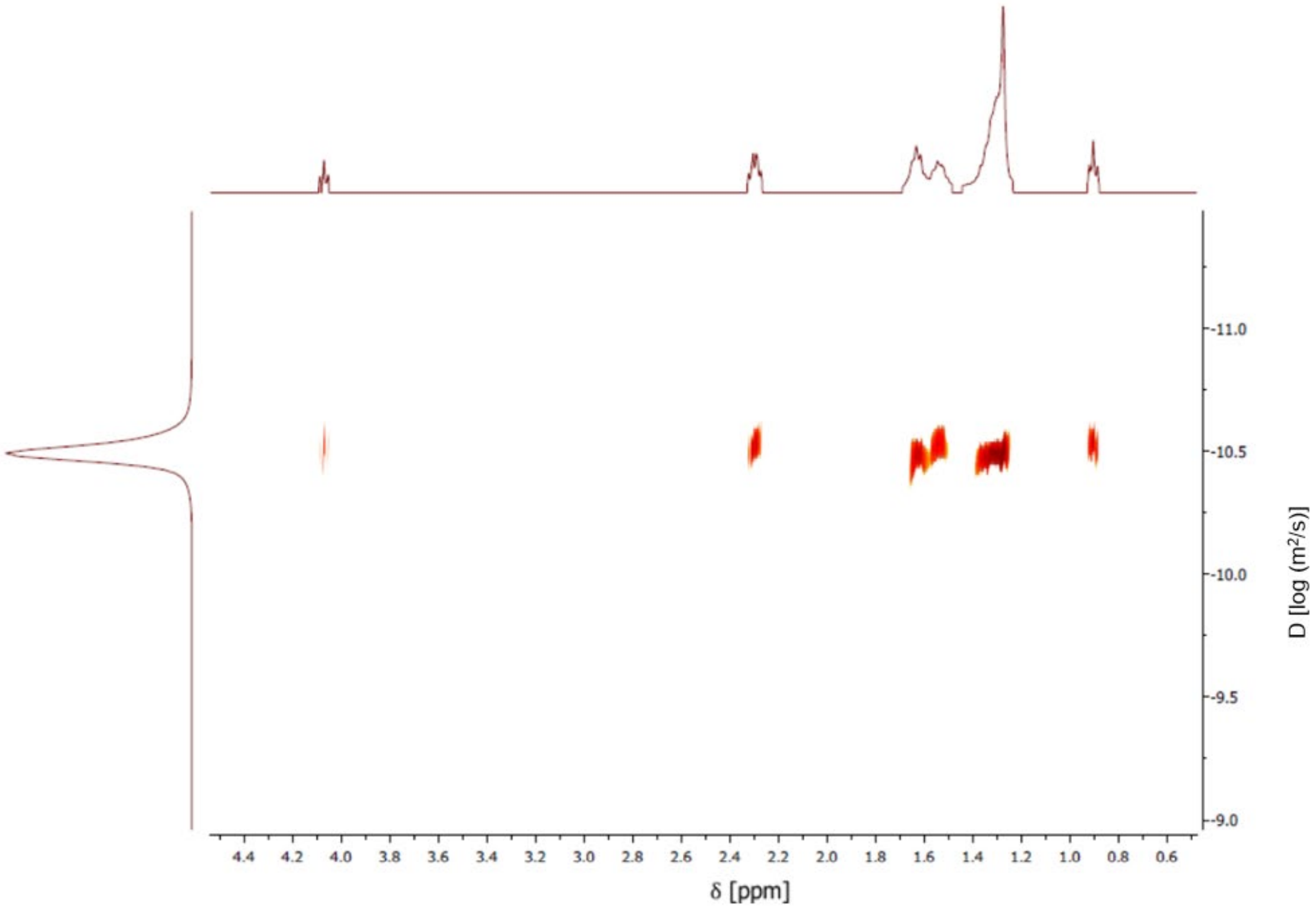

Figure S14. ^{1}H DOSY NMR spectrum (500 MHz, $CDCl_3$) of $PpDL_{40}$-PDL_{40} (Table S5, entry 3).

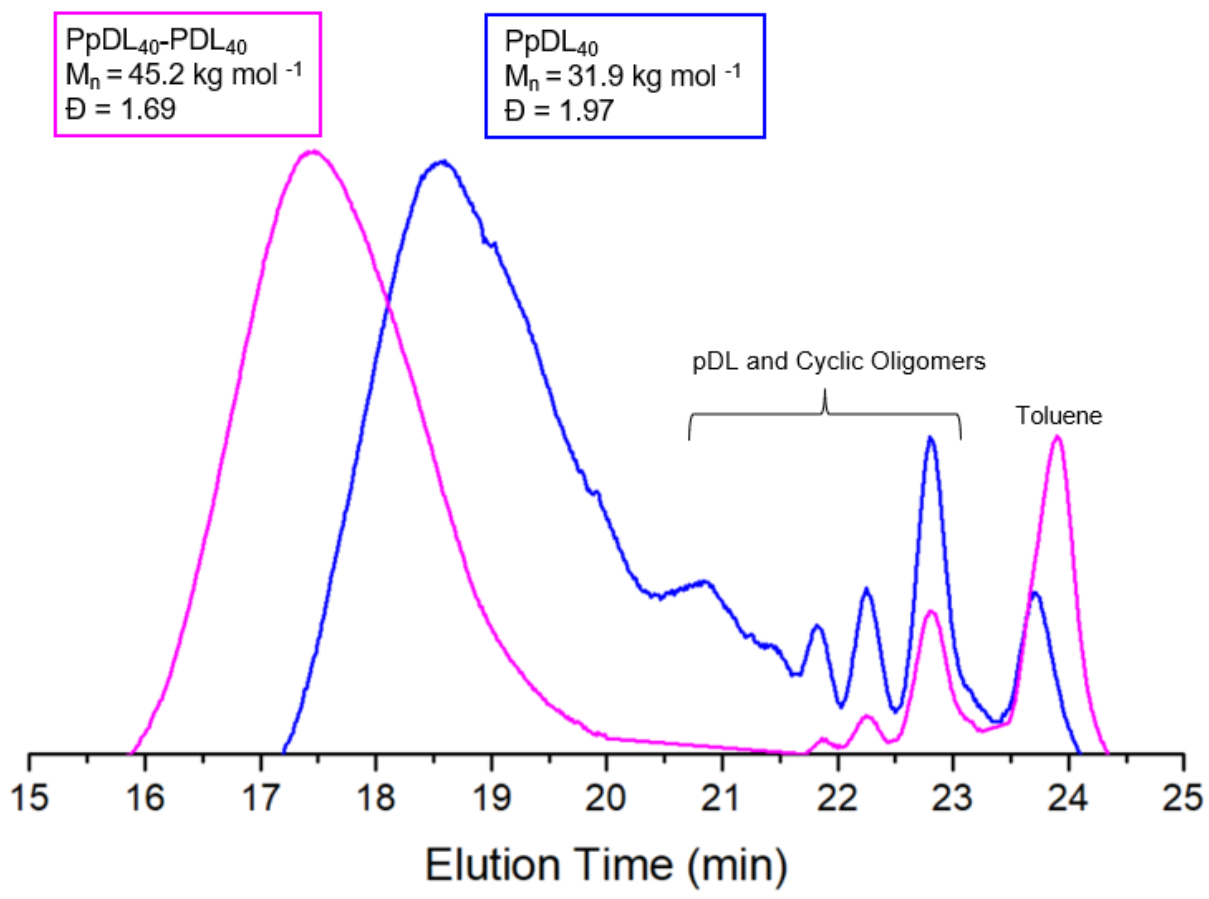

Figure S79. SEC traces of aliquots taken during the $PpDL_{40}$-PDL_{40} copolymerisation (Table S5, entry 3).

2.2.8.2. Sequential addition, DL first

When pDL was added to a propagating PDL* chain, no pDL propagation occurred, presumably due to the greater steric bulk and lower electrophilicity of DL preventing coordination and ring-opening.

2.2.8.3. Simultaneous addition

Under an argon atmosphere in glovebox, DL (130 mg, 0.55 mmol) and pDL (132 mg, 0.55 mmol) were dissolved in dry toluene (0.4mL). In a separate reaction vial, **LZn_2Et** (11 mg, 13.9 µmol) was dissolved in dry toluene (0.1 mL) and BnOH (1.5 mg, 1.4 µL, 13.9 µmol) from a stock solution (0.5 µM in toluene) was added. The two solutions were stirred separately for 30 min at 70 °C and then combined by adding the monomers solution to the catalyst solution. Aliquots were taken at appropriate times to monitor the reaction until termination by quenching with $CDCl_3$. The product was obtained as a white powder by precipitation in cold, acidified methanol.

^{1}H NMR (500 MHz, $CDCl_3$) δ 5.13 (s, 2H·m, -C*H*(CH_3)O), 4.88-4.86 (quint, 1H·n, -C*H*BuO-), 4.08-4.06 (t, 2H·m, -C*H_2*O-), 2.32-2.28 (m, 2H·n + 2H·m , -COC*H_2*-), 1.65-1.63 (m, 2H·n + 2H·m, -COCH_2C*H_2*), 1.55-1.53 (m, 4H·n -C*H_2*CH-C*H_2*-), 1.35-1.27 (m, 6H·n + 18H·m, -CH_2C*H_2*CH_2-; m, 2H·n, -C*H_2*), 0.89-0.86 (t, 3H·n, -CH_2C*H_3*).

^{13}C NMR (126 MHz, $CDCl_3$) δ 174.0, 173.3, 73.9, 64.4, 34.5, 34.4, 29.7, 29.3, 29.2, 28.7, 27.5, 26.0, 25.0, 22.6, 14.0.

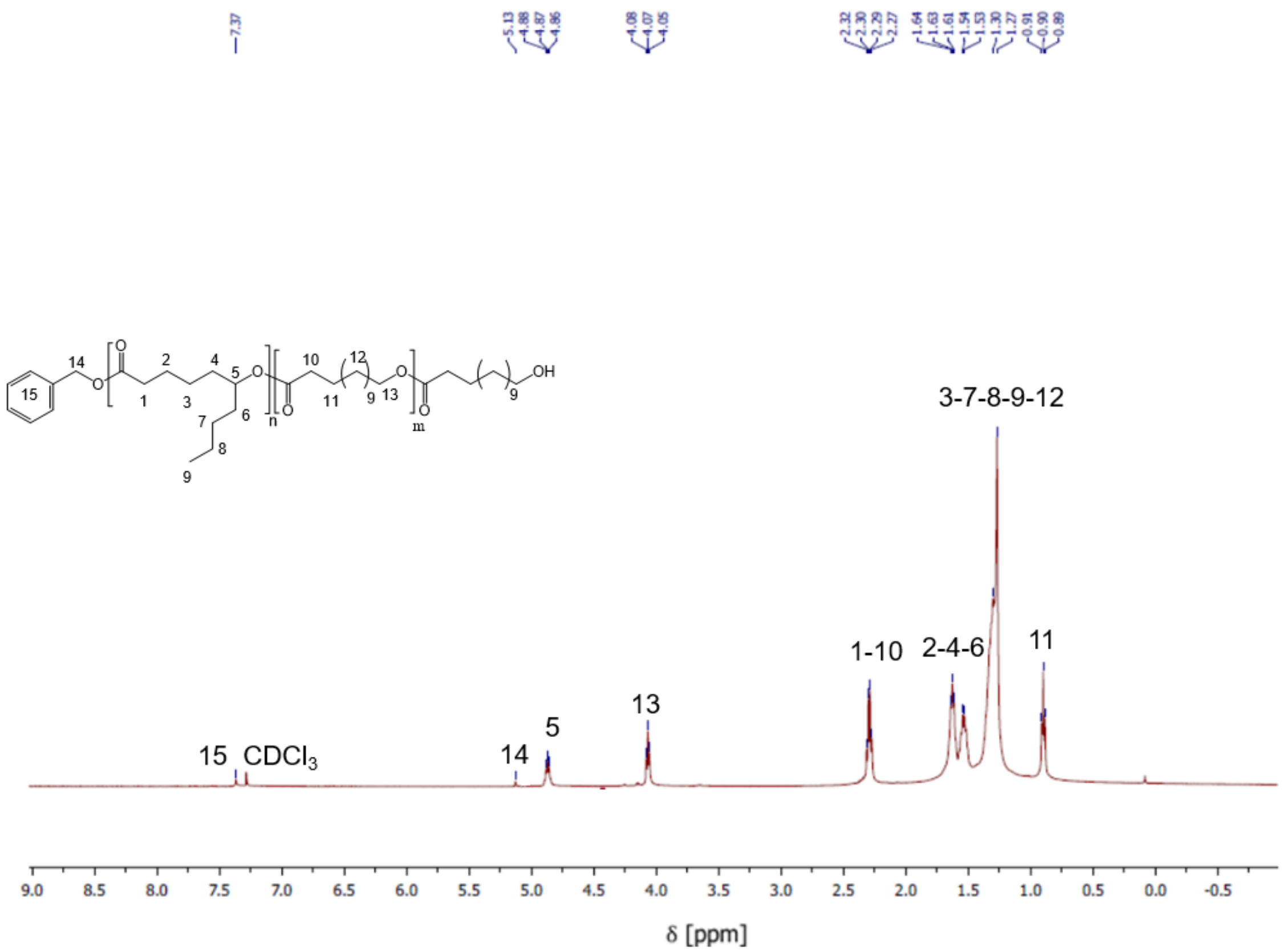

Figure S15. ^{1}H NMR spectrum (500 MHz, $CDCl_3$) of PDL_{40}-$PpDL_{40}$ (Table S5, entry 7).

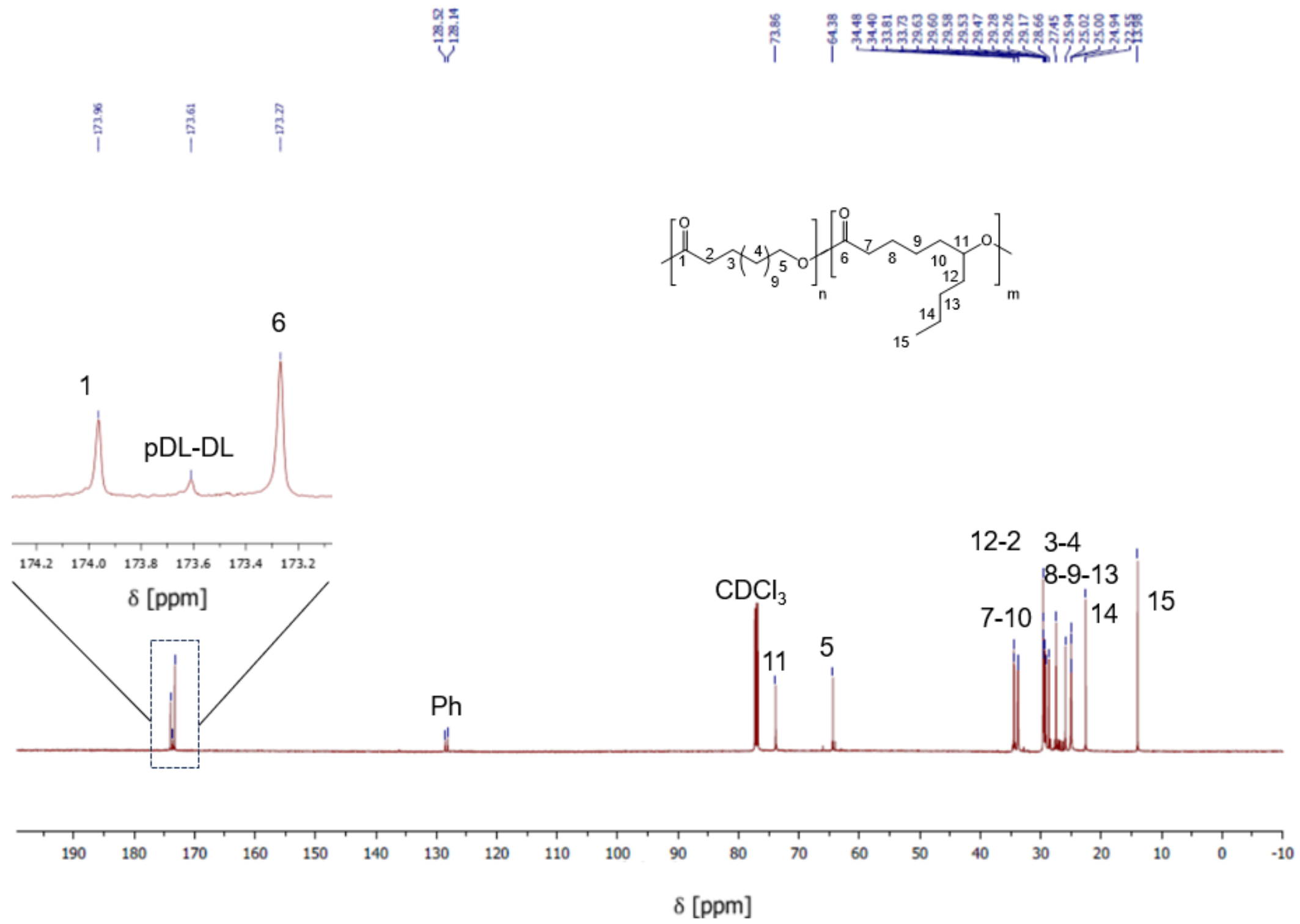

Figure S16. Quantitative ^{13}C NMR spectrum (126 MHz, CDCl3) of PDL40-PpDL40 (Table S5, entry 7).

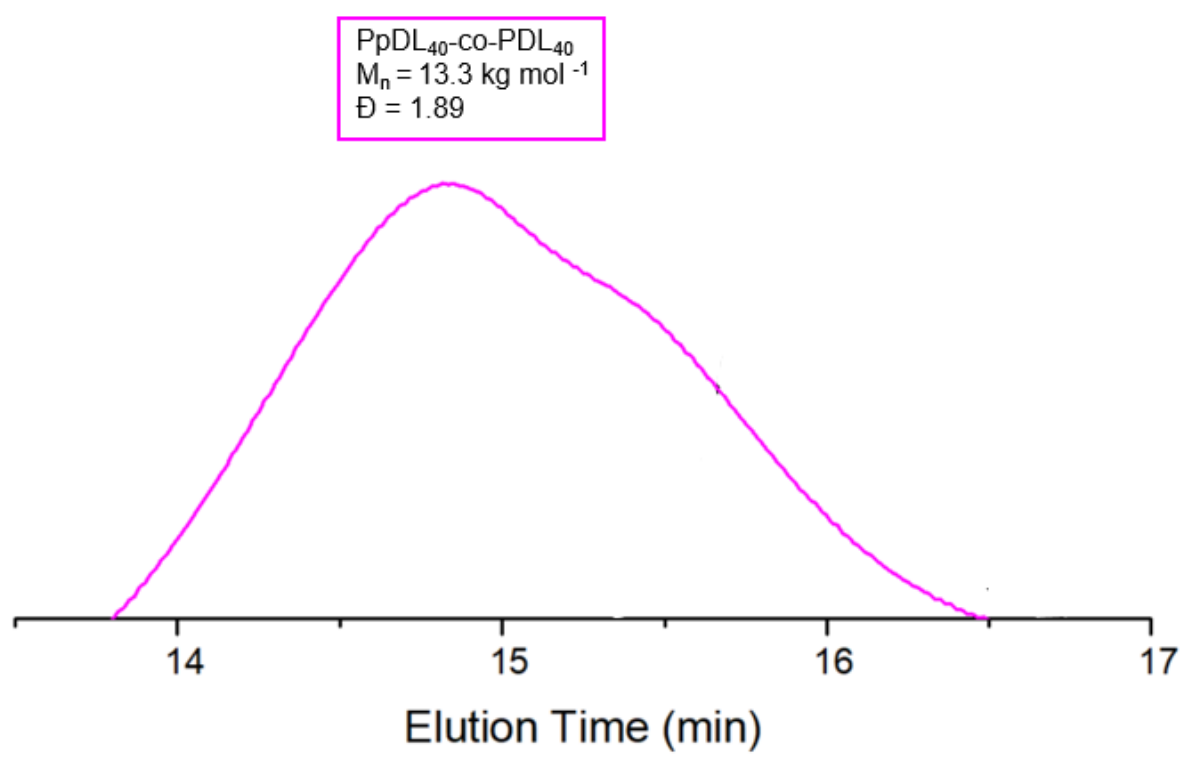

Figure S82. SEC traces of aliquots taken during the PDL40-PpDL40 copolymerisation(Table S5, entry 7).

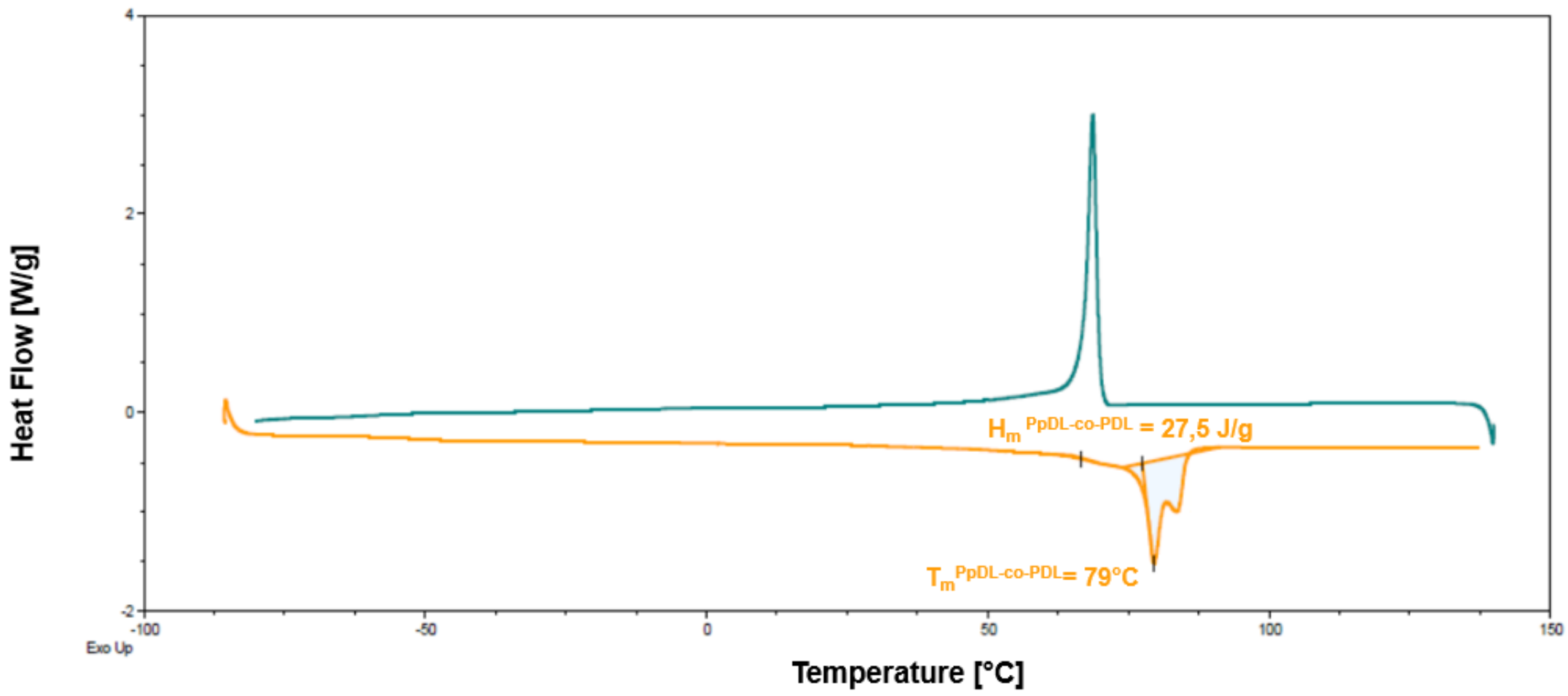

Figure S83. DSC curve (first cooling, second heating) of PpDL-co-PDL prepared via simultaneous monomer addition (Table S5, entry 7).

Table S5. Overview of data for pDL-CL/DL copolymerisation.

Entry	M_1 (eq.)	M_2 (eq.)	Molar Comp. (M_1:M_2)	$M_{n,theo}$[c] (kDa)	$M_{n,obs}$[d] (kDa)	$Đ$[d]	T_m (°C)	T_c (°C)
1[a]	pDL (40)	CL (40)	44:56	17.2	20.0	2.0	55 90	40 76
2[a]	pDL (40)	CL (80)	20:80	20.6	26.0	3.3	58 90	38 72
3[a]	pDL (40)	DL (40)	50:50	12.2	22.7	1.7	-	-
4[a]	CL (40)	pDL (40)	67:37	10.4	42.0	1.7	74	60
5[a]	DL (40)	pDL (40)	100:0	-	-	-	-	-
6[b]	pDL and CL (40)		42:58	15.9	13.3	1.9	72	58
7[b]	pDL and DL (40)		50:50	12.2	22.7	1.9	79	69

[a] Sequential monomer addition.
[b] Simultaneous monomer addition.
[c] Calculated from conversion $M_{n,}^{theo}$ conversion M_1 × MM_1 × ($[M_1]_0$/[**LZn₂OBn**]) + conversion M_2 x MM_2 × ($[M_2]_0$[**LZn₂OBn**]).
[d] obtained by SEC in THF using a PS standard.

2.3. Sequential copolymerisation, heteroatom containing monomers

2.3.1. PLA-P*p*DO diblocks, LA first

Under an argon atmosphere in a glovebox, *rac*-LA (40 mg, 0.3 mmol) was dissolved in dry toluene (0.3 mL). Additionally, **LZn_2Et** (11 mg, 13.9 µmol) was dissolved in dry toluene (0.1 mL) and BnOH (1.5 mg, 1.4 µL, 13.9 µmol) from a stock solution (0.5 µM in toluene) was added. The two solutions were stirred separately for 30 min at 70 °C and then combined by adding *rac*-LA solution to the catalyst solution. An aliquot was taken and the poly(lactic acid) solution was added to a *p*DO solution (27 mg, 0.3 mmol in 0.3 mL of dry toluene). Aliquots were taken at appropriate times to monitor the reaction until termination by quenching with $CDCl_3$. The product was obtained as a white solid by precipitation in cold, acidified methanol.

^{1}H NMR (500 MHz, $CDCl_3$) δ 5.22-5.14 (m, 1H·n + 2H, -CO*CH*CH_3 + PhC*H_2*O-), 4.39-4.36 (t, 2H·m, -OCH_2*CH_2*OCH_2CO-), 4.19 (s, 2H·m, -OCH_2CH_2O*CH_2*CO-), 3.82-3.80 (t, 2H·n, -O*CH_2*CH_2OCH_2CO-), 1.69-1.55 (d, 3H·n, - COCH*CH_3*).

^{13}C NMR (126 MHz, $CDCl_3$) δ 173.9, 170.5, 129.0, 128.6, 69.7, 68.6, 64.6, 64.2, 34.6, 28.8, 26.0, 25.00.

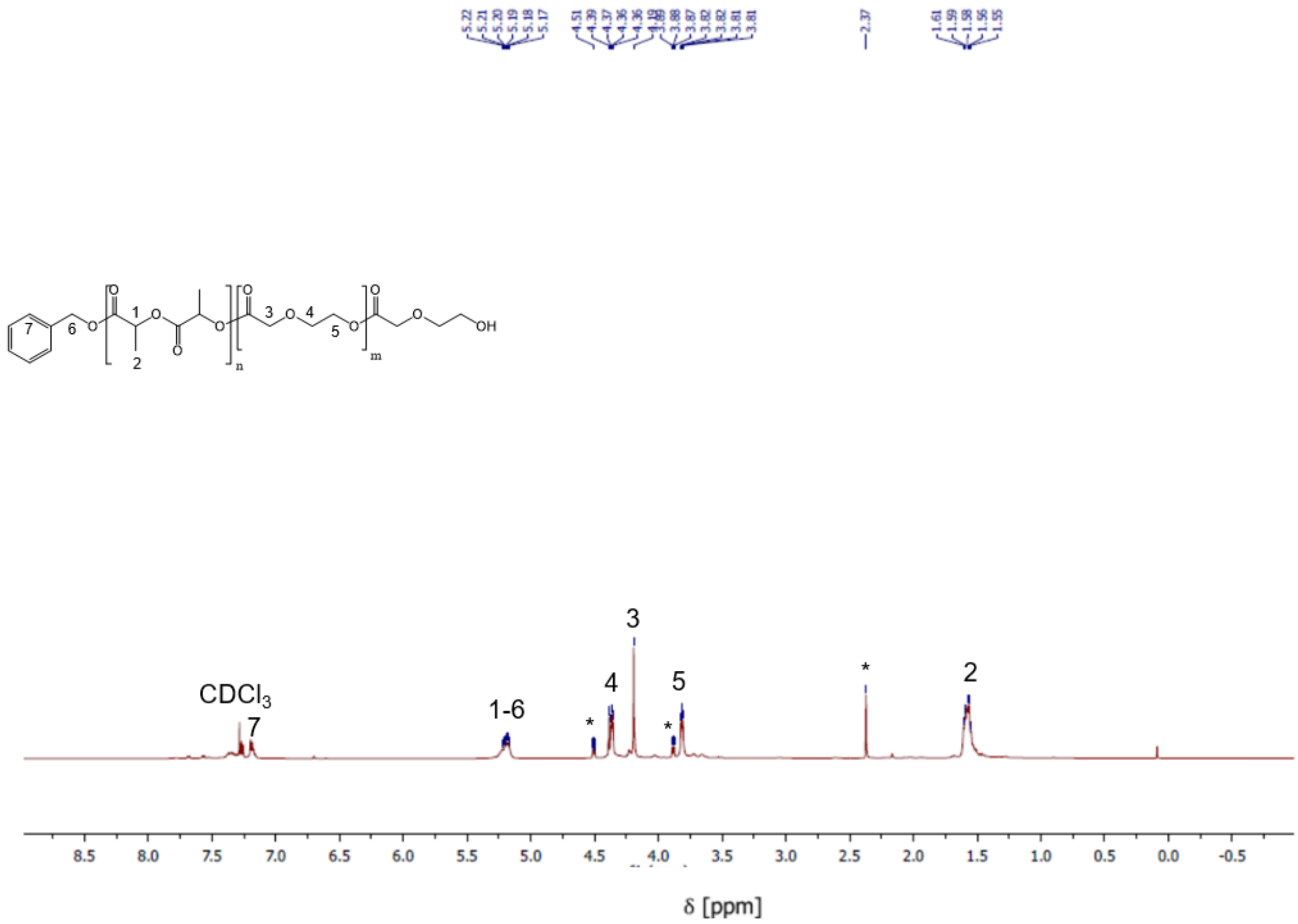

Figure S17. ^{1}H NMR spectrum (500 MHz, $CDCl_3$) of PLA_{20}-$PpDO_{20}$ (indicates pDO unreacted) (Table 3, entry 2).*

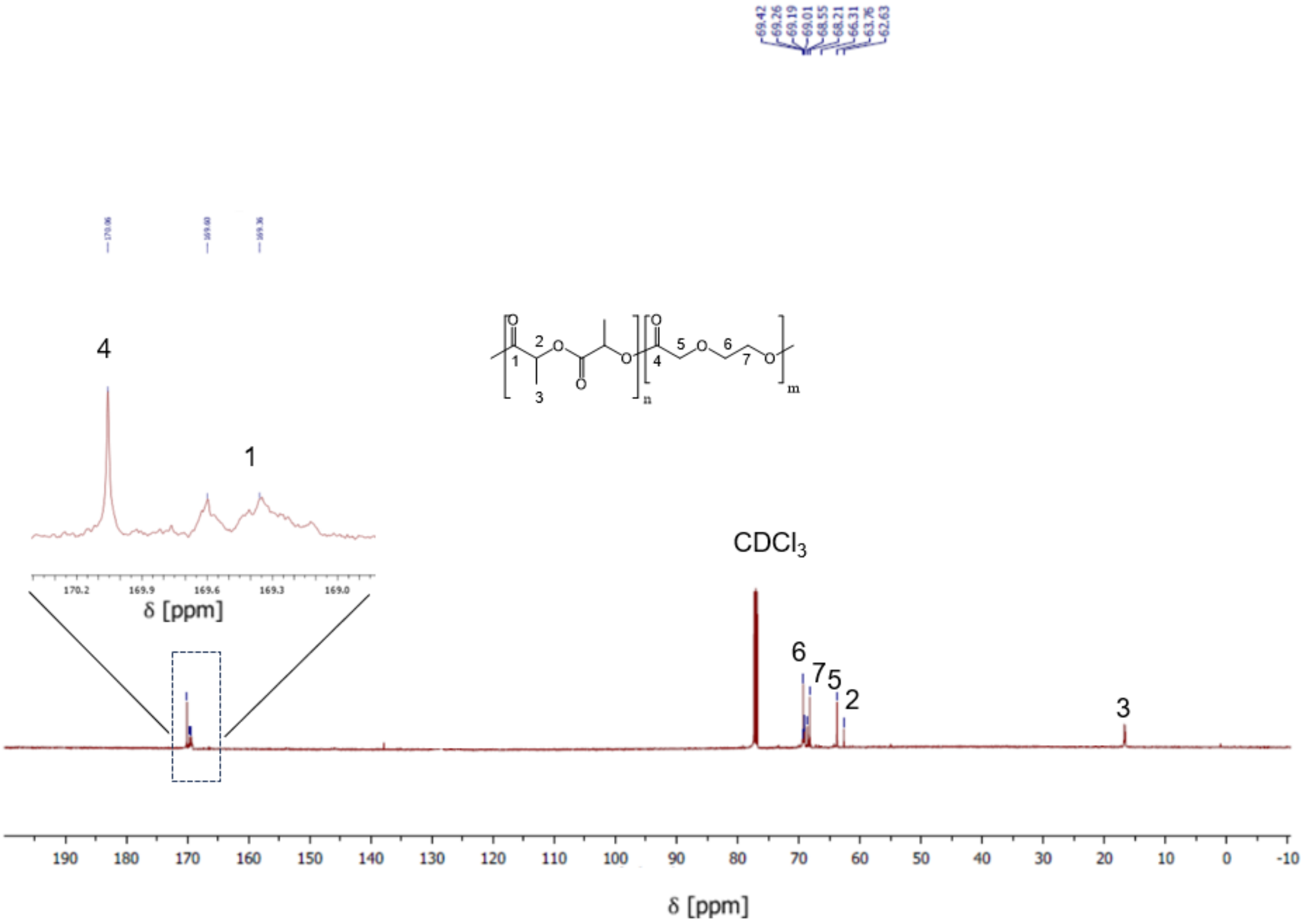

Figure S18. Quantitative ^{13}C NMR spectrum (126 MHz, $CDCl_3$) of PLA_{20}-$PpDO_{20}$ (Table 3, entry 2).

The average sequence lengths, l_e, of LA and pDO blocks can be determined from proton spectra using the following equations:[8]

$$l_e^{pDO} = \frac{i_{pDO-pDO}}{i_{LA-pDO}} + 1 \qquad \text{(S23)}$$

$$l_e^{LA} = \frac{l_e^{pDO}}{k} \qquad \text{(S24)}$$

where $i_{pDO-pDO}$ and i_{LA-pDO} represent the resonances of methylene groups in the dioxanyl unit of the pDO–*pDO** sequence (4.14-4.18 ppm) and of sequence LA-*p*DO*, respectively (4.19-4.22 ppm) calculated from ^1H NMR, and *k* represents the actual *p*DO/LA molar ratio in the copolymer determined by ^1H NMR.

The theoretical lengths of LA and *p*DO blocks in completely random chains (l_R^{LA} and l_R^{pDO} respectively) can also be calculated from the following equations:

$$l_R^{pDO} = k + 1 \qquad \text{(S25)}$$

$$l_R^{LA} = \frac{k+1}{k} \qquad \text{(S26)}$$

Copolymer randomness was evaluated by determining the sequence distribution in each copolymer. Therefore, the degree of randomness of the copolymer chains can be calculated from the equation below:

$$R = \frac{l_R^{pDO}}{l_e^{pDO}} = \frac{l_R^{LA}}{l_e^{LA}} \qquad \text{(S27)}$$

2.3.2. PCL-P*p*DO diblocks, CL first

Under an argon atmosphere in a glovebox, CL (30 mg, 0.3 mmol) was dissolved in dry toluene (0.3 mL). In a separate reaction vial, **LZn_2Et** (11 mg, 13.9 μmol) was dissolved in dry toluene (0.1 mL) and BnOH (1.5 mg, 1.4 μL, 13.9 μmol) from a stock solution (0.5 μM in toluene) was added. The two solutions were stirred separately for 30 min at 70 °C and then combined by adding the CL to the catalyst solution. An aliquot was taken and the solution was added to a *p*DO solution (27 mg, 0.3 mmol) in 0.3 mL of dry toluene, previously stirred at 70 °C. Aliquots were taken at appropriate times to monitor the reaction until termination by quenching with $CDCl_3$. The product was obtained as a white solid by precipitation in cold, acidified methanol.

^{1}H NMR (500 MHz, $CDCl_3$) δ, 4.38-4.36 (t, 2H·n, $-OCH_2CH_2OCH_2CO-$), 4.20 (s, 2H·n $-OCH_2CH_2OCH_2CO-$), 4.10-4.07 (t, 2H·n, $-CO(CH_2)_4CH_2O-$), 3.83-3.81 (t, 2H·n, $-OCH_2CH_2OCH_2CO-$), 2.34-2.31 (t, 2H·n, $-COCH_2(CH_2)_4O-$), 1.69-1.64 (m, 4H, $-COCH_2CH_2-$), 1.42-1.39 (m, 2H·n, $-CH_2CH_2CH_2-$).

^{13}C NMR (126 MHz, $CDCl_3$) δ 173.9, 170.5, 129.0, 128.6, 69.7, 68.6, 64.6, 64.2, 34.6, 28.8, 26.0, 25.00.

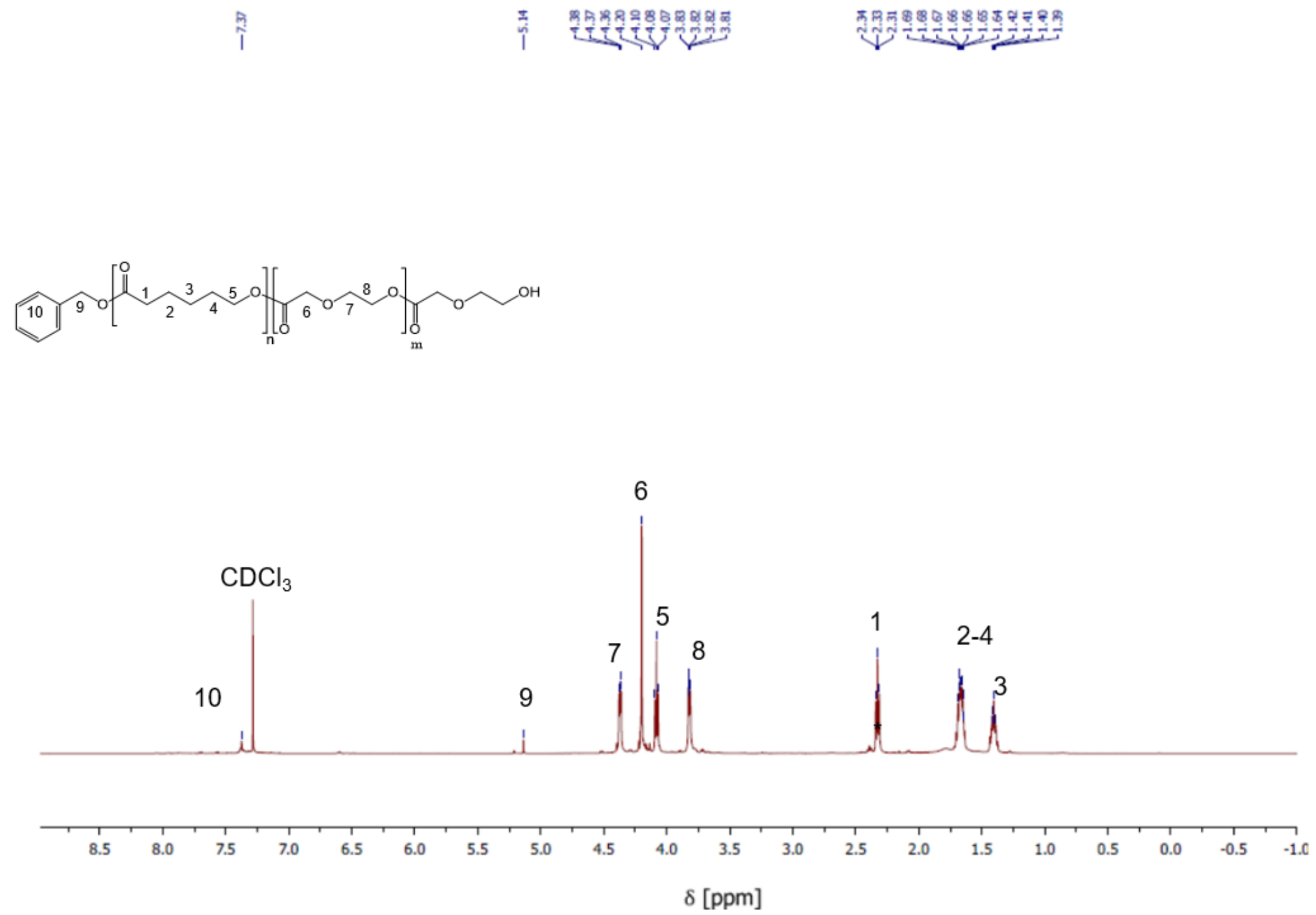

*Figure S19. ^{1}H NMR spectrum (500 MHz, $CDCl_3$) of PCL_{20}-P*p*DO_{20} (Table 3, entry 6).*

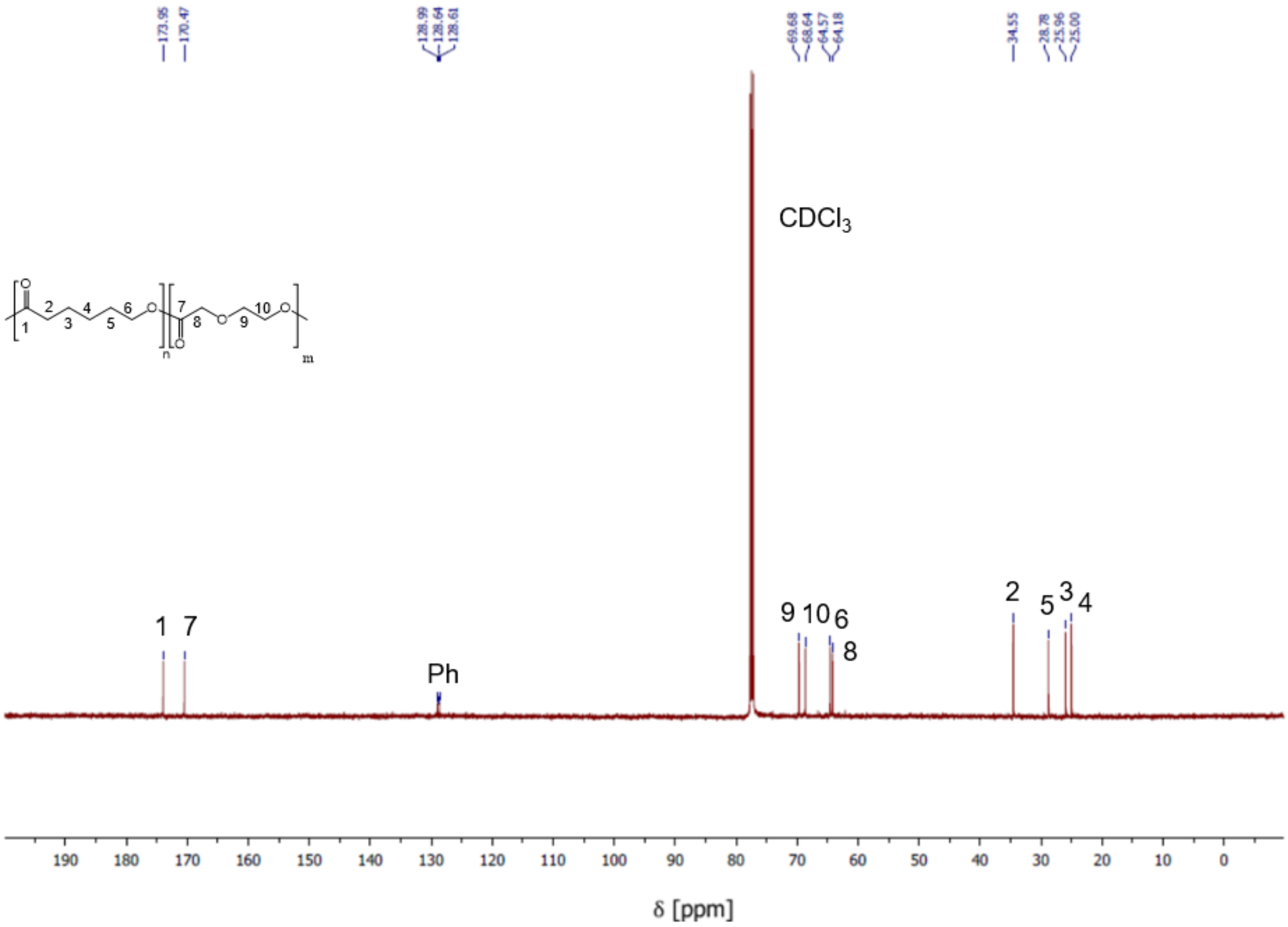

Figure S20. Quantitative ^{13}C NMR spectrum (126 MHz, $CDCl_3$) of PCL_{20}-$PpDO_{20}$ (Table 3, entry 6).

By comparison of peak intensity representing the resonances of the methylene groups in the dioxanyl unit *p*DO-*pDO** and CL-*pDO**, and the peak intensity representing the resonances of methylene groups in the caproyl unit CL-*CL** and *p*DO-*CL**, it is possible to calculate the experimental number-average lengths of CL and *p*DO blocks, l_e^{CL} and l_e^{pDO}, respectively, in the copolymers according to the following equations:[9]

$$l_e^{pDO} = \frac{i_{pDO-pDO*}}{i_{CL-pDO*}} + 1 \quad (S28)$$

$$l_e^{CL} = \frac{i_{CL-CL*}}{i_{CL-pDO*}} + 1 \quad (S29)$$

where $i_{pDO-pDO*}$ and $i_{CL-pDO*}$ indicate the peak intensity of *p*DO-*pDO** (4.35-4.40 ppm) and CL-*pDO** (4.25-4.30 ppm) sequences of the copolymer calculated from ^{1}H NMR spectroscopy, and i_{CL-CL*} and $i_{pDO-CL*}$ represent the peak intensity of CL-*CL** (2.25-2.33 ppm) and *p*DO-*CL** (2.33-2.39 ppm) sequences of the copolymer determined by ^{1}H NMR spectroscopy. Copolymer randomness was evaluated by determining the sequence distribution in each copolymer. Therefore, the degree of randomness (*R*) of the copolymer chains can be calculated from the equation below:

$$R = \frac{100}{l_R^{pDO}} + \frac{100}{l_e^{CL}} \quad (S30)$$

2.3.3. PLA-PTMC diblocks, LA first

Under an argon atmosphere in a glovebox, *rac*-LA (40 mg, 0.3 mmol) was dissolved in dry toluene (0.3 mL). In a separate reaction vial, **LZn_2Et** (11 mg, 13.9 µmol) was dissolved in dry toluene (0.1 mL) and BnOH (1.5 mg, 1.4 µL, 13.9 µmol) from a stock solution (0.5 µM in toluene) was added. The two solutions were stirred separately for 30 min at 70 °C and then

combined by adding the *rac*-LA solution to the catalyst solution. An aliquot was taken and the solution was added to a TMC solution (28 mg, 0.3 mmol) in 0.2 mL of dry toluene, previously stirred at 70°C. Aliquots were taken at appropriate times to monitor the reaction until termination by quenching with $CDCl_3$. The product was obtained as a white solid by precipitation in cold, acidified methanol.

^1H NMR (500 MHz, $CDCl_3$) δ 5.23-5.00 (m, 2H·n + 2H, -CO*CH*CH_3 + PhCH_2O-), 4.29-4.23 (t, 2H·m, -O$CH_2$$CH_2$$CH_2$O-), 2.07-2.04 (t, 2H·m, -O$CH_2$$CH_2$$CH_2$O-), 1.60-1.54 (m, 6H·n, -COCHCH_3).

^{13}C NMR (126 MHz, $CDCl_3$) δ 170.7, 155.0, 69.1, 64.4, 28.2, 17.0.

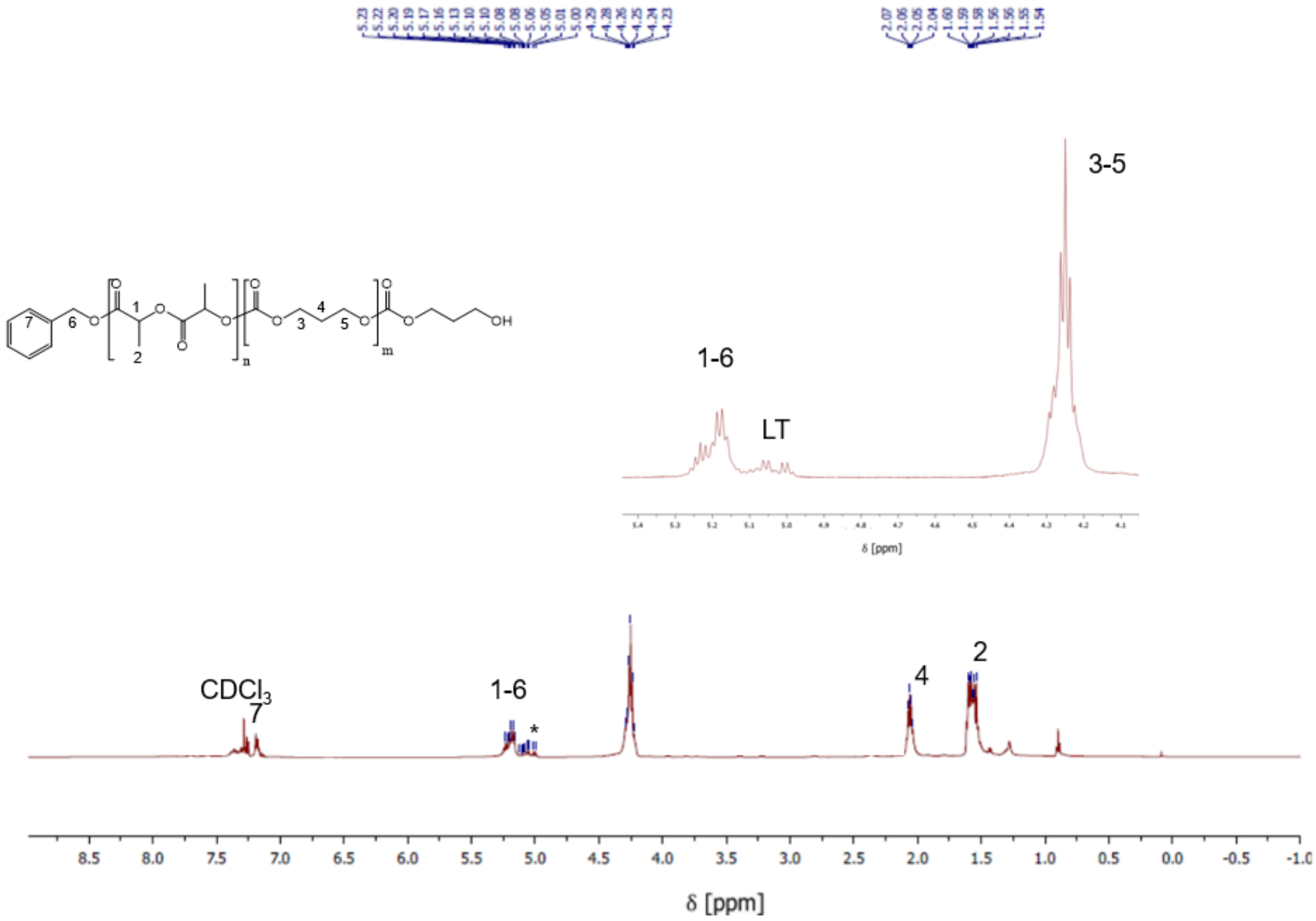

Figure S21. ^1H NMR spectrum (500 MHz, $CDCl_3$) of PLA_{20}-$PTMC_{20}$ (Table 3, entry 1).

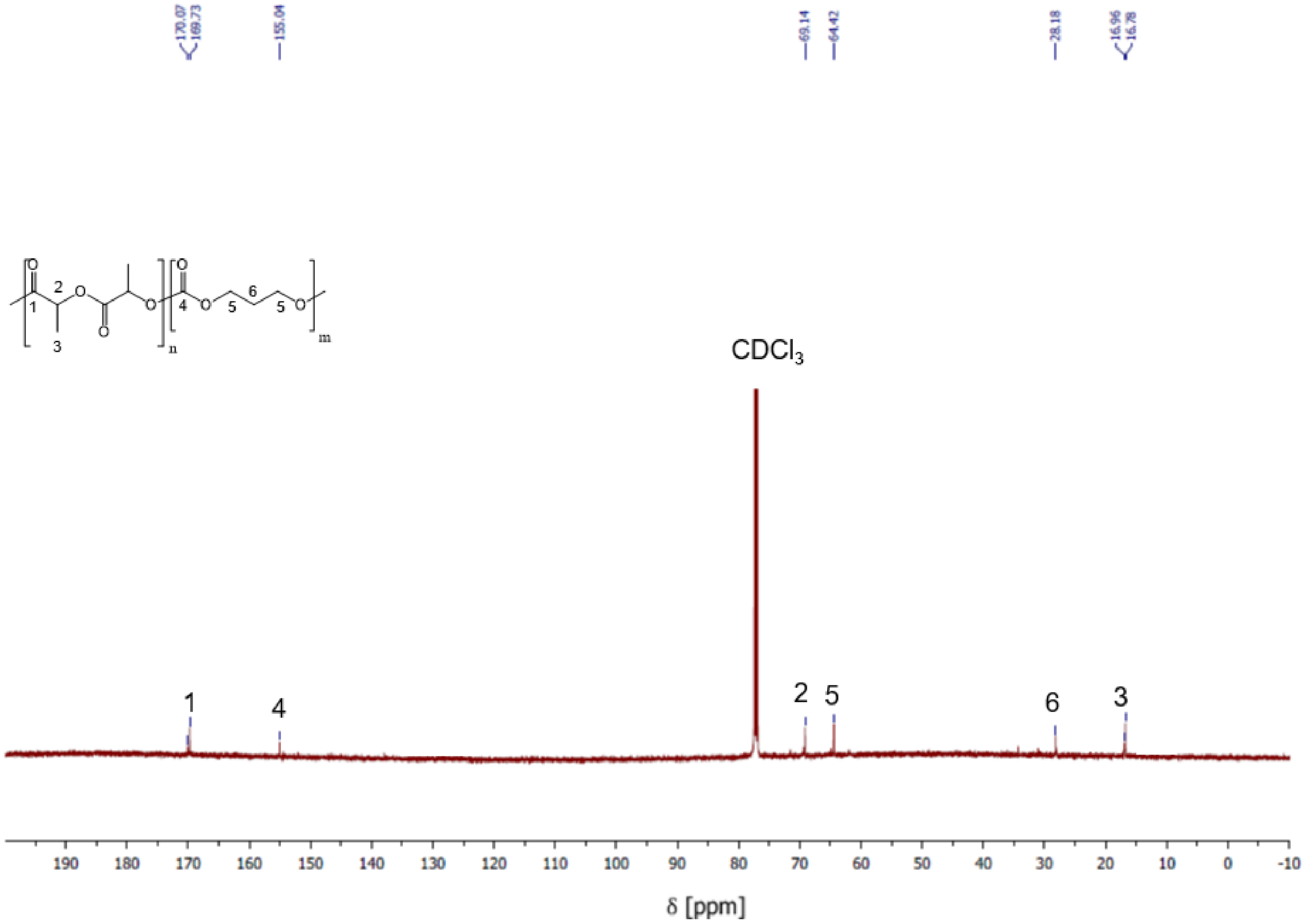

Figure S89. Quantitative ^{13}C NMR spectrum (126 MHz, $CDCl_3$) of PLA_{20}-$PTMC_{20}$ (Table 3, entry 1).

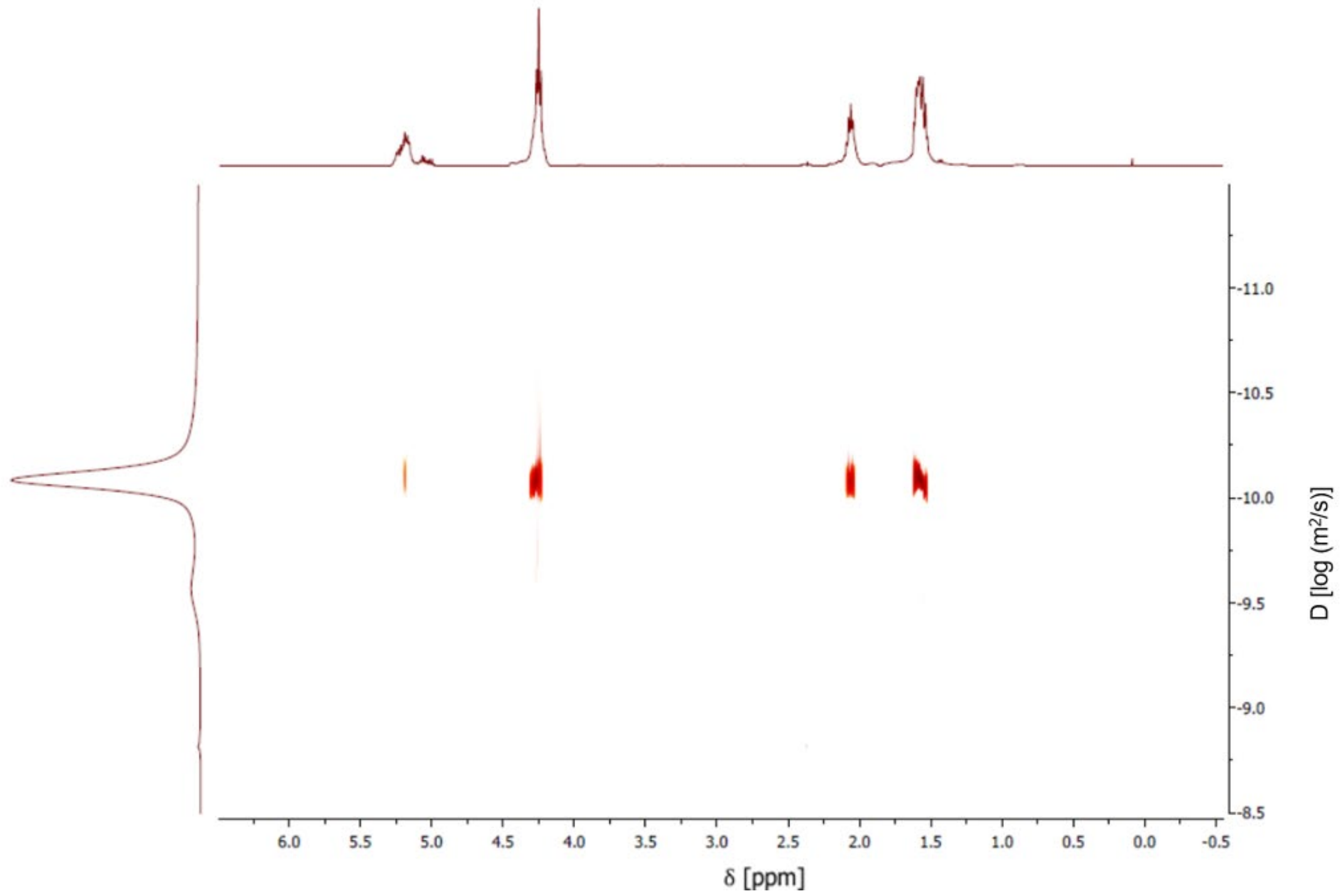

Figure S22. ^{1}H DOSY NMR spectrum (500 MHz, $CDCl_3$) of PLA_{20}-$PTMC_{20}$ (Table 3, entry 1).

The average lengths l_e of lactide and trimethylene carbonate blocks can be determined from proton spectra using the following relations:[10]

$$l_e^{LA} = \frac{i_{LA} + i_{LA-TMC}}{i_{LA-TMC}} \quad \text{(S31)}$$

$$l_e^{TMC} = l_e^{LA} \ \frac{1-F_{LA}}{F_{LA}} \quad \text{(S32)}$$

where i_{LA} and i_{LA-TMC} indicate the peak intensity of LA-LA* (5.13 ppm) and LA-TMC* (5.00 ppm) sequences of the copolymer and F_{LA} is the molar fraction of lactidyl units in the copolymer chain, calculated from ^{1}H NMR. The randomization ratio of copolymer chains (R) may be determined with the following equation:

$$R = \frac{l_R^{LA}}{l_e^{LA}} = \frac{l_R^{TMC}}{l_e^{TMC}} \quad \text{(S33)}$$

$$l_R^{LA} = \frac{k'+1}{2k'} \quad \text{(S34)}$$

$$l_R^{TMC} = k'+1 \quad \text{(S35)}$$

$$K' = \frac{1-F_L}{F_L} \quad \text{(S36)}$$

$$F_L = \frac{2F_{LA}}{F_{LA}+1} \quad \text{(S37)}$$

Where l_R^{LA} and l_R^{TMC} are the average lengths of lactidyl and TMC blocks, respectively, in chains with completely random contributions of carbonate and lactidyl units, while F_L is the molar fraction of lactyl units in the copolymer chain.

2.3.4. PCL-PTMC diblocks, CL first

Under an argon atmosphere in glovebox, CL (30 mg, 0.3 mmol) was dissolved in dry toluene (0.3 mL). In a separate reaction vial, **LZn_2Et** (11 mg, 13.9 μmol) was dissolved in dry toluene (0.1 mL) and BnOH (1.5 mg, 1.4 μL, 13.9 μmol) from a stock solution (0.5 μM in toluene) was added. The two solutions were stirred separately for 30 min at 70 °C and then combined by adding the CL solution to the catalyst solution. An aliquot was taken and the solution was added to a TMC solution (28 mg, 0.3 mmol) in 0.2 mL of dry toluene, previously stirred at 70°C. Aliquots were taken at appropriate times to monitor the reaction until termination by quenching with $CDCl_3$. The product was obtained as a white solid by precipitation in cold, acidified methanol.

^{1}H NMR (500 MHz, $CDCl_3$) δ 74.29-4.23 (t, 2H·m, -O*CH_2*CH_2*CH_2*O-), 4.08-4.05 (t, 2H·n, CH_2*CH_2*O-), , 2.32-2.29 (t, 2H·n, -CO*CH_2*), 1.91-1.63 (t, 4H·n, -$COCH_2$*CH_2*CH_2*CH_2*CH_2O-), 1.42-1.37 (t, 4H·n, -$COCH_2CH_2$*CH_2*CH_2CH_2O-).

^{13}C NMR (126 MHz, $CDCl_3$) δ 173.5, 154.9, 128.5, 128.2, 64.3, 34.1, 28.3, 28.0, 25.5, 24.6.

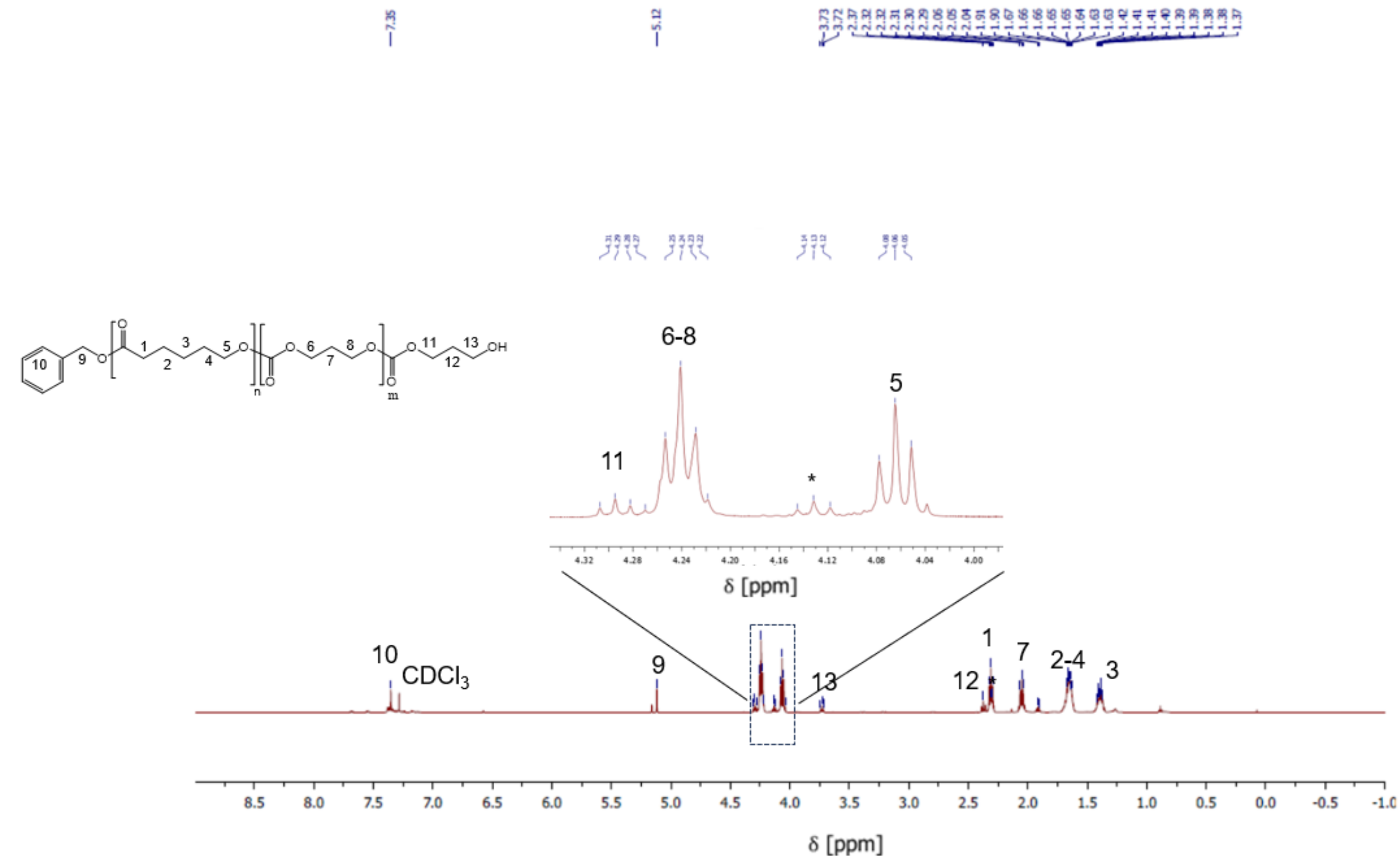

Figure S23. ^{1}H NMR spectrum (500 MHz, $CDCl_3$) of PCL_{20}-$PTMC_{20}$ (Table 3, entry 5).

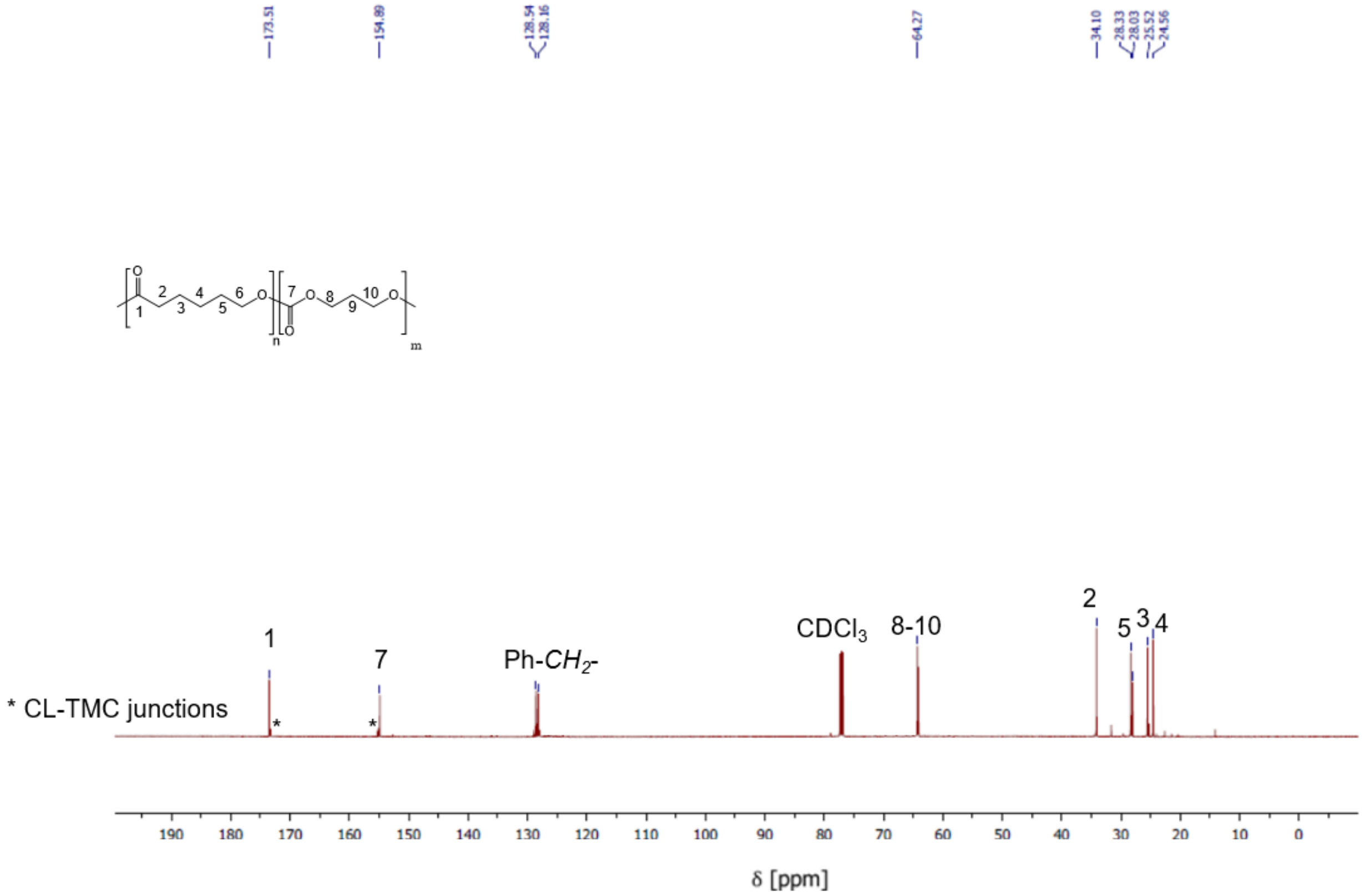

Figure S24. Quantitative ^{13}C NMR spectrum (126 MHz, $CDCl_3$) of PCL_{20}-$PTMC_{20}$ (Table 3, entry 5).

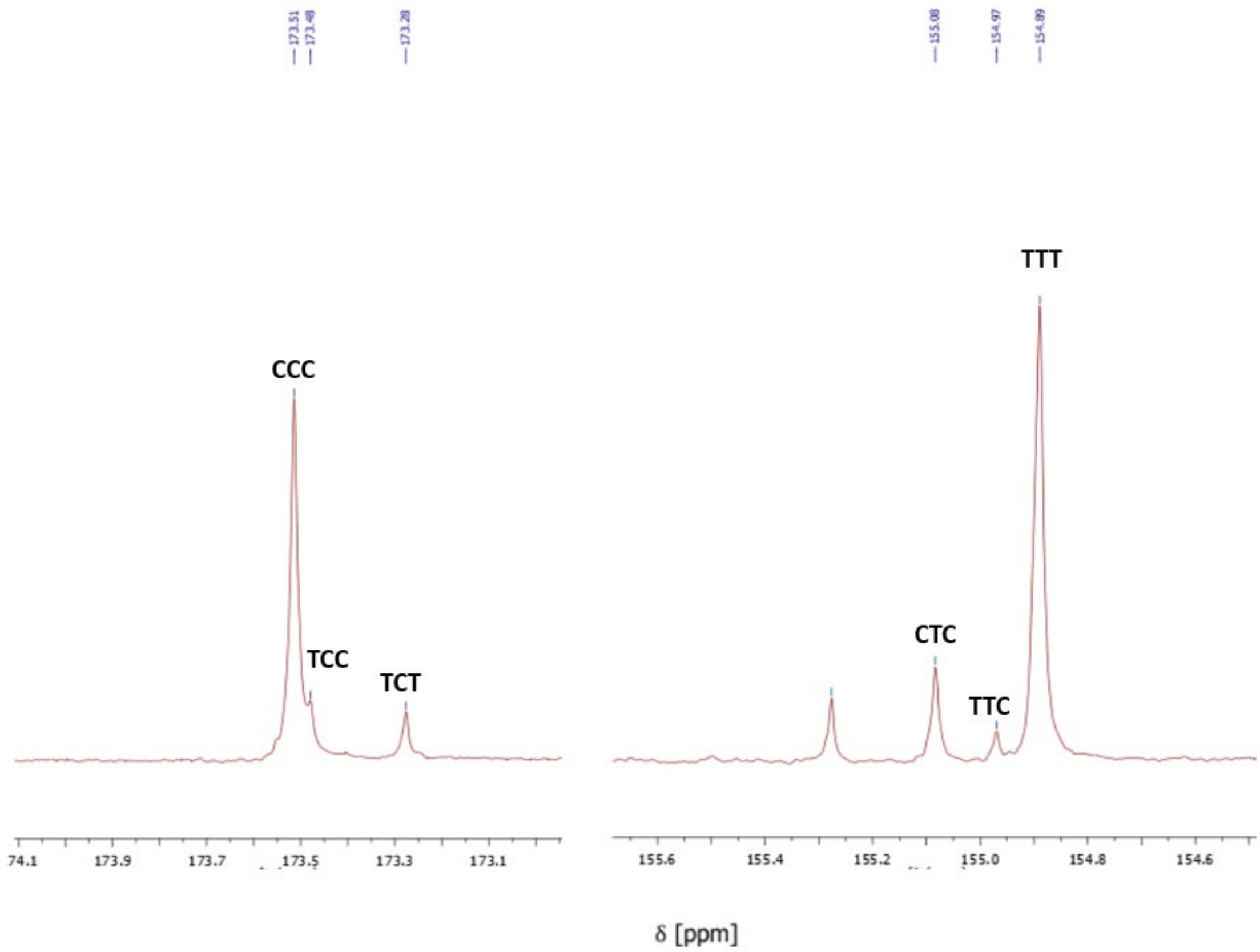

Figure S25. Enlargement of quantitative ^{13}C NMR spectrum (126 MHz, $CDCl_3$) of PCL_{20}-$PTMC_{20}$ (Table 3, entry 5).

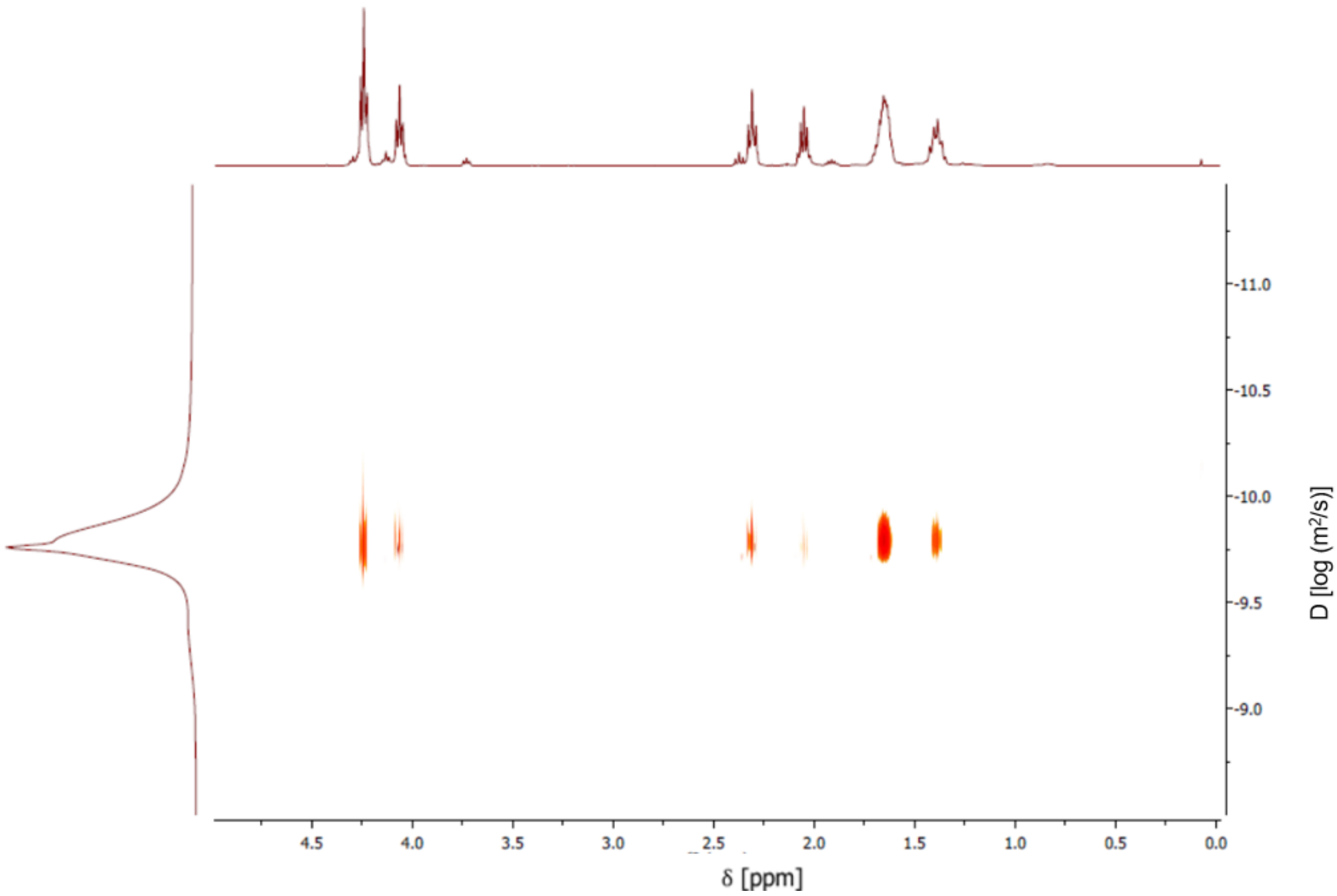

Figure S26. ^{1}H DOSY NMR spectrum (500 MHz, $CDCl_3$) of PCL_{20}-$PTMC_{20}$ (Table 3, entry 5).

The average lengths l_e of trimethylene carbonate (l_e^{TMC}) and ε-oxycaproyl (l_e^{CL}) blocks can be determined from carbon spectra using the following equations:[11]

$$l_e^{TMC} = \frac{TTT+TTC+CCT+CTC}{CTC+0.5\,[TTC+CTT]} \tag{S38}$$

$$l_e^{CL} = \frac{CCC+TCC+CCT+TCT}{TCT+0.5\,[TCC+CCT]} \tag{S39}$$

The average lengths of trimethylene carbonate and ε-oxycaproyl blocks in chains with completely random distribution of trimethylene carbonate and ε-oxycaproyl units were calculated using the equations:

$$l_R^{TMC} = \frac{1}{1-N\,TMC} \tag{S40}$$

$$l_R^{CL} = \frac{1}{N\,TMC} \tag{S41}$$

where N_{TMC} is the molar fraction of TMC units in the copolymer.

2.3.5. PLA-PGA diblocks, LA first

Under an argon atmosphere in a glovebox, *rac*-LA (80.0 mg, 555 µmol) was dissolved in dry toluene (1000 µL) and in a separate vial GA (16.0 mg, 138 µmol) was suspended in dry toluene (276 µL). In a third vial, **LZn_2Et** (5.5 mg, 6.9 µmol) was dissolved in dry toluene (100 µL) and BnOH (1.5 mg, 1.4 µL, 13.9 µmol) from a stock solution (30.0 mg mL^{-1}) was added. The three solutions were stirred separately for 30 min at 70 °C and then the vial containing the *in situ* formed **LZn_2OBn** catalyst was added to the vial of *rac*-LA in toluene ($[M]_0$ = 0.5 M). After 30 minutes of stirring at 500 rpm, the PLA* solution was added to the vial containing the

suspension of GA in toluene. After a subsequent 20 minutes, the solution had no suspended solids, and the reaction was terminated by quenching with $CDCl_3$. The product was precipitated in cold methanol to afford a white solid.

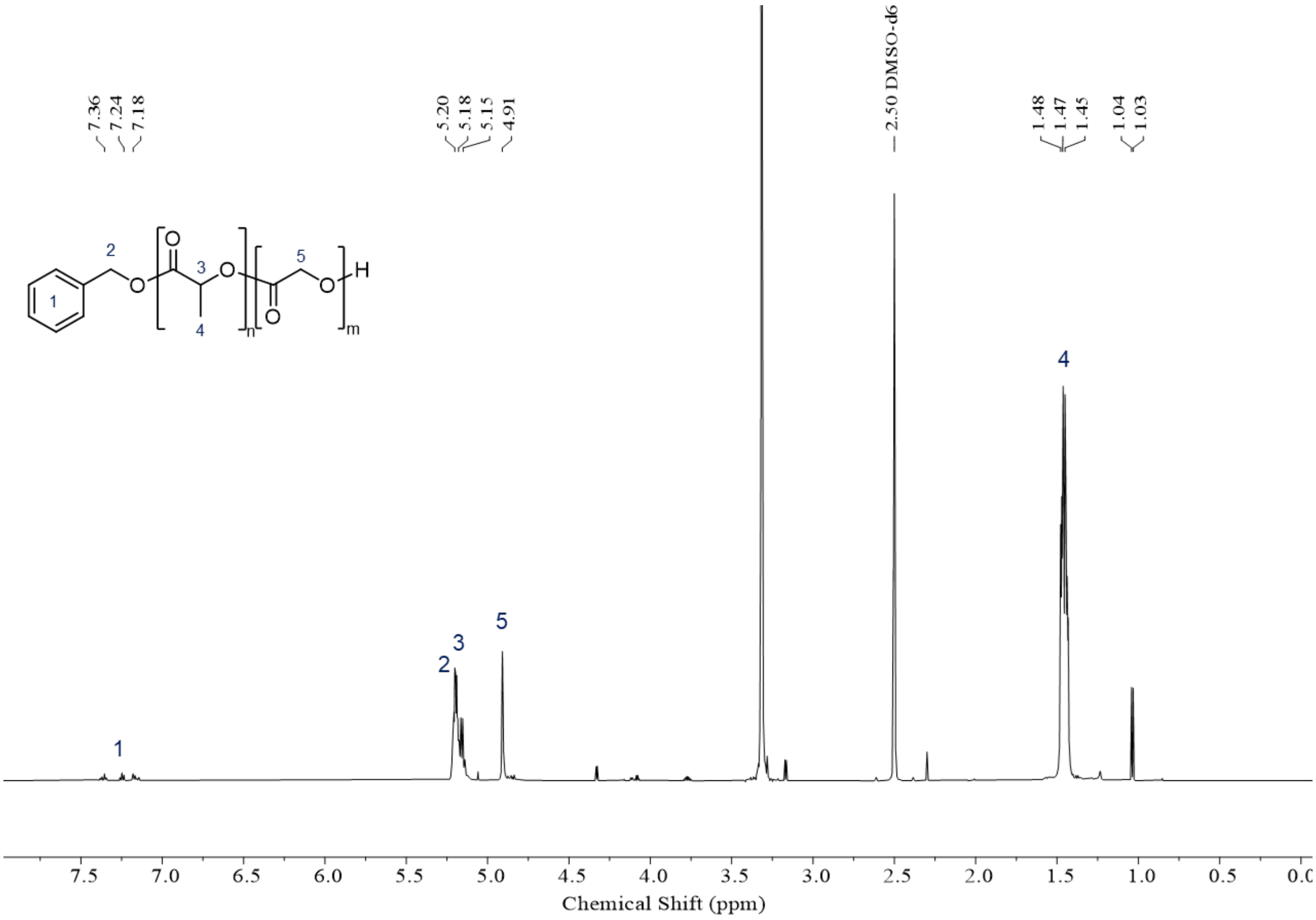

*Figure S27. ^{1}H NMR spectrum (600 MHz, DMSO-*d$_6$*) of PLA$_{80}$-PGA$_{15}$ (Table 3, entry 4)*.

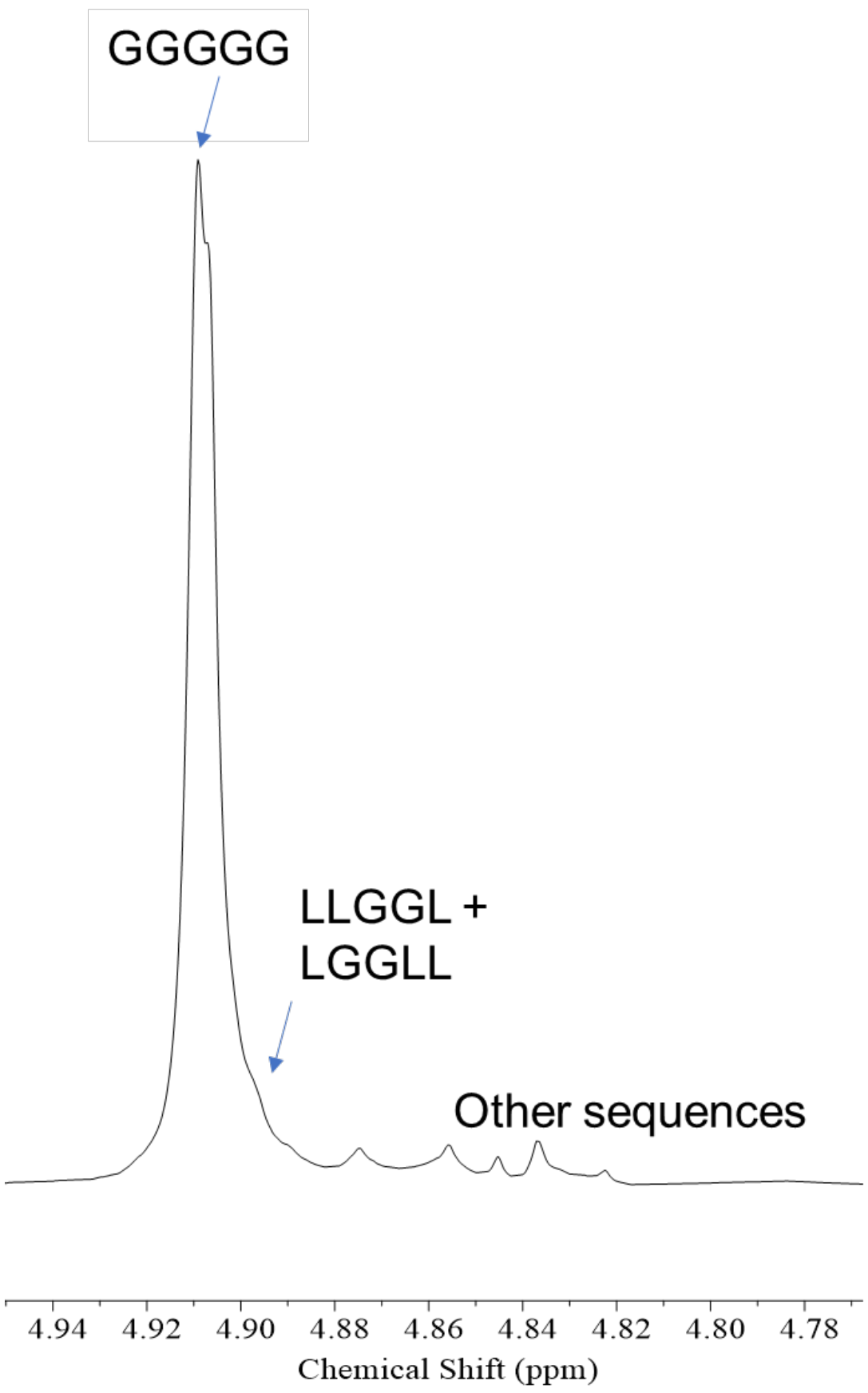

Figure S28. ^{1}H NMR spectrum (600 MHz, DMSO-d$_6$) of PLA$_{80}$-PGA$_{15}$, zoomed in on the region showing PGA sequences. Labelling as reported by Kasperczyk and co-workers (reported in DMSO-d$_6$) (Table 3, entry 4).[12]

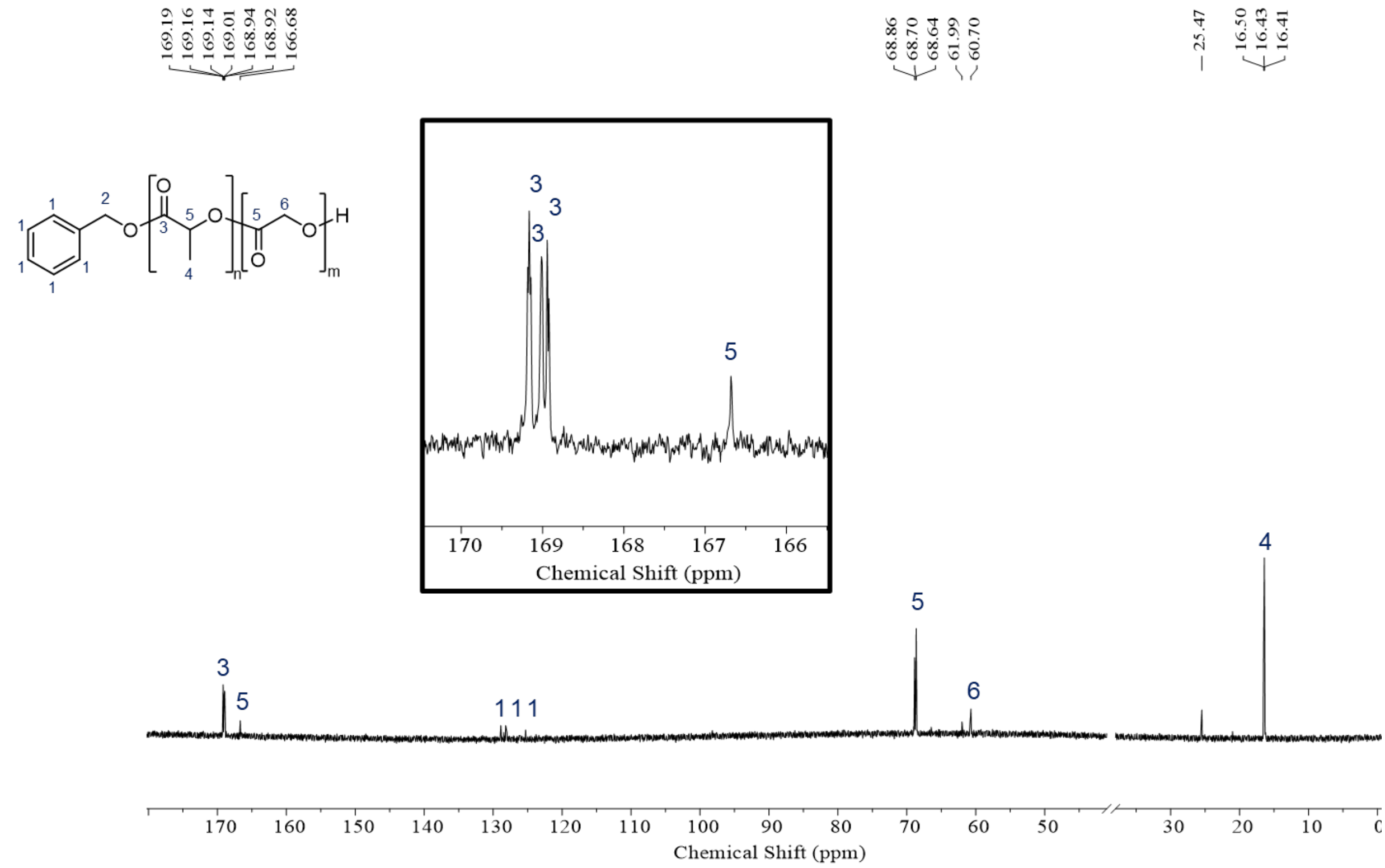

Figure S29. ^{13}C{^{1}H} NMR spectrum (126 MHz, DMSO-d$_6$, 1028 scans) of PLA$_{80}$-PGA$_{15}$ with zoomed in section on the carbonyl region to confirm block structure (multiple C=O$_{PLA}$ resonances due to atactic PLA block). Low signal to noise ratio for the carbonyl region due to solubility limitations in DMSO-d$_6$, which was necessary for comparison to the work of Kasperczyk and co-workers (Table 3, entry 4).[12]

2.4. Sequential copolymerisations with VL

2.4.1. PVL-PCL

Under an argon atmosphere in a glovebox, VL (27.6 mg, 276 µmol) was dissolved in dry toluene (200 µL) and in a separate vial **LZn_2Et** (11.0 mg, 13.8 µmol) was dissolved in dry toluene (50 µL) and BnOH (1.5 mg, 1.4 µL, 13.9 µmol) from a stock solution (30.0 mg mL^{-1}) was added. In a third vial, CL (31.5 mg, 276 µmol) was dissolved in 300 µL toluene. All vials were stirred at 70 ºC for 30 minutes, before the solution of VL in toluene was added to the catalyst solution, giving a monomer concentration of 1 M in toluene. After 20 minutes, the solution of CL in toluene was added, and the reaction was stirred for a further 30 minutes. The reaction was terminated by quenching with $CDCl_3$. The product was precipitated in cold methanol to afford a viscous clear oil.

^{1}H NMR (500 MHz, $CDCl_3$): δ 7.37-7.35 (m, 5H, aromatic), 5.12 (s, 2H, Ar-CH_2-O), 4.07 – 4.05 (m, 4H, -CH_2C(O)O- for VL + CL), 2.34 – 2.29 (m, 4H, -C(O)OCH_2- for VL + CL), 1.67 – 1.64 (m, 8H, -C(O)OCH_2*CH_2*- and -*CH_2* CH_2C(O)O- for VL + CL), 1.41 – 1.37 (m, 2H, -C(O)OCH_2CH_2*CH_2*- for CL).

^{13}C NMR (126 MHz, $CDCl_3$): δ 173.68, 173.41, 128.72, 128.35, 64.37, 64.29, 64.06, 63.98, 34.27, 34.24, 33.88, 33.85, 28.50, 28.25, 25.68, 24.73, 21.59.

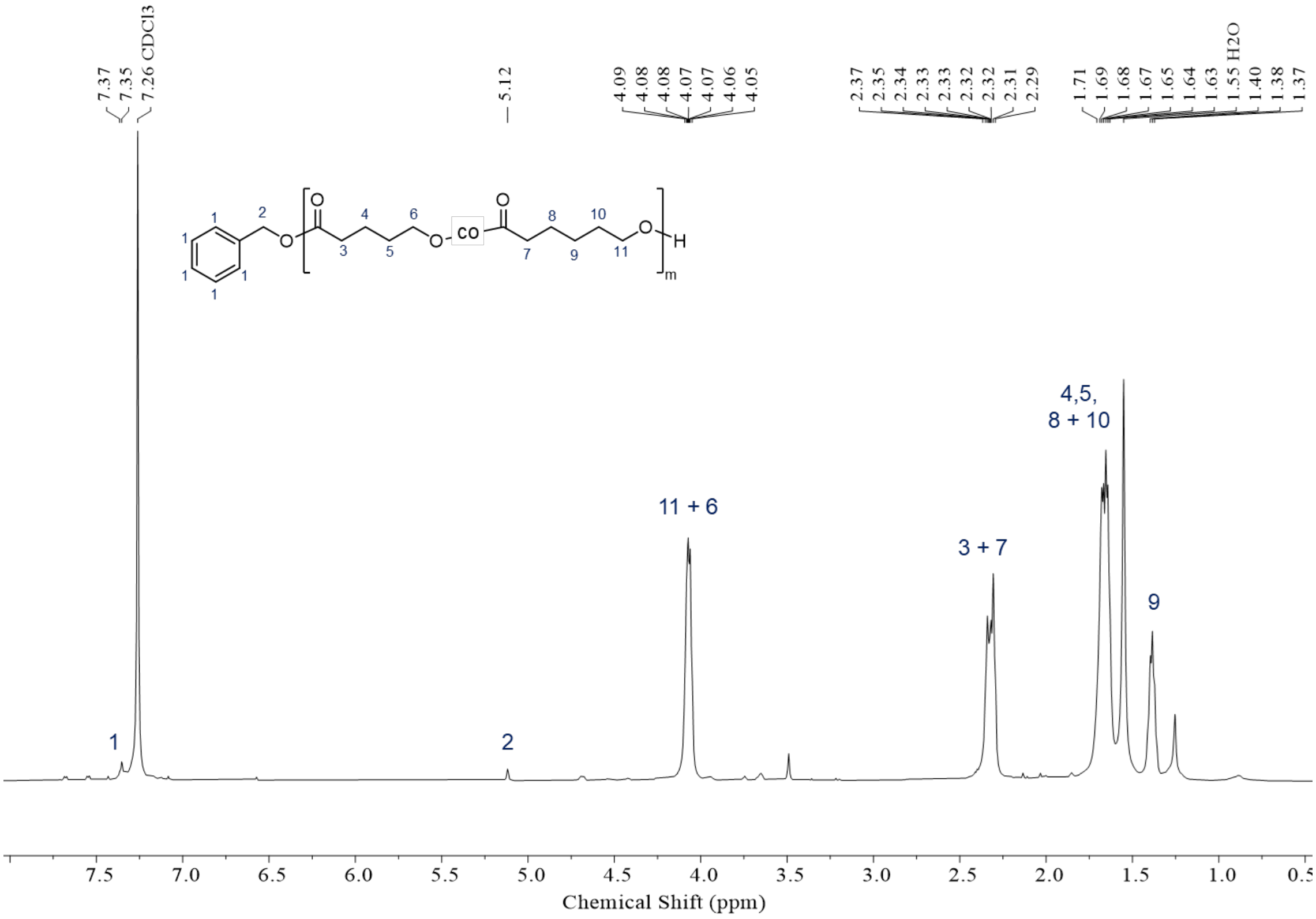

Figure S30. ^{1}H NMR (500 MHz, $CDCl_3$) of the polymer resulting from ROP of 20 eq. CL from PVL_{20}.*

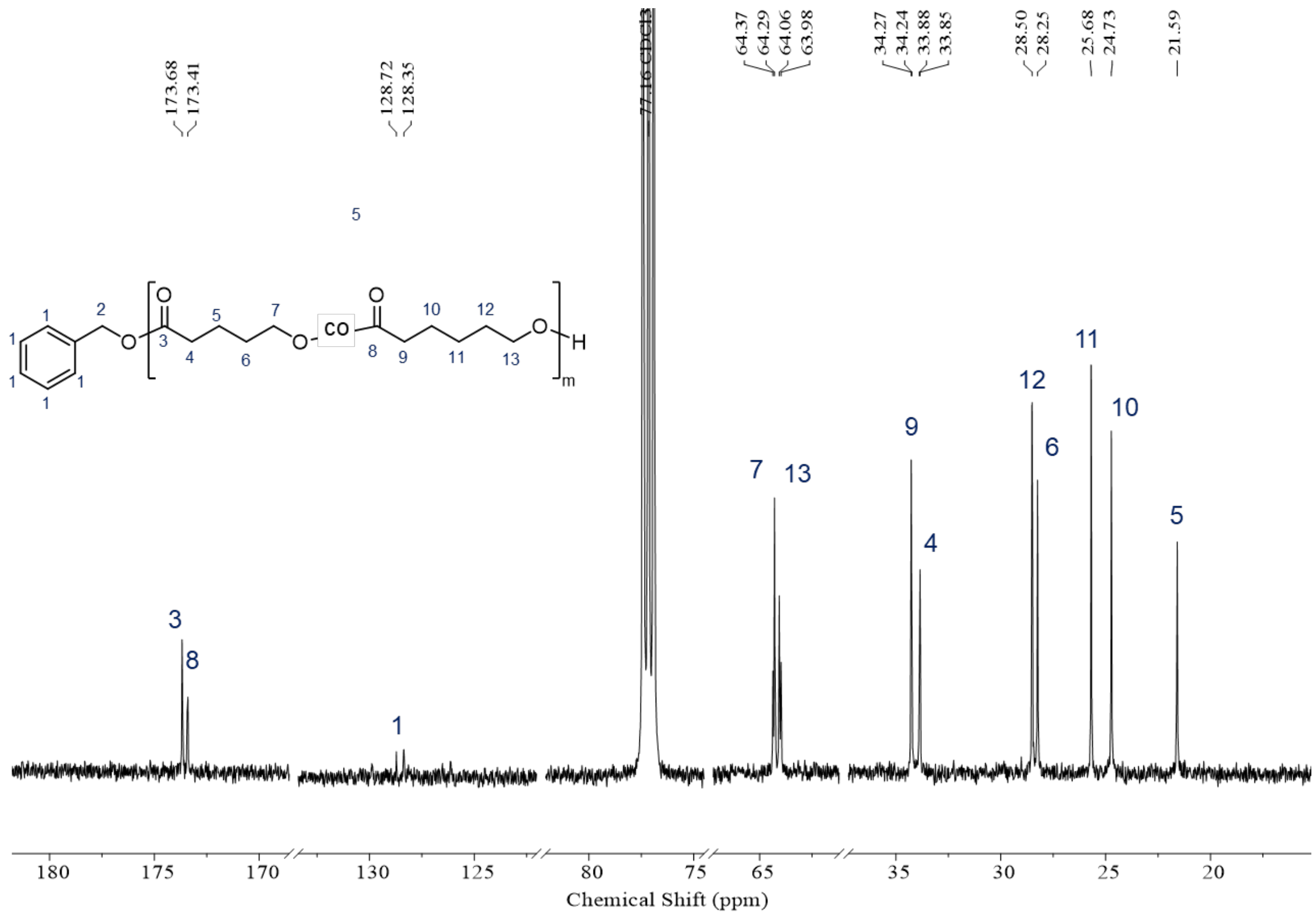

Figure S99. ^{13}C NMR (126 MHz, $CDCl_3$) of the polymer resulting from ROP of 20 eq. CL from PVL_{20}.*

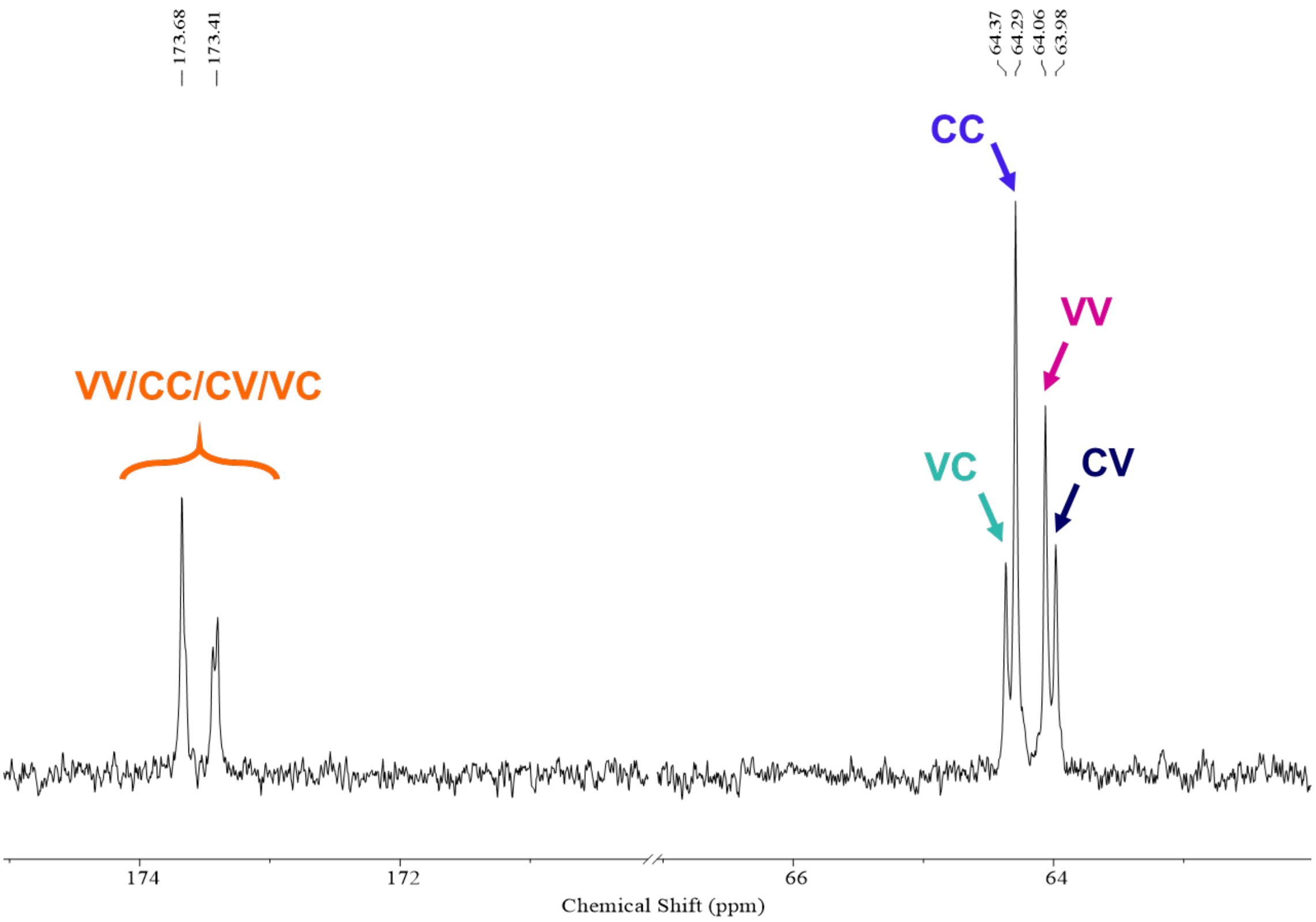

Figure S31. Zoom in of regions of Figure S99, highlighting the sequences associated with a random PVL/PCL copolymer structure, assigned according to Verpoort and co-workers.[4,13]

2.4.2. PCL-PVL

Under an argon atmosphere in a glovebox, CL (31.5 mg, 276 μmol) was dissolved in dry toluene (200 μL) and in a separate vial **LZn_2Et** (11.0 mg, 13.8 μmol) was dissolved in dry toluene (50 μL) and BnOH (1.5 mg, 1.4 μL, 13.9 μmol) from a stock solution (30.0 mg mL^{-1}) was added. In a third vial, VL (27.6 mg, 276 μmol) was dissolved in 300 μL toluene. All vials were stirred at 70 ºC for 30 minutes, before the solution of CL in toluene was added to the catalyst solution, giving a monomer concentration of 1 M in toluene. After 30 minutes, the solution of VL in toluene was added, and the reaction was stirred for a further 30 minutes. The react was terminated by quenching with $CDCl_3$. The product was precipitated in cold methanol to afford a white waxy solid.

^1H NMR (500 MHz, $CDCl_3$): δ 7.38-7.32 (m, 5H, aromatic), 5.11 (s, 2H, Ar-*CH_2*-O), 4.09 – 4.05 (m, 4H, -$CH_2C(O)O$- for VL + CL), 2.38 – 2.28 (m, 4H, -$C(O)OCH_2$- for VL + CL), 1.70 – 1.61 (m, 8H, -$C(O)OCH_2$*CH_2*- and -*CH_2* $CH_2C(O)O$- for VL + CL), 1.41 – 1.36 (m, 2H, -$C(O)OCH_2CH_2$*CH_2*- for CL).

^{13}C NMR (126 MHz, $CDCl_3$): δ 173.66, 173.39, 128.72, 128.35, 64.27, 64.05, 63.98, 34.25, 33.85, 33.83, 28.49, 28.22, 25.67, 24.71, 21.56.

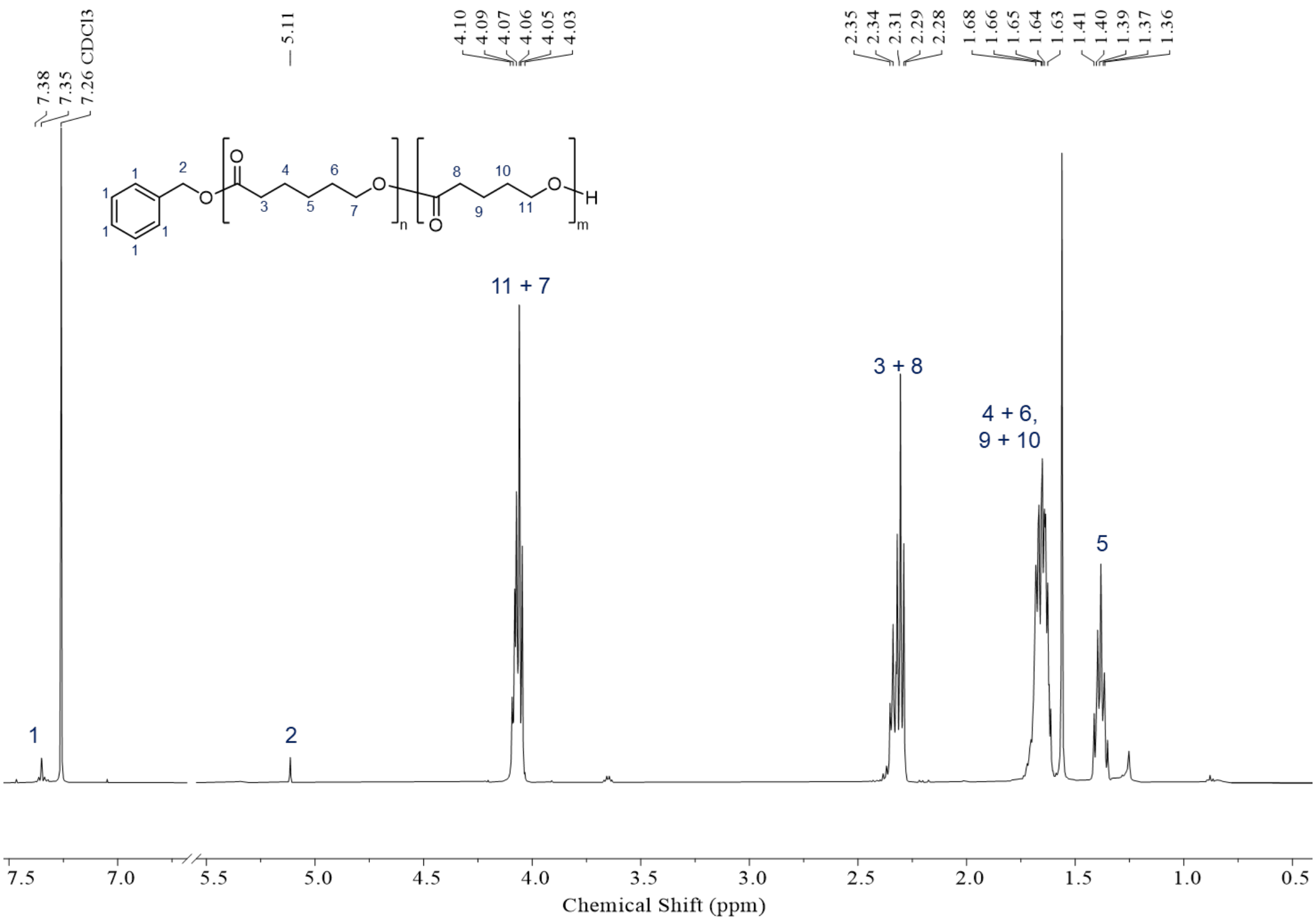

Figure S32. ^1H NMR (500 MHz, $CDCl_3$) of the polymer resulting from ROP of 20 eq. VL from PCL_{20}.*

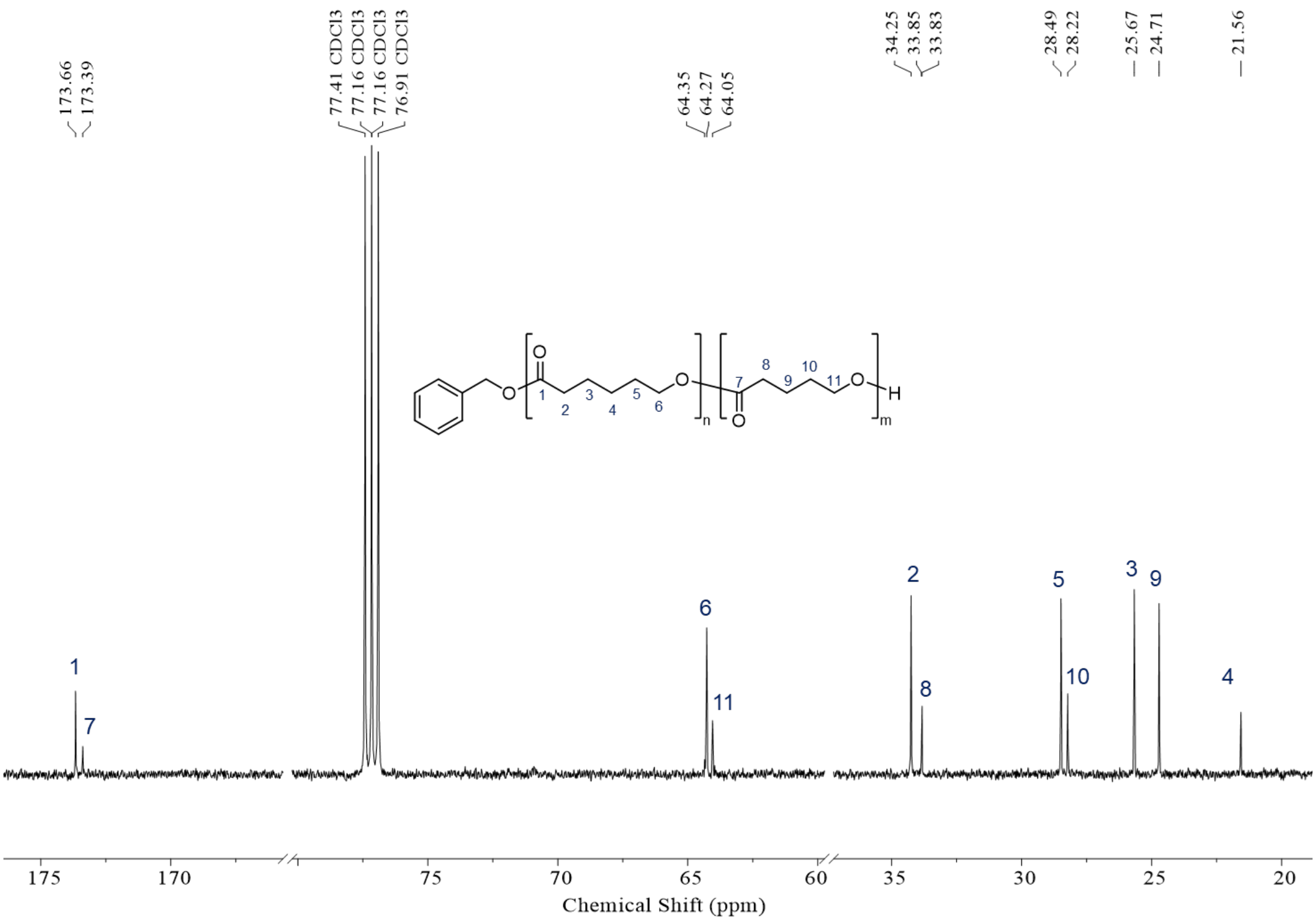

Figure S33. ^{13}C NMR (126 MHz, $CDCl_3$) of the polymer resulting from ROP of 20 eq. VL from PCL_{20}.*

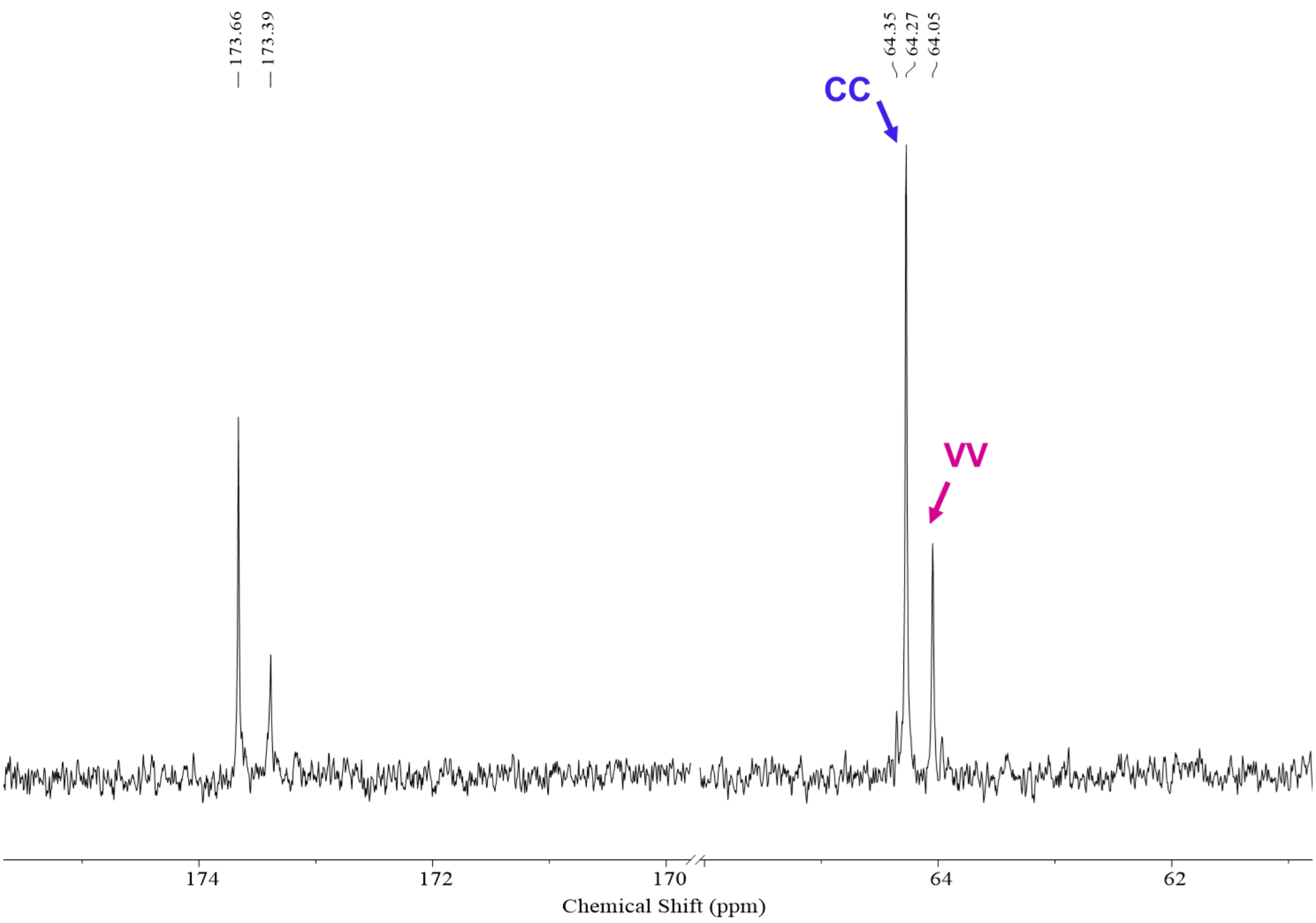

Figure S34. Zoom in on carbonyl region of figure S102, highlighting the blocky structure

2.4.3. PVL-PLA

Under an argon atmosphere in a glovebox, VL (27.6 mg, 276 μmol) was dissolved in dry toluene (200 μL). In a separate vial, **LZn_2Et** (11.0 mg, 13.8 μmol) was dissolved in dry toluene (50 μL), and BnOH (1.5 mg, 1.4 μL, 13.9 μmol) from a stock solution (30.0 mg mL^{-1}) was added. In a third vial, *rac*-LA (39.2 mg, 276 μmol) was dissolved in 300 μL toluene. All three vials were stirred at 70 ºC for 30 minutes, before the solution of VL in toluene was added to the catalyst solution, giving a monomer concentration of 1 M in toluene. After 20 minutes, the solution of VL and catalyst in toluene was added to the vial containing *rac*-LA in toluene, and the reaction was stirred for a further 30 minutes. The reaction was terminated by quenching with $CDCl_3$. The product was precipitated in cold methanol to afford a fibrous white solid.

^1H NMR (500 MHz, $CDCl_3$): δ 7.36-7.29 (m, 5H, aromatic), 5.24-5.10 (m, 2H·m, -C*H*(CH_3)O-), 4.16 – 4.14 (bt, 2H, VL-LA junction $CH_2C(O)O$), 4.09 – 4.07 (t, 2H·n, -CH_2O-), 2.41 – 2.37 (bt, 2H, -C*H*$_2$CO- junction peak), 2.39-2.31 (m, 2H, -C*H*$_2$CO-), 1.72-1.66 (m, 4H·n, -C*H*$_2$C*H*$_2$$CH_2O$-), 1.59 – 1.49 (m, 6H, -COCH_2C*H*$_2$-, -C*H*$_2$CHO-, -CH(C*H*$_3$)O-).

^{13}C NMR (126 MHz, $CDCl_3$): δ 173.40, 169.74, 169.55, 169.49, 169.27, 128.72, 128.39, 128.34, 128.13, 126.14, 69.56, 69.32, 69.15, 66.38, 65.13, 64.06, 51.01, 33.88, 33.84, 33.70, 28.24, 28.20, 28.07, 27.91, 24.18, 21.57, 21.36, 20.66, 16.89, 16.82, 16.79, 16.76.

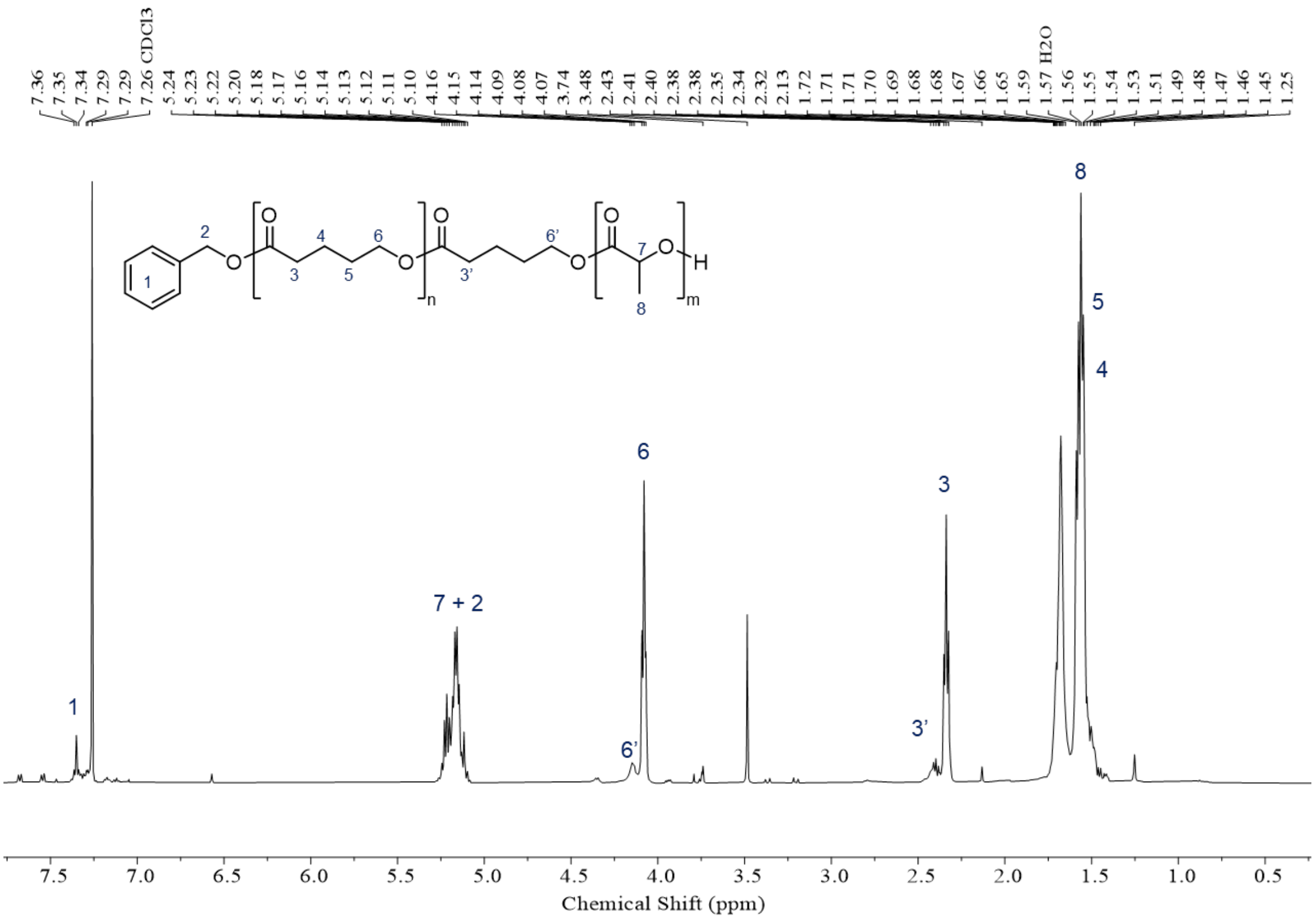

Figure S35. ^1H NMR (500 MHz, $CDCl_3$) of the polymer resulting from ROP of 20 eq. rac-*LA from PVL_{20}*.*

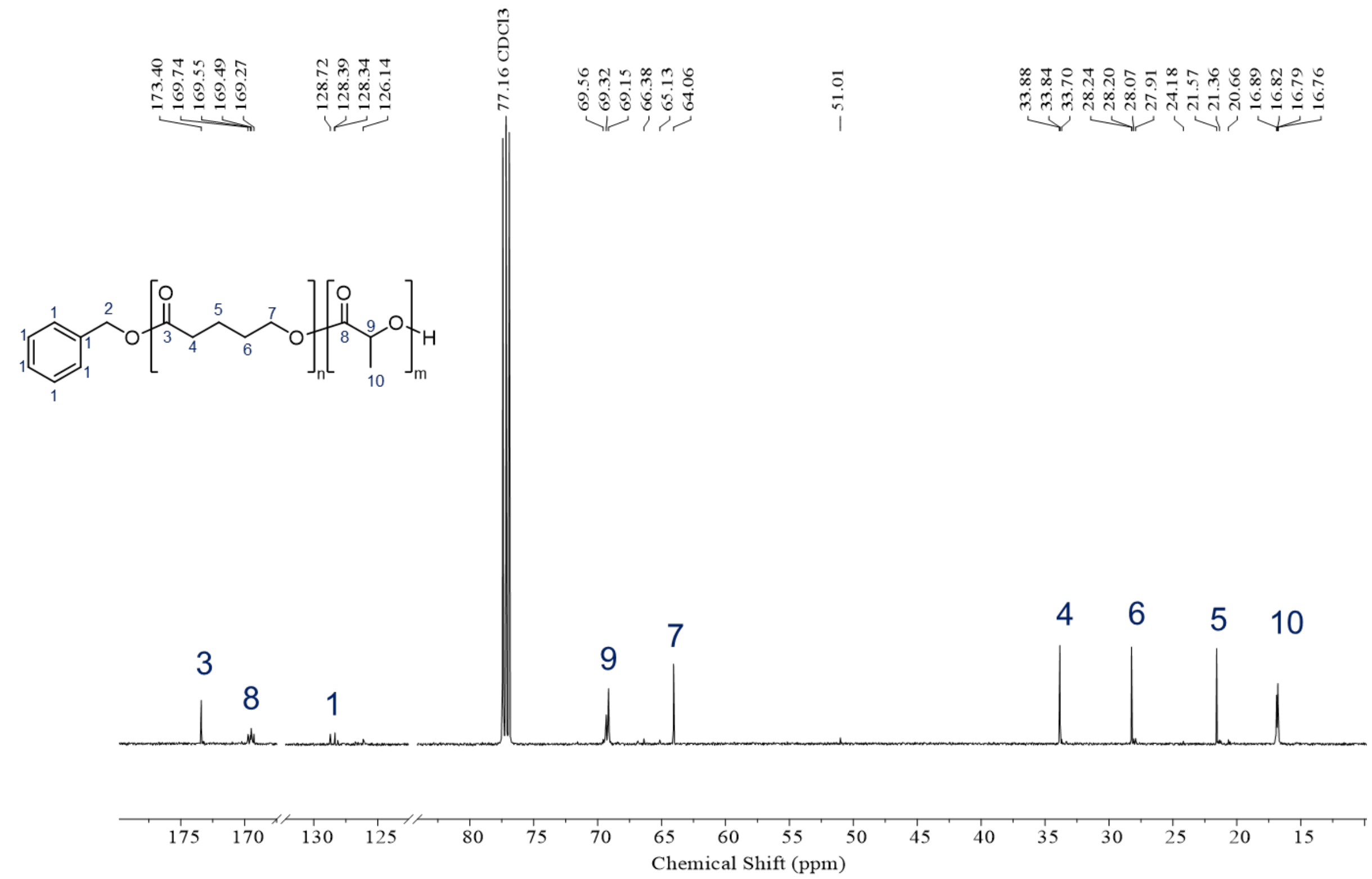

Figure S36. ^{13}C NMR (126 MHz, $CDCl_3$) of the polymer resulting from ROP of 20 eq. rac-LA from PVL_{20}.*

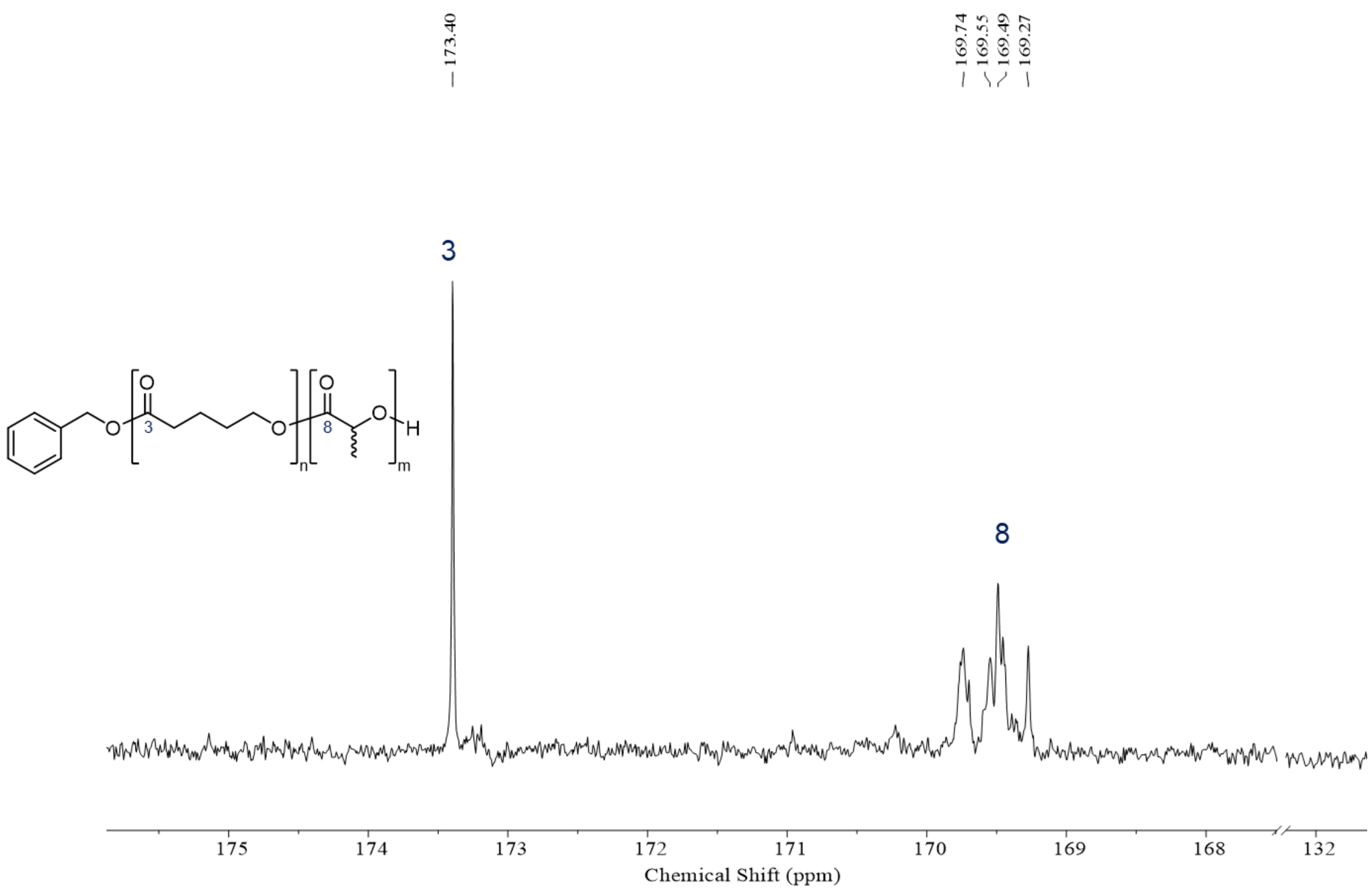

Figure S37. Zoom in on carbonyl region of Figure S105, highlighting the blocky nature of the PVL-b-PLA. Multiple signals observed for PLA due to multiple stereosequences present.

2.4.4. PLA-PVL

Under an argon atmosphere in a glovebox, *rac*-LA (39.2 mg, 276 µmol) was dissolved in dry toluene (200 µL). In a separate vial, **LZn_2Et** (11.0 mg, 13.8 µmol) was dissolved in dry toluene (50 µL) and BnOH (1.5 mg, 1.4 µL, 13.9 µmol) from a stock solution (30.0 mg mL^{-1}) was added. In a third vial, VL (27.6 mg, 276 µmol) was dissolved in 300 µL toluene. All three vials were stirred at 70 ºC for 30 minutes, before the solution of **LZn_2Et**/BnOH in toluene was added to the *rac*-LA solution, giving a monomer concentration of 1 M in toluene. After 30 minutes, the solution of VL was added to the vial containing *rac*-LA + catalyst in toluene, and the reaction was stirred for a further 240 minutes. The reaction was terminated by quenching with $CDCl_3$. The product was precipitated in cold methanol to afford a clear oil.

^{1}H NMR (500 MHz, $CDCl_3$): δ 7.35 (m, 5H, aromatic), 5.20-5.07 (m, 2H·m, -C*H*(CH_3)O-), 4.15 (bt, CH_2C(O)O VL-LA junction), 4.08 (t, 2H·n, -CH_2O- of VL), 2.43 – 2.42 (bt, 2H, -C*H*$_2$CO- VL-LA junction), 2.35-2.34 (m, 2H, -C*H*$_2$CO-), 1.71-1.68 (m, 4H·n, -C*H*$_2$C*H*$_2$$CH_2$O-), 1.57 – 1.43 (m, 6H, -COCH_2C*H*$_2$-, -C*H*$_2$CHO-, -CH(C*H*$_3$)O-).

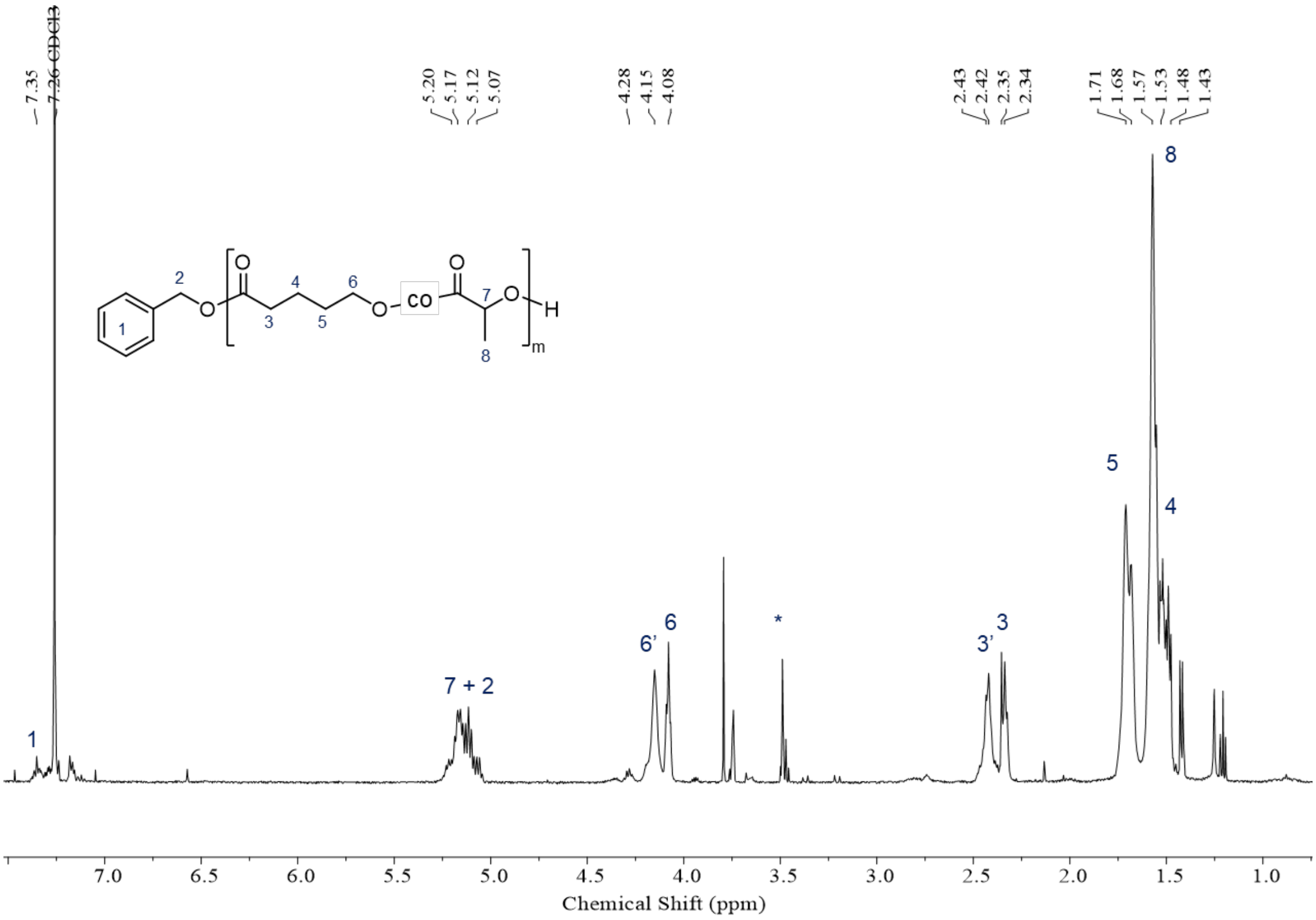

Figure S38. ^{1}H NMR (500 MHz, $CDCl_3$) of the polymer resulting from ROP of 20 eq. VL from PLA_{20}. * indicates residual solvent (MeOH, toluene).*

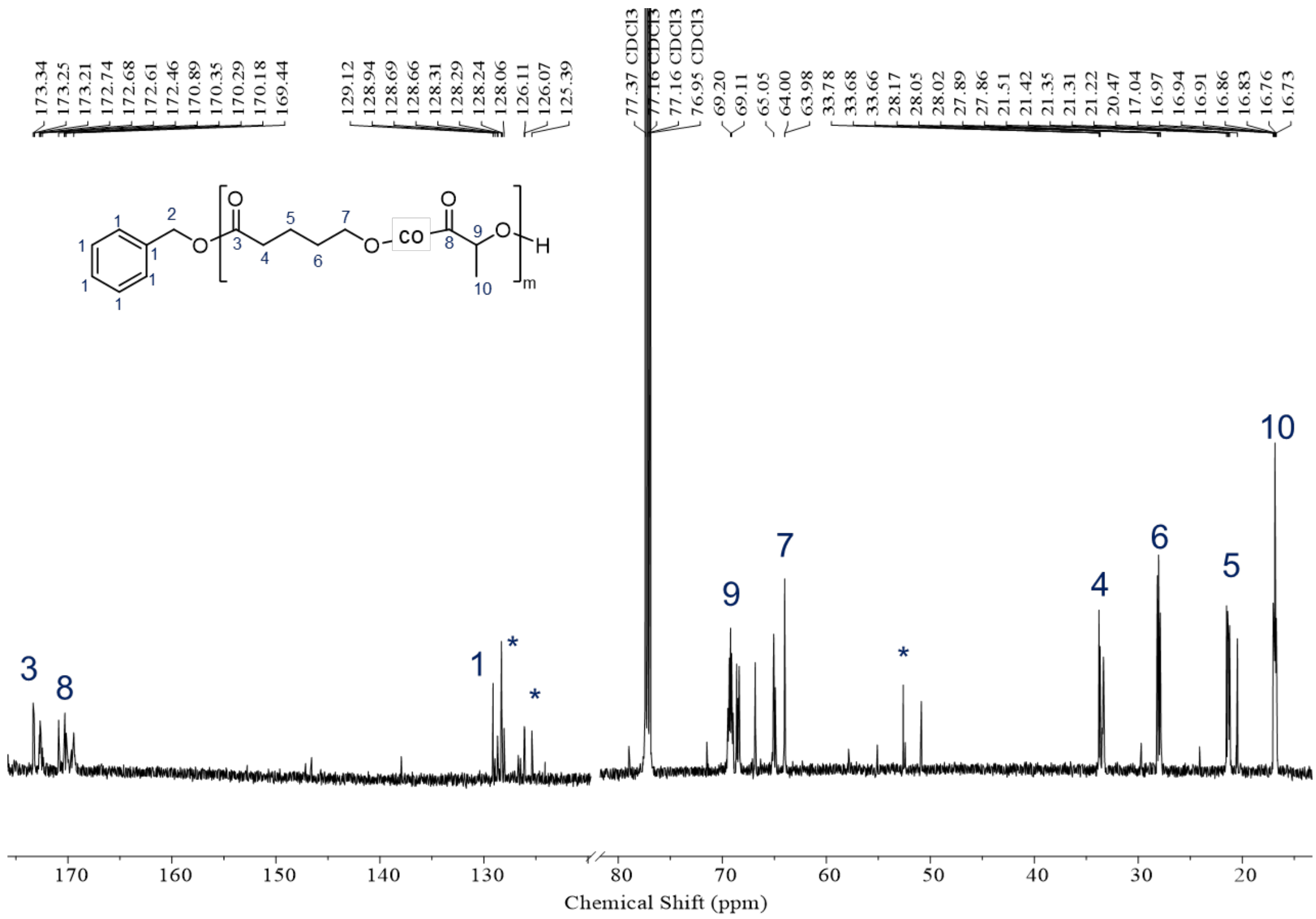

Figure S39. ^{13}C NMR (126 MHz, $CDCl_3$) of the polymer resulting from ROP of 20 eq. VL from PLA_{20}. (MeOH, toluene).*

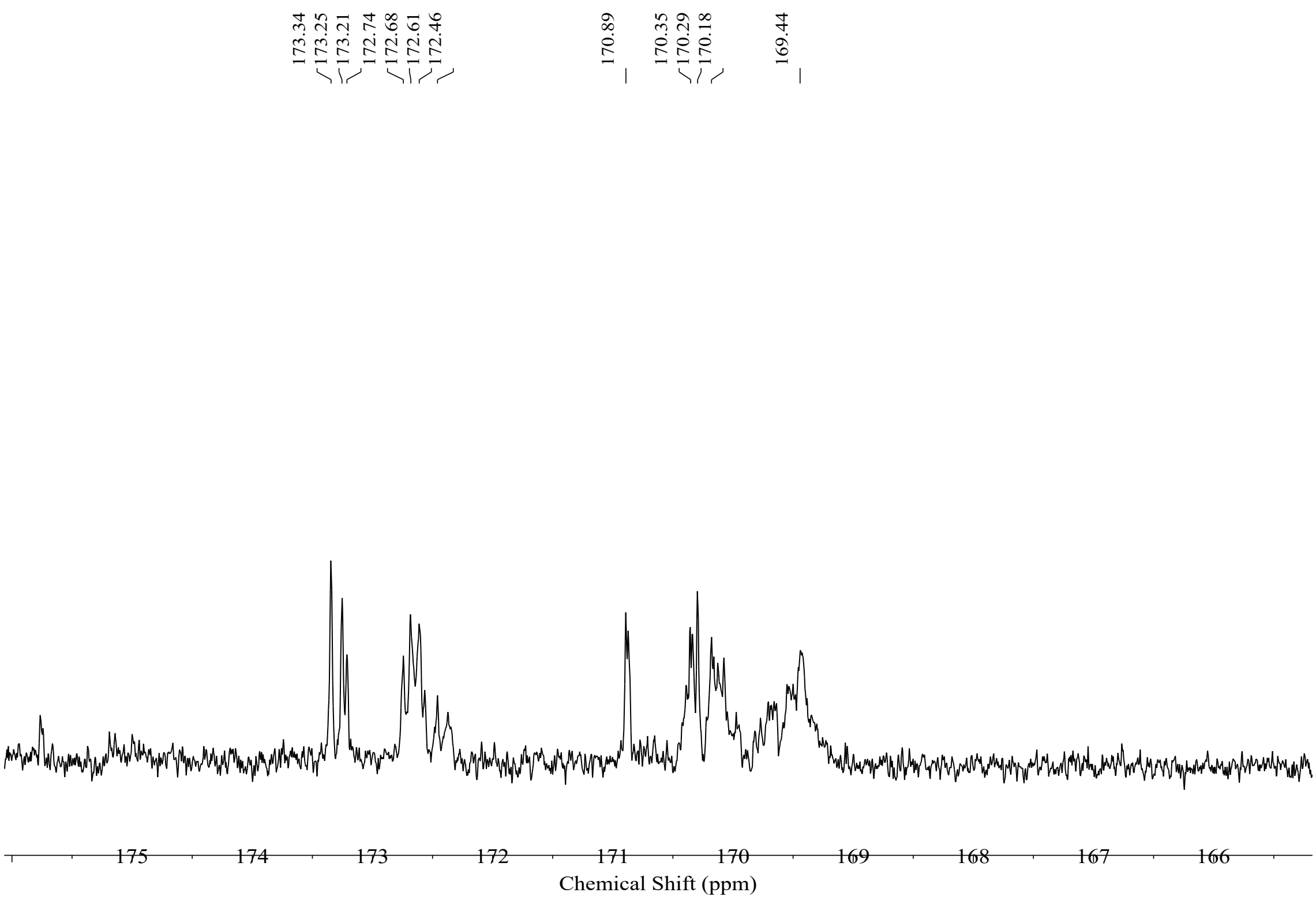

Figure S40. Zoom in of Figure S108, highlighting the random copolymer structure.

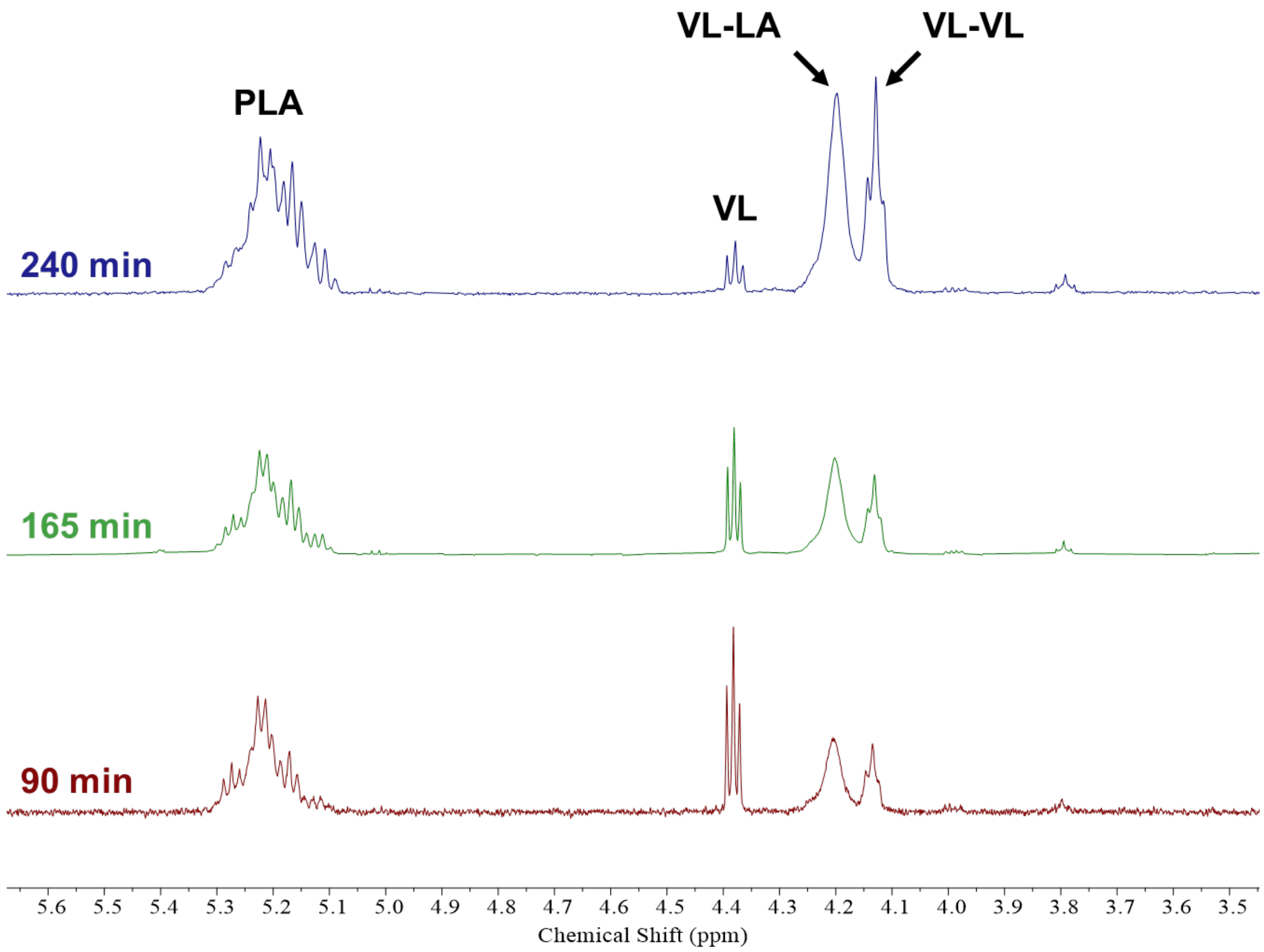

Figure S4110. ^1H NMR (500 MHz, $CDCl_3$) of aliquots of VL ROP from PLA from 90 – 240 min, highlighting the emergence of transesterification even at low conversions of VL (evidenced by the ratio of VL-LA to VL-VL units).*

3. Hydrogen bond acceptor (HBA) character for monomers and polymers

3.1. Methodology

To probe the hydrogen bond acceptability of monomers and polymers, ^{19}F NMR experiments using pentafluorobenzoic acid (PFBA) were adapted from the methodology of Franz and co-workers.[14] Based on a final PFBA concentration of 5.45 μM for the NMR sample, the selected HBA (1.0 – 20.0 eq.) was dissolved in 0.4 mL of CH_2Cl_2 and transferred to an oven-dried NMR tube in a glovebox. Subsequently, 0.15 mL (1.0 eq., 0.003 mmol) of a stock solution of PFBA (0.02 M in CD_2Cl_2) was added to the NMR tube, which was shaken several times to allow for mixing. The ^{19}F NMR spectrum of each sample was collected at room temperature with 16 scans and compared to a 5.45 μM external standard of PFBA (0.15 mL probe stock solution in CD_2Cl_2 + 0.4 mL of CH_2Cl_2) to measure the change in the chemical shift (ppm). All monomers considered in the HBA studies were purified according to the methodology in the general methods section. Where possible, polymers were used as received from SigmaAldrich. PDL and PVL were synthesised using the methodology described in the respective homopolymer sections.

3.2. Exemplar spectra

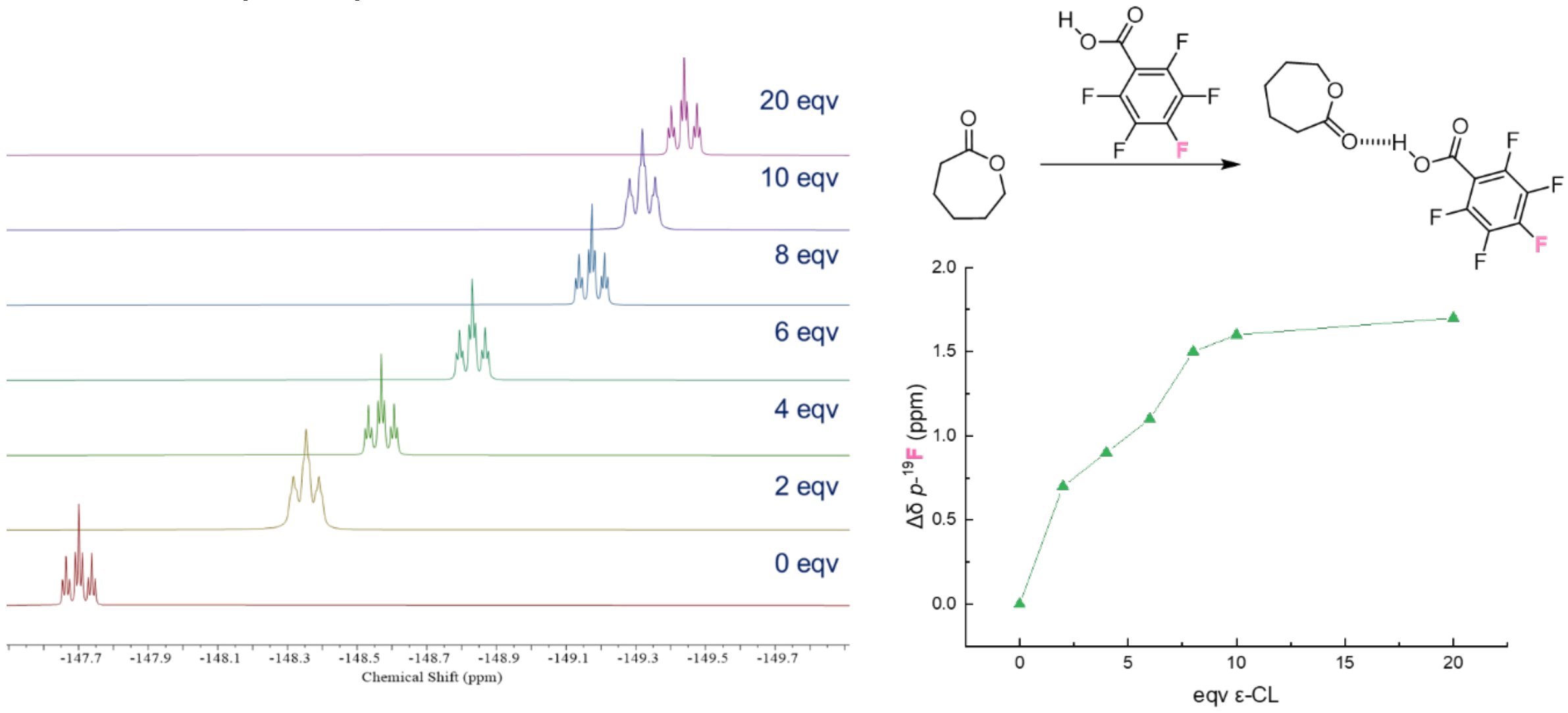

Figure S42. Stacked ^{19}F NMR plots of PFBA with increasing equivalents (eq.) of CL measured at 558 MHz in CD_2Cl_2/CH_2Cl_2 at 303 K (left). Plot of $\Delta\delta_p$ vs eq. CL added (right).

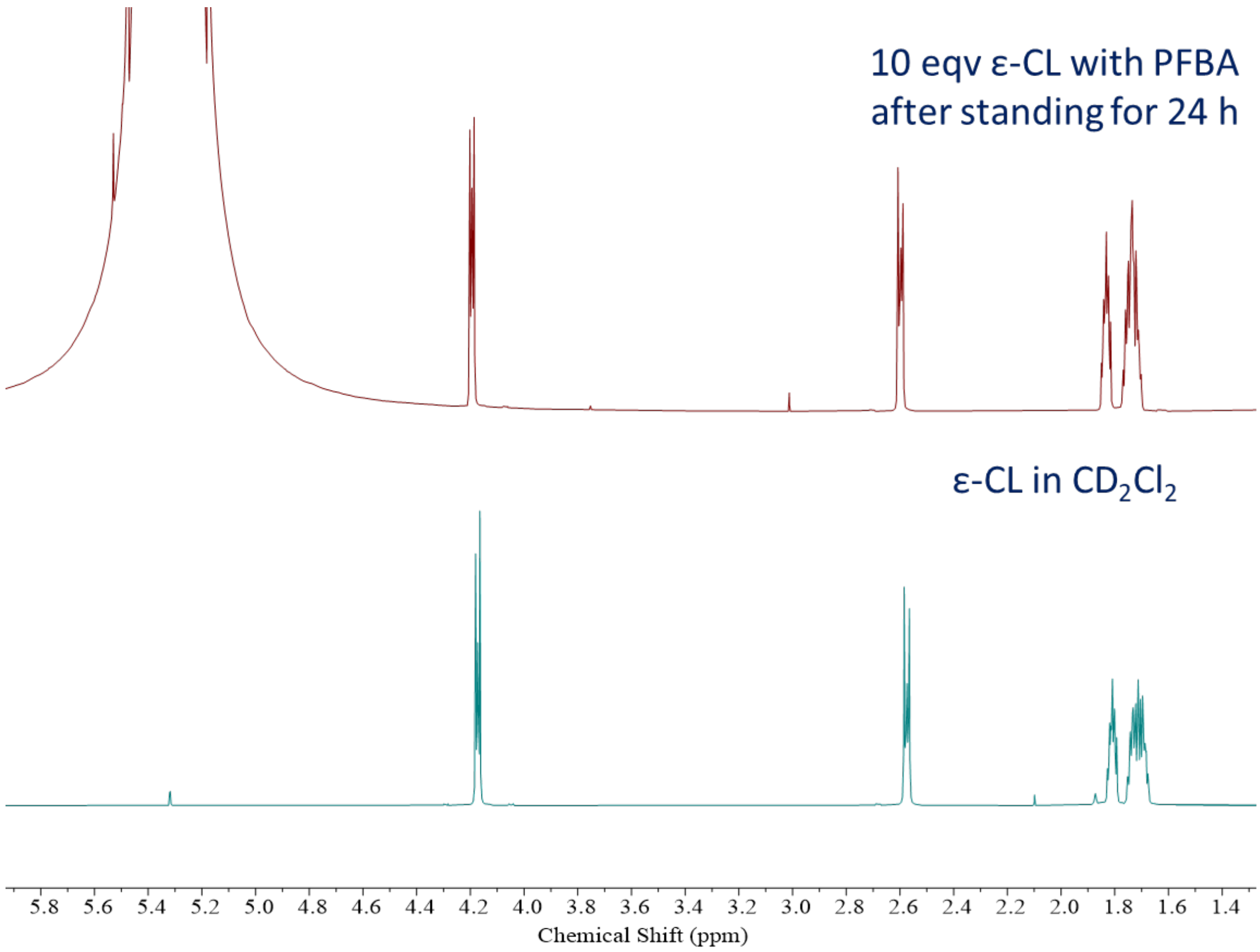

Figure S43. Stacked ^{1}H NMR spectra (600 MHz, CD_2Cl_2) of CL (bottom) and CL after sitting with 0.1 eq. PFBA probe for 24 h (top).

3.3. $\Delta\delta_p$ vs equivalents for all monomers considered in this study

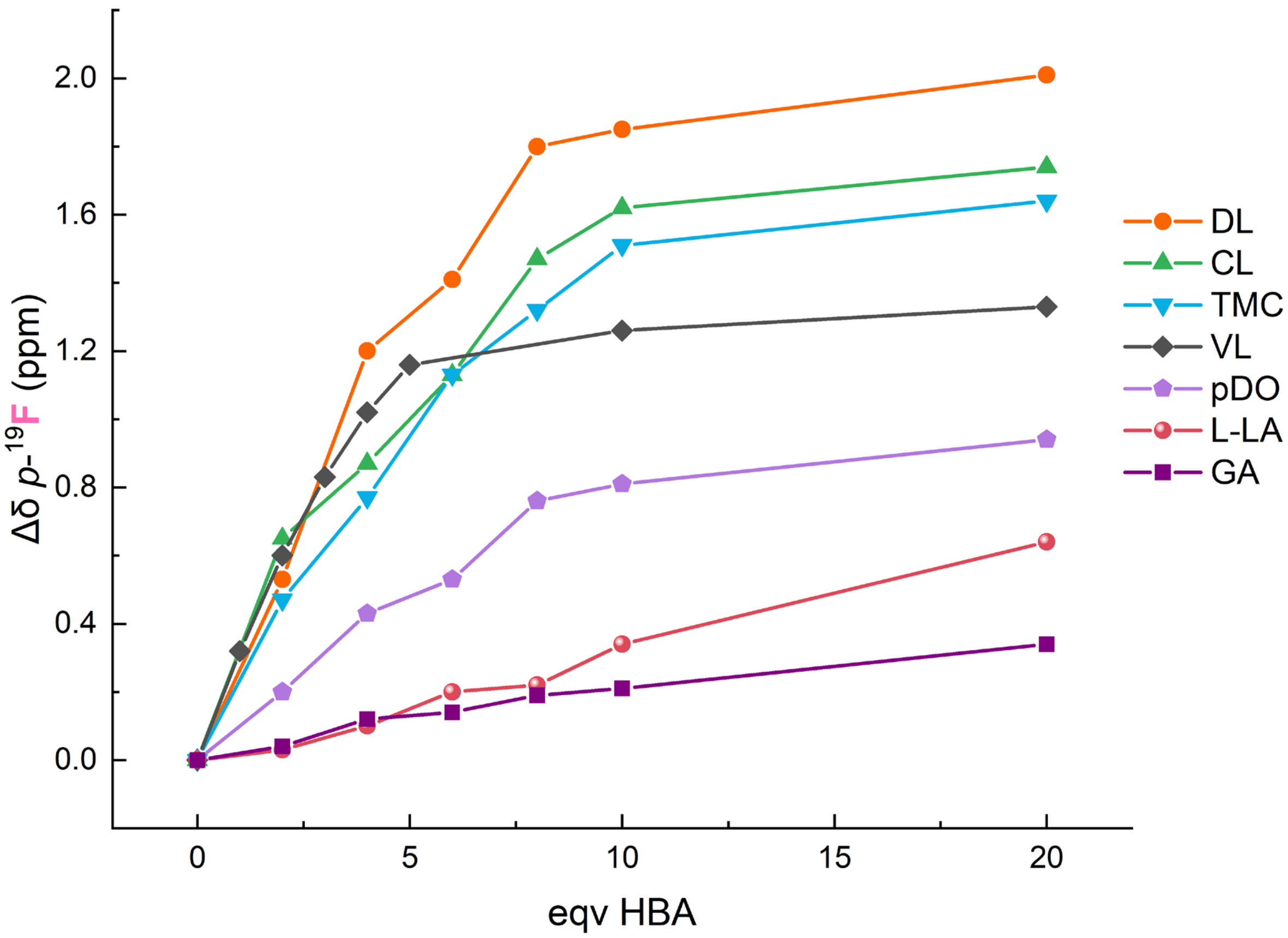

Figure S44. Plot of Δδp vs eq. HBA added for a range of monomers. All measured up to and including 20 eq. of HBA per PFBA probe.

Table S6. Testing reproducibility of $\Delta\delta_p(^{19}F)$ NMR values over multiple trials with standard deviation and error calculated for CL and PDL.

Entry	HBA	Eq. per PFBA	$\Delta\delta_p(^{19}F)$ (ppm)					Std. dev (±)	Std. error
			Trial 1	Trial 2	Trial 3	Trial 4	Average		
1	CL	10	1.62	1.63	1.72	1.75	1.70	0.0624	0.0255
2	PDL	10	2.07	1.97	2.06	-	2.03	0.0551	0.0225
3	PDL	20	2.75	2.94	3.07	-	2.92	0.1610	0.0657

Table S7. $\Delta\delta_p$ ^{19}F NMR data for polyesters and polycarbonates (TMC) binding to PFBA, along with $\delta_{C=O}$ ^{13}C NMR data and C=O FT-IR stretching frequencies for polyesters and polycarbonates.[a]

Polymer	$\Delta\delta_p$ ^{19}F NMR (ppm)[b]	^{13}C NMR peak for $^{13}C=O$ ($CDCl_3$)[c]	FT-IR C=O Stretching Frequency (cm^{-1})[15–22]	Assigned Relative Lewis Basicity
PGA	-[d]	-[d]	1760[15]	Medium-high
P3HB	0.5	169.1[23]	1730[16]	Low
PLA	0.5	169.4	1749[17]	Low
PDL	2.9	173.1	1743[18]	Medium
PCL	0.9	173.5	1721[19]	Medium-high
P*p*DO	-[d]	-[d]	1733[20]	Medium-high
PVL	1.1	173.4	1730[22]	Medium-high
PTMC	1.3	155.0	1741[21]	Medium-high

[a] 20 molar eq. of hydrogen bond acceptor (HBA) (monomer or repeat unit of polymer) in CH_2Cl_2 (27% CD_2Cl_2, PFBA probe concentration = 5.45 μM) at 300 ± 1 K analysed by ^{19}F NMR spectroscopy (558 MHz).
[b] ($\Delta\delta_p$F reported for *para*-fluorine of PFBA in the presence of HBA compared to PFBA external standard. See Supporting Information for all standard deviations and errors.
[c] The ^{13}C NMR shifts for PTMC are significantly lower as the carbonyl ^{13}C is part of a carbonate unit, rather than an ester unit.
[d] Not measured due to poor solubility of P*p*DO and PGA in CH_2Cl_2.

Table S8. $\Delta\delta_p$ ^{19}F NMR values upon binding to PFBA for all HBA molecules studied.

Entry	HBA	Eq. per PFBA	$\Delta\delta_p$ ^{19}F (ppm)
1	L-LA	10	0.34
2	L-LA	20	0.64
3	CL	10	1.62
4	CL	20	1.74
5	TMC	10	1.51
6	TMC	20	1.64
7	*p*DO	10	0.81
8	*p*DO	20	0.94
9	DL	10	1.85
10	DL	20	2.01
11	GA	10	0.21
12	GA	20	0.34
13	VL	10	1.26
14	VL	20	1.33
15	PLLA	10	0.38
16	PLLA	20	0.53
17	PCL	10	0.82
18	PCL	20	0.84
19	PTMC	10	0.88
20	PTMC	20	1.34

21	PVL	10	0.78
22	PVL	20	1.11
23	PDL	10	2.07
24	PDL	20	2.75

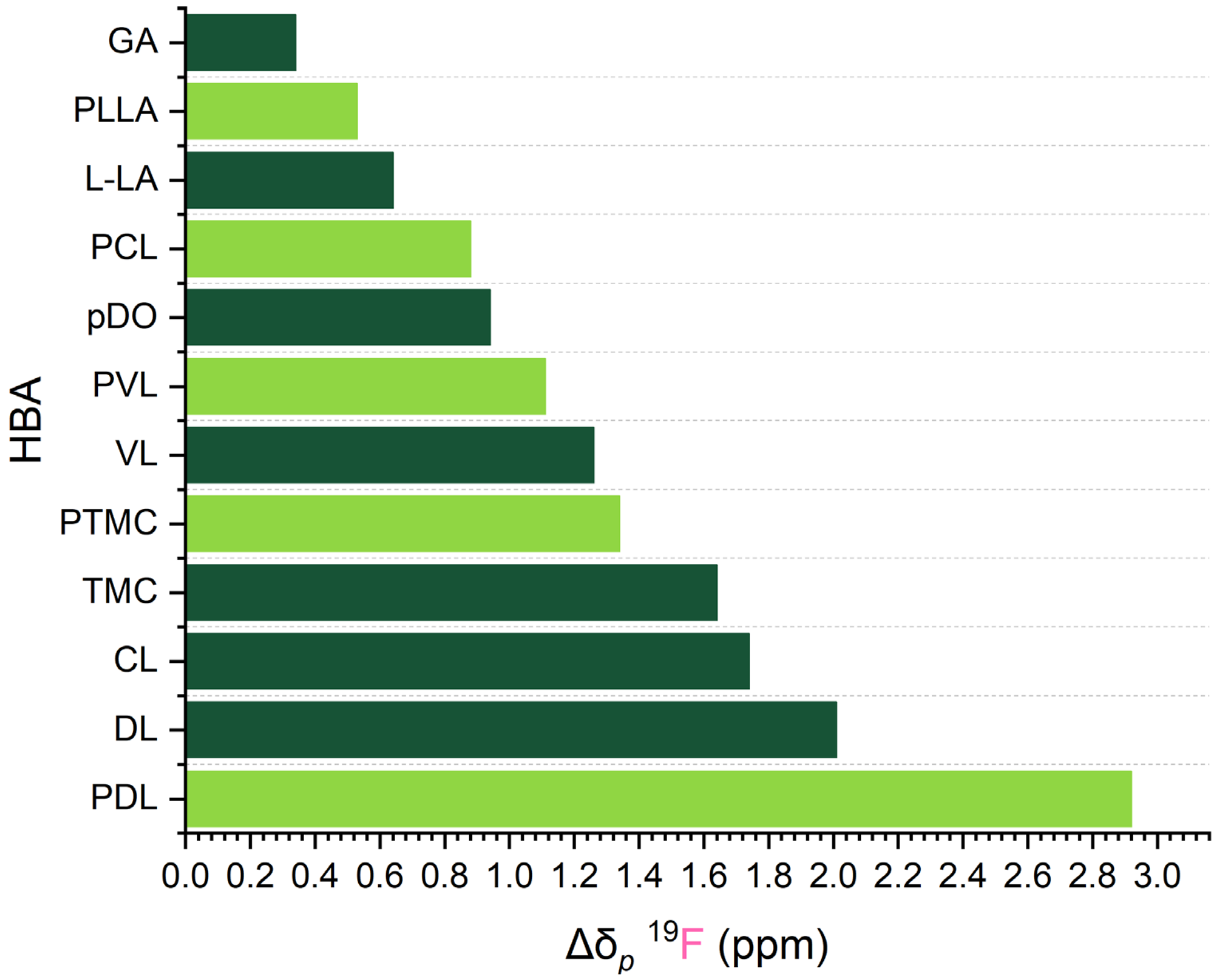

Figure S45. $\Delta\delta_p(^{19}F)$ NMR values versus HBA studied at 20 eq. per PFBA. Polymeric HBAs in light green, monomeric HBAs in dark green.

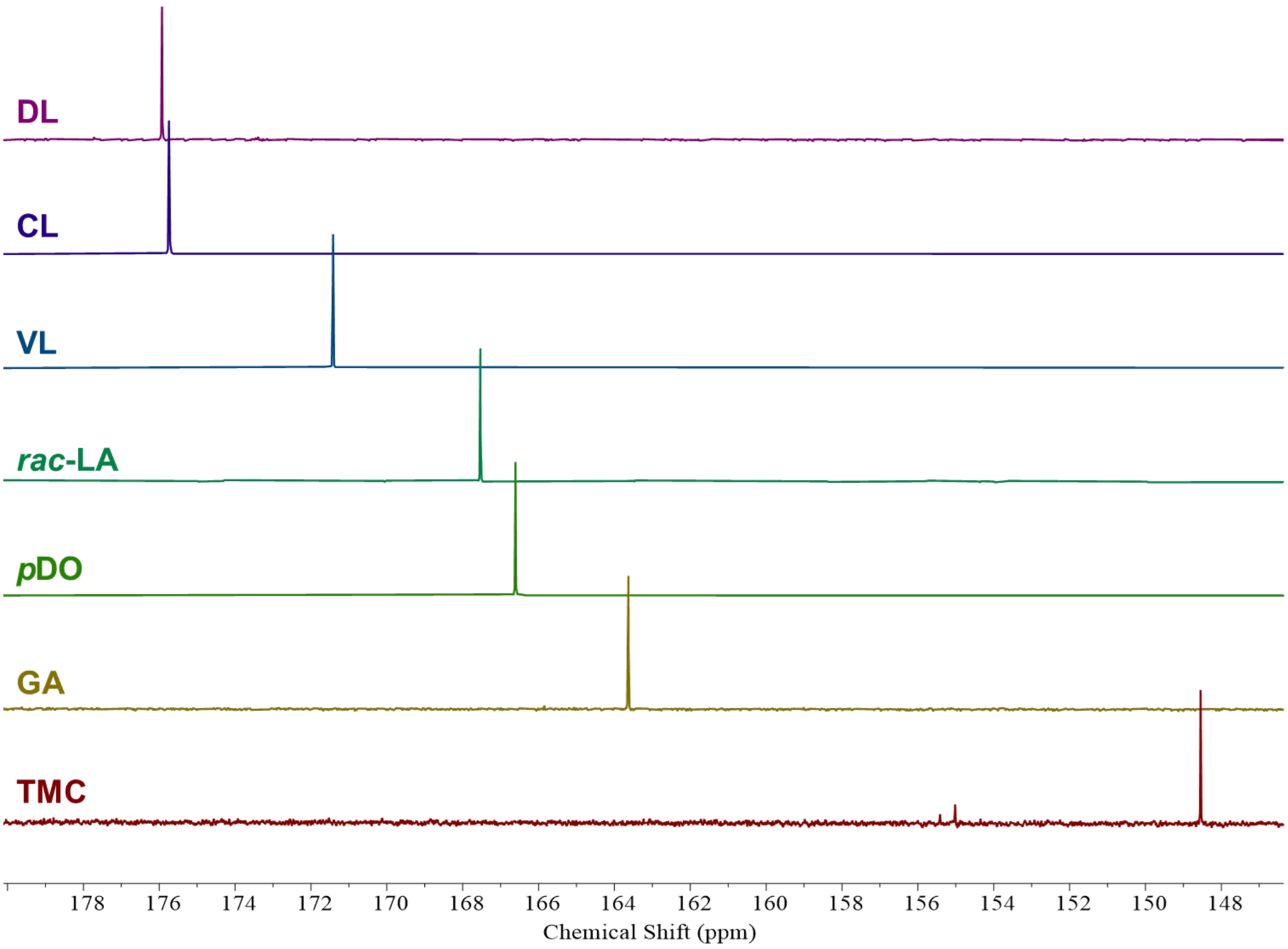

Figure S46. Stacked plot of ^{13}C NMR spectra (126 MHz, $CDCl_3$) for the monomers considered in this work.

Table S9. Comparison of literature examples of Zn–O bond lengths (Å) for a zinc salophen complex upon coordination of various donors.

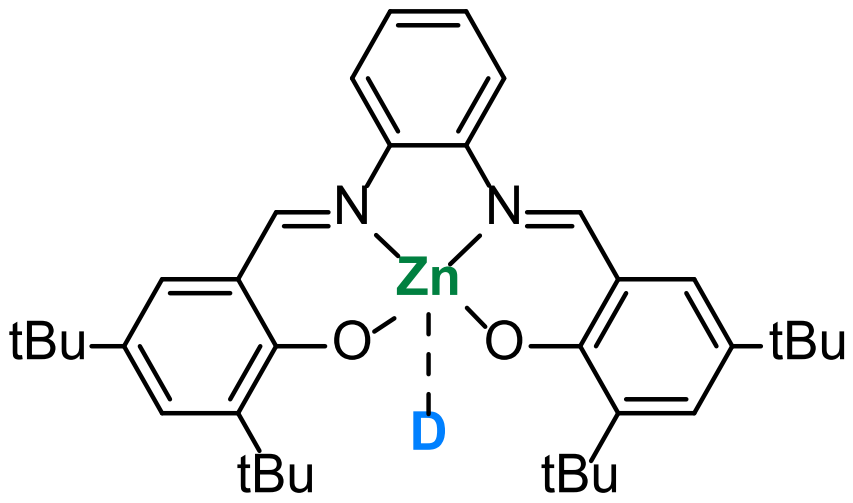

Entry	Donor (D)	Zn – O bond length (Å)[a]
1	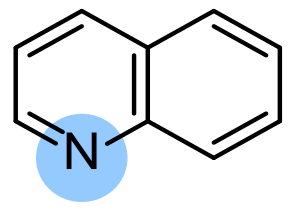	1.982 – 1.985
2	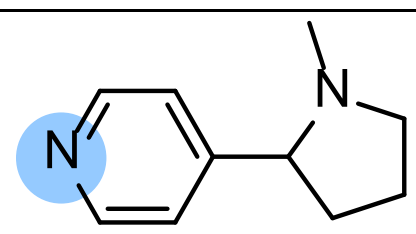	1.966 – 1.983
3	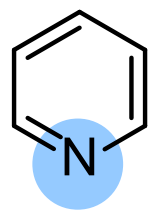	1.979
4	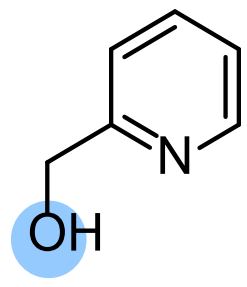	1.942 – 1.967
5	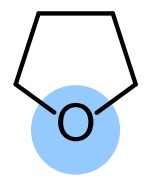	1.941 – 1.942

a – taken from crystal data obtained in references.[24–27]

4. References

[1] W. Gruszka, L. C. Walker, M. P. Shaver, J. A. Garden, “In Situ Versus Isolated Zinc Catalysts in the Selective Synthesis of Homo and Multi-block Polyesters” *Macromolecules* **2020**, *53*, 4294–4302.

[2] J. Kasperczyk, “Copolymerization of Glycolide and ε-Caprolactone, 1. Analysis of the Copolymer Microstructure by Means of ^{1}H and ^{13}C NMR Spectroscopy” *Macromol. Chem. Phys.* **1999**, *200*, 903–910.

[3] J. Fernández, A. Etxeberria, J. R. Sarasua, “Synthesis, Structure and Properties of Poly(L-lactide-co-ε-caprolactone) Statistical Copolymers” *J. Mech. Behav. Biomed. Mater.* **2012**, *9*, 100–112.

[4] T. J. Neal, E. D. Neal, J. Cumby, J. A. Garden, “Exploiting Controlled Transesterification as a ‘Top Down’ Approach to Tailor Poly(ε-caprolactone)-Poly(lactic acid) Copolymer Structures with bis-Zn Catalysts” *Polym. Chem.* **2024**, *15*, 1704–1713.

[5] S. Kamavichanurat, K. Jampakaew, P. Hormnirun, “Controlled and Effective Ring-Opening (Co)polymerization of rac-Lactide, ε-Caprolactone and ε-Decalactone by β-Pyrimidyl Enolate Aluminum Complexes” *Polym. Chem.* **2023**, *14*, 1752–1772.

[6] S. Thongkham, J. Monot, B. Martin-Vaca, D. Bourissou, “Simple In-Based Dual Catalyst Enables Significant Progress in ε-Decalactone Ring-Opening (Co)polymerization” *Macromolecules* **2019**, *52*, 8103–8113.

[7] P. Olsén, T. Borke, K. Odelius, A.-C. Albertsson, “ε-Decalactone: A Thermoresilient and Toughening Comonomer to Poly(l-lactide)” *Biomacromolecules* **2013**, *14*, 2883–2890.

[8] H. Zhao, T. Wang, X. Zhao, Y. Liu, J. Hao, “Synthesis and Properties of Poly(d,l-lactide-co-p-dioxanone) Random and Segmented Copolymers” *J. Polym. Environ.* **2013**, *21*, 405–414.

[9] T. Wang, H. Zhao, Y. Liu, J. Hao, “Correlation of Chemical Composition and Microstructure with Properties of Poly(ε-caprolactone-co-p-dioxanone) Random Copolymers” *J. Appl. Polym. Sci.* **2013**, *130*, 2978–2986.
[10] P. Dobrzynski, J. Kasperczyk, “Synthesis of Biodegradable Copolymers with Low-toxicity Zirconium Compounds. V. Multiblock and Random Copolymers of L-lactide with Trimethylene Carbonate Obtained in Copolymerizations Initiated with Zirconium(IV) Acetylacetonate” *J. Polym. Sci. Part Polym. Chem.* **2006**, *44*, 3184–3201.
[11] M. Pastusiak, P. Dobrzynski, J. Kasperczyk, M. Sobota, B. Kaczmarczyk, H. Janeczek, “Synthesis of Trimethylene Carbonate/ϵ-Caprolactone Copolymers Initiated with Zinc Alkoxide: Influence of Copolymer Chain Microstructure on Thermal and Mechanical Properties” *Polym. Int.* **2017**, *66*, 1259–1268.
[12] J. Kasperczyk, “Microstructural Analysis of Poly[(l,l-lactide)-*co*-(glycolide)] by ^{1}H and ^{13}C NMR Spectroscopy” *Polymer* **1996**, *37*, 201–203.
[13] R. M. Abdur, B. Mousavi, H. M. Shahadat, N. Akther, Z. Luo, S. Zhuiykov, F. Verpoort, “Ring-opening Copolymerization of ε-Caprolactone and δ-Valerolactone by a Titanium-based Metal–organic Framework” *New J. Chem.* **2021**, *45*, 11313–11316.
[14] M. Milic, K. Targos, M. Tellez Chavez, M. A. M. Thompson, J. J. Jennings, A. K. Franz, “NMR Quantification of Hydrogen-Bond-Accepting Ability for Organic Molecules” *J. Org. Chem.* **2021**, *86*, 6031–6043.
[15] G. Kister, G. Cassanas, M. Vert, “Morphology of Poly(glycolic acid) by IR and Raman Spectroscopies” *Spectrochim. Acta. A. Mol. Biomol. Spectrosc.* **1997**, *53*, 1399–1403.
[16] W. Almustafa, D. W. Schubert, S. Grishchuk, J. Sebastian, G. Grun, “Chemical Synthesis of Atactic Poly-3-hydroxybutyrate (a-P3HB) by Self-Polycondensation: Catalyst Screening and Characterization” *Polymers* **2024**, *16*, 1655.
[17] L. Yao, Y. Li, Y. Li, Y. Wang, L. Wang, D. Qiu, Y. Weng, “Surface Modification of Polylactide Films and their Hydrolytic Degradation Performances in Artificial Seawater” *J. Appl. Polym. Sci.* **2024**, *141*, e55999.
[18] F. Robles-González, T. Rodríguez-Hernández, A. S. Ledezma-Pérez, R. Díaz de León, M. A. De Jesús-Téllez, H. R. López-González, “Development of Biodegradable Polyesters: Study of Variations in Their Morphological and Thermal Properties through Changes in Composition of Alkyl-Substituted (ε-DL) and Non-Substituted (ε-CL, EB, L-LA) Monomers” *Polymers* **2022**, *14*, 4278.
[19] J. S. Lyu, J.-S. Lee, J. Han, “Development of a Biodegradable Polycaprolactone Film Incorporated with an Antimicrobial Agent via an Extrusion Process” *Sci. Rep.* **2019**, *9*, 20236.
[20] K.-K. Yang, X.-L. Wang, Y.-Z. Wang, “Poly(p-dioxanone) and its Copolymers” *J. Macromol. Sci. Part C* **2002**, *42*, 373–398.
[21] W. Liu, M. Wang, Z. Ding, Y. Li, B. Wang, “Modular Access to Aliphatic Polycarbonates with Tunable Properties and Dual Closed-Loop Recyclability by Polycondensation–Depolymerization–Repolymerization Strategy” *Angew. Chem. Int. Ed.* **2025**, *64*, e202505333.
[22] W. S. Saeed, A.-B. Al-Odayni, A. Ali Alghamdi, A. Abdulaziz Al-Owais, A. Semlali, T. Aouak, “Miscibility of Poly(Ethylene-co-Vinylalcohol)/Poly(δ-Valerolactone) Blend and Tissue Engineering Scaffold Fabrication Using Naphthalene as Porogen” *Polym. Plast. Technol. Mater.* **2019**, *58*, 1–23.
[23] P. J. Hocking, R. H. Marchessault, “Microstructure of Poly[(R,S)-.beta.-hydroxybutyrate] by ^{13}C NMR” *Macromolecules* **1995**, *28*, 6401–6409.
[24] M. E. Germain, T. R. Vargo, P. G. Khalifah, M. J. Knapp, “Fluorescent Detection of Nitroaromatics and 2,3-Dimethyl- 2,3-dinitrobutane (DMNB) by a Zinc Complex: (salophen)Zn” *Inorg. Chem.* **2007**, *46*, 4422–4429.
[25] E. C. Escudero-Adán, J. Benet-Buchholz, A. W. Kleij, “Supramolecular Adsorption of Alkaloids by Metallosalphen Complexes” *Inorg. Chem.* **2008**, *47*, 4256–4263.
[26] S. J. Wezenberg, E. C. Escudero-Adán, J. Benet-Buchholz, A. W. Kleij, “Colorimetric Discrimination between Important Alkaloid Nuclei Mediated by a Bis-Salphen Chromophore” *Org. Lett.* **2008**, *10*, 3311–3314.
[27] M. Martínez Belmonte, E. C. Escudero-Adán, J. Benet-Buchholz, A. W. Kleij, “Formation of Unusual Trinuclear Assemblies: Scope and Mechanism of Zn(salphen)-Templated Activation of Pyridine-Alcohol Substrates” *Eur. J. Inorg. Chem.* **2009**, *2009*, 5307–5318.
